# Construction of spirocarbocycles *via* gold-catalyzed intramolecular dearomatization of naphthols†
†Electronic supplementary information (ESI) available. See DOI: 10.1039/c5sc04130a


**DOI:** 10.1039/c5sc04130a

**Published:** 2016-02-02

**Authors:** Wen-Ting Wu, Ren-Qi Xu, Liming Zhang, Shu-Li You

**Affiliations:** a State Key Laboratory of Organometallic Chemistry , Shanghai Institute of Organic Chemistry , Chinese Academy of Sciences , 345 Lingling Lu , Shanghai 200032 , China . Email: slyou@sioc.ac.cn; b University of California , Santa Barbara , California 93106 , USA . Email: zhang@chem.ucsb.edu

## Abstract

A highly efficient, gold-catalyzed intramolecular dearomatization reaction of naphthols *via* 5-*endo-dig* cyclization is described. This facile and direct approach furnishes spirocarbocycles in excellent yields under mild conditions.

## Abstract 2

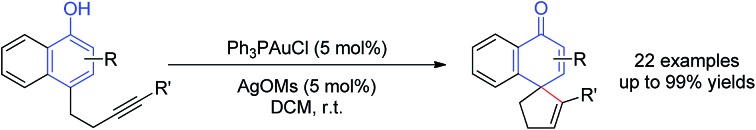
A highly efficient, gold-catalyzed intramolecular dearomatization reaction of naphthols *via* 5-*endo-dig* cyclization is described. This facile and direct approach furnishes spirocarbocycles in excellent yields under mild conditions.

## 

Spirocarbocycles have captured the close attention of organic chemists due to their unique structural characteristics, including fully substituted carbon centers. Moreover, spirocarbocycles often appear in diverse natural products and biologically active molecules (Fig. 1).^1^ Given the distinctive properties of spirocarbocycles, especially the relatively congested quaternary carbon center, it has been a challenging task for chemists to develop a synthetically applicable methodology for a long time, until recent progress in organometallic catalysis.^2^ However, highly efficient, mild and streamlined synthetic routes are still in great demand.

**Fig. 1:**
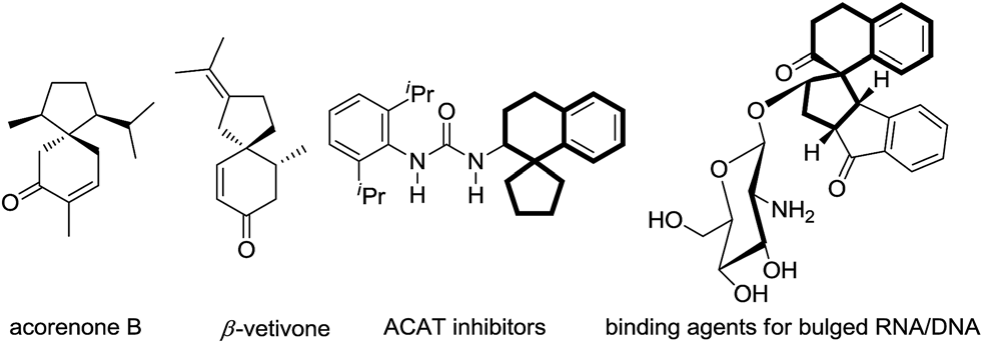
Examples of natural products and biologically active compounds containing spirocarbocyclic backbones.

Meanwhile, gold catalysis has exhibited remarkable capacity for the construction of complex molecules since the new millennium.^3^ The potent soft Lewis acidity of cationic gold(i) complexes enables, upon coordination, efficient attack on alkynes and alkenes by various nucleophiles, thereby leading to the formation of functional products including spirocarbocycles. Despite remarkable progress, there are relatively few reports on the application of gold catalysis in dearomatization reactions.^4^,^5^ Particularly, dearomatization reactions of phenol and derivatives have been much under-developed despite potential direct access to highly functionalized spirocarbocycles.^6^,^7^ In this regard, Hamada and coworkers recently reported an elegant gold-catalyzed 5-*exo-dig* carbocyclization of phenols in the presence of methanesulfonic acid and 2,6-di-*tert*-butylpyridine.6*b* Interestingly, we found 5-*endo-dig* cyclization products could be selectively obtained *via* gold-catalyzed dearomatizations of naphthols under mild conditions. Herein, we report our findings.

We began our investigation by testing 1-naphthol derivative **1a** with commercially available gold complex Ph_3_PAuCl and various chloride scavengers. The results are summarized in Table 1. To our delight, in the presence of Ph_3_PAuCl (5 mol%) and NaBARF (tetrakis[3,5-bis(trifluoromethyl)phenyl]boron sodium) (5 mol%), the gold-catalyzed dearomatization reaction of **1a** (0.1 mmol) in DCM (1.0 mL) at room temperature proceeded smoothly to afford the desired spirocarbocyclic product **2a** in 72% yield (100% yield based on conversion) in 5 h, albeit with incomplete conversion (entry 1, Table 1). However, other chloride scavengers such as AgOTf, AgNTf_2_, and Cu(OTf)_2_ (entries 2–4, Table 1) led to little desired product despite nearly complete substrate consumption. In the presence of AgNTf_2_, the tricycle product **2aa** was isolated in 86% yield. Interestingly, AgOMs was found to be the best chloride scavenger, and the reaction proceeded to completion in 5 h, affording **2a** in excellent yield (>95% NMR yield, entry 5, Table 1). Next, solvent screening was carried out. Among the solvents evaluated, toluene and THF (entries 6 and 7, Table 1) led to slow reactions while no desired product **2a** was detected in MeOH (entry 8, Table 1). As expected, Ph_3_PAuCl, AgOMs, or HOMs alone could not catalyze this dearomative spirocylization (entries 9–11, Table 1), suggesting that the combination of Ph_3_PAuCl and AgOMs is essential for this reaction. Ph_3_PAuOMs is most likely the catalyst. Indeed, when Ph_3_PAuOMs (entry 12, Table 1) was prepared in pure form and used as the catalyst, the reaction outcome was identical to that when the catalyst was prepared *in situ* (entry 5). Moreover, the reaction could be conducted open-flask without erosion of the yield (entry 13, Table 1).

**Table 1:** Optimization of reaction conditions^*a*^

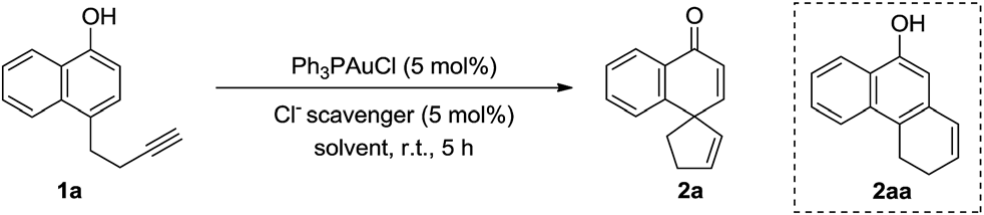				
Entry	Cl^–^ scavenger	Solvent	Conversion^*b*^ (%)	Yield^*b*^ (%)
1	NaBARF	DCM	72	72
2	AgOTf	DCM	>95	<5
3^*c*^	AgNTf_2_	DCM	>95	<5
4^*d*^	Cu(OTf)_2_	DCM	>95	<5
5	AgOMs	DCM	>95	>95 (96)
6	AgOMs	Toluene	42	42
7	AgOMs	THF	10	10
8	AgOMs	MeOH	<5	<5
9^*e*^	AgOMs	DCM	<5	<5
10	—	DCM	<5	<5
11^*f*^	—	DCM	<5	<5
12^*g*^	—	DCM	>95	>95 (92)
13^*h*^	AgOMs	DCM	>95	>95 (96)

^*a*^Reaction conditions: **1a** (0.1 mmol), Ph_3_PAuCl (5 mol%), Cl^–^ scavenger (5 mol%) in 1.0 mL solvent, r.t, 5 h.

^*b*^Determined by ^1^H NMR using CH_2_Br_2_ (0.1 mmol) as internal standard; isolated yield in parentheses.

^*c*^ **2aa** was isolated in 86% yield.

^*d*^2.5 mol% of Cu(OTf)_2_ was added.

^*e*^Reaction was performed without Ph_3_PAuCl.

^*f*^Reaction was performed with HOMs (5 mol%) instead of Ph_3_PAuCl.

^*g*^Reaction was performed with Ph_3_PAuOMs (5 mol%).

^*h*^Reaction was performed open-flask.

Under the above optimized reaction conditions, we then explored the substrate scope of this reaction. The results are summarized in Scheme 1. For substrates bearing terminal alkyne pendants, the dearomatization reactions all proceeded well, delivering the corresponding spirocarbocyclic products **2a**, **2b** and **2c** in satisfactory yields (**2a**, 96% yield; **2b**, 99% yield; **2c**, 98% yield). Despite the fact that the reaction of a substrate with a 2-methyl group on the naphthyl ring was sluggish (**2d**, 44% yield, 44% conversion after 34 h), the yield of **2d** could be improved to 79% with the addition of 10 mol% catalyst in 2 portions. Next, various substrates bearing internal alkyne tethers were examined. Pleasingly, halogen-substituted alkynes did not interfere with the gold-catalyzed cyclization and the corresponding products (Br, **2e**; I, **2f**) could be obtained in nearly quantitative yields within 20 minutes. In addition, when phenyl-substituted alkyne substrate **1g** was subjected to the reaction conditions, the reaction was completed even faster, delivering spirocarbocyclic product **2g** in 95% yield in 10 min. Moreover, different electron-donating groups (**2h**, MeO; **2i**, Me) or electron-withdrawing groups (**2j**, F; **2k**, Cl; **2l**, Br; **2m**, CN; **2n**, CO_2_Me) on the *para*-position of the phenyl ring were compatible with this reaction, and the corresponding spirocarbocyclic products were formed in superb yields (97–99%). Likewise, a methyl or methoxyl group at either the *ortho*- or *meta*-position on the phenyl ring had little influence on the reaction, and the spirocarbocyclic products **2o** and **2p** were isolated in 99% and 96% yields, respectively. It is worth mentioning that substrates with other aromatic rings attached to the alkyne moiety also underwent the spirocyclization smoothly, affording spirocyclic products containing 1-naphthyl (**2q**), 5-indolyl (**2r**), or 2-thienyl (**2s**) motifs again in excellent yields (91–98%), and even pyridine-containing substrate **1t** was converted into the corresponding spirocarbocyclic product **2t** in 99% yield with an additional 1.5 equivalents of HOMs. Additionally, substrate **1u** bearing a 3-OMe substituent was also compatible with this reaction, delivering **2u** in 86% yield. When the linker was further extended, 5-*exo*-cyclization product **2v** was observed in 99% yield. Simple phenolic substrates **1w** and **1x** were unreactive under the standard reaction conditions.

**Scheme 1:**
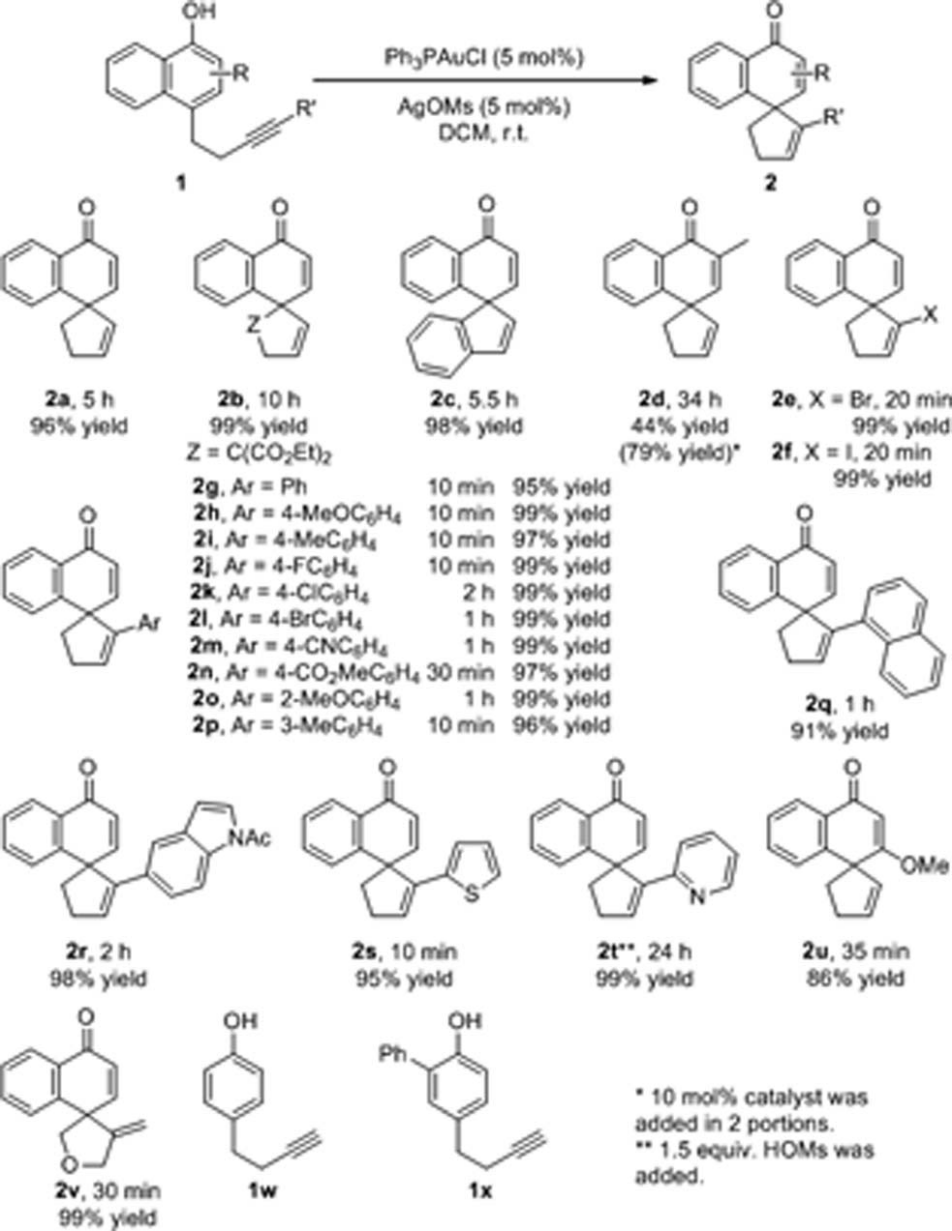
Substrate scope. Reaction conditions: **1** (0.2 mmol), Ph_3_PAuCl (5 mol%), AgOMs (5 mol%) in 2.0 mL DCM, r.t.

To test the practicality of this new methodology, a gram-scale reaction was conducted. As shown in Scheme 2, only 0.05 mol% of Ph_3_PAuOMs was required to accomplish the dearomatization reaction to give spirocarbocyclic compound **2g** in 99% yield after 2 hours. This level of efficiency in gold catalysis has only been observed in limited reports.^8^

**Scheme 2:**
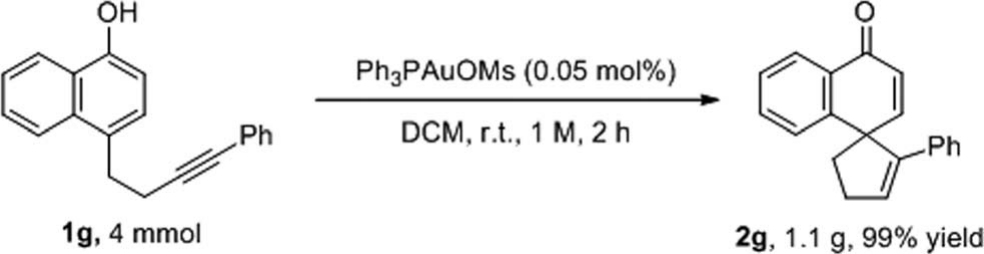
Gram-scale reaction.

To illustrate the synthetic utility of this methodology, transformations of product **2f** were conducted. The vinyl iodine moiety readily underwent the Sonogashira coupling reaction with ethynyltrimethylsilane to afford enyne **3f** in 99% yield. In addition, **2f** smoothly participated in radical reactions.^9^ Treatment with phenyl vinyl sulfone in the presence of Bu_3_SnH and AIBN afforded the polycyclic spiro-product **4f** with considerable molecular complexity in 55% yield (Scheme 3).

**Scheme 3:**
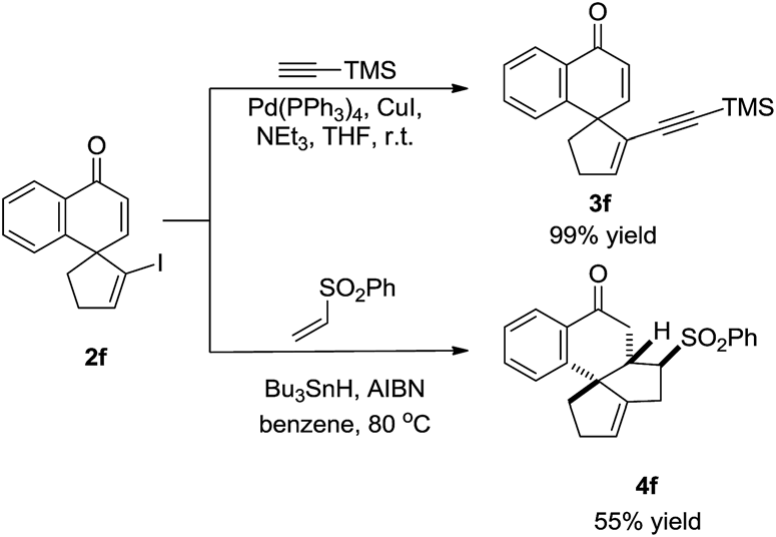
Transformations of product **2f**.

A preliminary attempt at the asymmetric reaction revealed that good enantioselectivity (90% ee) could be achieved in the presence of a catalytic amount of (4-CF_3_C_6_H_4_)_3_PAuCl and chiral silver phosphate ((*S*)-TRIP-CPA-Ag),^10^ while moderate enantiocontrol (26% ee) was obtained by using (*R*)-BINAP(AuCl)_2_ (Scheme 4).

**Scheme 4:**
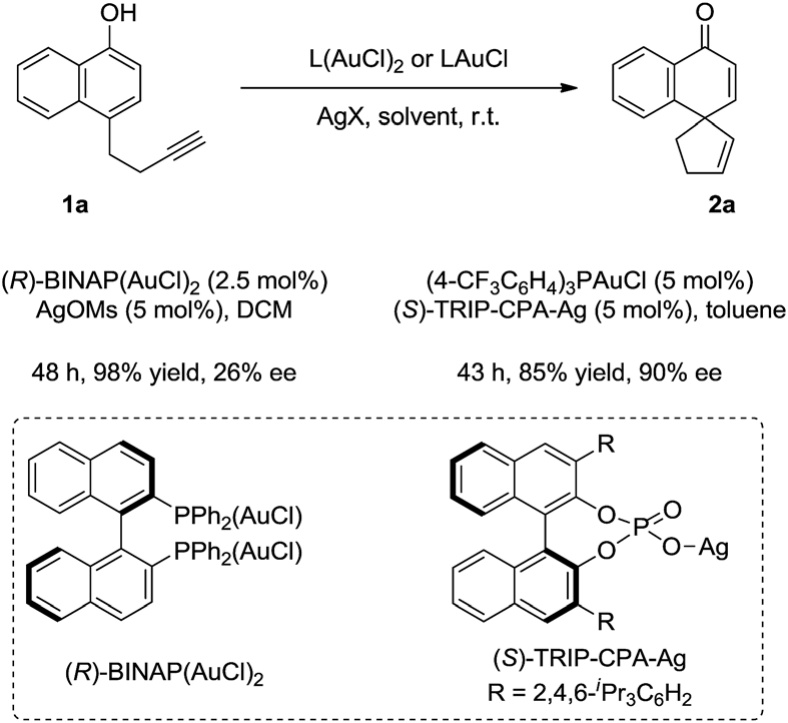
Preliminary results of the asymmetric reaction.

A catalytic cycle is proposed in Scheme 5. The *in situ* generated cationic gold(i) complex coordinates with and activates the C–C triple bond in **1**, and the subsequent 5-*endo-dig* cyclization is facilitated by the concomitant deprotonation by the counter anion MsO^–^, directly yielding the spirocyclic gold intermediate **A**. Alternatively, this cyclization might follow a typical 1,5-enyne cycloisomerization route to afford a cyclopropyl gold carbene intermediate **B**, which in turn would undergo MsO^–^-promoted deprotonative fragmentation to arrive at the same intermediate. Although with relatively basic MsO^–^ as the counter anion the direct route is more likely, the intermediacy of **B** in the step-wise route offers a straightforward rationale for the formation of **2aa**, where in the presence of less basic NTf_2_^–^ the fragmentation of the bold bond of the cyclopropane ring in **B** is preferred over the depicted deprotonative fragmentation. Protodemetallation of **A** by the *in situ* generated MsOH then delivers the desired spirocarbocyclic product **2** while regenerating the active gold catalyst (Scheme 5).

**Scheme 5:**
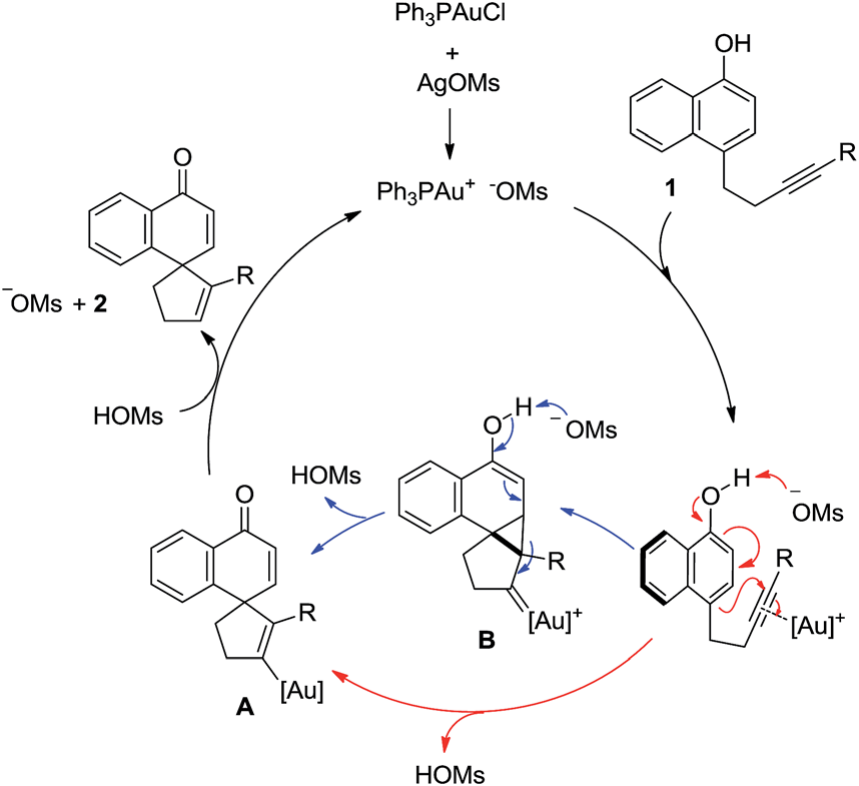
A proposed catalytic cycle.

## Conclusions

In conclusion, we have realized a highly efficient and straightforward construction of spirocarbocycles *via* the gold-catalyzed dearomatization reaction of naphthols under mild reaction conditions. The employment of commercially available catalysts and the compatibility with reaction scale-up and low catalyst loading point to the potential synthetic application of this methodology.

## Supplementary Material

Supplementary information
