# Supplementary material for: Construction of spirocarbocycles via gold-catalyzed intramolecular dearomatization of naphthols
Source: Chem Sci. 2016 Feb 2;7(5):3427–31. doi: 10.1039/c5sc04130a (PMC6006865; doi:10.1039/c5sc04130a)
Supplement: Supplementary file 1 [file SC-007-C5SC04130A-s001.pdf]

Electronic Supplementary Material (ESI) for Chemical Science.
This journal is © The Royal Society of Chemistry 2016

Supporting Information for

Construction of Spirocarbocycles *via* Gold-Catalyzed Intramolecular Dearomatization of Naphthols

Wen-Ting Wu, Ren-Qi Xu, Liming Zhang*, and Shu-Li You*

State Key Laboratory of Organometallic Chemistry, Shanghai Institute of Organic Chemistry, Chinese Academy of Sciences, 345 Lingling Lu, Shanghai 200032, China

E-mail: slyou@sioc.ac.cn

Department of Chemistry & Biochemistry, University of California, Santa Barbara California 93106, United States

E-mail: zhang@chem.ucsb.edu

Table of Contents

General methods	S2
General procedure for the preparation of 1-naphthol derivatives (**1a-1x**)	S3-S17
General procedure for gold-catalyzed dearomatization of naphthols	S17-S27
Gram-scale reaction of **1g** and transformations of **2f**	S27-S29
Gold-catalyzed asymmetric dearomatization of naphthols and HPLC chromatographs	S29-S34
Copies of NMR spectra	S35-S139
References	S140

General Methods. Unless stated otherwise, all reactions were carried out in flame-dried glassware under a dry argon atmosphere. All solvents were purified and dried according to standard methods prior to use. ^{1}H spectra were recorded on a Varian (300 MHz or 400 MHz) or Agilent instrument (400 MHz) and internally referenced to tetramethylsilane signal or residual solvent signals. ^{13}C NMR spectra were recorded on a Varian (100 MHz or 75 MHz) or Agilent instrument (100 MHz) and internally referenced to residual solvent signals. ^{19}F NMR spectra were recorded on a Varian or Agilent instrument (376 MHz) and referenced relative to $CFCl_3$. Data for ^{1}H NMR are recorded as follows: chemical shift (δ, ppm), multiplicity (s = singlet, d = doublet, t = triplet, m = multiplet or unresolved, brs = broad singlet, coupling constant (s) in Hz, integration). Data for ^{13}C NMR and ^{19}F NMR are reported in terms of chemical shift (δ, ppm).

General procedure for the preparation of 1-naphthol derivatives (1a-1t)

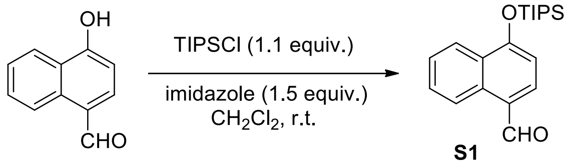

To a solution of 4-hydroxy-1-naphthaldehyde[1] (14.15 g, 82 mmol) and imidazole (8.40 g, 123 mmol) in DCM (100 mL), triisopropylsilyl chloride (TIPSCl, 20 mL, 90 mmol) was added dropwise and the mixture was stirred at room temperature. After completion (monitored by TLC), the reaction mixture was quenched with saturated NH_4Cl (100 mL) and extracted with DCM (50 mL x 3). The combined DCM extract was washed with brine, dried over anhydrous Na_2SO_4 and filtrated. After the solvent was concentrated under reduced pressure, the crude product was purified by recrystallization (PE) and **S1** was obtained in 77% yield (22.60 g).

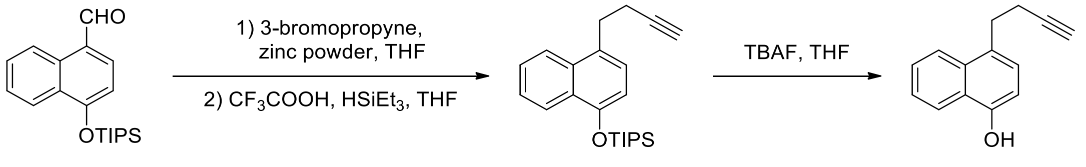

To a dry two-necked, round-bottomed flask containing a solution of **S1** (3.28 g, 10 mmol) in THF (50 mL), activated zinc powder (845.0 mg, 13 mmol) was added and 3-bromopropyne (1.65 g, 14 mmol) was added dropwise under argon. The mixture was stirred at room temperature. After completion (monitored by TLC), the reaction mixture was quenched with saturated NH_4Cl (50 mL). THF was removed under reduced pressure. The mixture was extracted with EtOAc (50 mL x 3). The combined EtOAc extract was washed with brine, dried over anhydrous Na_2SO_4 and filtrated. After the solvent was concentrated under reduced pressure, the crude product was dissolved in DCM (100 mL) and the mixture was cooled at 0^oC. After addition of $HSiEt_3$ (2.33 g, 20 mmol), CF_3COOH (2.28 g, 20 mmol) was added dropwise. The mixture was warmed up to room temperature and stirred. After completion (monitored by TLC), the reaction mixture was quenched with saturated $NaHCO_3$ (100 mL) and extracted with DCM (50 mL x 3). The combined DCM extract was washed with brine, dried over anhydrous Na_2SO_4 and filtrated. After the solvent was concentrated under reduced pressure, the crude product **S2** was dissolved in THF (10 mL) and the

mixture was cooled at 0°C. Tetrabutylammonium fluoride (TBAF) (7.7 mL, 1M in THF, 7.7 mmol) was added dropwise and the mixture was stirred at 0°C for 10 min. After completion (monitored by TLC), the reaction mixture was quenched with H_2O (2 mL) and THF was removed under reduced pressure. The mixture was extracted with EtOAc (5 mL x 3). The combined EtOAc extract was washed with brine, dried over anhydrous Na_2SO_4 and filtrated. After the solvent was concentrated under reduced pressure, the crude product was purified by silica gel column chromatography (PE/EtOAc = 10/1) and **1a** was obtained in 61% yield (674.9 mg, 3.4 mmol).

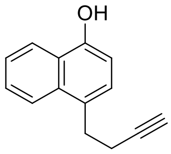

1a

White solid. M.P. = 59-61 °C. ^1H NMR (400 MHz, $CDCl_3$) δ 8.27 (d, *J* = 8.0 Hz, 1H), 7.98 (d, *J* = 8.4 Hz, 1H), 7.63-7.46 (m, 2H), 7.21 (d, *J* = 7.6 Hz, 1H), 6.75 (d, *J* = 8.0 Hz, 1H), 5.52 (s, 1H), 3.27 (t, *J* = 7.6 Hz, 2H), 2.62 (td, *J* = 7.2, 2.4 Hz, 2H), 2.08 (t, *J* = 2.8 Hz, 1H). ^{13}C NMR (100 MHz, $CDCl_3$) δ 150.3, 132.5, 128.8, 126.5, 126.0, 124.9, 124.7, 123.4, 122.4, 108.1, 84.12, 69.2, 31.6, 20.0. IR (thin film): ν_{max} (cm^{-1}) = 3670, 3280, 2920, 2113, 1646, 1586, 1514, 1437, 1382, 1336, 1277, 1252, 1143, 1047, 976, 833, 814, 762, 658, 637; HRMS (EI) calcd for $C_{14}H_{12}O$ $[M]^+$: 196.0888. Found: 196.0892.

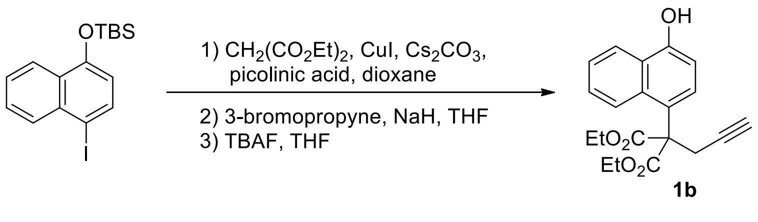

To a dry two-necked, round-bottomed flask containing a solution of 1-(*t*-butyldimethylsiloxy)-4-iodonaphthalene[2] (7.69 g, 20 mmol), CuI (1.52 g, 8 mmol), picolinic acid (1.97 g, 16 mmol), and Cs_2CO_3 (19.55 g, 60 mmol) in dioxane (20 mL), diethyl malonate (6.41 g, 20 mmol) was added dropwise under argon[3]. After completion (monitored by TLC), the reaction mixture was quenched with saturated NH_4Cl (100 mL). dioxane was removed under reduced pressure. The

mixture was extracted with EtOAc (50 mL x 3). The combined EtOAc extract was washed with brine, dried over anhydrous Na_2SO_4 and filtrated. After the solvent was concentrated under reduced pressure, the crude product was dissolved in THF (20 mL). The solution was cooled at 0 °C and NaH (60% in mineral, 40.2 mg, 1.68 mmol) was added under argon. After the mixture was stirred for 1 h, 3-bromopropyne (2.31 g, 1.94 mmol) was added dropwise. The mixture was stirred at room temperature. After completion (monitored by TLC), the reaction mixture was quenched with saturated NH_4Cl (50 mL) and THF was removed under reduced pressure. The mixture was extracted with EtOAc (50 mL x 3). The combined EtOAc extract was washed with brine, dried over anhydrous Na_2SO_4 and filtrated. After the solvent was concentrated under reduced pressure, the crude product was used directly without further purification. Analogous to **1a**, **1b** was obtained in 58% yield for 2 steps after the treatment of TBAF.

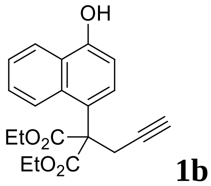

1b

White solid. M.P. = 89-90 °C. ^{1}H NMR (400 MHz, $CDCl_3$) δ 8.22 (d, *J* = 8.0 Hz, 1H), 7.82 (d, *J* = 8.4 Hz, 1H), 7.46-7.35 (m, 2H), 7.22 (d, *J* = 8.4 Hz, 1H), 6.73 (brs, 1H), 6.52 (d, *J* = 8.0 Hz, 1H), 4.33-4.21 (m, 4H), 3.43 (d, *J* = 1.6 Hz, 2H), 2.02 (s, 1H), 1.18 (t, *J* = 7.2 Hz, 6H). ^{13}C NMR (100 MHz, $CDCl_3$) δ 170.6, 152.4, 132.0, 126.2, 126.1, 125.3, 124.4, 124.0, 122.8, 107.4, 79.8, 71.4, 62.3, 62.1, 26.9, 13.7. IR (thin film): ν_{max} (cm^{-1}) = 3386, 3295, 2978, 1730, 1712, 1626, 1585, 1518, 1441, 1349, 1294, 1243, 1202, 1151, 1090, 1046, 1026, 1006, 957, 857, 836, 807, 763, 720, 677, 642; HRMS (ESI) calcd for $C_{20}H_{21}O_5$ $[M+H]^+$: 341.1384. Found: 341.1382.

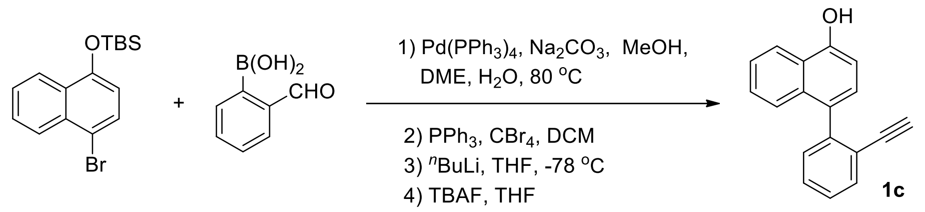

A flame-dried two-necked, round-bottomed flask was cooled down to room temperature under argon. To this flask were added $Pd(PPh_3)_4$ (750.0 mg, 0.65 mmol), Na_2CO_3 (2.80 g, 26.4 mmol), DME (30 mL), MeOH (10 mL) and H_2O (10 mL). The reaction mixture was stirred at room temperature for 10 min. Then (4-bromonaphthalen-1-yloxy)(tert-butyl)dimethylsilane[4] (5.00 g, 13.2 mmol) and (2-formylphenyl)boronic acid (2.20 g, 14.5 mmol) were added. The resulting solution was heated to reflux. After completion (monitored by TLC), the reaction mixture was filtrated through celite and the solvent was removed under reduced pressure. The crude product was used without further purification.

A flame-dried two-necked, round-bottomed flask was cooled down to room temperature under argon. After CBr_4 (2.76 g, 8.32 mmol) was dissolved in DCM (25 mL), PPh_3 (4.36 g, 16.64 mmol) was added at 0^oC. Then the above crude product in 10 mL DCM was added dropwise and the solution was warmed up to room temperature. After completion (monitored by TLC), the reaction mixture was quenched with saturated $NaHCO_3$ (100 mL) and extracted with DCM (10 mL x 3). The combined DCM extract was washed with brine, dried over anhydrous Na_2SO_4 and filtrated. After the solvent was concentrated under reduced pressure, the crude product was used without further purification.

A flame-dried two-necked, round-bottomed flask was cooled down to room temperature under argon. To a solution of the above crude product in THF (30 mL) at -78^oC, ^{n}BuLi (2.75 mL, 6.6 mmol, 2.4 M in hexane) was added dropwise. After completion (monitored by TLC), the reaction mixture was quenched with saturated NH_4Cl (30 mL) and extracted with EtOAc (10 mL x 3). The combined EtOAc extract was washed with brine, dried over anhydrous Na_2SO_4 and filtrated. After the solvent was concentrated under reduced pressure, the crude product was used without further purification. Following the previous procedure by treatment of TBAF, **1c** was obtained in 51% yield (372.0 mg, 1.52 mmol) for 2 steps.

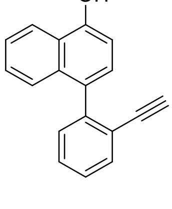

1c

Brown solid. M.P. = 130-131 ^oC. ^1H NMR (400 MHz, $CDCl_3$) δ 8.26 (d, J = 8.4 Hz, 1H), 7.67 (d, J = 8.4 Hz, 1H), 7.57 (d, J = 8.5 Hz, 1H), 7.50 (t, J = 6.8 Hz, 1H),

7.46-7.43 (m, 2H), 7.41-7.34 (m, 2H), 7.29 (d, *J* = 7.6 Hz, 1H), 6.88 (d, *J* = 8.0 Hz, 1H), 5.38 (s, 1H), 2.78 (s, 1H). ^{13}C NMR (100 MHz, $CDCl_3$) δ 151.1, 143.4, 133.1, 132.9, 131.2, 131.1, 128.5, 127.2, 127.1, 126.4, 126.1, 125.1, 124.1, 122.6, 121.7, 107.9, 82.8, 80.2. IR (thin film): ν_{max} (cm^{-1}) = 3672, 3477, 3259, 2986, 2902, 2348, 2322, 1940, 1584, 1513, 1405, 1372, 1339, 1246, 1221, 1048, 894, 819, 764, 665, 631; HRMS (EI) calcd for $C_{18}H_{12}O$ $[M]^+$: 244.0888. Found: 244.0887.

1d was prepared following the procedure for the preparation of **1a**.

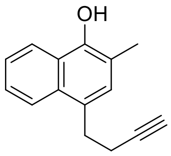

1d

Brown solid. M.P. = 94-95 °C. ^{1}H NMR (400 MHz, $CDCl_3$) δ 8.19-8.17 (m, 1H), 7.95-1.92 (m, 1H), 7.52-7.45 (m, 2H), 7.14 (s, 1H), 5.02 (s, 1H), 3.23 (t, *J* = 7.6 Hz, 2H), 2.58 (td, *J* = 8.0, 2.8 Hz, 2H), 2.40 (s, 3H), 2.03 (t, *J* = 2.0 Hz, 1H). ^{13}C NMR (100 MHz, $CDCl_3$) δ 147.5, 131.1, 129.3, 128.4, 125.4, 125.0, 124.7, 123.3, 121.7, 115.7, 84.1, 69.0, 31.6, 20.1, 15.6. IR (thin film): ν_{max} (cm^{-1}) = 3673, 3418, 3284, 2987, 2901, 2115, 1580, 1511, 1384, 1289, 1244, 1151, 1066, 927, 881, 744, 637; HRMS (EI) calcd for $C_{15}H_{14}O$ $[M]^+$: 210.1045. Found: 210.1040.

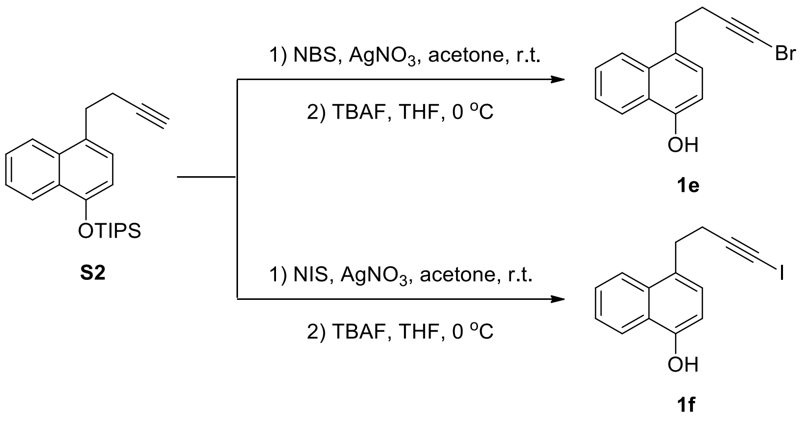

1e and **1f** were prepared from **S2** according to known literature[5] and subsequent removal of the TIPS protection group, and **1e** and **1f** were obtained in 87% and 44% yield for 2 steps, respectively.

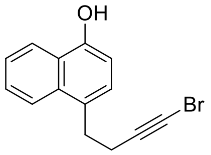

1e

White solid. M.P. = 71-72 °C. ^{1}H NMR (400 MHz, $CDCl_3$) δ 8.24 (d, *J* = 8.0 Hz, 1H), 7.96 (d, *J* = 8.4 Hz, 1H), 7.59-7.48 (m, 2H), 7.18 (d, *J* = 7.6 Hz, 1H), 6.75 (d, *J* = 7.6 Hz, 1H), 5.19-5.14 (m, 1H), 3.24 (t, *J* = 7.6 Hz, 2H), 2.60 (t, *J* = 7.6 Hz, 2H). ^{13}C NMR (100 MHz, $CDCl_3$) δ 150.4, 132.5, 128.7, 126.5, 126.0, 125.0, 124.6, 123.4, 122.4, 108.1, 79.8, 38.9, 31.5, 21.3. IR (thin film): ν_{max} (cm^{-1}) =3303, 3064, 3045, 2926, 2872, 2852, 1946, 1628, 1586, 1515, 1462, 1428, 1398, 1379, 1355, 1282, 1251, 1219, 1185, 1143, 1047, 1018, 975, 815, 759, 741, 619; HRMS (EI) calcd for $C_{14}H_{11}OBr$ $[M]^+$: 273.9993. Found: 273.9997.

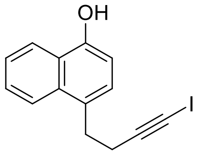

1f

White solid. M.P. = 78-79 °C. ^{1}H NMR (400 MHz, $CDCl_3$) δ 8.29-8.23 (m, 1H), 7.97 (d, *J* = 8.0 Hz, 1H), 7.59-7.50 (m, 2H), 7.18 (d, *J* = 7.6 Hz, 1H), 6.74 (d, *J* = 7.6 Hz, 1H), 5.37 (s, 1H), 3.26 (t, *J* = 7.6 Hz, 2H), 2.81-2.73 (m, 2H). ^{13}C NMR (100 MHz, $CDCl_3$) δ 150.2, 132.4, 128.7, 126.5, 126.0, 124.9, 124.6, 123.3, 122.3, 108.1, 94.1, 31.6, 22.4. IR (thin film): ν_{max} (cm^{-1}) = 3451, 3304, 3064, 3043, 2943, 2924, 2907, 2850, 2186, 1985, 1943, 1915, 1818, 1629, 1586, 1515, 1462, 1428, 1398, 1379, 1354, 1330, 1281, 1251, 1218, 1187, 1143, 1046, 1016, 975, 829, 815, 760, 741, 618; HRMS (EI) calcd for $C_{14}H_{11}OI$ $[M]^+$: 321.9855. Found: 321.9854.

General procedure for the preparation of **1g-1t** (**1g** as an example).

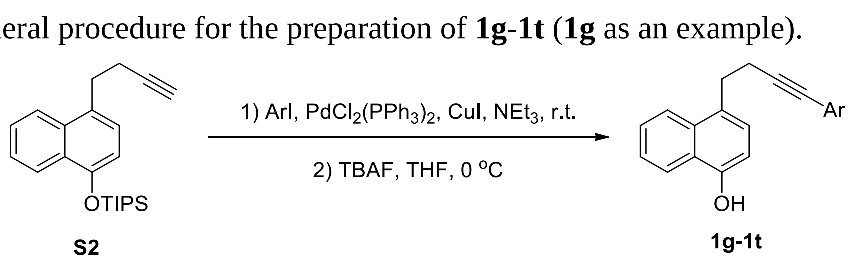

To a flame-dried two-necked, round-bottomed flask at room temperature under argon were added **S3** (6.90 g, 19.6 mmol), $Pd(PPh_3)_2Cl_2$ (701.9 mg, 1.0 mmol), CuI

(571.4 mg, 3.0 mmol), PhI (2.1 mL, 19.6 mmol), and NEt_3 (100 mL). The resulting reaction mixture was stirred at room temperature. After completion (monitored by TLC), the reaction mixture was diluted by EtOAc (100 mL), neutralized by 1M HCl (100 mL) and extracted with EtOAc (50 mL x 3). The combined EtOAc extract was washed with brine, dried over anhydrous Na_2SO_4 and filtrated. After the solvent was concentrated under reduced pressure, the crude product was used without further purification. Following the previous procedure on removing the TIPS group by the treatment of TBAF, **1g** was obtained in 66% yield (3.52 g) for 2 steps.

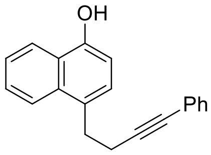

1g

White solid. 66% yield for 2 steps. M.P. = 82-83 °C. ^{1}H NMR (400 MHz, $CDCl_3$) δ 8.24 (d, *J* = 8.4 Hz, 1H), 8.04 (d, *J* = 8.4 Hz, 1H), 7.58-7.49 (m, 2H), 7.38-7.36 (m, 2H), 7.29-7.25 (m, 4H), 6.77 (d, *J* = 7.6 Hz, 1H), 5.16 (d, *J* = 6.8 Hz, 1H), 3.33 (t, *J* = 7.6 Hz, 2H), 2.80 (t, *J* = 8.0 Hz, 2H). ^{13}C NMR (100 MHz, $CDCl_3$) δ 150.3, 132.7, 131.5, 129.2, 128.2, 127.6, 126.5, 126.2, 124.9, 124.7, 123.8, 123.6, 122.3, 108.1, 89.7, 81.5, 31.9, 21.2. IR (thin film): ν_{max} (cm^{-1}) = 3669, 3333, 2984, 1627, 1585, 1514, 1485, 1439, 1379, 1353, 1240, 1143, 1048, 1017, 908, 828, 751, 686; HRMS (ESI) calcd for $C_{20}H_{17}O$ $[M+H]^+$: 273.1274. Found: 273.1275.

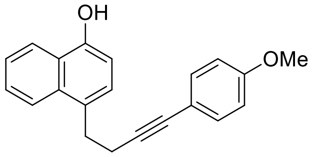

1h

White solid. 60% yield for 2 steps. M.P. = 96-97 °C. ^{1}H NMR (400 MHz, $CDCl_3$) δ 8.23 (d, *J* = 8.0 Hz, 1H), 8.02 (d, *J* = 8.0 Hz, 1H), 7.53 (t, *J* = 6.8 Hz, 1H), 7.48 (t, *J* = 6.8 Hz, 1H), 7.30 (d, *J* = 8.4 Hz, 2H), 7.21 (d, *J* = 8.0 Hz, 1H), 6.80 (d, *J* = 8.4 Hz, 2H), 6.72 (d, *J* = 7.6 Hz, 1H), 5.47 (s, 1H), 3.77 (s, 3H), 3.29 (t, *J* = 7.6 Hz, 2H), 2.77 (t, *J* = 7.6 Hz, 2H). ^{13}C NMR (100 MHz, $CDCl_3$) δ 159.0, 150.3, 132.8, 132.7, 129.2, 126.4, 126.1, 124.8, 124.7, 123.6, 122.3, 116.0, 113.8, 108.1, 88.2, 81.2, 55.2, 32.0, 21.1. IR (thin film): ν_{max} (cm^{-1}) = 3350, 2996, 2928, 2833, 1628, 1602, 1584, 1508,

1440, 1381, 1354, 1289, 1245, 1170, 1148, 1103, 1048, 1030, 828, 756, 702, 661, 629; HRMS (EI) calcd for $C_{21}H_{18}O_2$ $[M]^+$: 302.1307. Found: 302.1315.

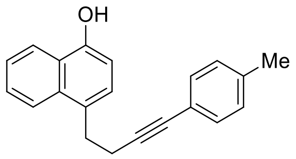

1i

White solid. 69% yield for 2 steps. M.P. = 104-105 °C. ^{1}H NMR (400 MHz, $CDCl_3$) δ 8.25-8.18 (m, 1H), 7.99 (d, *J* = 8.0 Hz, 1H), 7.55-7.41 (m, 2H), 7.26 (d, *J* = 8.0 Hz, 2H), 7.18 (d, *J* = 7.6 Hz, 1H), 7.06 (d, *J* = 8.0 Hz, 2H), 6.68 (d, *J* = 7.6 Hz, 1H), 5.44 (s, 1H), 3.28 (t, *J* = 7.6 Hz, 2H), 2.76 (t, *J* = 7.6 Hz, 2H), 2.30 (s, 3H). ^{13}C NMR (100 MHz, $CDCl_3$) δ 150.2, 137.6, 132.6, 131.3, 129.1, 128.9, 126.4, 126.1, 124.8, 124.6, 123.5, 122.3, 120.6, 108.1, 89.0, 81.6, 31.9, 21.4, 21.1. IR (thin film): ν_{max} (cm^{-1}) = 3687, 3381, 2910, 2321, 1910, 1849, 1835, 1628, 1600, 1584, 1510, 1474, 1452, 1426, 1406, 1379, 1354, 1292, 1261, 1241, 1144, 1105, 1060, 1047, 1017, 946, 830, 816, 755, 703; HRMS (EI) calcd for $C_{21}H_{18}O$ $[M]^+$: 286.1358. Found: 286.1361.

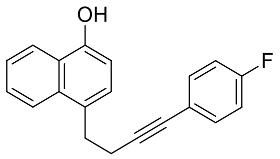

1j

White solid. 62% yield for 2 steps. M.P. = 108-109 °C. ^{1}H NMR (400 MHz, $CDCl_3$) δ 8.28 (d, *J* = 8.0 Hz, 1H), 8.05 (d, *J* = 8.4 Hz, 1H), 7.62-7.49 (m, 2H), 7.36 (t, *J* = 6.0 Hz, 2H), 7.25 (d, *J* = 7.6 Hz, 1H), 7.00 (t, *J* = 7.6 Hz, 2H), 6.77 (d, *J* = 7.6 Hz, 1H), 5.46 (s, 1H), 3.34 (t, *J* = 7.2 Hz, 2H), 2.81 (t, *J* = 7.6 Hz, 2H). ^{13}C NMR (100 MHz, $CDCl_3$) δ 162.0 (d, *J* = 247.0 Hz), 160.8, 150.3, 133.2 (d, *J* = 8.1 Hz), 132.6, 129.0, 126.5, 126.1, 124.9, 124.7, 123.5, 122.3, 119.8 (d, *J* = 3.4 Hz), 115.4 (d, *J* = 21.8 Hz), 108.1, 89.4 (d, *J* = 1.4 Hz), 80.5, 31.8, 21.0. ^{19}F NMR (386 MHz, $CDCl_3$) δ -114.2 (m). IR (thin film): ν_{max} (cm^{-1}) = 3353, 2939, 2321, 1628, 1596, 1584, 1503, 1474, 1380, 1354, 1334, 1291, 1264, 1220, 1147, 1089, 1046, 1015, 972, 832, 759, 703, 659; HRMS (EI) calcd for $C_{20}H_{15}OF$ $[M]^+$: 290.1107. Found: 290.1108.

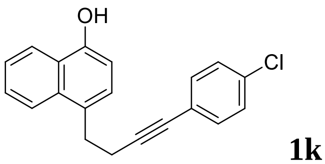

1k

White solid. 70% yield for 2 steps. M.P. = 104-105 °C. ^{1}H NMR (400 MHz, $CDCl_3$) δ 8.28 (d, *J* = 8.0 Hz, 1H), 8.04 (d, *J* = 8.4 Hz, 1H), 7.60-7.50 (m, 2H), 7.31-7.23 (m, 5H), 6.76 (d, *J* = 7.6 Hz, 1H), 5.49 (s, 1H), 3.33 (t, *J* = 7.6 Hz, 2H), 2.81 (t, *J* = 7.6 Hz, 2H). ^{13}C NMR (100 MHz, $CDCl_3$) δ 150.3, 133.5, 132.7, 132.6, 128.9, 128.4, 126.5, 126.1, 124.9, 124.7, 123.5, 122.9, 122.3, 108.1, 90.8, 80.5, 31.7, 21.1. IR (thin film): ν_{max} (cm^{-1}) = 3517, 2926, 2906, 1910, 1623, 1584, 1514, 1488, 1473, 1397, 1379, 1351, 1286, 1264, 1242, 1205, 1141, 1089, 1046, 1012, 974, 826, 772, 702, 636, 619; HRMS (EI) calcd for $C_{20}H_{15}OCl$ $[M]^+$: 306.0811. Found: 306.0816.

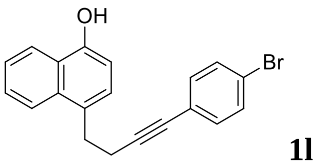

1l

White solid. 41% yield for 2 steps. M.P. = 108-109 °C. ^{1}H NMR (400 MHz, $CDCl_3$) δ 8.28-8.24 (m, 1H), 8.03 (d, *J* = 8.4 Hz, 1H), 7.58-7.50 (m, 2H), 7.41 (d, *J* = 8.4 Hz, 2H), 7.26-7.20 (m, 3H), 6.77 (d, *J* = 7.6 Hz, 1H), 5.37 (s, 1H), 3.32 (t, *J* = 7.6 Hz, 2H), 2.79 (t, *J* = 7.6 Hz, 2H). ^{13}C NMR (100 MHz, $CDCl_3$) δ 150.4, 133.0, 132.6, 131.4, 129.0, 126.5, 126.1, 124.9, 124.7, 123.5, 122.7, 122.4, 121.7, 108.1, 91.0, 80.6, 31.7, 21.2. IR (thin film): ν_{max} (cm^{-1}) = 3518, 2905, 2360, 1973, 1907, 1847, 1734, 1649, 1623, 1583, 1513, 1483, 1394, 1378, 1350, 1286, 1263, 1241, 1204, 1140, 1068, 1045, 1006, 976, 822, 772, 701, 635, 618; HRMS (EI) calcd for $C_{20}H_{15}OBr$ $[M]^+$: 350.0306. Found: 350.0309.

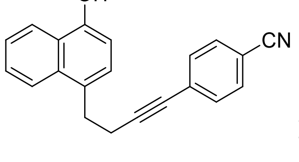

1m

Yellow solid. 78% yield for 2 steps. M.P. = 119-120 °C. ^{1}H NMR (400 MHz, $CDCl_3$) δ 8.26 (d, *J* = 8.4 Hz, 1H), 8.01 (d, *J* = 8.4 Hz, 1H), 7.61-7.48 (m, 4H), 7.39 (d, *J* = 8.4 Hz, 2H), 7.24 (d, *J* = 7.6 Hz, 1H), 6.78 (d, *J* = 7.6 Hz, 1H), 5.50-5.27 (br, 1H),

3.32 (t, *J* = 7.6 Hz, 2H), 2.83 (t, *J* = 7.6 Hz, 2H). ^{13}C NMR (100 MHz, d6-acetone) δ 153.0, 133.6, 132.9, 132.8, 129.5, 128.0 127.7, 127.0, 126.1, 125.1, 124.2, 123.6, 119.0, 111.6, 108.3, 95.7, 81.0, 31.9, 21.7. IR (thin film): ν_{max} (cm^{-1}) = 3417, 2923, 2537, 2321, 2225, 1965, 1925, 1824, 1624, 1600, 1585, 1514, 1499, 1475, 1380, 1338, 1263, 1243, 1214, 1175, 1143, 1104, 1045, 1016, 974, 837, 762, 702; HRMS (EI) calcd for $C_{21}H_{15}NO$ [M]$^+$: 297.1154. Found: 297.1149.

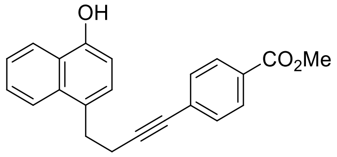

1n

Yellow solid. 67% yield for 2 steps. M.P. = 134-135 ^{o}C. ^{1}H NMR (400 MHz, $CDCl_3$) δ 8.27 (d, *J* = 8.0 Hz, 1H), 8.03 (d, *J* = 8.4 Hz, 1H), 7.96 (d, *J* = 8.0 Hz, 2H), 7.56 (t, *J* = 7.2 Hz, 1H), 7.51 (t, *J* = 6.8 Hz, 1H), 7.40 (d, *J* = 8.0 Hz, 2H), 7.24 (d, *J* = 7.6 Hz, 1H), 6.79 (d, *J* = 8.0 Hz, 1H), 5.69 (s, 1H), 3.92 (s, 3H), 3.33 (t, *J* = 7.6 Hz, 2H), 2.83 (t, *J* = 7.6 Hz, 2H). ^{13}C NMR (100 MHz, $CDCl_3$) δ 150.6, 132.7, 131.4, 129.4, 128.8, 128.75, 128.73, 126.5, 126.2, 124.9, 124.8, 123.5, 122.5, 108.0, 93.3, 81.1, 52.2, 31.7, 21.2. IR (thin film): ν_{max} (cm^{-1}) = 3426, 2901, 2321, 2218, 1939, 1828, 1696, 1626, 1602, 1588, 1557, 1514, 1435, 1404, 1381, 1336, 1310, 1292, 1247, 1193, 1173, 1146, 1118, 1047, 959, 857, 821, 768, 748, 697; HRMS (ESI) calcd for $C_{22}H_{22}NO$ [M+NH$_4$]$^+$: 348.1594. Found: 348.1598.

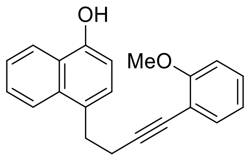

1o

White solid. 43% yield for 2 steps. M.P. = 114-115 ^{o}C. ^{1}H NMR (400 MHz, $CDCl_3$) δ 8.26-8.21 (m, 1H), 8.04 (d, *J* = 8.4 Hz, 1H), 7.58-7.45 (m, 2H), 7.34 (dd, *J* = 7.6, 1.6 Hz, 1H), 7.28-7.26 (m, 1H), 7.25-7.23 (m, 1H), 6.91-6.85 (m, 2H), 6.77 (d, *J* = 7.6 Hz, 1H), 5.25 (s, 1H), 3.88 (s, 3H), 3.35 (t, *J* = 8.0 Hz, 2H), 2.86 (t, *J* = 8.0 Hz, 2H). ^{13}C NMR (100 MHz, $CDCl_3$) δ 159.6, 150.3, 133.7, 132.6, 129.1, 129.0, 126.4, 126.2, 124.8, 124.6, 123.6, 122.3, 120.4, 112.8, 110.4, 108.0, 94.1, 55.7, 32.0, 21.5. IR (thin

film): ν_{max} (cm^{-1}) = 3671, 3441, 2974, 2321, 1838, 1624, 1586, 1516, 1490, 1453, 1431, 1407, 1377, 1350, 1283, 1257, 1182, 1151, 1115, 1047, 1016, 974, 933, 895, 825, 793, 758, 742, 707; HRMS (EI) calcd for $C_{21}H_{18}O_2$ [M]$^+$: 302.1307. Found: 302.1305.

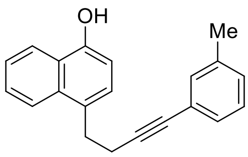

1p

White solid. 63% yield for 2 steps. M.P. = 99-100 ^{o}C. ^{1}H NMR (400 MHz, $CDCl_3$) δ 8.24 (d, *J* = 8.4 Hz, 1H), 8.04 (d, *J* = 8.0 Hz, 1H), 7.59-7.48 (m, 2H), 7.24 (s, 1H), 7.20-7.15 (m, 3H), 7.09 (t, *J* = 4.4 Hz, 1H), 6.77 (d, *J* = 8.0 Hz, 1H), 5.19 (s, 1H), 3.32 (t, *J* = 7.6 Hz, 2H), 2.79 (t, *J* = 7.6 Hz, 2H), 2.31 (s, 3H). ^{13}C NMR (100 MHz, $CDCl_3$) δ 150.3, 137.8, 132.6, 132.1, 129.1, 128.51,128.49, 128.1, 126.5, 126.1, 124.9, 124.7, 123.54, 123.50, 122.3, 108.1, 89.4, 81.6, 31.9, 21.2, 21.1. IR (thin film): ν_{max} (cm^{-1}) = 3275, 3033, 2911, 2321, 2226, 1628, 1599, 1583, 1515, 1480, 1443, 1379, 1354, 1301, 1273, 1248, 1218, 1142, 1091, 1045, 1017, 970, 901, 875, 827, 780, 756, 687; HRMS (EI) calcd for $C_{21}H_{18}O$ [M]$^+$: 286.1358. Found: 286.1353.

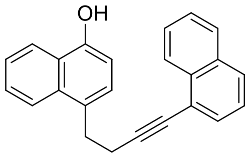

1q

White solid. 70% yield for 2 steps. M.P. = 76-77 ^{o}C. ^{1}H NMR (400 MHz, $CDCl_3$) δ 8.34 (d, *J* = 8.0 Hz, 1H), 8.27 (d, *J* = 7.6 Hz, 1H), 8.12 (d, *J* = 8.4 Hz, 1H), 7.92-7.85 (m, 1H), 7.82 (d, *J* = 8.4 Hz, 1H), 7.67 (d, *J* = 6.8 Hz, 1H), 7.63-7.50 (m, 4H), 7.44 (t, *J* = 7.6 Hz, 1H), 7.34 (d, *J* = 7.6 Hz, 1H), 6.77 (d, *J* = 7.6 Hz, 1H), 5.58 (s, 1H), 3.47 (t, *J* = 7.2 Hz, 2H), 3.03 (t, *J* = 7.2 Hz, 2H). ^{13}C NMR (100 MHz, $CDCl_3$) δ 150.4, 133.4, 133.1, 132.6, 130.0, 129.0, 128.1, 128.0, 126.5, 126.4, 126.33, 126.29, 126.2, 125.2, 124.9, 124.7, 123.5, 122.4, 121.4, 108.1, 94.7, 79.6, 31.9, 21.3. IR (thin film): ν_{max} (cm^{-1}) = 3672, 3285, 2986, 2321, 1925, 1627, 1586, 1514, 1443, 1380, 1355, 1338, 1274, 1250, 1220, 1143, 1047, 971, 902, 825, 795, 759; HRMS (EI) calcd for

$C_{24}H_{18}O$ [M]$^+$: 322.1358. Found: 322.1364.

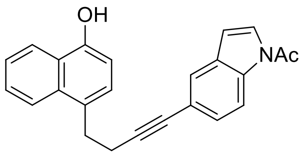

1r

Yellow solid. 56% yield for 2 steps. M.P. = 164-165 ^oC. ^1H NMR (400 MHz, d6-DMSO) δ 10.07 (s, 1H), 8.29 (d, *J* = 8.8 Hz, 1H), 8.24 (d, *J* = 8.4 Hz, 1H), 8.02 (d, *J* = 8.4 Hz, 1H), 7.85 (d, *J* = 3.6 Hz, 1H), 7.59 (s, 1H), 7.57-7.43 (m, 2H), 7.34-7.26 (m, 2H), 6.88 (d, *J* = 8.0 Hz, 1H), 6.69 (d, *J* = 3.2 Hz, 1H), 3.25 (t, *J* = 7.2 Hz, 2H), 2.78 (t, *J* = 7.2 Hz, 2H), 2.63 (s, 3H). ^{13}C NMR (100 MHz, d6-DMSO) δ 169.6, 152.2, 134.1, 132.4, 130.4, 128.2, 127.7, 126.9, 126.6, 126.2, 125.0, 124.2, 123.7, 123.5, 122.7, 118.2, 116.0, 107.9, 107.5, 89.1, 81.9, 31.1, 23.8, 20.7. IR (thin film): ν_{max} (cm^{-1}) = 3183, 3150, 2937, 1671, 1622, 1584, 1542, 1517, 1467, 1440, 1380, 1351, 1330, 1276, 1262, 1217, 1182,1148, 1089, 1047, 1016, 941, 874, 825, 816, 766, 712, 638; HRMS (ESI) calcd for $C_{24}H_{20}NO_2$ [M+H]$^+$: 354.1489. Found: 354.1487.

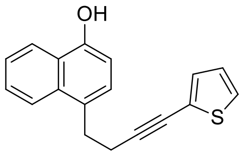

1s

White solid. 66% yield for 2 steps. M.P. = 63-64 ^oC. ^1H NMR (400 MHz, $CDCl_3$) δ 8.27 (d, *J* = 8.0 Hz, 1H), 8.04 (d, *J* = 8.4 Hz, 1H), 7.58 (t, *J* = 7.2 Hz, 1H), 7.52 (t, *J* = 6.8 Hz, 1H), 7.24 (d, *J* = 7.6 Hz, 1H), 7.19 (d, *J* = 4.8 Hz, 1H), 7.13 (d, *J* = 3.2 Hz, 1H), 6.96 (t, *J* = 4.0 Hz, 1H), 6.77 (d, *J* = 7.6 Hz, 1H), 5.46 (s, 1H), 3.33 (t, *J* = 7.6 Hz, 2H), 2.83 (t, *J* = 7.6 Hz, 2H). ^{13}C NMR (100 MHz, $CDCl_3$) δ 150.4, 132.6, 131.0, 128.9, 126.8, 126.5, 126.2, 126.0, 124.9, 124.7, 123.9, 123.5, 122.4, 108.1, 93.8, 74.7, 31.7, 21.4. IR (thin film): ν_{max} (cm^{-1}) = 3273, 3100, 2916, 2843, 2321, 2223, 1840, 1808, 1625, 1583, 1515, 1475, 1438, 1383, 1334, 1269, 1252, 1237, 1213, 1187, 1139, 1062, 1044, 1024, 972, 847, 826, 768,741, 708; HRMS (EI) calcd for $C_{18}H_{14}OS$ [M]$^+$: 278.0765. Found: 278.0766.

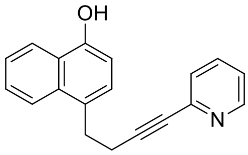

1t

White solid. 65% yield for 2 steps. M.P. = 165-166 °C. ^{1}H NMR (400 MHz, d6-DMSO) δ 10.02 (s, 1H), 8.50 (d, *J* = 4.8 Hz, 1H), 8.18 (d, *J* = 8.0 Hz, 1H), 8.01 (d, *J* = 8.4 Hz, 1H), 7.74 (td, *J* = 7.6, 1.6 Hz, 1H), 7.53 (t, *J* = 7.2 Hz, 1H), 7.45 (t, *J* = 7.2 Hz, 1H), 7.36 (d, *J* = 8.0 Hz, 1H), 7.32 (dd, *J* = 7.6, 4.8 Hz, 1H), 7.27 (d, *J* = 7.6 Hz, 1H), 6.82 (d, *J* = 7.6 Hz, 1H), 3.24 (t, *J* = 7.6 Hz, 2H), 2.80 (t, *J* = 7.2 Hz, 2H). ^{13}C NMR (100 MHz, d6-DMSO) δ 152.2, 149.9, 142.9, 136.6, 132.3, 126.9, 126.3, 124.9, 124.2, 123.4, 122.9, 122.7, 107.5, 90.2, 81.5, 30.7, 20.3. IR (thin film): ν_{max} (cm^{-1}) = 2943, 2577, 2221, 1623, 1583, 1517, 1466, 1432, 1383, 1359, 1273, 1247, 1220, 1152, 1050, 1015, 967, 824, 772, 757, 734, 635; HRMS (EI) calcd for $C_{19}H_{15}NO$ $[M]^{+}$: 273.1154. Found: 273.1152.

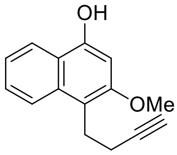

1u

Colorless oil. ^{1}H NMR (400 MHz, $CDCl_3$) δ 8.11 (d, *J* = 8.4 Hz, 1H), 7.94 (d, *J* = 8.4 Hz, 1H), 7.51 (t, *J* = 8.0 Hz, 1H), 7.34 (t, *J* = 7.6 Hz, 1H), 6.68 (s, 1H), 5.51 (s, 1H), 3.87 (s, 3H), 3.33-3.20 (m, 2H), 2.49-2.41 (m, 2H), 2.00 (s, 1H). ^{13}C NMR (100 MHz, $CDCl_3$) δ 154.5, 151.4, 133.4, 127.1, 122.9, 122.5, 121.9, 119.9, 114.1, 97.5, 84.8, 68.3, 56.3, 24.0, 19.0. IR (thin film): ν_{max} (cm^{-1}) = 3290, 3069, 2939, 2845, 2115, 1626, 1590, 1518, 1456, 1379, 1362, 1292, 1232, 1173, 1150, 1111, 1071, 1025, 997, 824, 760, 632; HRMS (EI) calcd for $C_{15}H_{14}O_2$ $[M]^{+}$: 226.0994. Found: 226.0990.

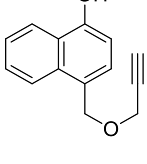

1v

White solid. M.P. = 82-83 °C. ^{1}H NMR (400 MHz, $CDCl_3$) δ 8.22 (d, *J* = 7.6 Hz, 1H), 8.14 (d, *J* = 8.0 Hz, 1H), 7.59-7.48 (m, 2H), 7.30 (d, *J* = 7.6 Hz, 1H), 6.66 (d, *J* = 7.6

Hz, 1H), 5.93 (s, 1H), 5.00 (s, 2H), 4.21 (d, J = 2.4 Hz, 2H), 2.54 (t, J = 2.4 Hz, 1H). ^{13}C NMR (100 MHz, $CDCl_3$) δ 152.3, 133.1, 128.3, 126.9, 125.1, 124.8, 124.4, 124.0, 122.2, 107.6, 79.5, 75.0, 69.8, 56.6. IR (thin film): ν_{max} (cm^{-1}) = 3285, 3209, 2953, 2931, 2866, 1582, 1516, 1384, 1352, 1278, 1224, 1152, 1043, 953, 879, 826, 767, 698, 671, 630; Anal. calcd for $C_{14}H_{12}O_2$: C, 79.22; H, 5.70; Found: C, 79.03; H, 5.75.

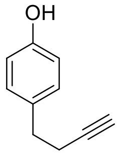

1w

White solid. M.P. = 46-47 °C. ^{1}H NMR (400 MHz, $CDCl_3$) δ 7.10 (d, J = 8.4 Hz, 2H), 6.79 (d, J = 8.4 Hz, 2H), 5.37 (s, 1H), 2.79 (t, J = 7.6 Hz, 2H), 2.45 (dt, J = 7.2, 2.8 Hz, 2H), 2.01 (t, J = 2.8 Hz, 1H). ^{13}C NMR (100 MHz, $CDCl_3$) δ 153.8, 132.7, 129.6, 115.2, 84.0, 68.9, 33.9, 20.8. IR (thin film): ν_{max} (cm^{-1}) = 3280, 3201, 2930, 2115, 1888, 1660, 1610, 1511, 1444, 1363, 1288, 1232, 1170, 1100, 1015, 849, 832, 776, 646; HRMS (EI) calcd for $C_{10}H_{10}O$ $[M]^+$: 146.0732. Found: 146.0735.

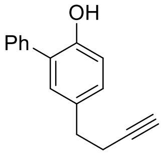

1x

Colorless oil. ^{1}H NMR (400 MHz, $CDCl_3$) δ 7.60-7.51 (m, 4H), 7.48-7.42 (m, 1H), 7.23-7.16 (m, 2H), 6.98 (d, J = 8.4 Hz, 1H), 5.61 (s, 1H), 2.91 (t, J = 7.6 Hz, 2H), 2.57 (td, J = 7.6, 2.8 Hz, 2H), 2.09 (t, J = 2.8 Hz, 1H). ^{13}C NMR (100 MHz, $CDCl_3$) δ 150.7, 137.1, 132.6, 130.1, 129.0, 128.8, 128.7, 127.8, 127.5, 115.7, 83.9, 69.0, 33.7, 20.6. IR (thin film): ν_{max} (cm^{-1}) = 3519, 3288, 2926, 2321, 2114, 1600, 1504, 1486, 1446, 1416, 1330, 1289, 1269, 1229, 1174, 1125, 1074, 1024, 890, 815, 755, 731, 700, 628; HRMS (EI) calcd for $C_{16}H_{14}O$ $[M]^+$: 222.1045. Found: 222.1047.

General procedure for gold-catalyzed dearomatization of naphthols

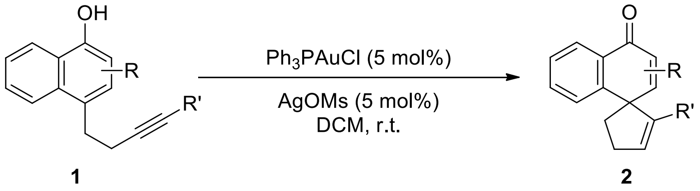

A flame-dried Schlenk tube was cooled down to room temperature under argon. To this tube were added Ph_3PAuCl (4.9 mg, 0.01 mmol, 0.5 mol%), 1-naphthol derivative **1** (0.2 mmol, 1.0 equiv.), and DCM (2.0 mL), and AgOMs (2.0 mg, 0.01 mmol, 0.5 mol%) was added last. Then the reaction mixture was stirred at room temperature in the dark. After completion (monitored by TLC), the reaction mixture was loaded on the silica column. The crude product was purified by silica gel column chromatography (PE/EtOAc = 10/1) to afford the desired product **2**.

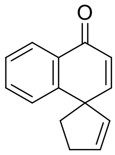

2a

Colorless oil. 96% yield (39.1 mg). ^{1}H NMR (400 MHz, $CDCl_3$) δ 8.18-8.13 (m, 1H), 7.56-7.52 (m, 1H), 7.41-7.34 (m, 2H), 6.89 (d, *J* = 10.0 Hz, 1H), 6.37 (d, *J* = 10.0 Hz, 1H), 6.17-6.09 (m, 1H), 5.45-5.39 (m, 1H), 2.81-2.66 (m, 2H), 2.37-2.25 (m, 2H). ^{13}C NMR (100 MHz, $CDCl_3$) δ 185.0, 153.9, 148.7, 134.9, 134.0, 132.7, 130.9, 127.6, 126.9, 126.2, 54.6, 39.4, 32.8. IR (thin film): ν_{max} (cm^{-1}) = 3669, 2968, 2904, 1658, 1599, 1477, 1454, 1390, 1299, 1130, 1061, 968, 918, 841, 766, 742, 708, 681; HRMS (ESI) calcd for $C_{14}H_{13}O$ $[M+H]^+$: 197.0961. Found: 197.0962.

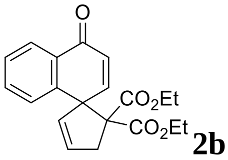

2b

Pink solid, M.P. = 58-59 ^oC. 99% yield (67.6 mg). ^{1}H NMR (400 MHz, $CDCl_3$) δ 8.13 (dd, *J* = 7.6, 1.2 Hz, 1H), 7.46 (td, *J* = 7.6, 1.2 Hz, 1H), 7.40-7.33 (m, 2H), 7.25 (d, *J* = 7.6 Hz, 1H), 6.52 (d, *J* = 10.4 Hz, 1H), 6.27-6.18 (m, 1H), 5.60-5.52 (m, 1H), 4.24-4.06 (m, 2H), 3.73-3.63 (m, 1H), 3.55 (dt, *J* = 18.0, 2.0 Hz, 1H), 3.37-3.27 (m,

1H), 3.01-2.92 (m, 1H), 1.20 (t, *J* = 7.2 Hz, 3H), 0.77 (t, *J* = 7.2 Hz, 3H). ^{13}C NMR (100 MHz, $CDCl_3$) δ 184.5, 170.1, 168.6, 149.1, 142.3, 133.5, 132.4, 132.1, 131.9, 129.8, 127.9, 127.4, 126.4, 70.0, 61.8, 61.3, 58.8, 41.7, 13.8, 13.1. IR (thin film): ν_{max} (cm^{-1}) = 3077, 2989, 2964, 2932, 2901, 2869, 1748, 1727, 1667, 1594, 1453, 1392, 1302, 1235, 1148, 1073, 1050, 1014, 966, 925, 886, 847, 767, 744, 723, 706, 667, 622; HRMS (ESI) calcd for $C_{22}H_{24}NO_5$ $[M+NH_4]^+$: 358.1649. Found: 358.1648.

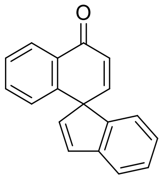
2c

Yellow solid, M.P. = 98-99 °C. 95% yield (54.4 mg). ^{1}H NMR (400 MHz, $CDCl_3$) δ 8.24 (d, *J* = 7.6 Hz, 1H), 7.45 (d, *J* = 7.6 Hz, 1H), 7.41-7.28 (m, 3H), 7.16 (t, *J* = 7.2 Hz, 1H), 7.10 (d, *J* = 5.2 Hz, 1H), 6.95 (d, *J* = 7.6 Hz, 1H), 6.71 (d, *J* = 7.6 Hz, 1H), 6.64 (d, *J* = 10.0 Hz, 1H), 6.41 (d, *J* = 10.0 Hz, 1H), 6.28 (d, *J* = 5.2 Hz, 1H). ^{13}C NMR (100 MHz, $CDCl_3$) δ 185.3, 148.8, 147.4, 144.2, 140.9, 139.7, 134.0, 132.6, 132.0, 129.4, 128.1, 127.6, 126.9, 126.81, 126.77, 123.6, 121.9, 59.1. IR (thin film): ν_{max} (cm^{-1}) = 3059, 2923, 2321, 1973, 1659, 1597, 1478, 1452, 1384, 1300, 1268, 1242, 1168, 1150, 1123, 1080, 1002, 945, 879, 845, 771, 758, 735, 676; HRMS (ESI) calcd for $C_{18}H_{13}O$ $[M+H]^+$: 245.0961. Found: 245.0962.

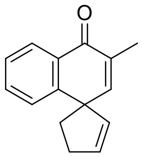
2d

Yellowish oil, 44% yield (20.0 mg). ^{1}H NMR (400 MHz, $CDCl_3$)δ 8.21-8.12 (m, 1H), 7.51 (td, *J* = 8.0, 1.6 Hz, 1H), 7.37 (t, *J* = 7.6 Hz, 2H), 6.69 (d, *J* = 1.2 Hz, 1H), 6.13-6.05 (m, 1H), 5.41 (dt, *J* = 4.8, 2.0 Hz, 1H), 2.78-2.64 (m, 2H), 2.35-2.21 (m, 2H), 2.01 (d, *J* = 1.2 Hz, 3H). ^{13}C NMR (100 MHz, $CDCl_3$) δ 185.5, 149.5, 148.9, 135.4, 133.4, 132.3, 132.2, 130.7, 127.4, 126.8, 126.4, 54.2, 39.4, 32.8, 16.3. IR (thin film): ν_{max} (cm^{-1}) = 3301, 2946, 2852, 1653, 1598, 1522, 1479, 1455, 1370, 1315, 1274, 1176, 1108, 1042, 1018, 984, 899, 800, 780, 753, 704, 627; HRMS (ESI) calcd

for $C_{15}H_{15}O$ $[M+H]^+$: 211.1117. Found: 211.1117.

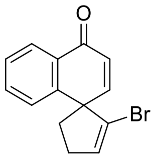

2e

Yellow solid, M.P. = 63-64 ^{o}C. 99% yield (54.5 mg). ^{1}H NMR (400 MHz, $CDCl_3$) δ 8.16 (dd, *J* = 8.0, 1.2 Hz, 1H), 7.58 (td, *J* = 7.6, 1.2 Hz, 1H), 7.45-7.35 (m, 2H), 6.85 (d, *J* = 10.0 Hz, 1H), 6.49 (d, *J* = 10.0 Hz, 1H), 6.28 (t, *J* = 2.4 Hz, 1H), 2.74-2.60 (m, 2H), 2.53-2.40 (m, 2H). ^{13}C NMR (100 MHz, $CDCl_3$) δ 184.5, 151.5, 145.9, 134.8, 133.0, 131.5, 128.5, 127.5, 126.8, 126.4, 125.5, 57.7, 39.1, 31.5. IR (thin film): ν_{max} (cm^{-1}) = 3068, 2926, 2855, 1656, 1622, 1598, 1453, 1385, 1298, 1152, 1130, 1056, 1018, 991, 926, 853, 835, 758, 677; HRMS (ESI) calcd for $C_{14}H_{12}BrO$ $[M+H]^+$: 275.0066. Found: 275.0065.

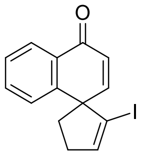

2f

Yellow solid, M.P. = 78-79 ^{o}C. 99% yield (64.2 mg). ^{1}H NMR (400 MHz, $CDCl_3$) δ 8.15 (dd, *J* = 7.6, 1.2 Hz, 1H), 7.59-7.54 (m, 1H), 7.44-7.39 (m, 1H), 7.32 (d, *J* = 8.0 Hz, 1H), 6.76 (d, *J* = 10.0 Hz, 1H), 6.52-6.48 (m, 2H), 2.78-2.60 (m, 2H), 2.44 (t, *J* = 7.2 Hz, 2H). ^{13}C NMR (100 MHz, $CDCl_3$) δ 184.6, 152.0, 146.6, 143.2, 133.0, 131.5, 128.4, 127.5, 127.0, 126.4, 101.4, 59.9, 38.1, 34.4. IR (thin film): ν_{max} (cm^{-1}) = 3025, 2924, 2854, 1730, 1659, 1596, 1476, 1452, 1388, 1301, 1167, 1126, 1064, 1033, 1013, 983, 958, 924, 843, 767, 681; HRMS (ESI) calcd for $C_{14}H_{12}IO$ $[M+H]^+$: 322.9927. Found: 322.9929.

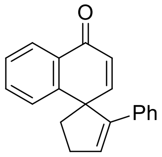

2g

White solid, M.P. = 162-163 ^{o}C. 99% yield (45.8 mg). ^{1}H NMR (400 MHz, $CDCl_3$) δ 8.23 (dd, *J* = 8.4, 1.6 Hz, 1H), 7.50-7.44 (m, 1H), 7.41-7.34 (m, 2H), 7.13 (d, *J* = 10.0

Hz, 1H), 7.11-7.05 (m, 3H), 7.07-7.00 (m, 2H), 6.58 (t, *J* = 2.8 Hz, 1H), 6.49 (d, *J* = 10.0 Hz, 1H), 2.86-2.70 (m, 2H), 2.54-2.45 (m, 1H), 2.37-2.27 (m, 1H). ^{13}C NMR (100 MHz, $CDCl_3$) δ 184.9, 155.5, 148.0, 145.6, 134.8, 132.9, 131.6, 130.9, 128.1, 127.2, 127.1, 126.9, 126.70, 126.66, 125.9, 56.0, 42.6, 31.1. IR (thin film): ν_{max} (cm^{-1}) = 2966, 2933, 2904, 1654, 1597, 1491, 1449, 1388, 1302, 1256, 1152, 1123, 1061, 845, 764, 694; HRMS (ESI) calcd for $C_{20}H_{17}O$ [M+H]$^+$: 273.1274. Found: 273.1273.

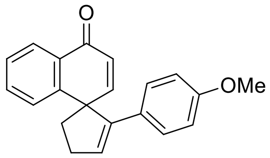

2h

Yellow solid, M.P. = 98-99 ^{o}C. 99% yield (60.2 mg). ^{1}H NMR (400 MHz, $CDCl_3$) δ 8.26-8.22 (m, 1H), 7.48-7.42 (m, 1H), 7.41-7.33 (m, 2H), 7.12 (d, *J* = 10.0 Hz, 1H), 7.00-6.94 (m, 2H), 6.64-6.58 (m, 2H), 6.49 (d, *J* = 10.0 Hz, 1H), 6.45 (t, *J* = 2.4 Hz, 1H), 3.65 (s, 3H), 2.81-2.67 (m, 2H), 2.52-2.41 (m, 1H), 2.33-2.24 (ddd, *J* = m, 1H). ^{13}C NMR (100 MHz, $CDCl_3$) δ 185.0, 158.7, 155.9, 148.2, 145.1, 133.0, 131.0, 129.6, 127.4, 127.14, 127.06, 127.0, 126.7 113.5, 56.1, 55.0, 42.6, 31.1. IR (thin film): ν_{max} (cm^{-1}) = 3066, 3037, 3004, 2968, 2931, 2839, 2322, 2297, 1888, 1732, 1651, 1601, 1570, 1507, 1476, 1453, 1387, 1296, 1246, 1179, 1153, 1123, 1111, 1029, 999, 967, 933, 891, 834, 808, 771, 682; HRMS (ESI) calcd for $C_{21}H_{19}O_2$ [M+H]$^+$: 303.1380. Found: 303.1389.

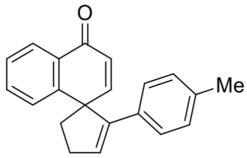

2i

Yellow solid, M.P. = 139-140 ^{o}C. 97% yield (55.6 mg). ^{1}H NMR (400 MHz, $CDCl_3$) δ 8.25-8.21 (m, 1H), 7.50-7.44 (m, 1H), 7.40-7.35 (m, 2H), 7.13 (d, *J* = 10.0 Hz, 1H), 6.95-6.88 (m, 4H), 6.53 (t, *J* = 2.4 Hz, 1H), 6.49 (d, *J* = 10.0 Hz, 1H), 2.83-2.69 (m, 2H), 2.52-2.42 (m, 1H), 2.33-2.24 (m, 1H), 2.21 (s, 3H). ^{13}C NMR (100 MHz, $CDCl_3$) δ 185.0, 155.7, 148.2, 145.6, 137.2, 133.0, 132.0, 131.0, 130.6, 128.8, 127.1, 126.9, 125.8, 56.1, 42.7, 31.1, 21.0. IR (thin film): ν_{max} (cm^{-1}) = 3670, 2970, 2917, 2853,

2321, 1658, 1597, 1567, 1510, 1473, 1452, 1385, 1297, 1251, 1188, 1150, 1124, 1027, 967, 893, 804, 772, 687, 647; HRMS (ESI) calcd for $C_{21}H_{19}O$ [M+H]$^+$: 287.1430. Found: 287.1435.

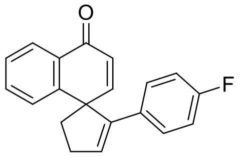

2j

Yellow solid, M.P. = 167-168 ^{o}C. 99% yield (58.6 mg). ^{1}H NMR (400 MHz, $CDCl_3$) δ 8.25-8.20 (m, 1H), 7.47 (td, *J* = 7.6, 1.6 Hz, 1H), 7.42-7.33 (m, 2H), 7.10 (d, *J* = 10.0 Hz, 1H), 7.02-6.94 (m, 2H), 6.81-6.71 (m, 2H), 6.54-6.43 (m, 2H), 2.84-2.67 (m, 2H), 2.52-2.42 (m, 1H), 2.37-2.26 (m, 1H). ^{13}C NMR (100 MHz, $CDCl_3$) δ 184.8, 163.2 (d, *J* = 246.0 Hz), 160.7, 155.2, 147.8, 144.7, 133.0, 131.5, 131.4, 131.03, 131.00, 127.6 (d, *J* = 7.8 Hz), 127.3, 127.1, 126.8, 126.7, 115.0 (d, *J* = 21.4 Hz), 56.1, 42.7, 31.1; ^{19}F NMR (386 MHz, $CDCl_3$) δ -114.3 (m). IR (thin film): ν_{max} (cm^{-1}) = 3055, 2942, 2854, 2321, 1914, 1650, 1598, 1503, 1476, 1453, 1388, 1299, 1261, 1245, 1218, 1153, 1124, 1100, 1064, 1032, 1017, 1001, 967, 928, 897, 843, 805, 766, 683; HRMS (ESI) calcd for $C_{20}H_{16}FO$ [M+H]$^+$: 291.1180. Found: 291.1182.

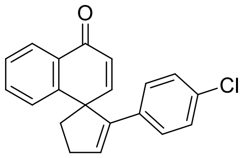

2k

Yellow solid, M.P. = 112-113 ^{o}C. 99% yield (61.1 mg). ^{1}H NMR (400 MHz, $CDCl_3$).δ 8.25-8.19 (m, 1H), 7.50-7.44 (m, 1H), 7.37 (t, *J* = 7.6 Hz, 1H), 7.34 (d, *J* = 8.4 Hz, 1H), 7.10 (d, *J* = 10.0 Hz, 1H), 7.04 (d, *J* = 8.8 Hz, 2H), 6.94 (d, *J* = 8.8 Hz, 2H), 6.55 (t, *J* = 2.4 Hz, 1H), 6.49 (d, *J* = 10.4 Hz, 1H), 2.83-2.69 (m, 2H), 2.52-2.42 (m, 1H), 2.362.27 (m, 1H). ^{13}C NMR (100 MHz, $CDCl_3$) δ 184.8, 155.0, 147.7, 144.7, 133.3, 133.1, 132.3, 131.0, 128.3, 127.4, 127.23, 127.16, 126.9, 126.7, 56.0, 42.7, 31.2. IR (thin film): ν_{max} (cm^{-1}) = 3033, 2925, 2348, 1728, 1659, 1598, 1489, 1452, 1385, 1298, 1269, 1152, 1125, 1092, 1064, 1008, 964, 892, 827, 808, 771, 720, 686; HRMS (ESI) calcd for $C_{20}H_{16}ClO$ [M+H]$^+$: 307.0884. Found: 307.0888.

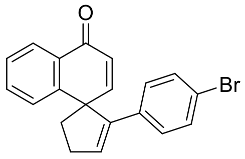

2l

Yellow solid, M.P. = 143-144 ^{o}C. 99% yield (78.6 mg). ^{1}H NMR (400 MHz, $CDCl_3$) δ 8.22 (d, *J* = 7.6 Hz, 1H), 7.49-7.43 (m, 1H), 7.41-7.30 (m, 2H), 7.19 (d, *J* = 8.4 Hz, 2H), 7.09 (d, *J* = 10.0 Hz, 1H), 6.87 (d, *J* = 8.8 Hz, 2H), 6.56 (t, *J* = 2.4 Hz, 1H), 6.49 (d, *J* = 10.0 Hz, 1H), 2.83-2.67 (m, 2H), 2.52-2.42 (m, 1H), 2.36-2.27 (m, 1H). ^{13}C NMR (100 MHz, $CDCl_3$) δ 184.7, 155.0, 147.6, 144.7, 133.7, 133.1, 132.4, 131.2, 131.0, 127.5, 127.3, 127.1, 126.8, 126.7, 121.2, 55.9, 42.7, 31.2. IR (thin film): ν_{max} (cm^{-1}) = 3093, 3060, 3033, 2941, 2851, 2321, 1733, 1659, 1598, 1568, 1484, 1452, 1385, 1297, 1269, 1167, 1151, 1126, 1109, 1072, 1026, 1005, 961, 891, 840, 825, 807, 773, 753, 714, 685; HRMS (ESI) calcd for $C_{20}H_{16}OBr$ $[M+H]^+$: 351.0379. Found: 351.0390.

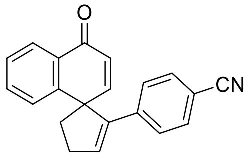

2m

Yellow solid, M.P. = 154-155 ^{o}C. 97% yield (59.3 mg). ^{1}H NMR (400 MHz, $CDCl_3$) δ 8.20 (d, *J* = 7.6 Hz, 1H), 7.45 (t, *J* = 7.6 Hz, 1H), 7.40-7.27 (m, 4H), 7.12-7.04 (m, 3H), 6.72 (t, *J* = 2.4 Hz, 1H), 6.49 (d, *J* = 10.4 Hz, 1H), 2.87-2.74 (m, 2H), 2.54-2.44 (m, 1H), 2.39-2.29 (m, 1H). ^{13}C NMR (100 MHz, $CDCl_3$) δ 184.4, 154.2, 147.2, 144.4, 139.3, 135.4, 133.2, 131.9, 130.9, 127.6, 127.3, 126.9, 126.5, 126.4, 118.5, 110.6, 55.8, 42.7, 31.3. IR (thin film): ν_{max} (cm^{-1}) = 3070, 3037, 2925, 2321, 2219, 1726, 1662, 1625, 1600, 1501, 1453, 1389, 1300, 1262, 1151, 1128, 1101, 1063, 1028, 995, 960, 888, 833, 805, 766, 690; HRMS (ESI) calcd for $C_{21}H_{16}NO$ $[M+H]^+$: 298.1226. Found: 298.1228.

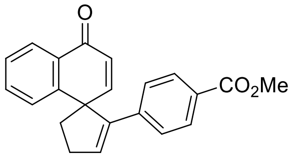

2n

Yellow solid, M.P. = 131-133 °C. 96% yield (64.3 mg). ^{1}H NMR (400 MHz, $CDCl_3$) δ 8.22 (dd, *J* = 7.8, 1.2 Hz, 1H), 7.74 (d, *J* = 8.4 Hz, 2H), 7.45 (td, *J* = 7.6, 1.2 Hz, 1H), 7.36 (t, *J* = 7.6 Hz, 1H), 7.32 (d, *J* = 8.0 Hz, 1H), 7.11 (d, *J* = 10.0 Hz, 1H), 7.07 (d, *J* = 8.4 Hz, 2H), 6.69 (t, *J* = 2.4 Hz, 1H), 3.81 (s, 3H), 2.84-2.74 (m, 2H), 2.52-2.43 (m, 1H), 2.38-2.28 (m, 1H). ^{13}C NMR (100 MHz, $CDCl_3$) δ 184.7, 166.5, 154.8, 147.6, 145.1, 139.3, 134.1, 133.0, 131.0, 129.4, 128.7, 127.4, 127.1, 126.8, 126.6, 125.8, 55.9, 51.9, 42.8, 31.3. IR (thin film): ν_{max} (cm^{-1}) = 3052, 2922, 2848, 2348, 2321, 1706, 1659, 1605, 1564, 1475, 1451, 1431, 1409, 1388, 1281, 1185, 1165, 1152, 1106, 1018, 999, 968, 890, 850, 823, 783, 766, 703; HRMS (ESI) calcd for $C_{22}H_{19}O_3$ $[M+H]^+$: 331.1329. Found: 331.1332.

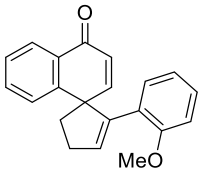

2o

Yellow solid, M.P. = 110-111 °C. 99% yield (60.3 mg). ^{1}H NMR (400 MHz, $CDCl_3$) δ 8.17 (d, *J* = 7.6 Hz, 1H), 7.49-7.39 (m, 2H), 7.36-7.30 (m, 1H), 7.19 (d, *J* = 10.0 Hz, 1H), 7.09-7.01 (m, 1H), 6.76 (d, *J* = 8.4 Hz, 1H), 6.70 (t, *J* = 2.4 Hz, 1H), 6.67-6.63 (m, 1H), 6.57 (t, *J* = 7.6 Hz, 1H), 6.43 (d, *J* = 10.0 Hz, 1H), 3.69 (s, 3H), 2.79 (td, *J* = 7.2, 2.4 Hz, 2H), 2.50-2.38 (m, 1H), 2.34-2.22 (m, 1H). ^{13}C NMR (100 MHz, $CDCl_3$) δ 185.1, 157.1, 156.3, 148.3, 142.3, 135.3, 132.6, 130.9, 128.7, 128.2, 126.8, 126.7, 126.5, 126.3, 124.0, 119.9, 110.6, 57.6, 54.8, 42.4, 31.8. IR (thin film): ν_{max} (cm^{-1}) = 3053, 2928, 2849, 2322, 1652, 1596, 1488, 1454, 1390, 1300, 1253, 1179, 1152, 1118, 1052, 1026, 1000, 930, 895, 842, 758, 695; HRMS (ESI) calcd for $C_{21}H_{19}O_2$ $[M+H]^+$: 303.1380. Found: 303.1379.

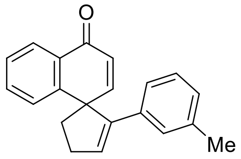

2p

Yellow solid, M.P. = 116-117 °C. 96% yield (54.9 mg). ^{1}H NMR (400 MHz, $CDCl_3$) δ 8.25 (d, *J* = 7.6 Hz, 1H), 7.53-7.45 (m, 1H), 7.44-7.33 (m, 2H), 7.13 (d, *J* = 10.0 Hz, 1H), 7.02-6.85 (m, 3H), 6.74 (d, *J* = 7.2 Hz, 1H), 6.57 (t, *J* = 2.4 Hz, 1H), 6.50 (d, *J* = 10.0 Hz, 1H), 2.85-2.68 (m, 2H), 2.54-2.42 (m, 1H), 2.36-2.25 (m, 1H), 2.18 (s, 3H). ^{13}C NMR (100 MHz, $CDCl_3$) δ 185.0, 155.7, 148.1, 145.8, 137.6, 134.8, 133.0, 131.4, 130.9, 128.2, 128.0, 127.1, 127.0, 126.8, 126.74, 126.72, 122.9, 56.1, 42.6, 31.1, 21.3. IR (thin film): ν_{max} (cm^{-1}) = 3051, 2938, 2322, 1951, 1653, 1599, 1481, 1451, 1385, 1298, 1248, 1152, 1124, 1095, 1031, 968, 928, 883, 841, 800, 780, 766, 699; HRMS (ESI) calcd for $C_{21}H_{19}O$ [M+H]$^+$: 287.1430. Found: 287.1436.

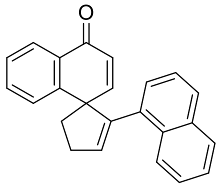

2q

Yellow solid, M.P. = 167-168 °C. 91% yield (59.0 mg). ^{1}H NMR (400 MHz, $CDCl_3$) δ 8.29 (d, *J* = 8.4 Hz, 1H), 8.13 (d, *J* = 8.0 Hz, 1H), 7.76 (d, *J* = 8.0 Hz, 1H), 7.63 (d, *J* = 7.6 Hz, 1H), 7.59 (d, *J* = 8.4 Hz, 1H), 7.53-7.42 (m, 3H), 7.34 (t, *J* = 7.6 Hz, 1H), 7.17 (d, *J* = 10.0 Hz, 1H), 7.05 (t, *J* = 7.6 Hz, 1H), 6.63 (d, *J* = 7.2 Hz, 1H), 6.37-6.32 (m, 2H), 3.09-2.93 (m, 2H), 2.69-2.53 (m, 2H). ^{13}C NMR (100 MHz, $CDCl_3$) δ 184.8, 154.2, 147.8, 143.8, 135.7, 133.7, 133.3, 132.7, 132.0, 131.3, 128.4, 127.6, 127.3, 127.1, 127.0, 126.4, 125.8, 125.4, 125.2, 124.6, 123.7, 58.6, 41.4, 32.3. IR (thin film): ν_{max} (cm^{-1}) = 3687, 3673, 2972, 2348, 2321, 1656, 1596, 1504, 1453, 1407, 1391, 1300, 1243, 1151, 1127, 1064, 988, 925, 894, 845, 775, 683, 660; HRMS (ESI) calcd for $C_{24}H_{19}O$ [M+H]$^+$: 323.1430. Found: 323.1432.

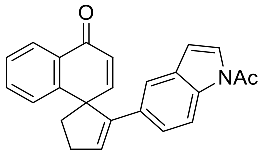

2r

White solid, M.P. = 168-169 °C. 98% yield (69.5 mg). ^{1}H NMR (400 MHz, $CDCl_3$) δ 8.24 (d, *J* = 7.6 Hz, 1H), 8.16 (d, *J* = 8.0 Hz, 1H), 7.48-7.43 (m, 1H), 7.42-7.34 (m, 2H), 7.30 (d, *J* = 3.2 Hz, 1H), 7.20-7.15 (m, 2H), 7.08 (s, 1H), 6.58 (t, *J* = 2.4 Hz, 1H), 6.51 (d, *J* = 10.0 Hz, 1H), 6.41 (d, *J* = 3.6 Hz, 1H), 2.83-2.74 (m, 2H), 2.55 (s, 3H), 2.53-2.46 (m, 1H), 2.38-2.29 (m, 1H). ^{13}C NMR (100 MHz, $CDCl_3$) δ 185.0, 168.3, 155.7, 148.1, 145.9, 134.6, 133.0, 131.2, 131.0, 130.6, 130.1, 127.1, 127.0, 126.78, 126.76, 125.4, 123.6, 118.0, 116.1, 109.1, 56.3, 42.8, 31.1, 23.7. IR (thin film): ν_{max} (cm^{-1}) = 3672, 2970, 2348, 2322, 1708, 1652, 1600, 1536, 1461, 1435, 1375, 1323, 1303, 1272, 1222, 1188, 1155, 1039, 935, 887, 829, 765, 728, 705, 630; HRMS (ESI) calcd for $C_{24}H_{20}NO_2$ $[M+H]^+$: 354.1489. Found: 354.1491.

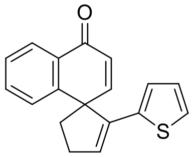

2s

White solid, M.P. = 120-121 °C. 95% yield (52.9 mg). ^{1}H NMR (400 MHz, $CDCl_3$) δ 8.23 (dd, *J* = 7.6, 1.2 Hz, 1H), 7.53-7.47 (m, 1H), 7.45-7.35 (m, 2H), 7.07 (d, *J* = 10.0 Hz, 1H), 6.97 (d, *J* = 4.4 Hz, 1H), 6.66 (dd, *J* = 4.8, 3.6 Hz, 1H), 6.51-6.44 (m, 2H), 6.34 (d, *J* = 3.6 Hz, 1H), 2.87-2.70 (m, 2H), 2.54-2.33 (m, 1H), 2.40-2.31 (m, 1H). ^{13}C NMR (100 MHz, $CDCl_3$) δ 184.8, 154.4, 147.7, 139.8, 137.9, 133.0, 131.2, 130.6, 127.4, 127.2, 127.10, 127.06, 126.6, 124.2, 124.1, 56.4, 41.9, 31.4. IR (thin film): ν_{max} (cm^{-1}) = 2935, 2847, 2321, 1657, 1598, 1570, 1515, 1452, 1423, 1390, 1302, 1245, 1168, 1125, 1079, 1029, 988, 966, 933, 847, 815, 770, 695; HRMS (ESI) calcd for $C_{24}H_{20}NO_2$ $[M+H]^+$: 279.0838. Found: 279.0840.

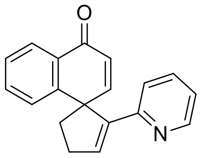

2t

White solid, M.P. = 160-161 °C. 99% yield (54.0 mg). ^{1}H NMR (400 MHz, $CDCl_3$) δ 8.40 (d, *J* = 4.4 Hz, 1H), 8.24-8.17 (m, 1H), 7.45-7.40 (m, 1H), 7.38-7.32 (m, 2H), 7.29 (dd, *J* = 8.0, 2.0 Hz, 1H), 7.16 (d, *J* = 2.8 Hz, 1H), 7.13 (d, *J* = 10.0 Hz, 1H),

6.95 (dd, J = 7.6, 4.8 Hz, 1H), 6.62 (d, J = 8.0 Hz, 1H), 6.49 (d, J = 10.0 Hz, 1H), 2.86-2.74 (m, 2H), 2.55-2.45 (m, 1H), 2.4-2.30 (m, 1H). ^{13}C NMR (100 MHz, $CDCl_3$) δ 185.0, 155.1, 152.6, 149.3, 148.1, 145.7, 136.2, 136.1 133.0, 131.1, 127.1, 127.0, 126.7, 126.5, 121.9, 120.0, 55.1, 42.6, 31.1. IR (thin film): ν_{max} (cm^{-1}) = 3051, 2961, 2933, 2844, 1653, 1586, 1437, 1388, 1303, 1155, 980, 842, 783, 763, 685; HRMS (ESI) calcd for $C_{19}H_{16}NO$ [M+H]$^+$: 274.1226. Found: 274,1229.

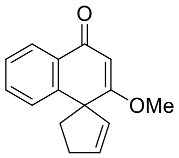

2u

White solid, M.P. = 88-89 ^{o}C. 86% yield (35.9 mg). ^{1}H NMR (400 MHz, $CDCl_3$) δ 8.14 (d, J = 6.8 Hz, 1H), 7.54-7.48 (m, 1H), 7.36 (t, J = 7.6 Hz, 1H), 7.31 (d, J = 8.0 Hz, 1H), 6.20-6.14 (m, 1H), 5.80 (s, 1H), 5.45-5.38 (m, 1H), 3.79 (s, 3H), 2.78-2.67 (m, 2H), 2.69-2.48 (m, 1H), 2.22-2.11 (m, 1H). ^{13}C NMR (100 MHz, $CDCl_3$) δ 185.7, 178.6, 146.8, 134.6, 134.1, 132.4, 129.7, 127.0, 126.7, 125.6, 101.3, 57.6, 56.0, 39.6, 33.4. IR (thin film): ν_{max} (cm^{-1}) = 3057, 3016, 2925, 2851, 1639, 1600, 1454, 1435, 1362, 1322, 1223, 1160, 1132, 1067, 1028, 834, 778, 748, 691, 664; HRMS (ESI) calcd for $C_{15}H_{14}O_2$ [M+H]$^+$: 227.1067. Found: 227.1067.

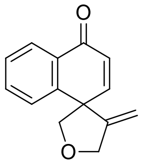

2v

Colorless oil. 99% yield (41.8 mg). ^{1}H NMR (400 MHz, $CDCl_3$) δ 8.16 (d, J = 8.4 Hz, 1H), 7.59-7.48 (m, 2H), 7.44-7.35 (m, 1H), 6.94 (d, J = 10.0 Hz, 1H), 6.47 (d, J = 10.4 Hz, 1H), 5.06 (s, 1H), 4.80-4.65 (m, 3H), 4.22 (d, J = 8.8 Hz, 1H), 4.14 (d, J = 8.8 Hz, 1H). ^{13}C NMR (100 MHz, $CDCl_3$) δ 184.8, 152.1, 149.8, 145.4, 133.0, 131.0, 127.4, 127.3, 127.0, 126.4, 108.5, 80.0, 72.1, 53.1. IR (thin film): ν_{max} (cm^{-1}) = 3068, 3060, 2976, 2853, 1659, 1599, 1455, 1390, 1298, 1157, 1070, 928, 840, 764; HRMS (ESI) calcd for $C_{14}H_{13}O_2$ [M+H]$^+$: 213.0910. Found: 213.0911.

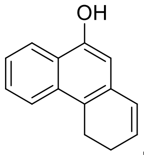

2aa

White solid, M.P. = 98-99 °C. 86% yield (33.7 mg). ^{1}H NMR (400 MHz, $CDCl_3$) δ 8.19 (d, *J* = 8.4 Hz, 1H), 8.00 (d, *J* = 8.4 Hz, 1H), 7.57-7.50 (m, 1H), 7.49-.40 (m, 1H), 6.58 (s, 1H), 6.45 (dt, *J* = 9.2, 2.0 Hz, 1H), 6.18-6.09 (m, 1H), 5.30 (s, 1H), 3.14 (t, *J* = 8.8 Hz, 2H), 2.51-2.42 (m, 2H). ^{13}C NMR (100 MHz, $CDCl_3$) δ 149.7, 132.5, 131.1, 128.8, 128.0, 126.5, 124.2, 123.9, 123.2, 122.6, 122.0, 108.2, 23.3, 22.0. IR (thin film): ν_{max} (cm^{-1}) = 3338, 3064, 3030, 2925, 1951, 1703, 1594, 1519, 1388, 1359, 1310, 1267, 1220, 1064, 852, 764, 710, 637, 613; HRMS (EI) calcd for $C_{14}H_{12}O$ $[M+H]^+$: 196.0888. Found: 196.0886.

Gram-scale reaction of 1g and transformations of 2f

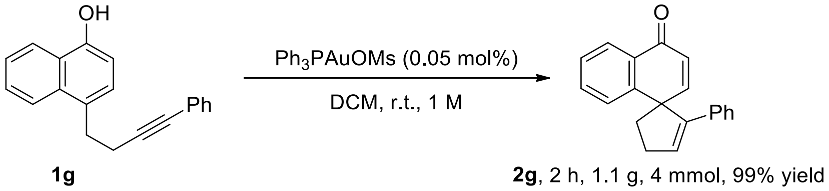

A flame-dried Schlenk tube was cooled down to room temperature under argon. To this tube were added $Ph_3PAuOMs$ (1.1 mg, 0.0002 mmol, 0.05 mol%), 1-naphthol derivatives **1g** (1.1 g, 4 mmol, 1.0 equiv.), and DCM (2.0 mL). Then the reaction mixture was stirred at room temperature in the dark. After completion (monitored by TLC), the crude product was purified by silica gel column chromatography (PE/EtOAc = 10/1) to afford the desired product **2g** (1.1 g, 99% yield).

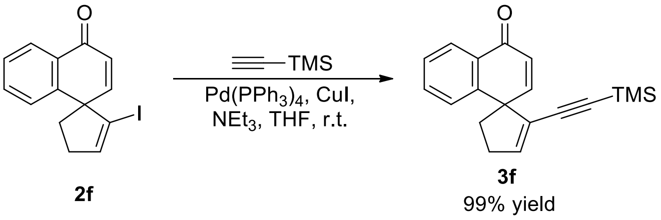

A flame-dried Schlenk tube was cooled down to room temperature under argon. To this tube were added $Pd(PPh_3)_2Cl_2$ (7.0 mg, 0.01 mmol, 5 mol%), CuI (1.9 mg, 0.01 mmol, 5 mol%), **2f** (0.2 mmol, 1.0 equiv.), NEt_3 (2.0 mL), and

ethynyltrimethylsilane (29.5 mg, 0.2 mmol, 1.0 equiv.). Then the reaction mixture was stirred at room temperature. After completion (monitored by TLC), the reaction mixture was filtrated through celite and the solvent was removed under reduced pressure. The crude product was purified by silica gel column chromatography (PE/EtOAc = 10/1) to afford the desired product **3f**.

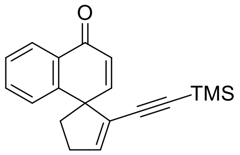

3f

Yellow oil. 99% yield (58.1 mg). ^{1}H NMR (400 MHz, $CDCl_3$) δ 8.14 (d, *J* = 8.0 Hz, 1H), 7.55 (td, *J* = 7.6, 1.6 Hz, 1H), 7.38 (t, *J* = 7.6 Hz, 2H), 6.85 (d, *J* = 10.4 Hz, 1H), 6.44 (d, *J* = 10.0 Hz, 1H), 6.35 (t, *J* = 2.4 Hz, 1H), 2.87-2.72 (m, 2H), 2.50-2.35 (m, 2H), -0.10 (s, 9H). ^{13}C NMR (100 MHz, $CDCl_3$) δ 185.0, 151.9, 147.0, 138.9 132.7, 131.6, 130.0, 127.5, 127.2, 127.1, 126.1, 99.0, 98.4, 57.3, 38.6, 32.4, -0.4. IR (thin film): ν_{max} (cm^{-1}) = 3064, 3034, 2959, 2899, 2851, 2147, 1662, 1600, 1456, 1389, 1300, 1249, 1155, 1128, 1065, 1027, 956, 838, 762, 702, 644, 625; HRMS (ESI) calcd for $C_{19}H_{21}OSi$ $[M+H]^+$: 293.1356. Found: 293.1358.

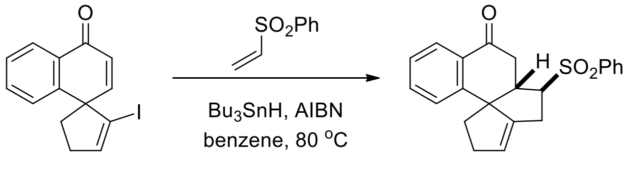

2f **4f**

55% yield

A flame-dried Schlenk tube was cooled down to room temperature under argon. To this tube were added **2f** (128.9 mg, 0.4 mmol, 1.0 equiv.), phenyl vinyl sulfone (672.8 mg, 4 mmol, 10.0 equiv.), and benzene (2.0 mL). Then the reaction mixture was stirred at 80°C. A solution of Bu_3SnH (232.8 mg, 0.8 mmol, 2.0 equiv.) and AIBN (13.1 mg, 0.08 mmol, 0.2 equiv.) in benzene (4 mL) was added into the reaction mixture through syringe pump over 6 h. After completion (monitored by TLC), benzene was removed under reduced pressure and the residue was diluted by Et_2O (10 mL) and saturated KF solution (10 mL). The mixture was stirred vigorously at room

temperature for 2 h.

The mixture was filtrated through filter paper and extracted with Et_2O (10 mL x 3). The combined Et_2O extract was washed with brine, dried over anhydrous Na_2SO_4 and filtrated. After the solvent was concentrated under reduced pressure, the crude product was purified by silica gel column chromatography (PE/EtOAc = 5/1) to afford the desired product **4f**.

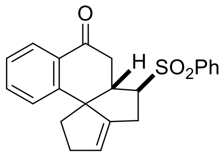

4f

White solid, M.P. = 161-162 °C. 55% yield (80.1 mg). ^{1}H NMR (600 MHz, $CDCl_3$) δ 8.00 (dd, *J* = 7.8, 1.2 Hz, 1H), 7.85 (dd, *J* = 7.8, 1.8 Hz, 2H), 7.67-7.62 (m, 1H), 7.55 (t, *J* = 7.8 Hz, 2H), 7.51 (td, *J* = 7.8, 1.2 Hz, 1H), 7.32-7.28 (m, 1H), 7.25 (d, *J* = 8.4 Hz, 1H), 5.64 (s, 1H), 3.50-3.44 (m, 1H), 2.98-2.90 (m, 1H), 2.89-2.86 (m, 1H), 2.85 (t, *J* = 5.4 Hz, 1H), 2.81-2.75 (m, 2H), 2.73 (dd, *J* = 16.8, 3.6 Hz, 1H), 2.34-2.26 (m, 1H), 2.14-2.02 (m, 2H). ^{13}C NMR (150 MHz, $CDCl_3$) δ 195.7, 149.5, 146.2, 138.6, 134.8, 133.9, 130.7, 129.4, 128.4, 127.2, 127.1, 126.8, 122.7, 67.9, 62.0, 45.8, 38.0, 37.4, 36.6, 25.9. IR (thin film): ν_{max} (cm^{-1}) = 3063, 2924, 2850, 1680, 1598, 1478, 1449, 1410, 1358, 1335, 1289, 1258, 1217, 1177, 1141, 1084, 1020, 996, 972, 941, 923, 889, 841, 796, 762, 744, 725, 688, 667; HRMS (ESI) calcd for $C_{22}H_{24}NO_3S$ $[M+NH_4]^+$: 382.1471. Found: 382.1474.

Gold-catalyzed asymmetric dearomatization of naphthols and HPLC chromatographs

Optimization of reaction conditions

CPA screening

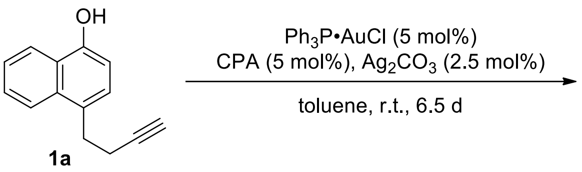

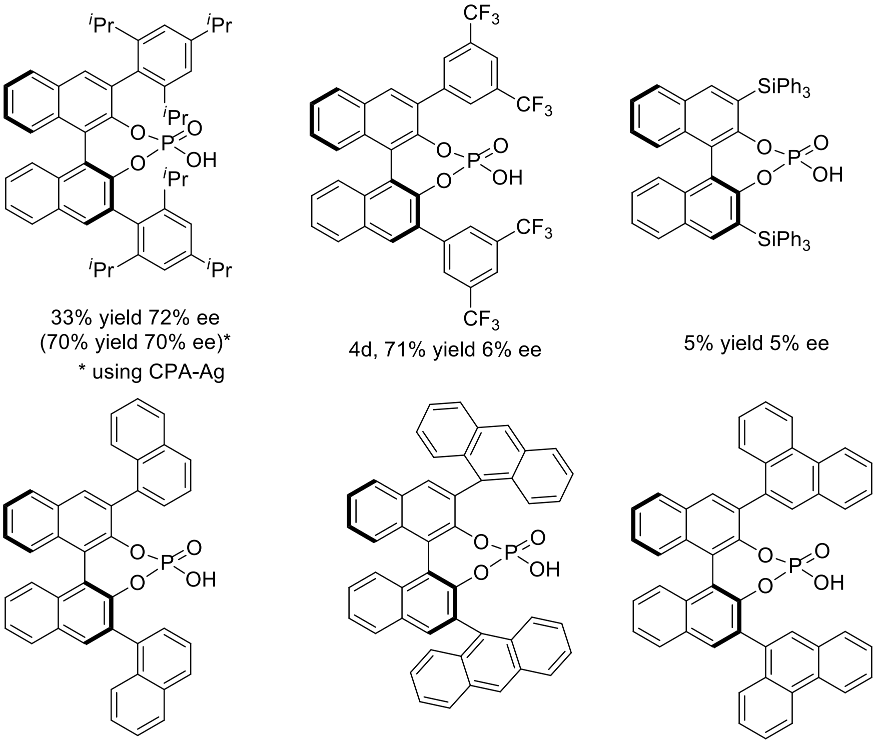

33% yield 72% ee
(70% yield 70% ee)*
* using CPA-Ag

4d, 71% yield 6% ee

5% yield 5% ee

20% yield 0% ee

9% yield 29% ee

29% yield 0% ee

Ligand Screening

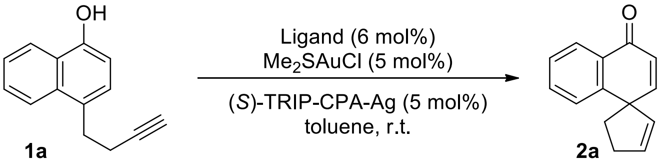

Entry	Ligand	Time	Isolated Yield (%)	ee (%)
1	PPh_3	7 d	70	70
2	$(4\text{-}CF_3C_6H_4)_3P$	7 d	72	85
3	IPr	4d	58	9
4	(2-furyl)$_3$P	4.5 d	49	62
5	$(4\text{-}OMeC_6H_4)_3P$	4.5 d	65	81
6	$(3,5\text{-}(CF_3)_2C_6H_3)_3P$	11 d	22	64
7	JohnPhos	1.5 d	76	36

8	$(2\text{-}MeC_6H_4)_3P$	5 d	22	57
9	SPhos	4d	59	36
10	$(4\text{-}FC_6H_4)_3P$	5.5d	41	81
11	$(C_6F_5)_3P$	6d	Trace	
12	$(4\text{-}MeC_6H_4)_3P$	5d	61	75

Solvent Screening

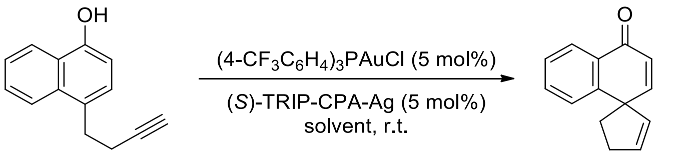

1a **2a**

Entry	Solvent	Time	Isolated Yield (%)	ee (%)
1	toluene	7 d	72	85
2	DCM	41h	82	63
3	THF	4d	trace	
4	MeOH	16h	12	--
5	*o*-xylene	7 d	72	79
6	PhF	7 d	83	60

Concentration Screening

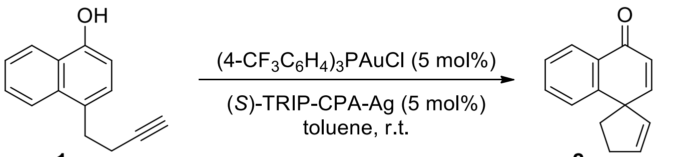

1a **2a**

Entry	Concentration	Time	Yield (%)	ee (%)
1	0.1M	7 d	66	83
2	0.2M	108 h	88	86
3	0.4M	84 h	81	88
4	0.8M	43 h	84	89
5	1.6M	24 h	77	87

Additive Screening

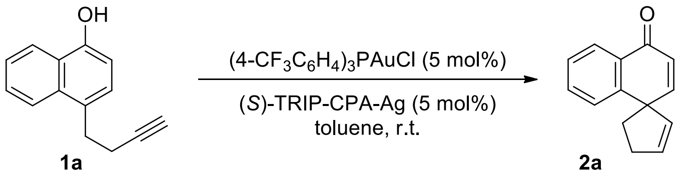

Entry	Additives	Time	Yield (%)	ee (%)	Note
1	none	43h	84	89	
2	3A MS	44.5 h	69	91	25 mg
3	4A MS	44.5 h	71	91	25 mg
4	5A MS	44.5 h	74	91	25 mg
5	HOAc	44.5 h	77	85	5 mol%
6	CF_3CF_2OH	44.5 h	75	88	5 mol%
7	5A MS	49.5 h	67	92	25 mg
8	5A MS	49.5 h	72	92	50 mg
9	5A MS	49.5 h	70	92	100 mg
10	5A MS	70 h	69	92	50 mg

Gold-catalyzed asymmetric dearomatization of naphthols

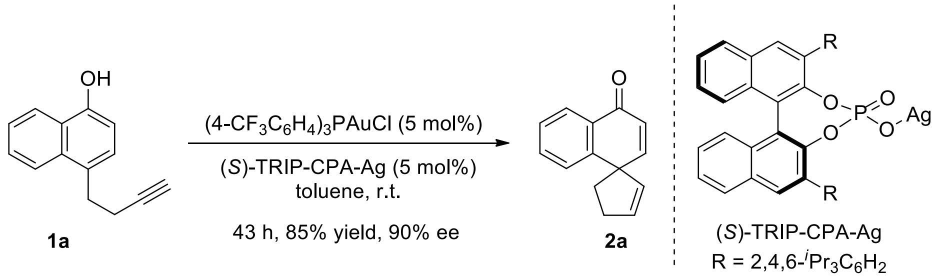

To a flame-dried Schlenk tube at room temperature under argon were added $(4\text{-}CF_3C_6H_4)_3PAuCl$ (13.8 mg, 0.02 mmol, 5 mol%), 1-naphthol derivatives **1a** (78.5 mg, 0.4 mmol, 1.0 equiv.), and toluene (0.5 mL). Then (*S*)-TRIP-CPA-Ag (17.2 mg, 0.02 mmol, 5 mol%) was added last. Then the reaction mixture was stirred at room temperature in the dark. After completion (monitored by TLC), the reaction mixture

was quenched by Bu_4NCl (1M in toluene, 0.1 mL) and loaded on the silica column. The crude product was purified by silica gel column chromatography (PE/EtOAc = 10/1) to afford the desired product **2a**.

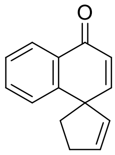

2a

Colorless oil, 85% yield (66.9 mg), 90% ee [Phenomenex Lux 5u Celluloxe-4 PC-4 (0.46 cm x 25 cm), *n*-hexane/2-propanol = 95/5, v = 1.0 mL·min^{-1}, λ = 254 nm, t (major) = 14.27 min, t (minor) = 16.13 min]; $[\alpha]_D^{20}$ = -55.9 (c = 1.0, $CHCl_3$).

HPLC chromatographs

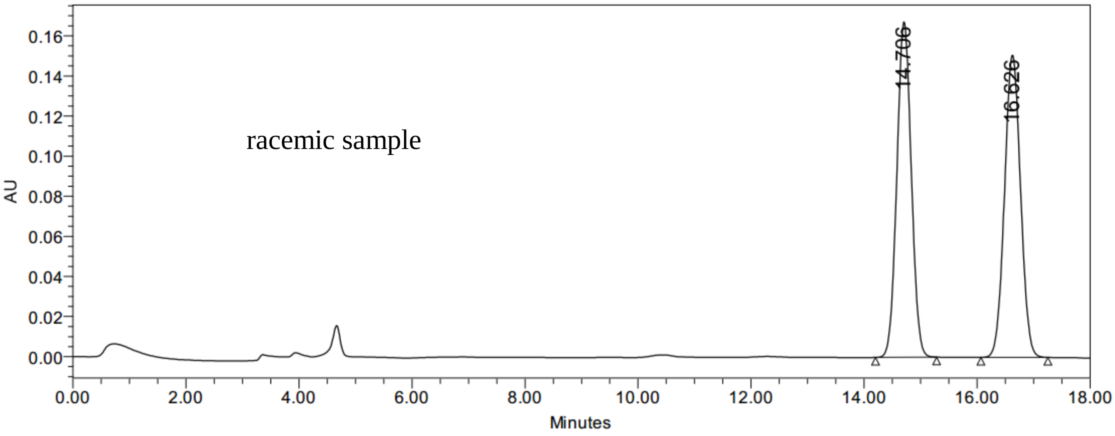

	RT	Area	% Area	Height
1	14.706	2964025	49.93	167475
2	16.626	2972135	50.07	150857

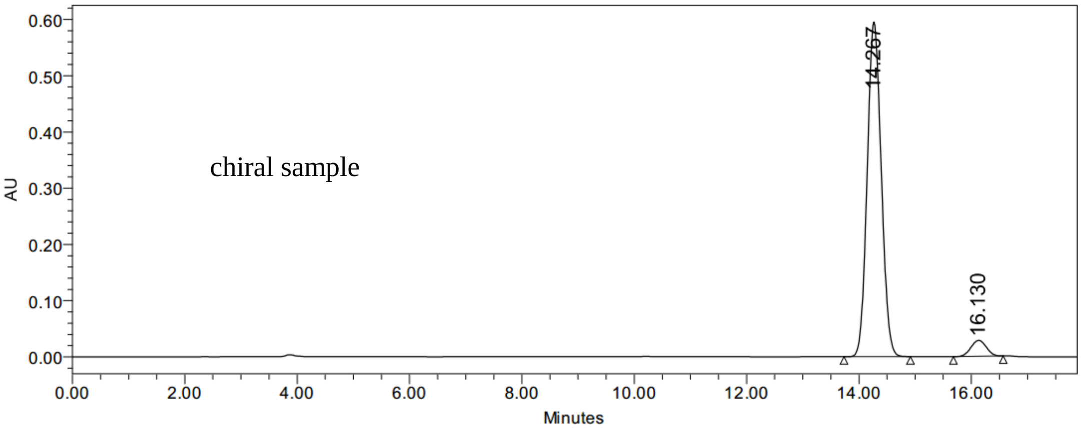

	RT	Area	% Area	Height
1	14.267	10316116	95.00	594898
2	16.130	542528	5.00	28526

Copies of NMR spectra

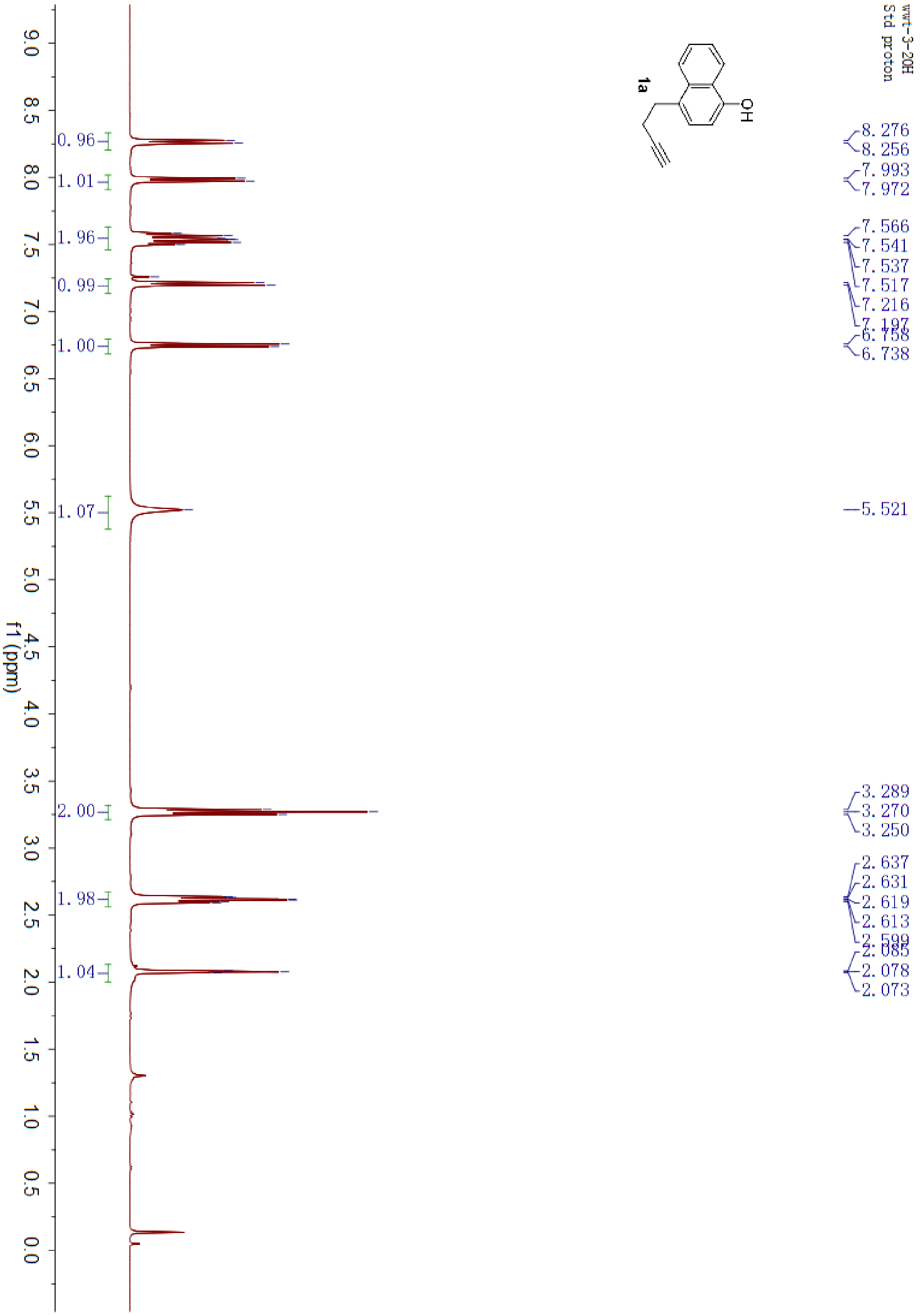

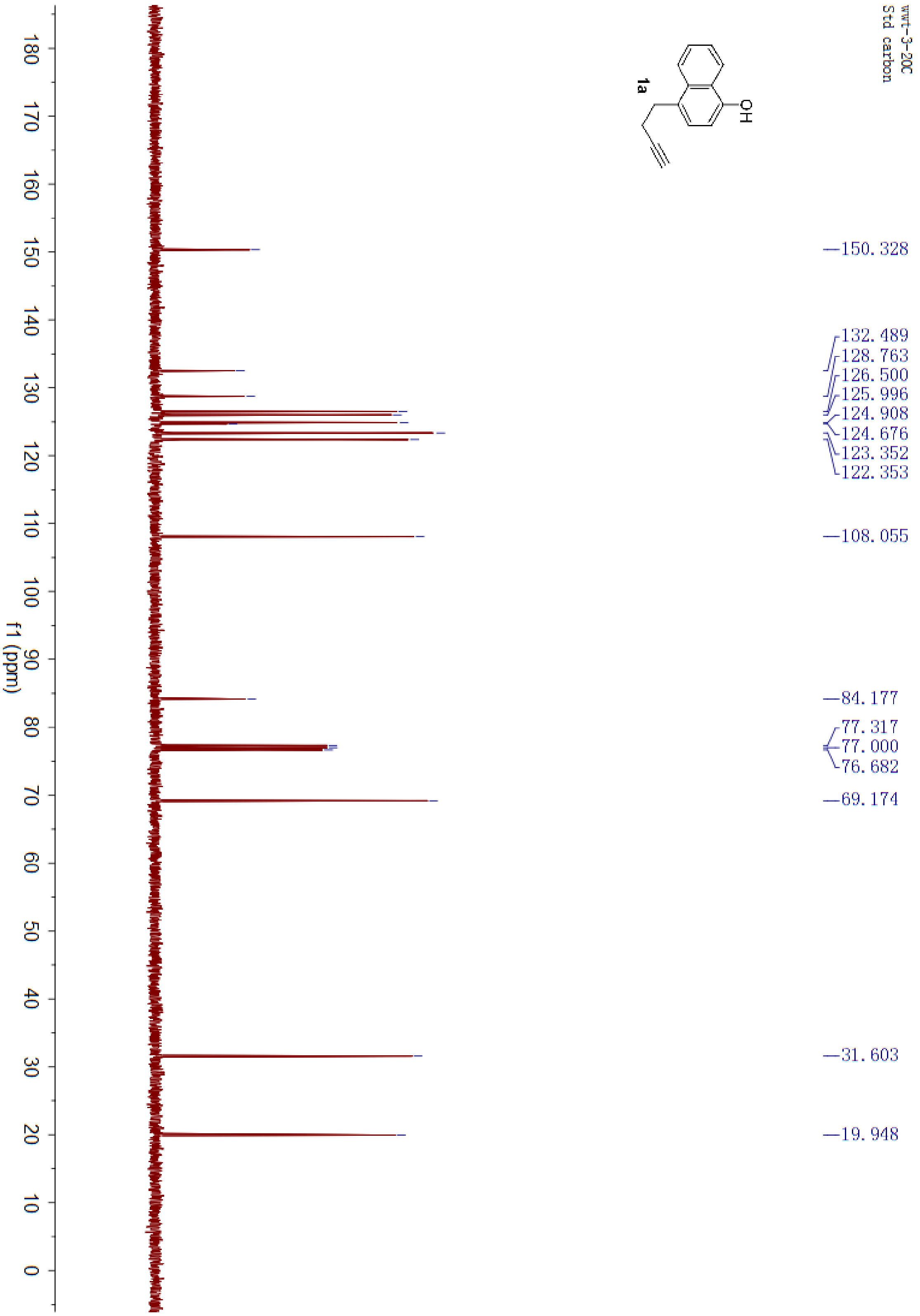

wwt-3-20C
Std carbon
OH
1a
150.328
132.489
128.763
126.500
125.996
124.908
124.676
123.352
122.353
108.055
84.177
77.317
77.000
76.682
69.174
31.603
19.948
f1 (ppm)

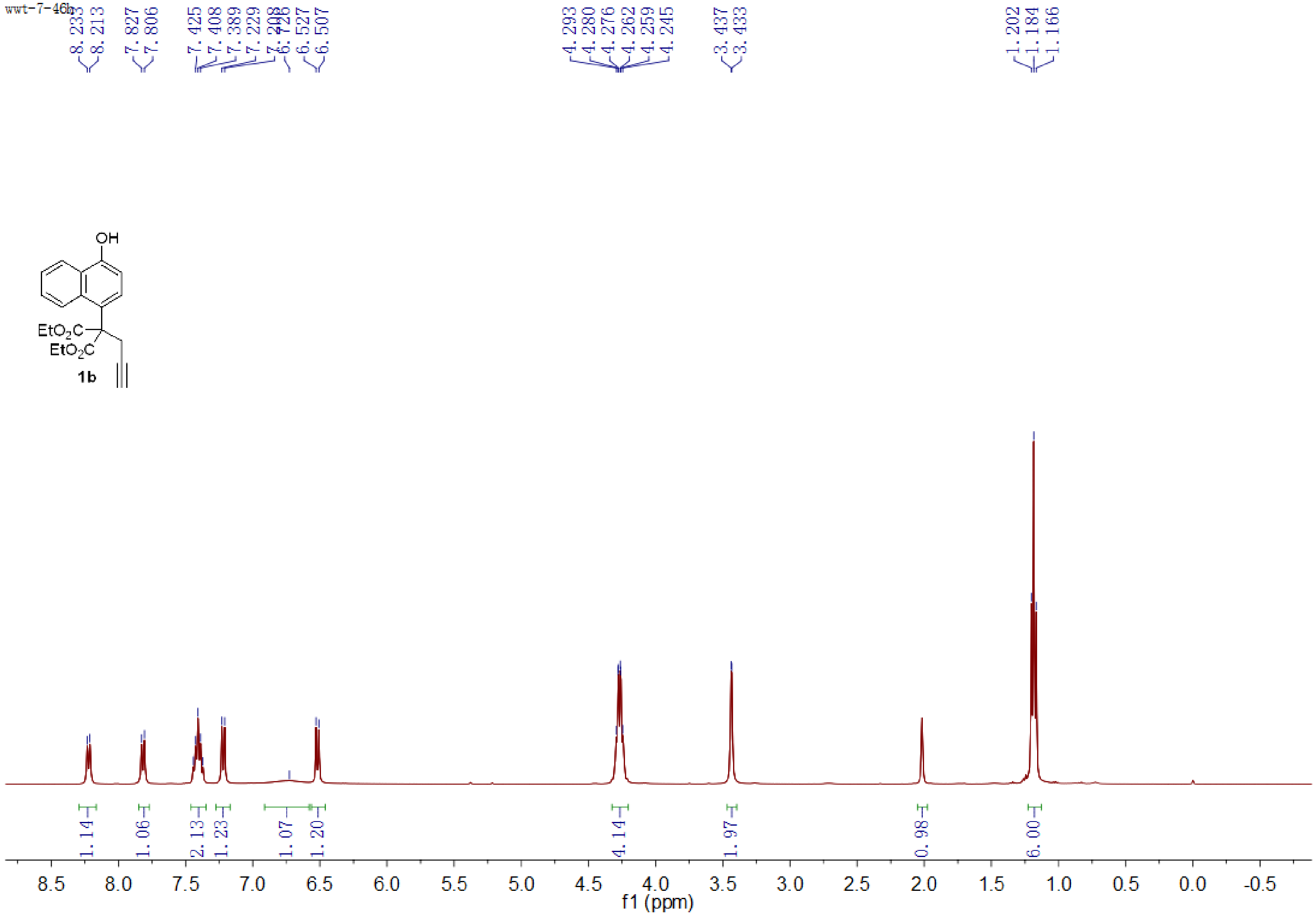

wwt-7-46b
8.233
8.213
7.827
7.806
7.425
7.408
7.389
7.229
7.208
6.726
6.527
6.507
4.293
4.280
4.276
4.262
4.259
4.245
3.437
3.433
1.202
1.184
1.166
1.14
1.06
2.13
1.23
1.07
1.20
4.14
1.97
0.98
6.00
8.5
8.0
7.5
7.0
6.5
6.0
5.5
5.0
4.5
4.0
3.5
3.0
2.5
2.0
1.5
1.0
0.5
0.0
-0.5
f1 (ppm)

1b

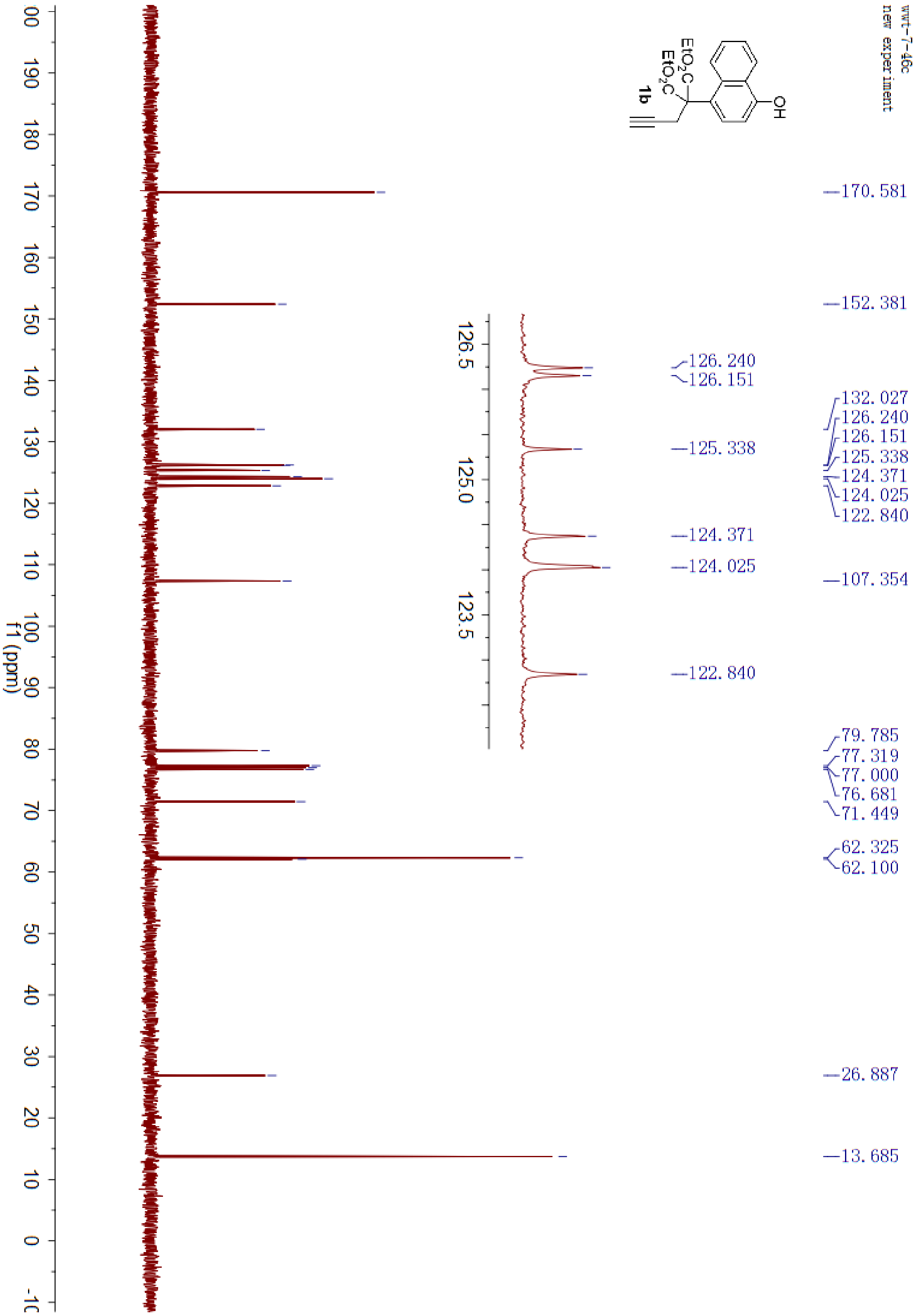

wwt-7-46c
new experiment
OH
EtO_2C
EtO_2C
1b
170.581
152.381
132.027
126.240
126.151
125.338
124.371
124.025
122.840
107.354
79.785
77.319
77.000
76.681
71.449
62.325
62.100
26.887
13.685
126.240
126.151
125.338
124.371
124.025
122.840
126.5
125.0
123.5
f1 (ppm)

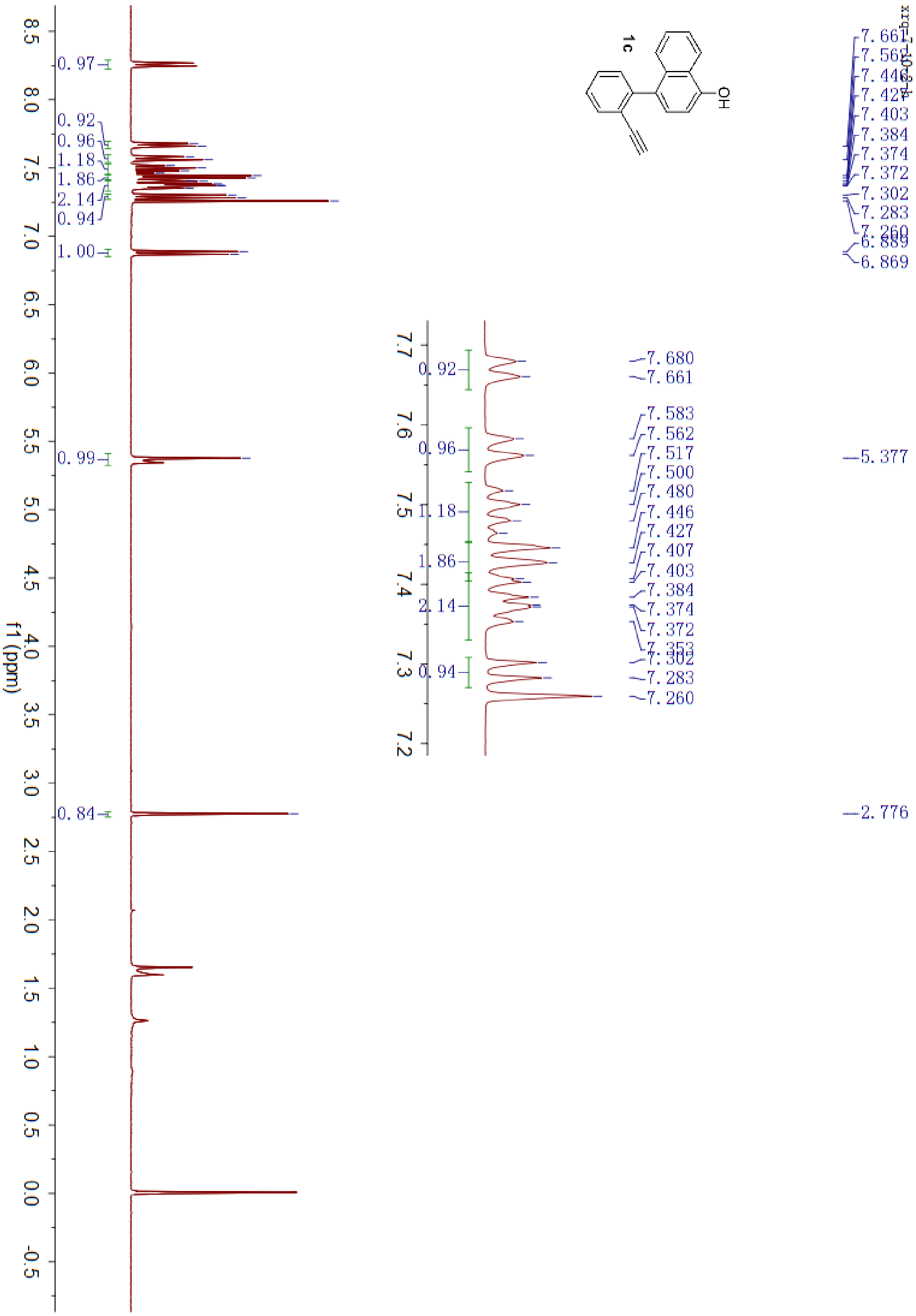
1c
OH
7.661
7.561
7.446
7.424
7.403
7.384
7.374
7.372
7.302
7.283
7.260
6.889
6.869
5.377
2.776
7.680
7.661
7.583
7.562
7.517
7.500
7.480
7.446
7.427
7.407
7.403
7.384
7.374
7.372
7.353
7.302
7.283
7.260
0.97
0.92
0.96
1.18
1.86
2.14
0.94
1.00
0.99
0.84
f1 (ppm)

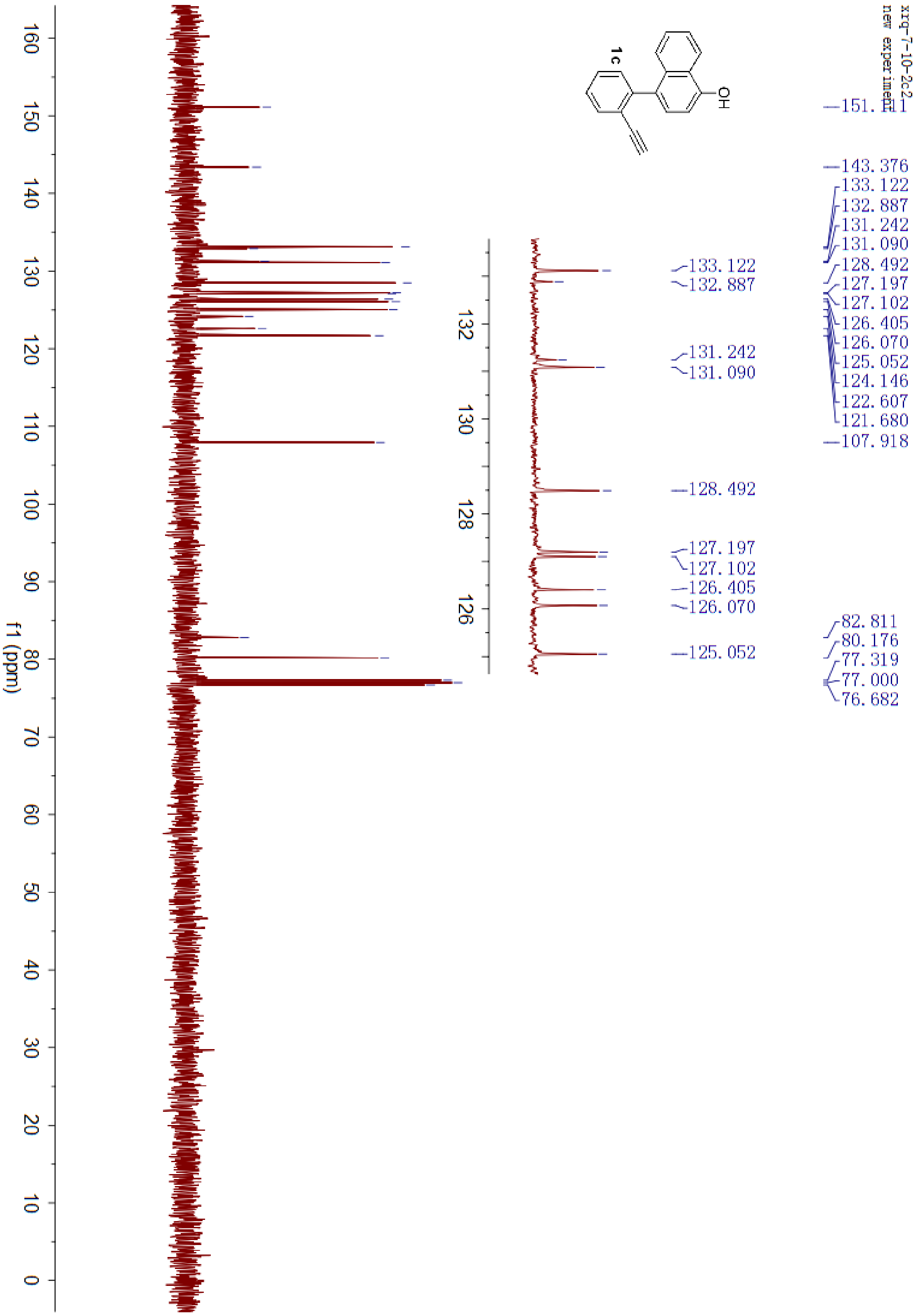
xrq-7-10-2c2
new experiment
151.111
143.376
133.122
132.887
131.242
131.090
128.492
127.197
127.102
126.405
126.070
125.052
124.146
122.607
121.680
107.918
82.811
80.176
77.319
77.000
76.682
1c
OH
f1 (ppm)

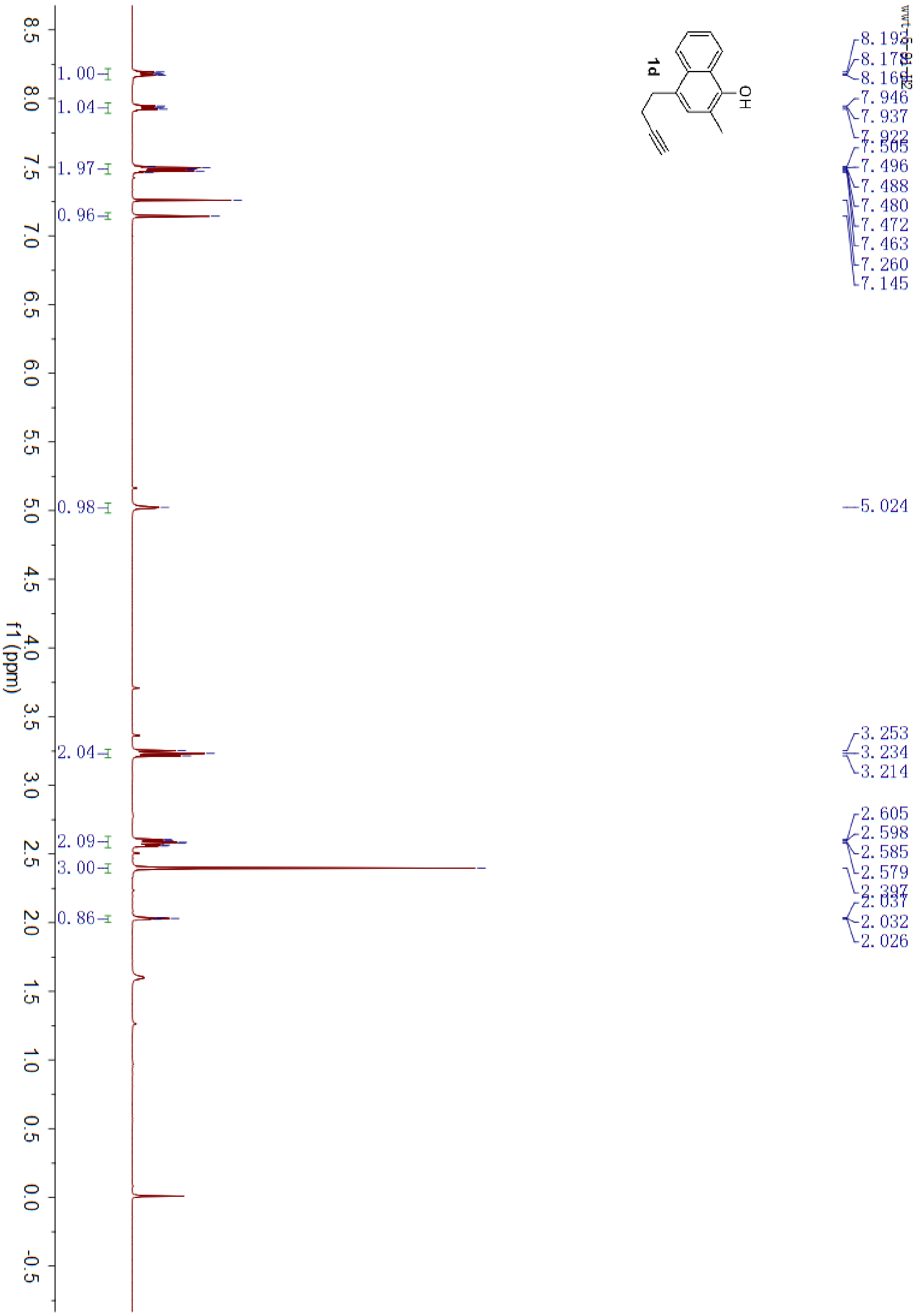
1d
OH
8.5
8.0
7.5
7.0
6.5
6.0
5.5
5.0
4.5
4.0
3.5
3.0
2.5
2.0
1.5
1.0
0.5
0.0
-0.5
f1 (ppm)
1.00
1.04
1.97
0.96
0.98
2.04
2.09
3.00
0.86
7.946
7.937
7.505
7.496
7.488
7.480
7.472
7.463
7.260
7.145
5.024
3.253
3.234
3.214
2.605
2.598
2.585
2.579
2.037
2.032
2.026

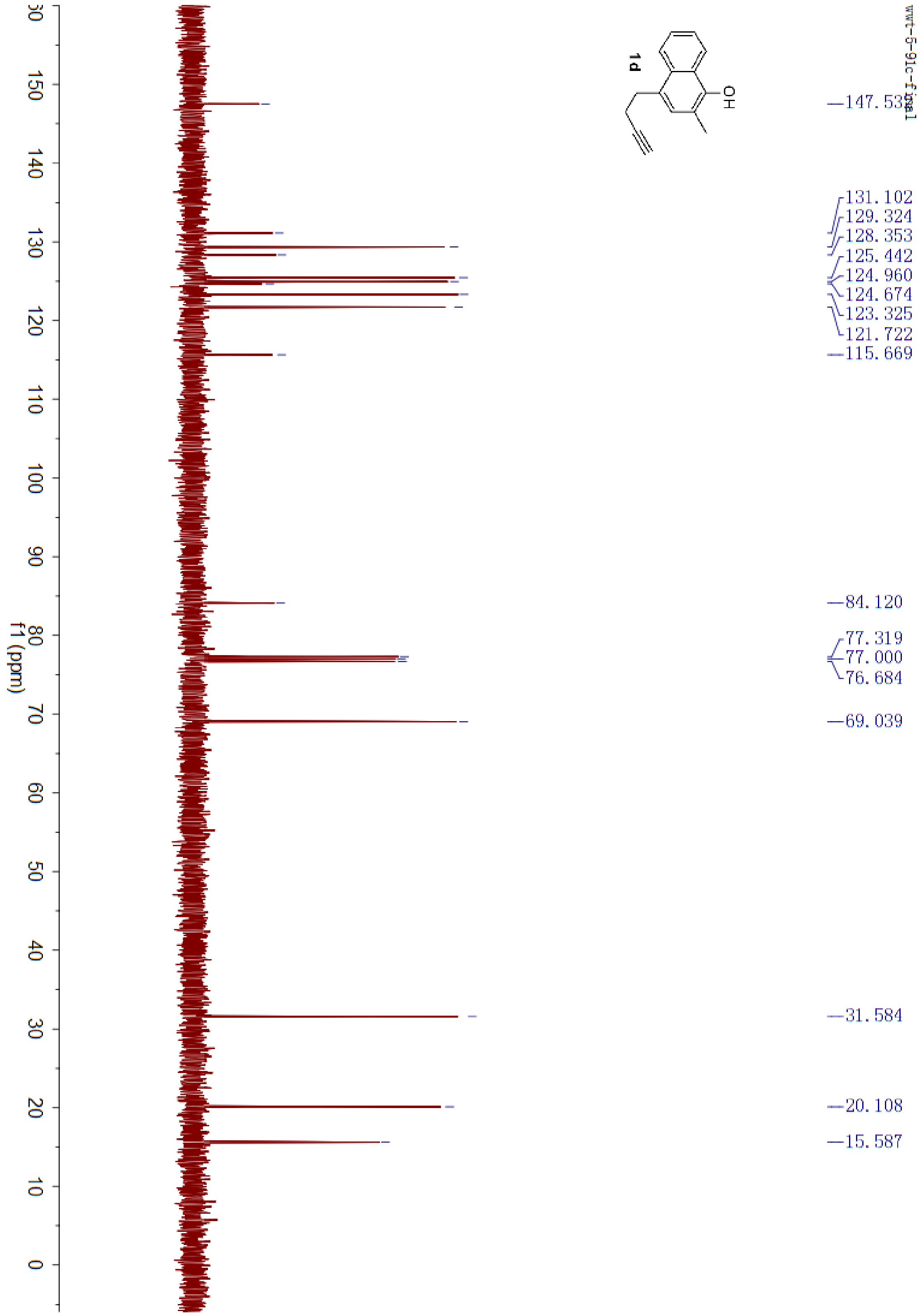
wwt-6-91c-final
1d
OH
147.538
131.102
129.324
128.353
125.442
124.960
124.674
123.325
121.722
115.669
84.120
77.319
77.000
76.684
69.039
31.584
20.108
15.587
f1 (ppm)

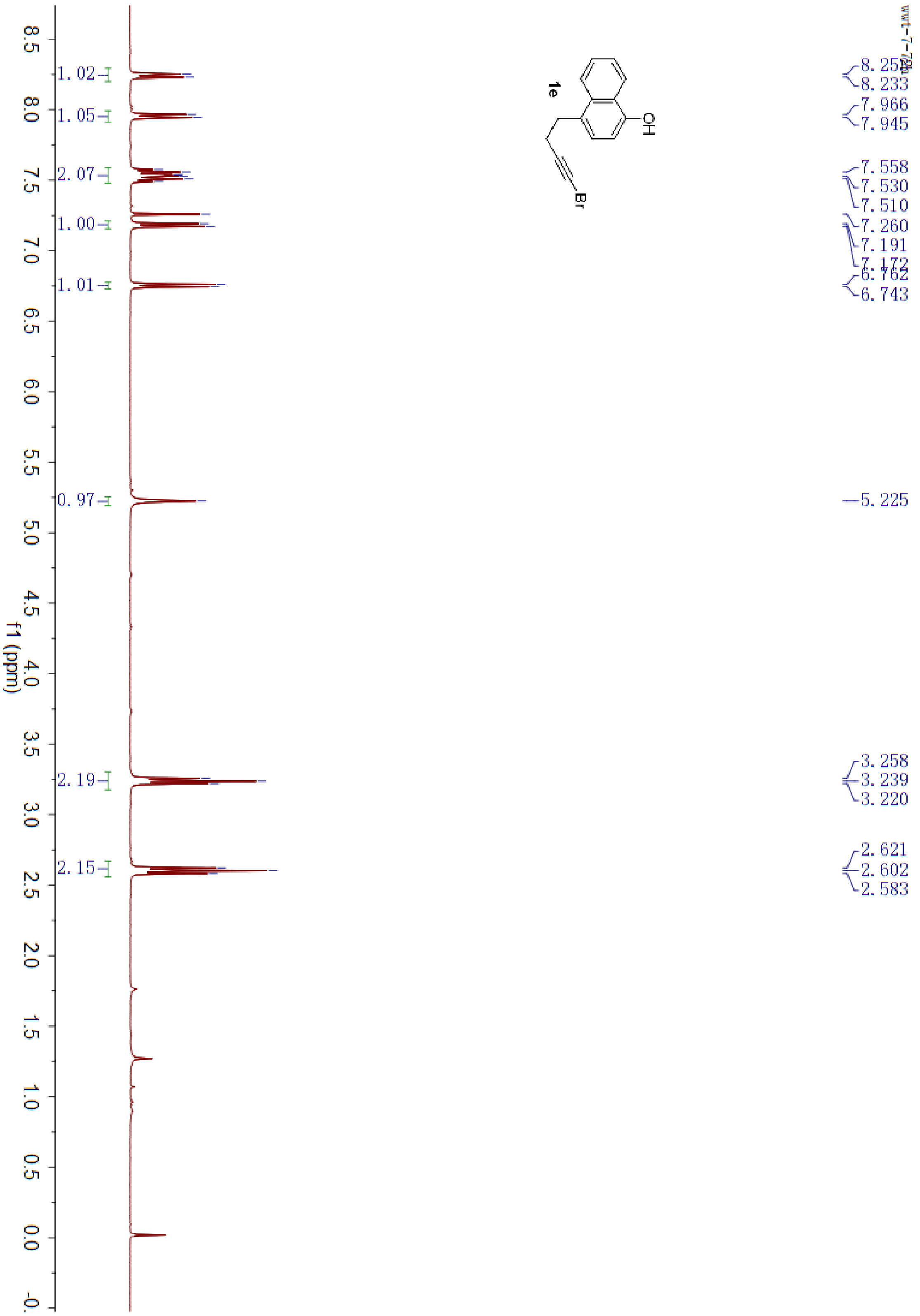
1e
OH
Br
8.233
7.966
7.945
7.558
7.530
7.510
7.260
7.191
6.743
5.225
3.258
3.239
3.220
2.621
2.602
2.583
1.02
1.05
2.07
1.00
1.01
0.97
2.19
2.15
f1 (ppm)

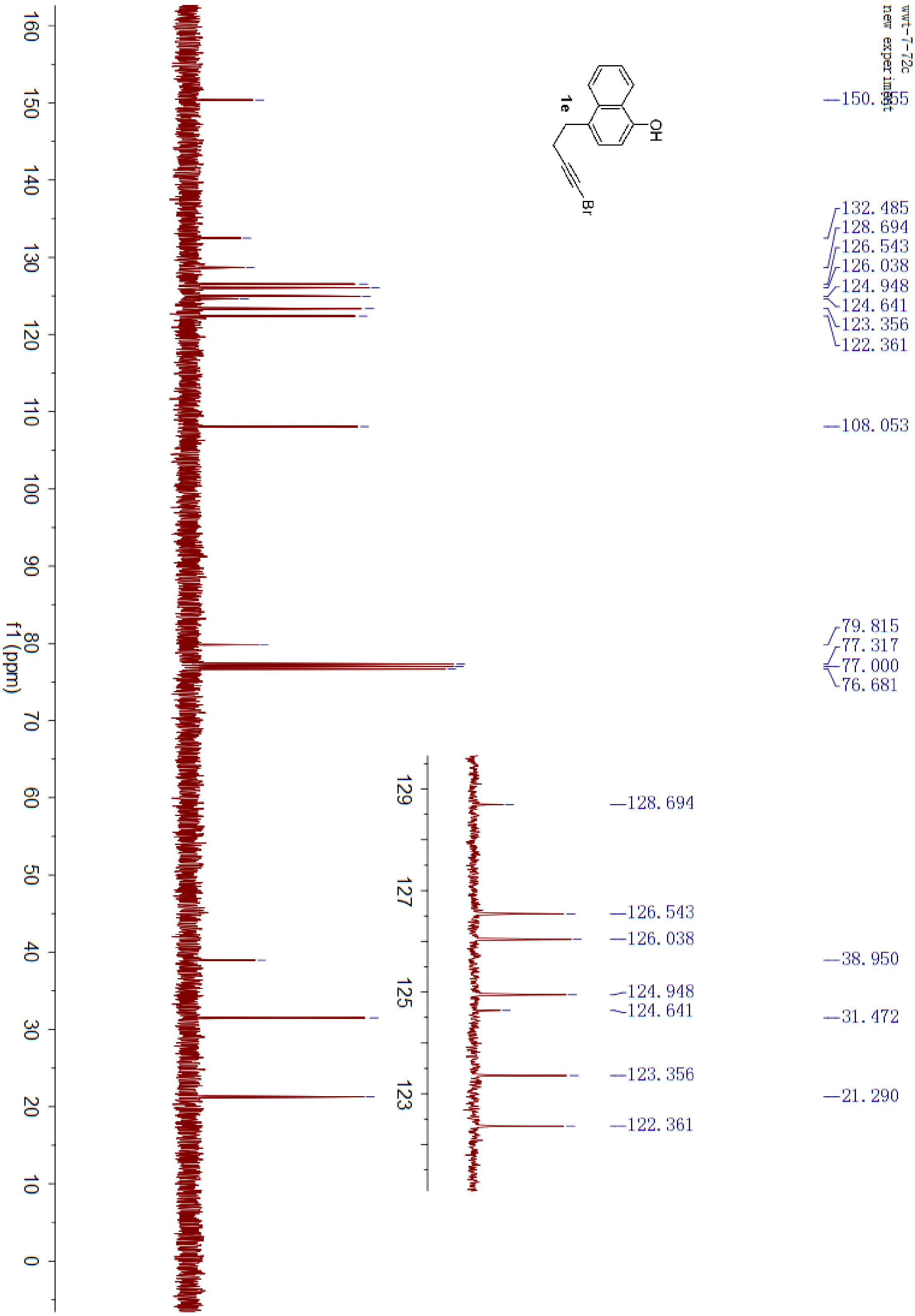
wwt-7-72c
new experiment
1e
OH
Br
132.485
128.694
126.543
126.038
124.948
124.641
123.356
122.361
108.053
79.815
77.317
77.000
76.681
38.950
31.472
21.290
f1 (ppm)

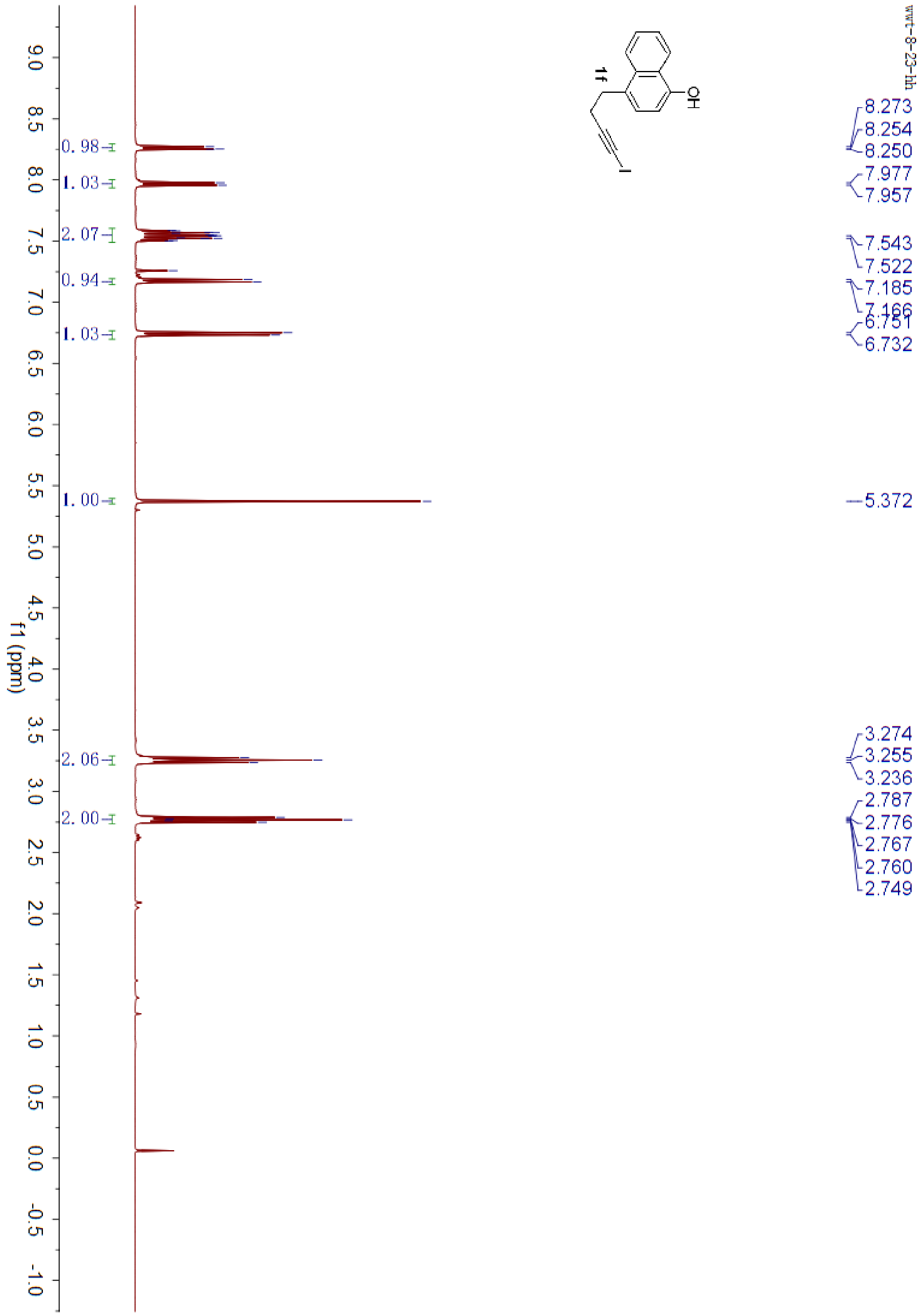
wwt-8-23-hh
1f
OH
I
8.273
8.254
8.250
7.977
7.957
7.543
7.522
7.185
7.166
6.751
6.732
5.372
3.274
3.255
3.236
2.787
2.776
2.767
2.760
2.749
0.98
1.03
2.07
0.94
1.03
1.00
2.06
2.00
f1 (ppm)

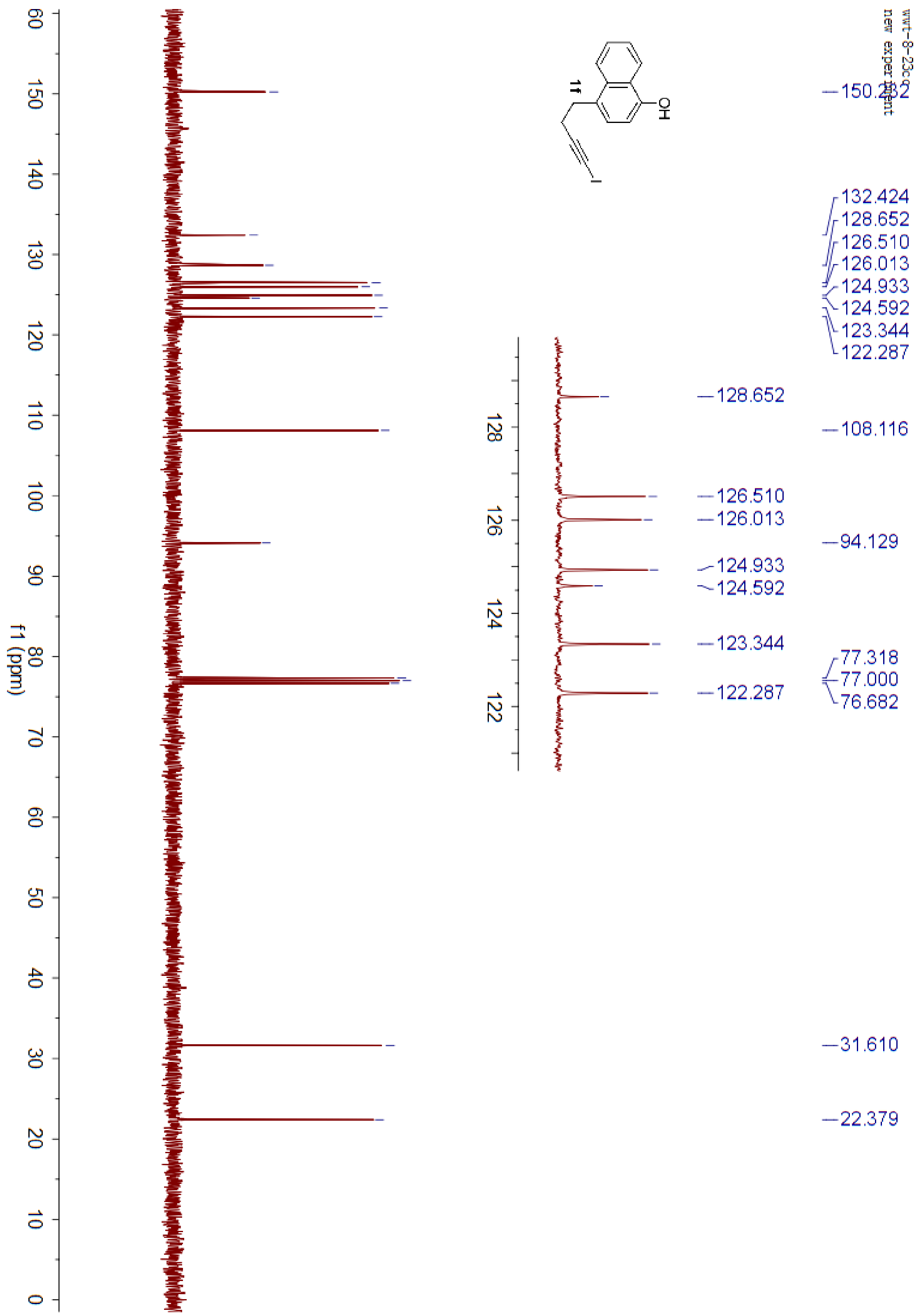

wwt-8-23cq
new experiment
1f
OH
I
150.232
132.424
128.652
126.510
126.013
124.933
124.592
123.344
122.287
108.116
94.129
77.318
77.000
76.682
31.610
22.379
f1 (ppm)

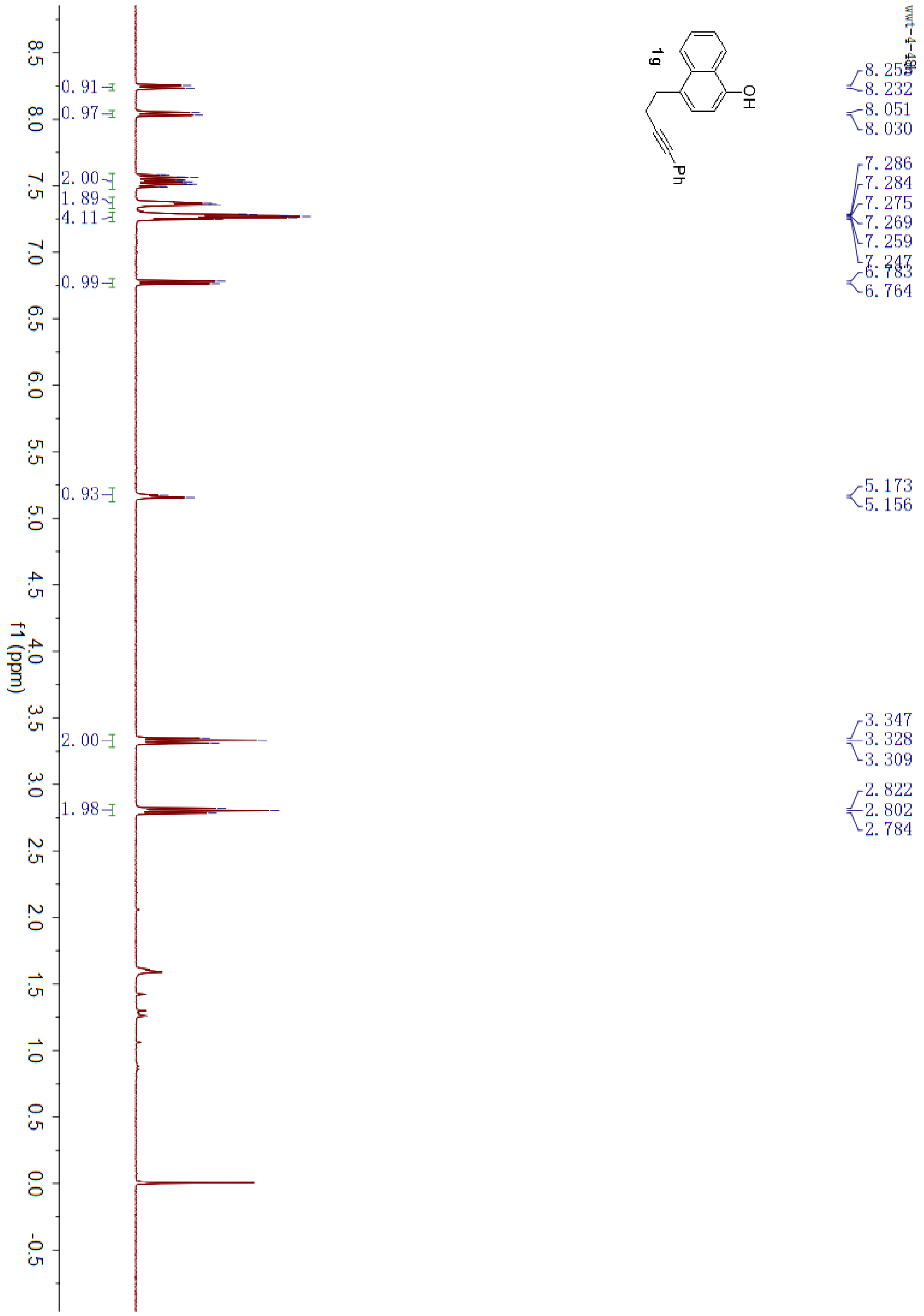
wwt-4-48h
8.255
8.232
8.051
8.030
7.286
7.284
7.275
7.269
7.259
7.247
6.783
6.764
5.173
5.156
3.347
3.328
3.309
2.822
2.802
2.784
OH
Ph
1g
0.91
0.97
2.00
1.89
4.11
0.99
0.93
2.00
1.98
8.5
8.0
7.5
7.0
6.5
6.0
5.5
5.0
4.5
4.0
3.5
3.0
2.5
2.0
1.5
1.0
0.5
0.0
-0.5
f1 (ppm)

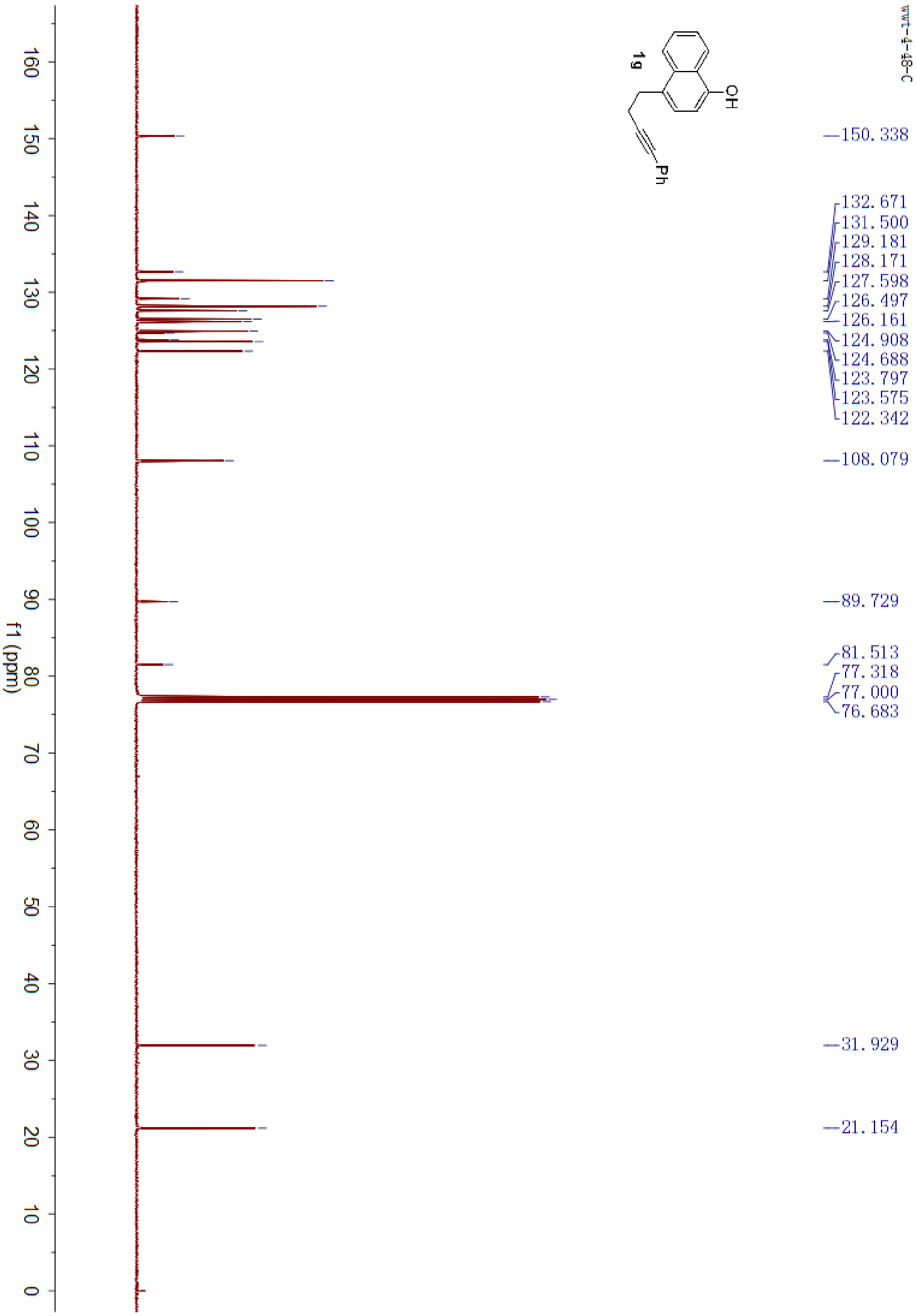
wwt-4-48-C
OH
Ph
1g
150.338
132.671
131.500
129.181
128.171
127.598
126.497
126.161
124.908
124.688
123.797
123.575
122.342
108.079
89.729
81.513
77.318
77.000
76.683
31.929
21.154
f1 (ppm)

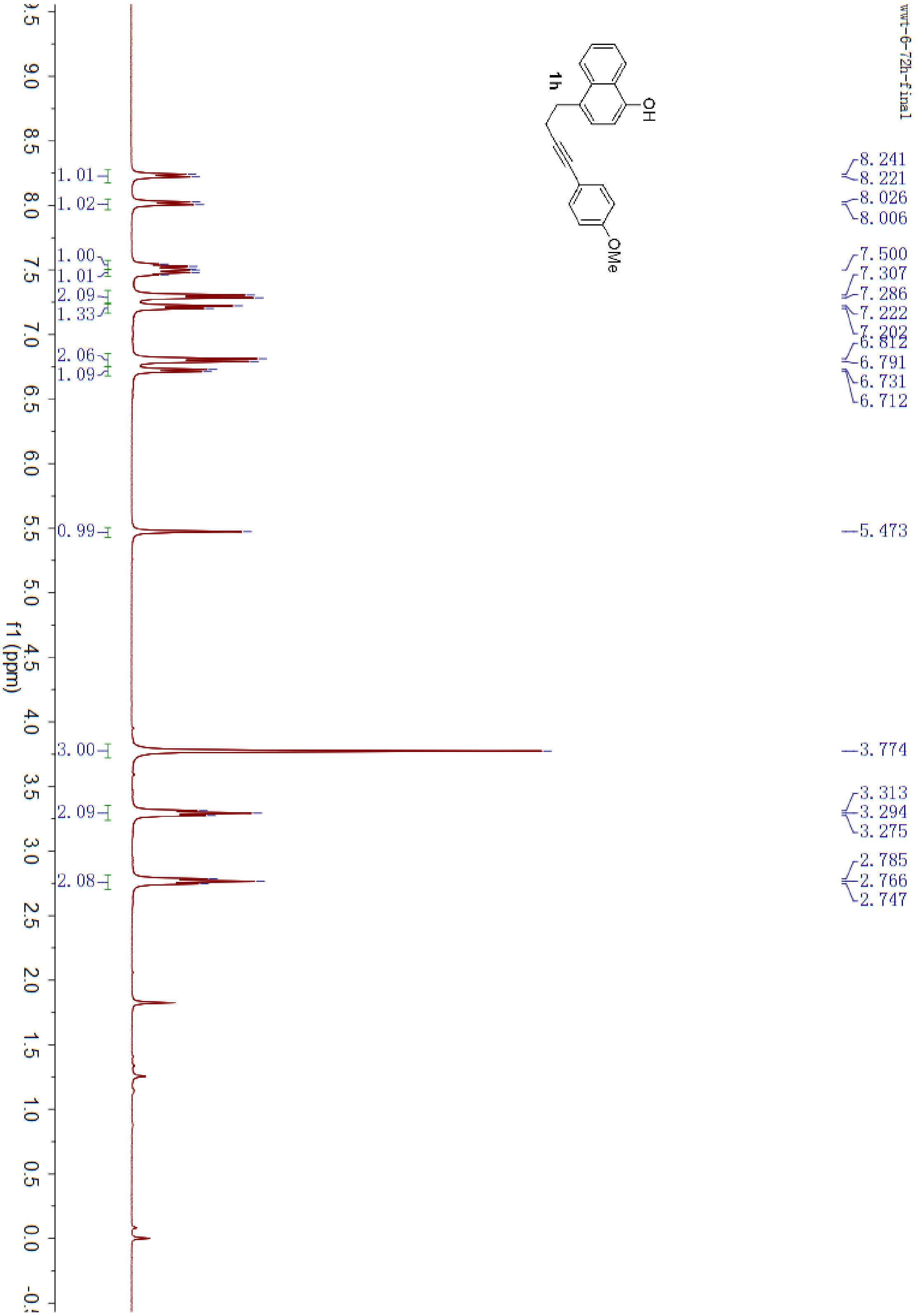
wwt-6-72h-final
1h
OH
OMe
8.241
8.221
8.026
8.006
7.500
7.307
7.286
7.222
7.202
6.812
6.791
6.731
6.712
5.473
3.774
3.313
3.294
3.275
2.785
2.766
2.747
1.01
1.02
1.00
1.01
2.09
1.33
2.06
1.09
0.99
3.00
2.09
2.08
f1 (ppm)

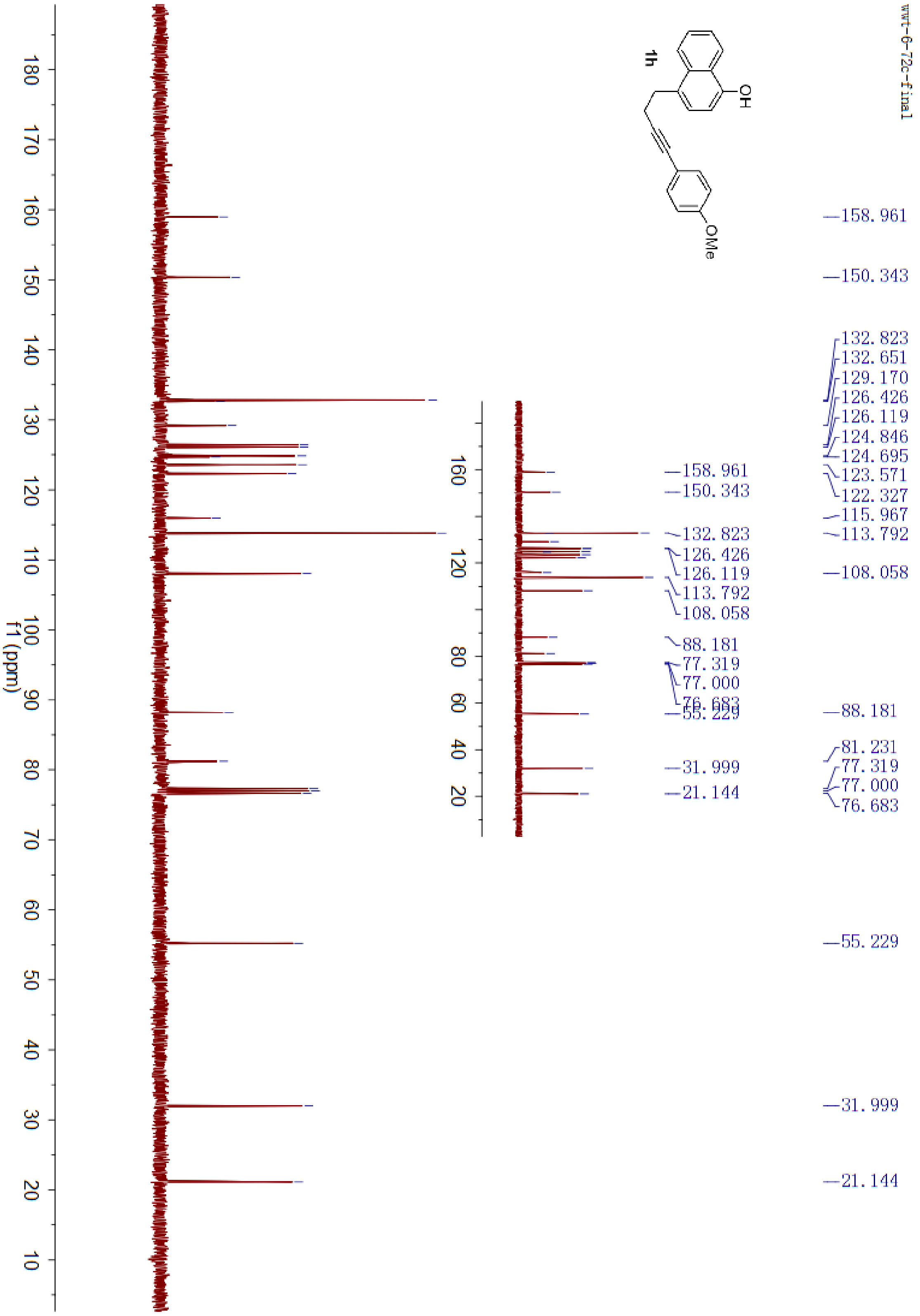
wwt-6-72c-final
1h
OH
OMe
158.961
150.343
132.823
132.651
129.170
126.426
126.119
124.846
124.695
123.571
122.327
115.967
113.792
108.058
88.181
81.231
77.319
77.000
76.683
55.229
31.999
21.144
f1 (ppm)

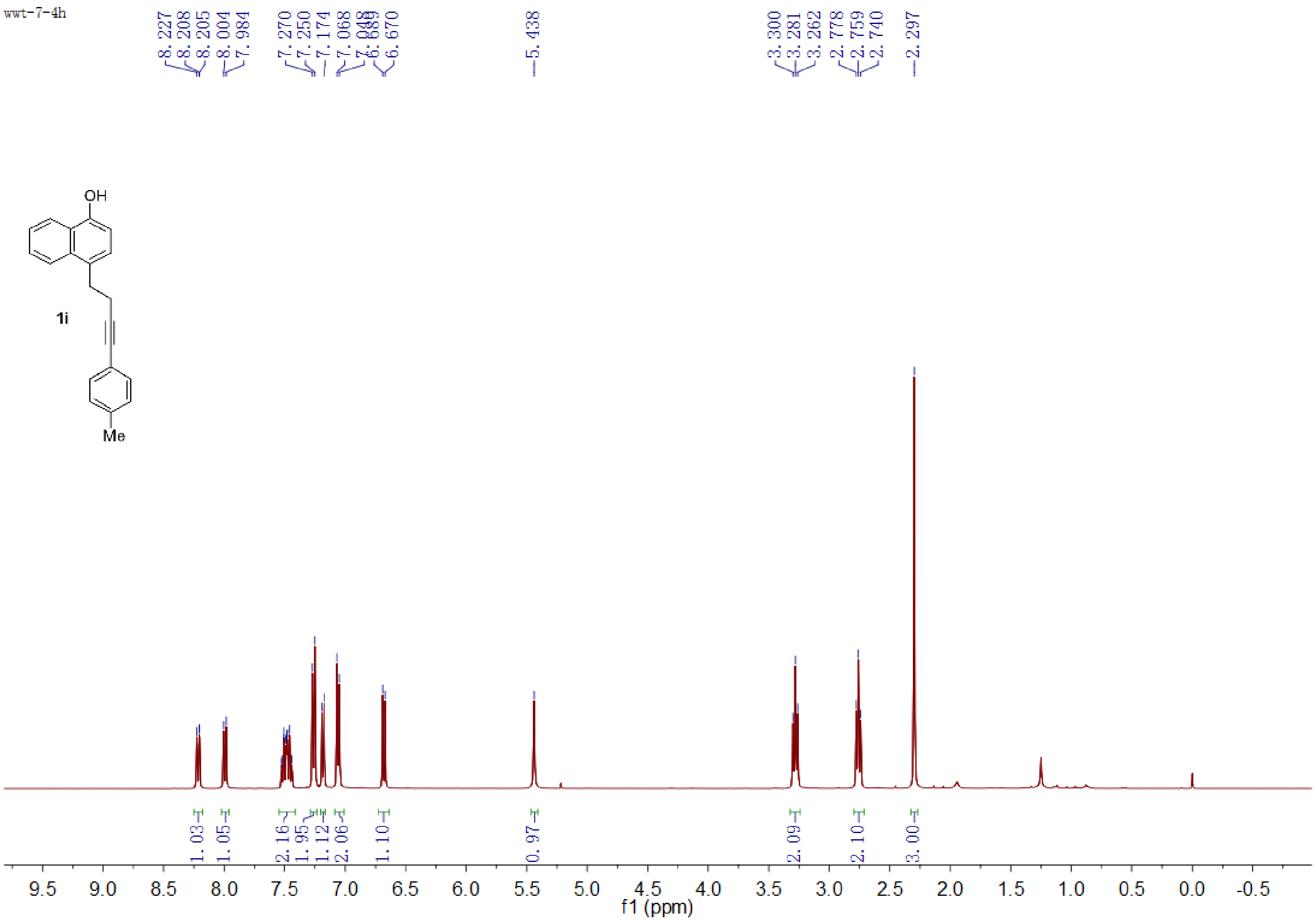
wwt-7-4h
8.227
8.208
8.205
8.004
7.984
7.270
7.250
7.174
7.068
7.048
6.689
6.670
5.438
3.300
3.281
3.262
2.778
2.759
2.740
2.297
OH
1i
Me
1.03
1.05
2.16
1.95
1.12
2.06
1.10
0.97
2.09
2.10
3.00
9.5
9.0
8.5
8.0
7.5
7.0
6.5
6.0
5.5
5.0
4.5
4.0
3.5
3.0
2.5
2.0
1.5
1.0
0.5
0.0
-0.5
f1 (ppm)

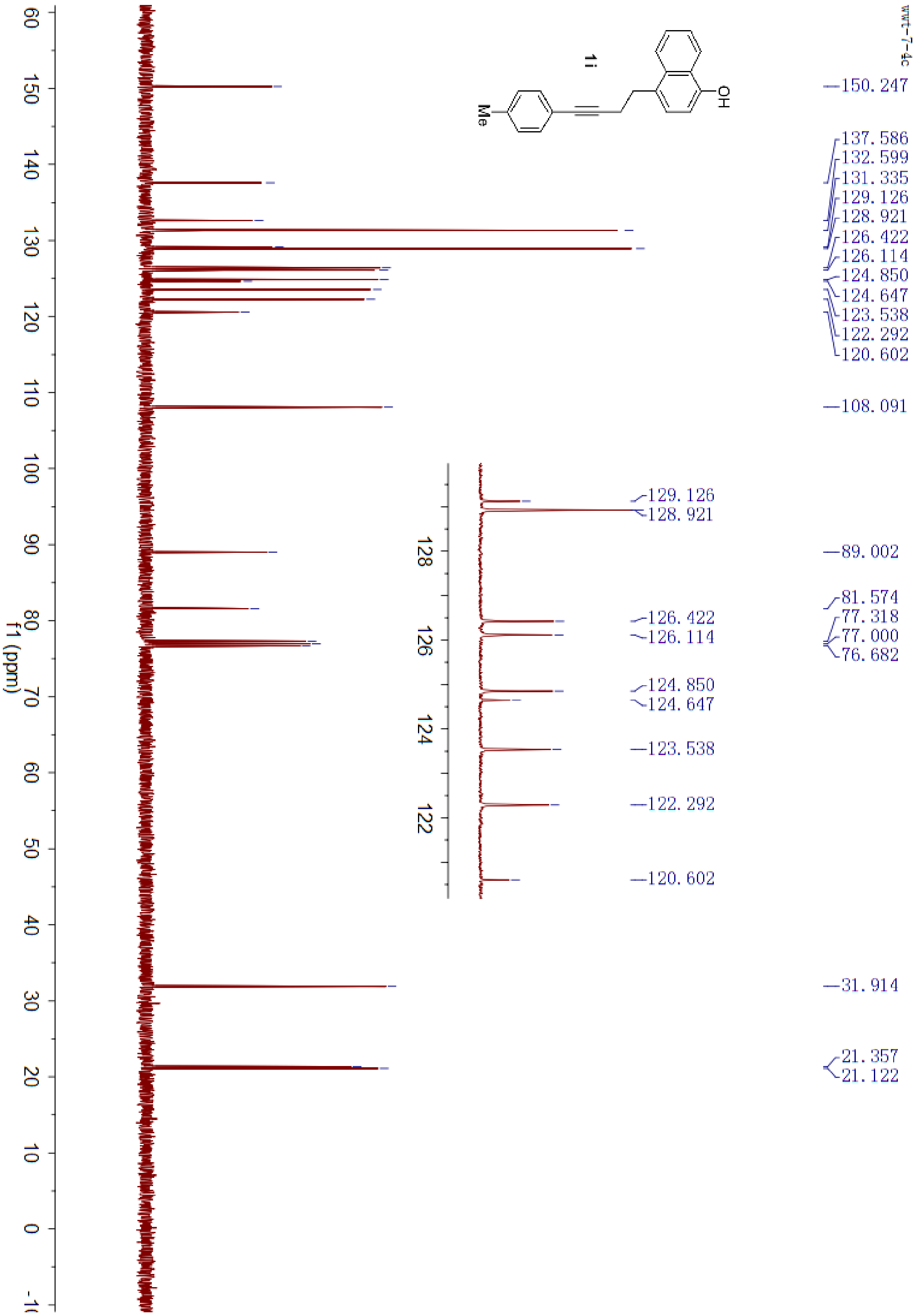
wwt-7-4c
1i
OH
Me
150.247
137.586
132.599
131.335
129.126
128.921
126.422
126.114
124.850
124.647
123.538
122.292
120.602
108.091
89.002
81.574
77.318
77.000
76.682
31.914
21.357
21.122
f1 (ppm)

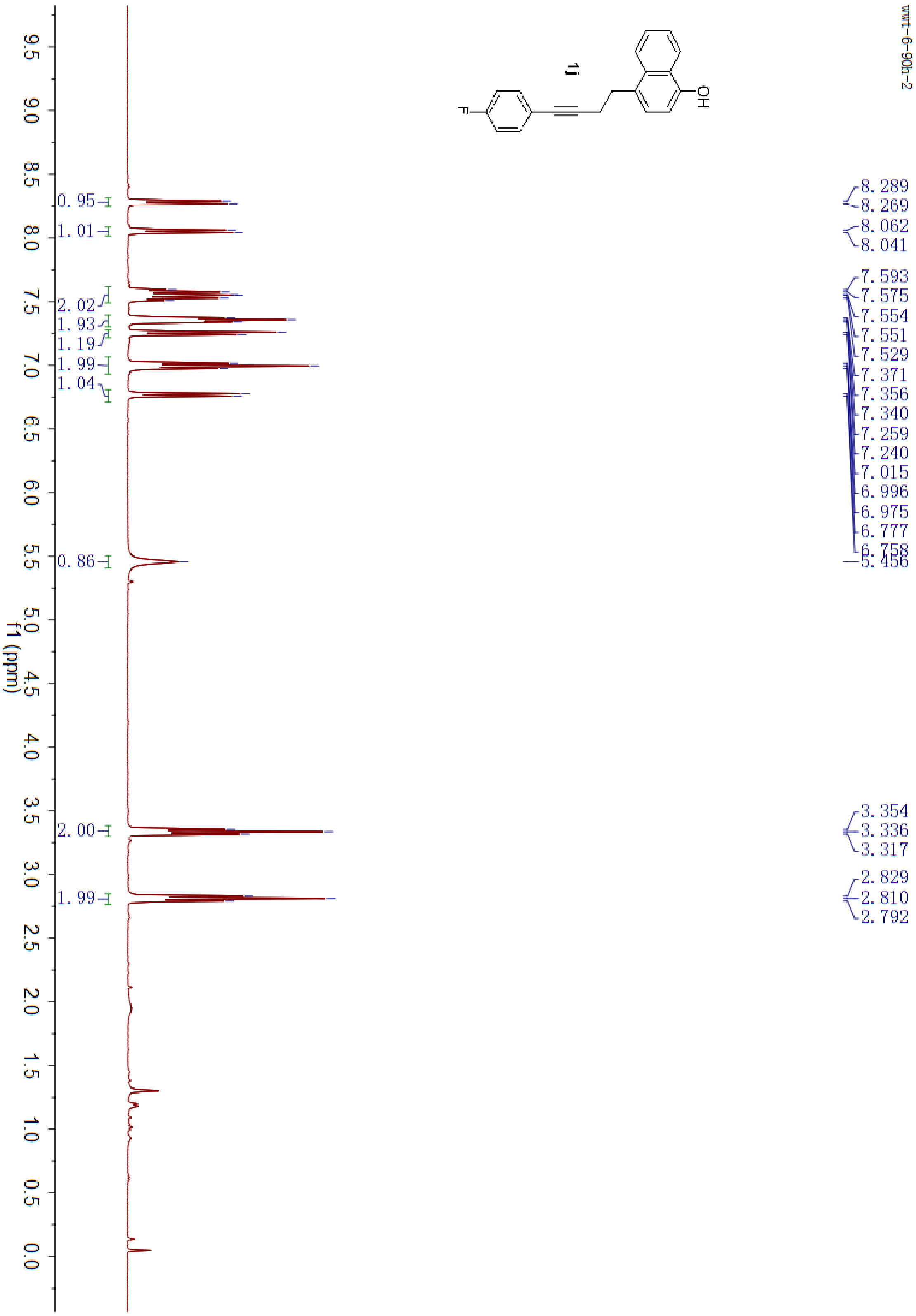
wwt-6-90h-2
1j
OH
F
8.289
8.269
8.062
8.041
7.593
7.575
7.554
7.551
7.529
7.371
7.356
7.340
7.259
7.240
7.015
6.996
6.975
6.777
6.758
5.456
3.354
3.336
3.317
2.829
2.810
2.792
0.95
1.01
2.02
1.93
1.19
1.99
1.04
0.86
2.00
1.99
f1 (ppm)

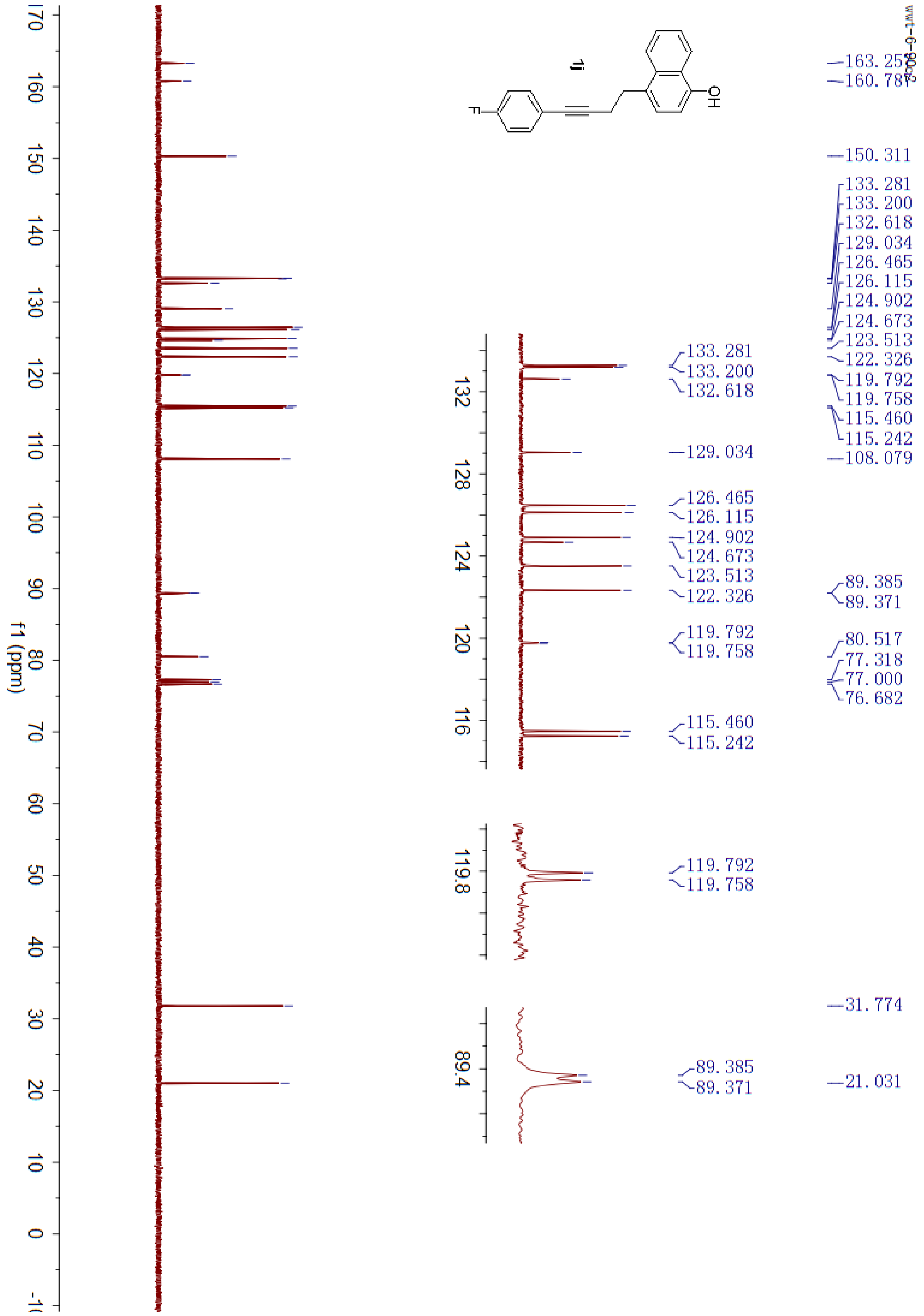
1j
OH
F
163.258
160.787
150.311
133.281
133.200
132.618
129.034
126.465
126.115
124.902
124.673
123.513
122.326
119.792
119.758
115.460
115.242
108.079
89.385
89.371
80.517
77.318
77.000
76.682
31.774
21.031
f1 (ppm)

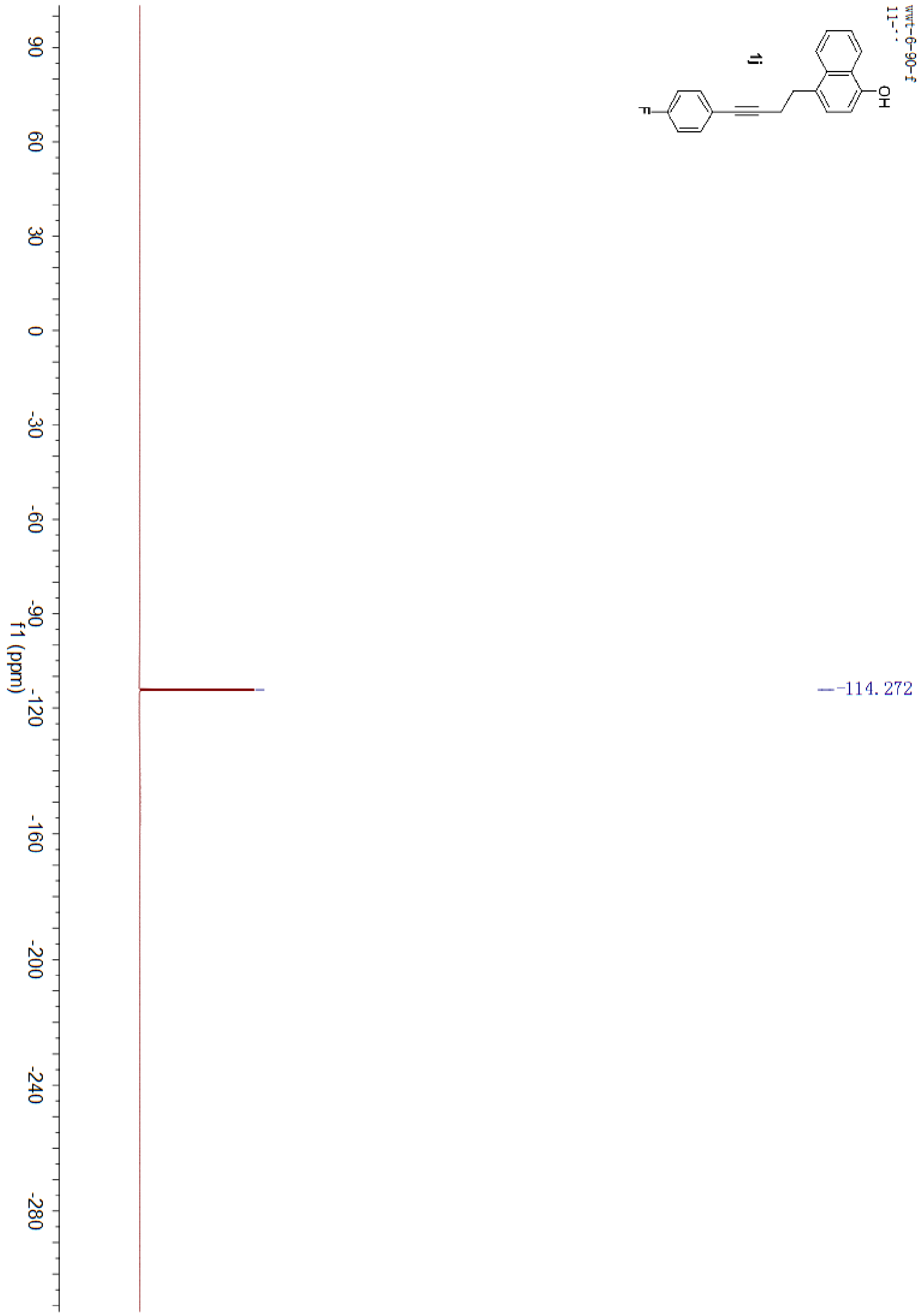

wwt-6-90-f
1j
OH
F
−114.272
f1 (ppm)

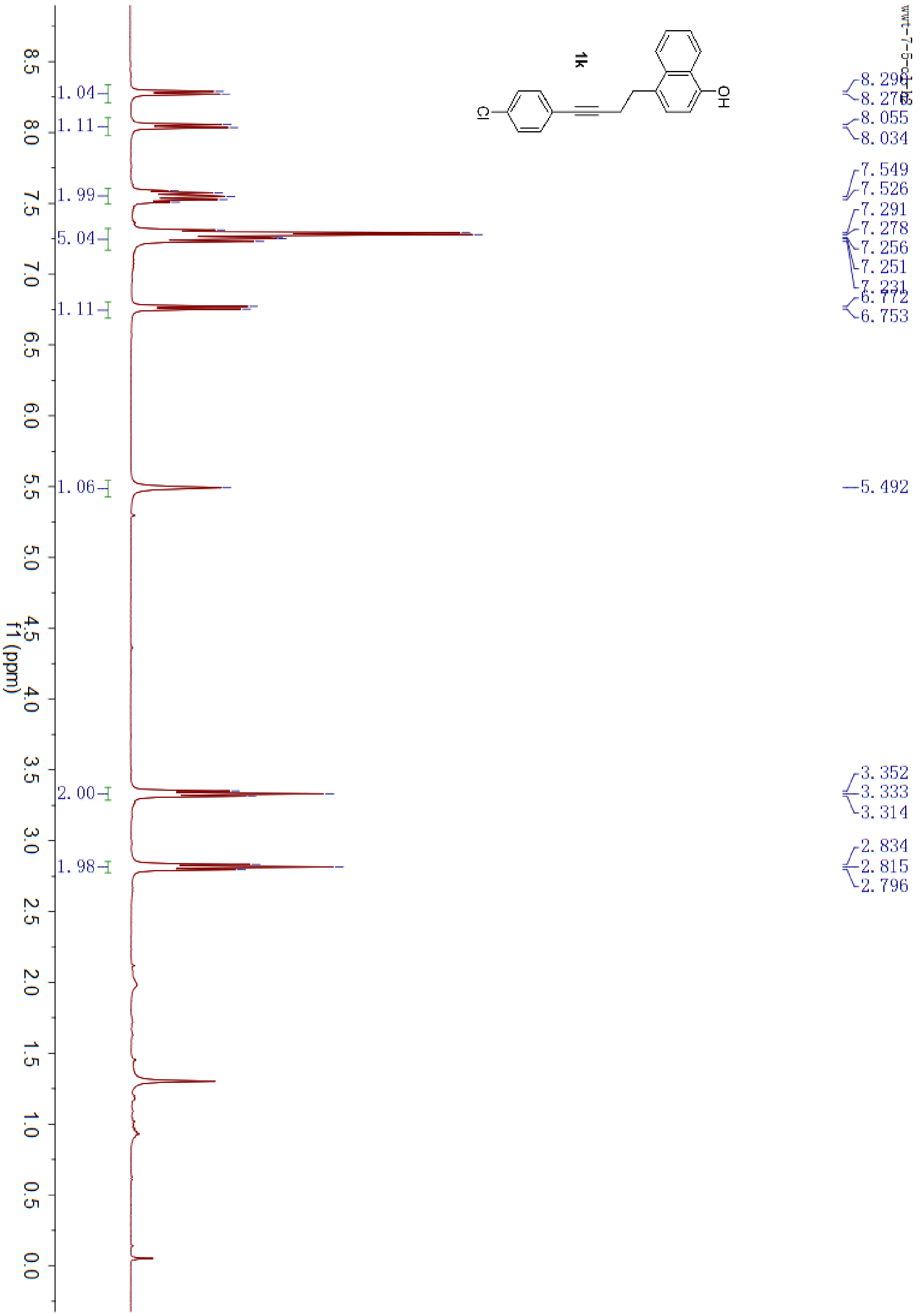
1k
OH
Cl
8.290
8.276
8.055
8.034
7.549
7.526
7.291
7.278
7.256
7.251
7.231
6.772
6.753
5.492
3.352
3.333
3.314
2.834
2.815
2.796
1.04
1.11
1.99
5.04
1.11
1.06
2.00
1.98
f1 (ppm)

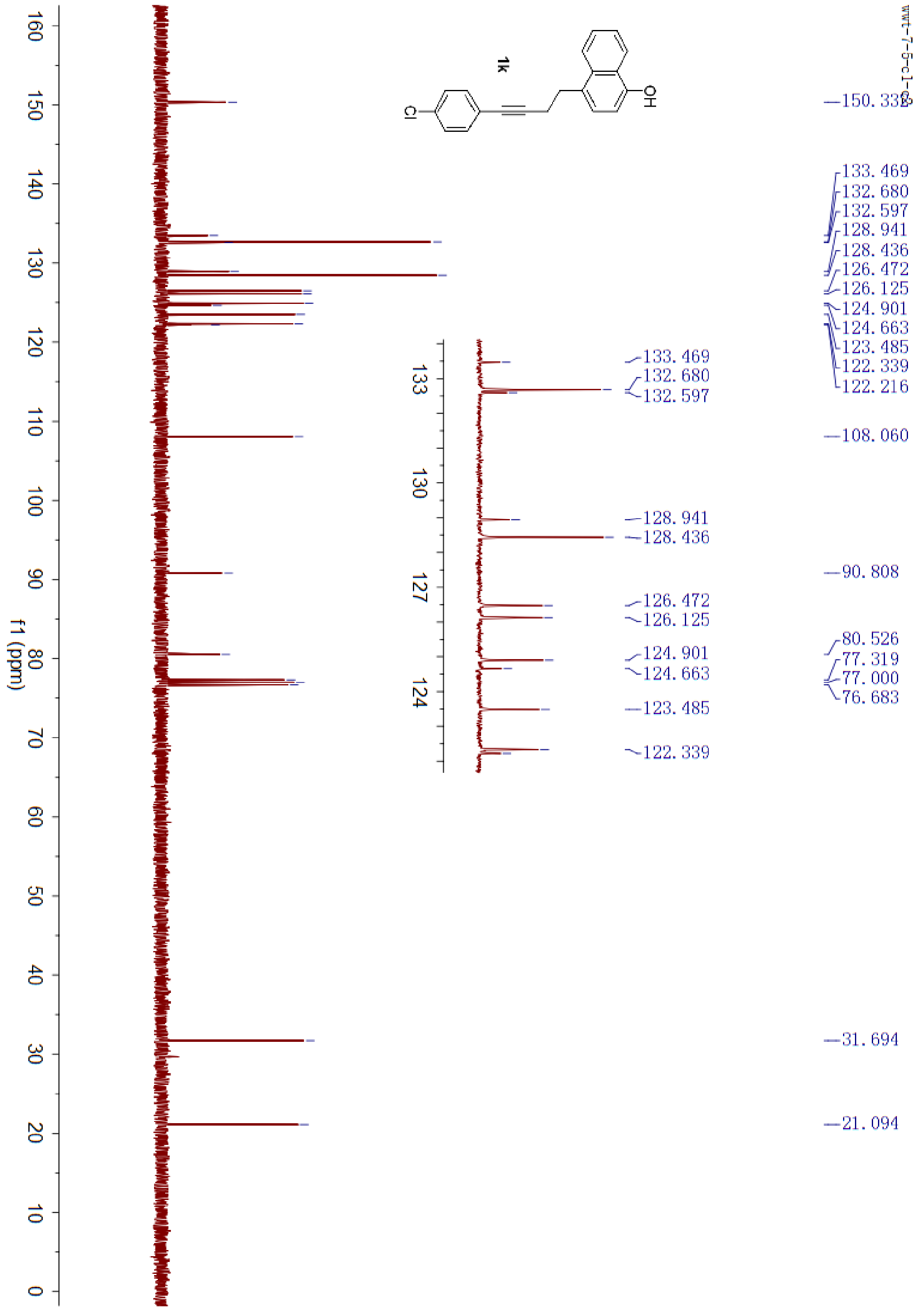

wwt-7-5-cl-c
1k
OH
Cl
150.332
133.469
132.680
132.597
128.941
128.436
126.472
126.125
124.901
124.663
123.485
122.339
122.216
108.060
90.808
80.526
77.319
77.000
76.683
31.694
21.094
f1 (ppm)

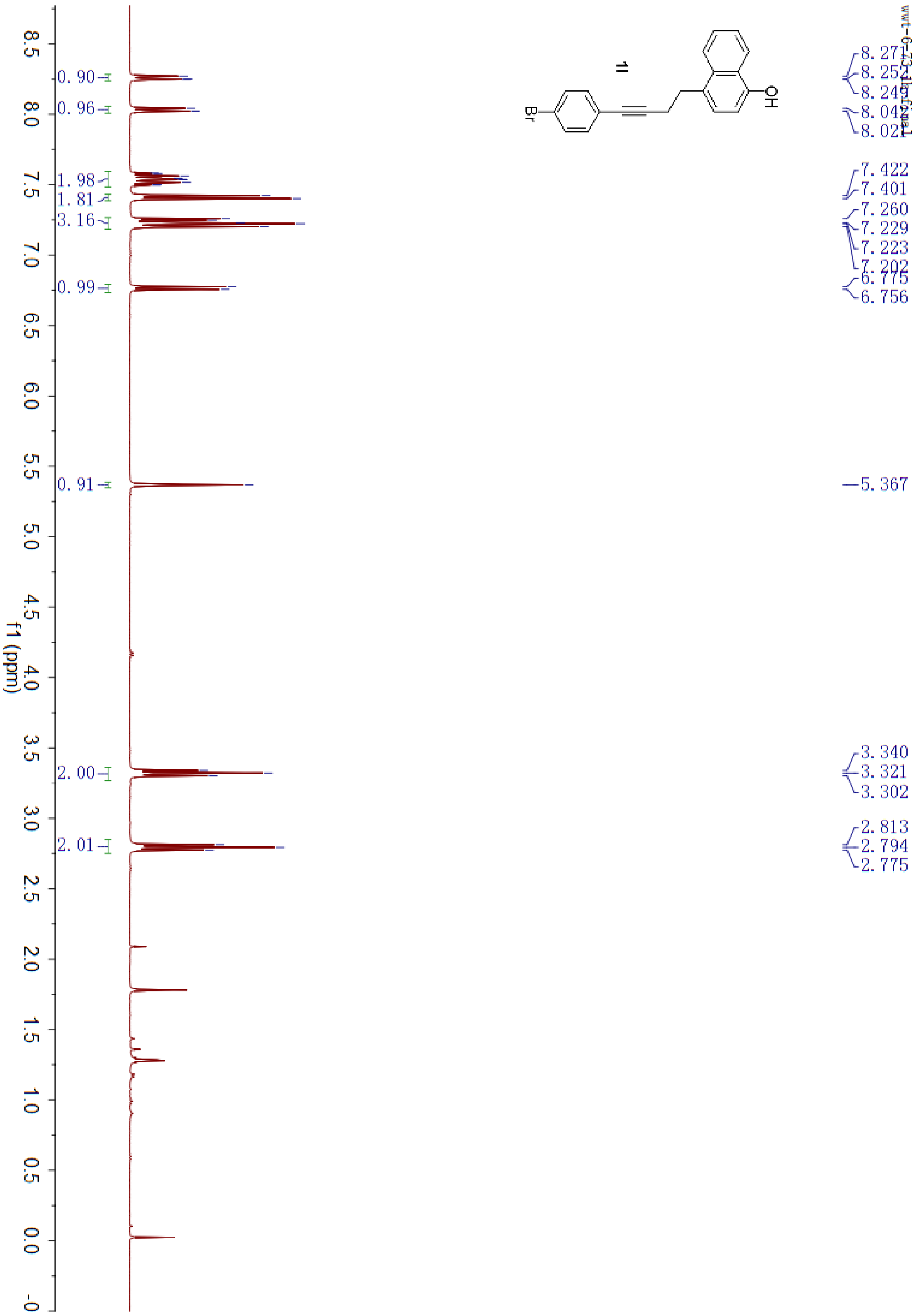
1l
OH
Br
8.271
8.252
8.249
8.042
8.021
7.422
7.401
7.260
7.229
7.223
7.202
6.775
6.756
5.367
3.340
3.321
3.302
2.813
2.794
2.775
0.90
0.96
1.98
1.81
3.16
0.99
0.91
2.00
2.01
f1 (ppm)

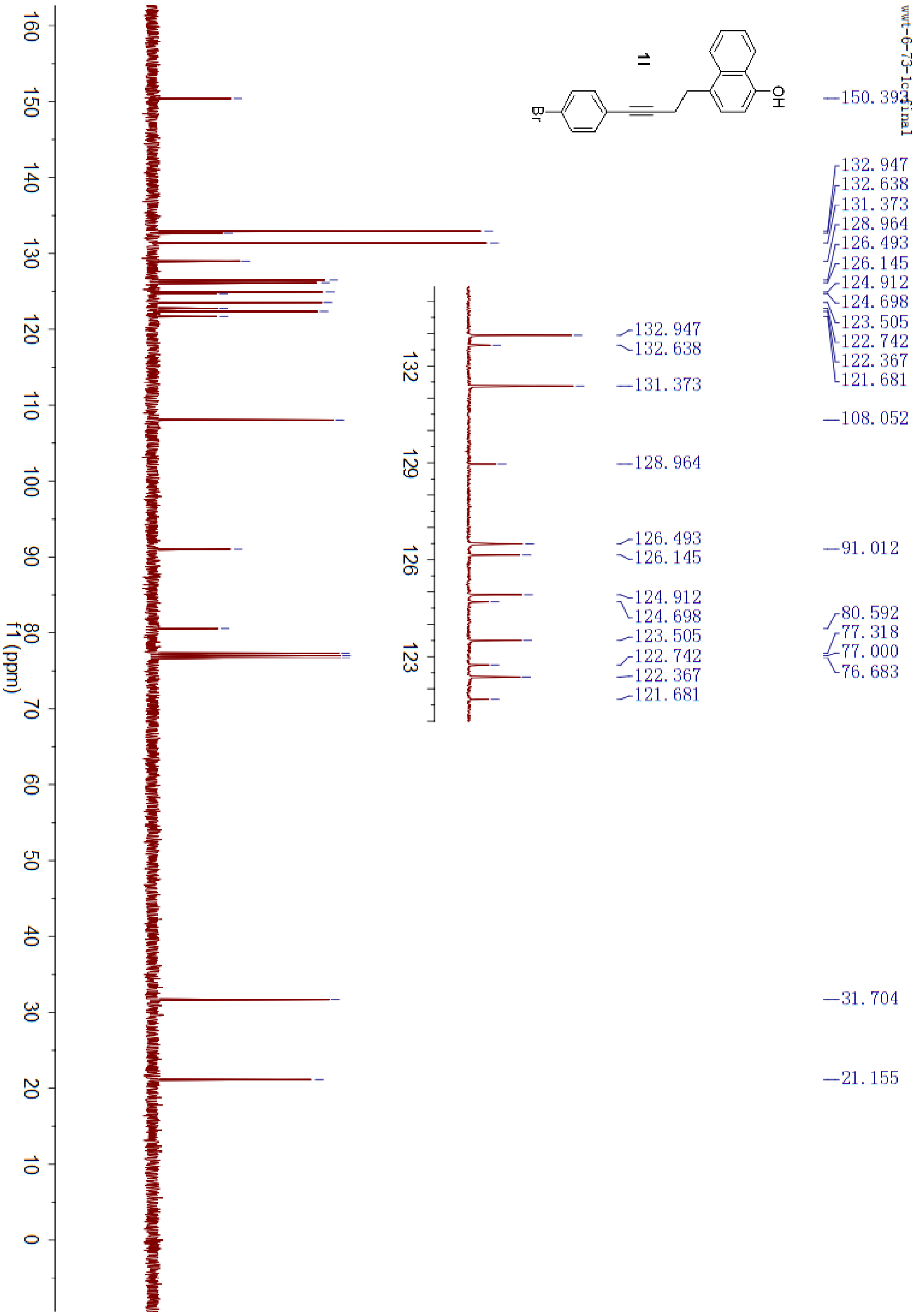

wwt-6-73-1c-final
OH
Br
11
150.393
132.947
132.638
131.373
128.964
126.493
126.145
124.912
124.698
123.505
122.742
122.367
121.681
108.052
91.012
80.592
77.318
77.000
76.683
31.704
21.155
f1 (ppm)

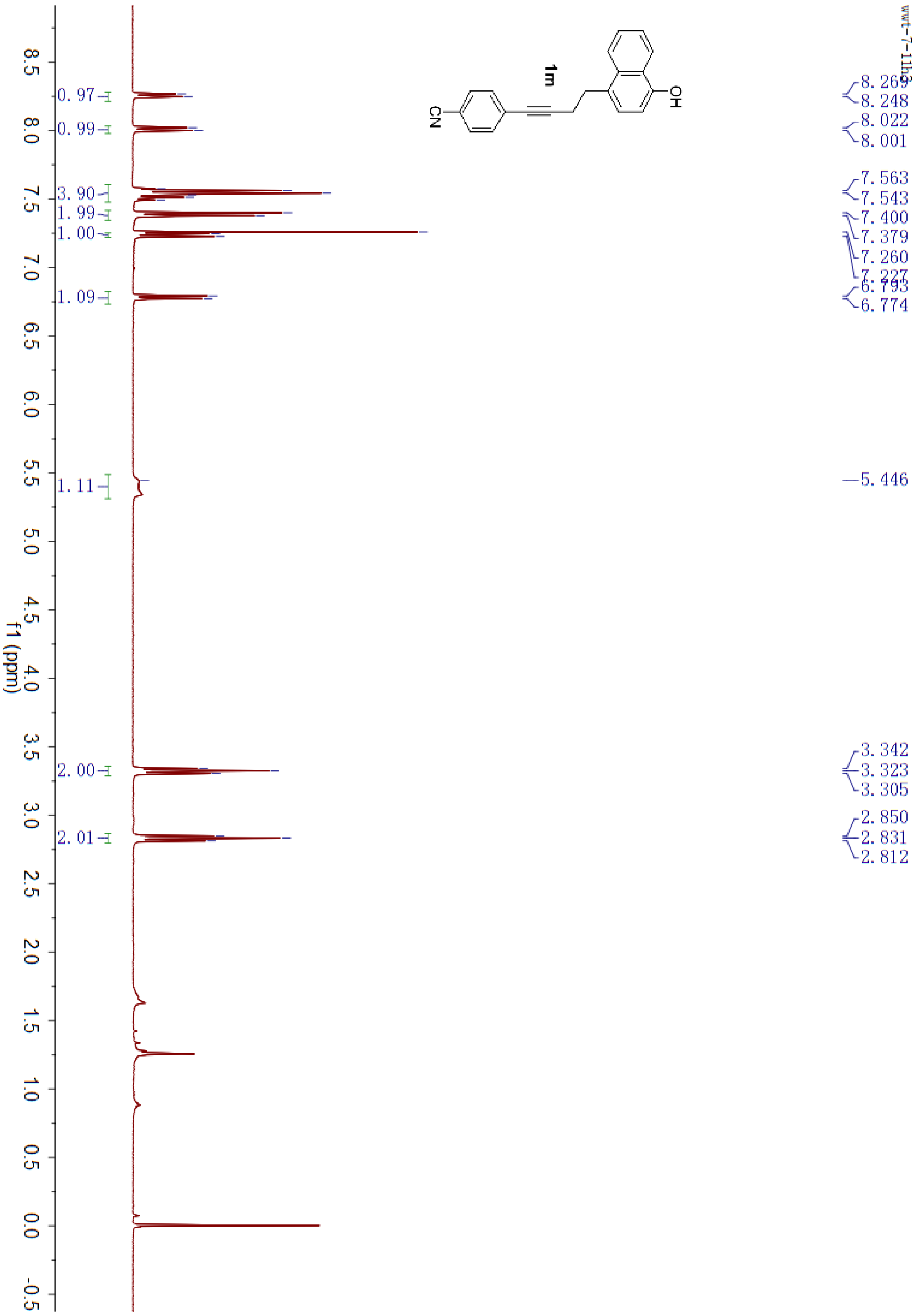
wwt-7-11h
1m
CN
OH
8.269
8.248
8.022
8.001
7.563
7.543
7.400
7.379
7.260
7.227
6.793
6.774
5.446
3.342
3.323
3.305
2.850
2.831
2.812
0.97
0.99
3.90
1.99
1.00
1.09
1.11
2.00
2.01
f1 (ppm)

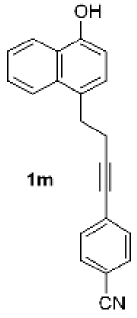
OH
CN

1m

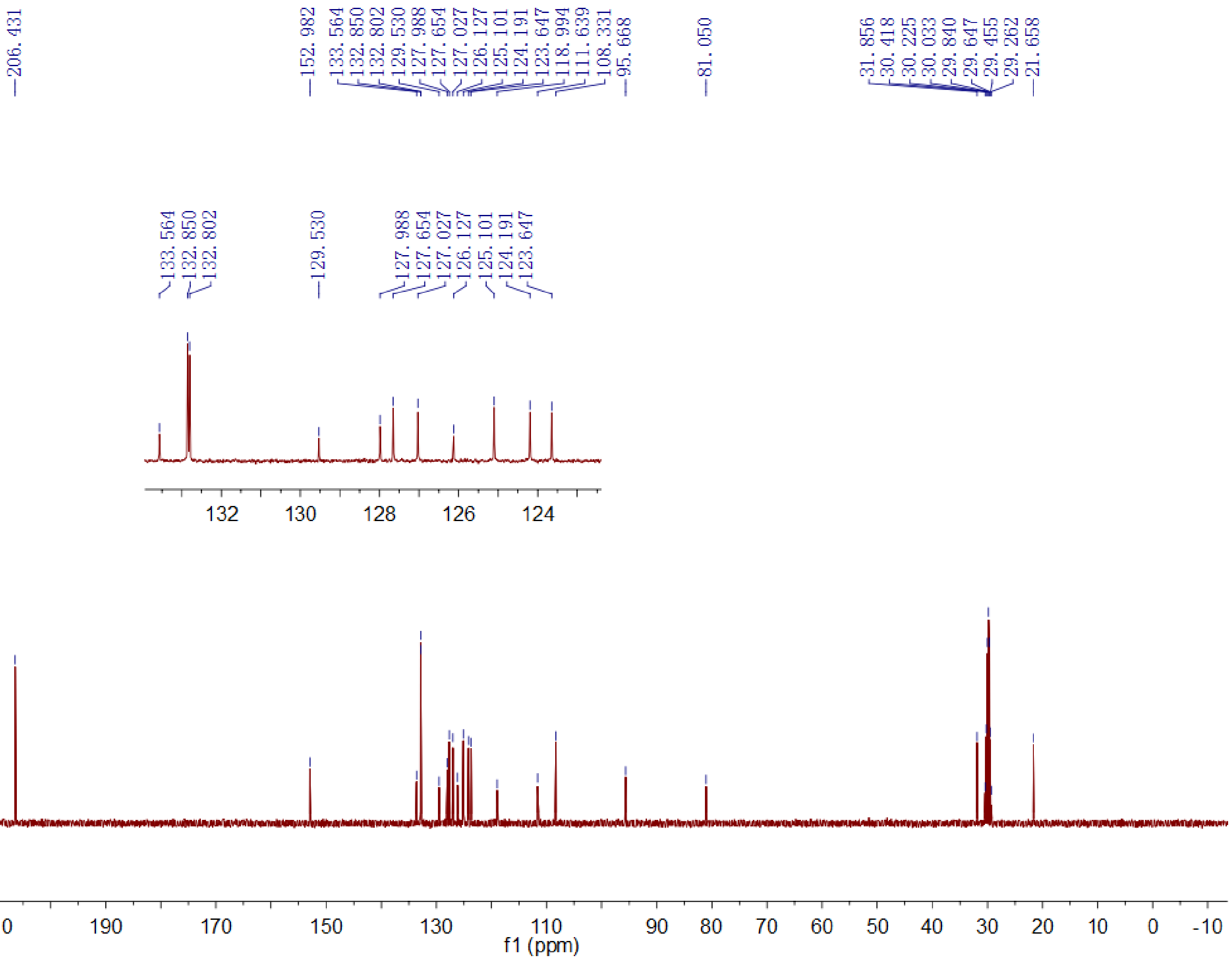
wwt-7-11c-acetone
new experiment
206.431
152.982
133.564
132.850
132.802
129.530
127.988
127.654
127.027
126.127
125.101
124.191
123.647
118.994
111.639
108.331
95.668
81.050
31.856
30.418
30.225
30.033
29.840
29.647
29.455
29.262
21.658
f1 (ppm)

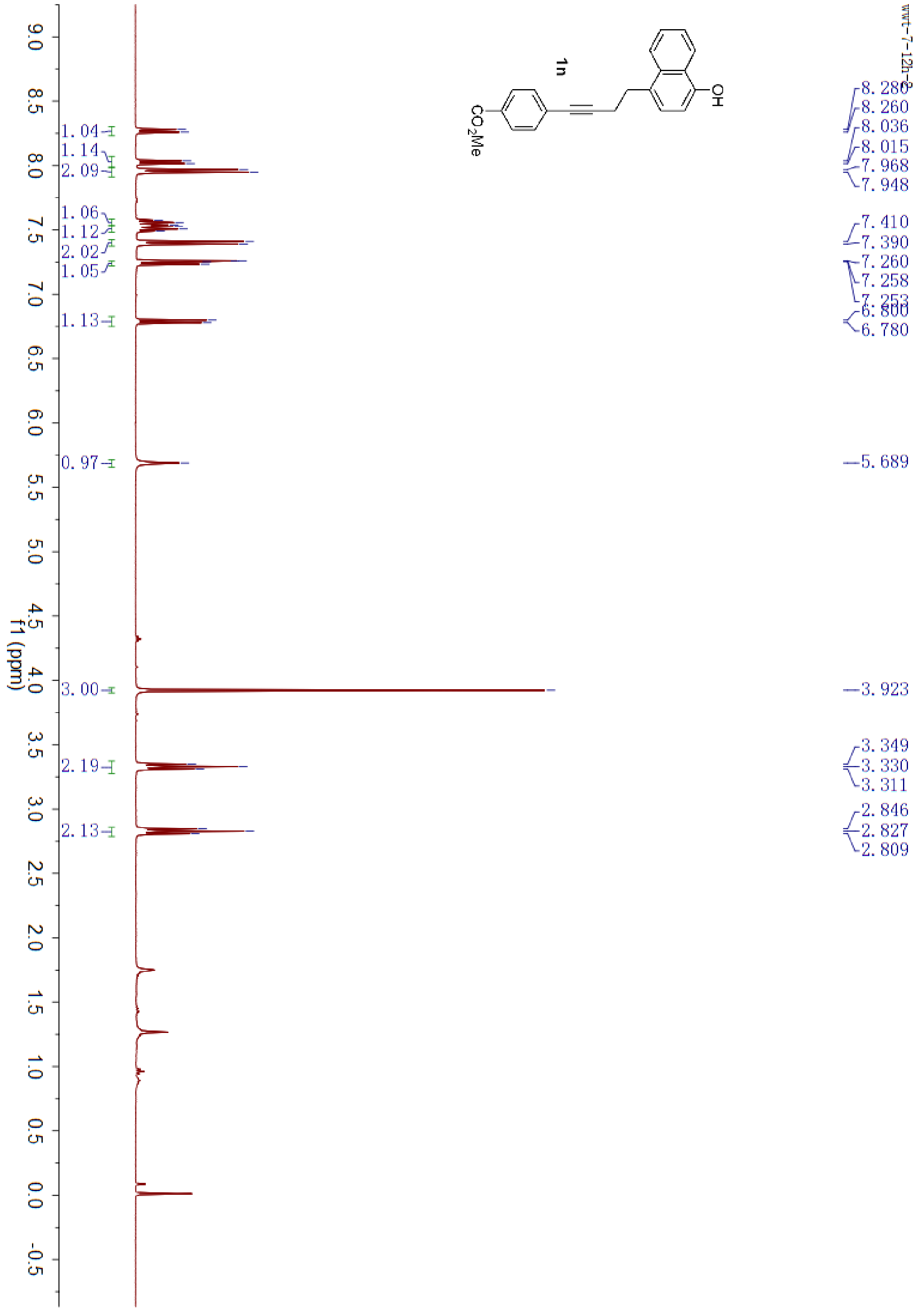
wwt-7-12h-2
1n
OH
CO2Me
8.280
8.260
8.036
8.015
7.968
7.948
7.410
7.390
7.260
7.258
7.253
6.800
6.780
5.689
3.923
3.349
3.330
3.311
2.846
2.827
2.809
1.04
1.14
2.09
1.06
1.12
2.02
1.05
1.13
0.97
3.00
2.19
2.13
f1 (ppm)

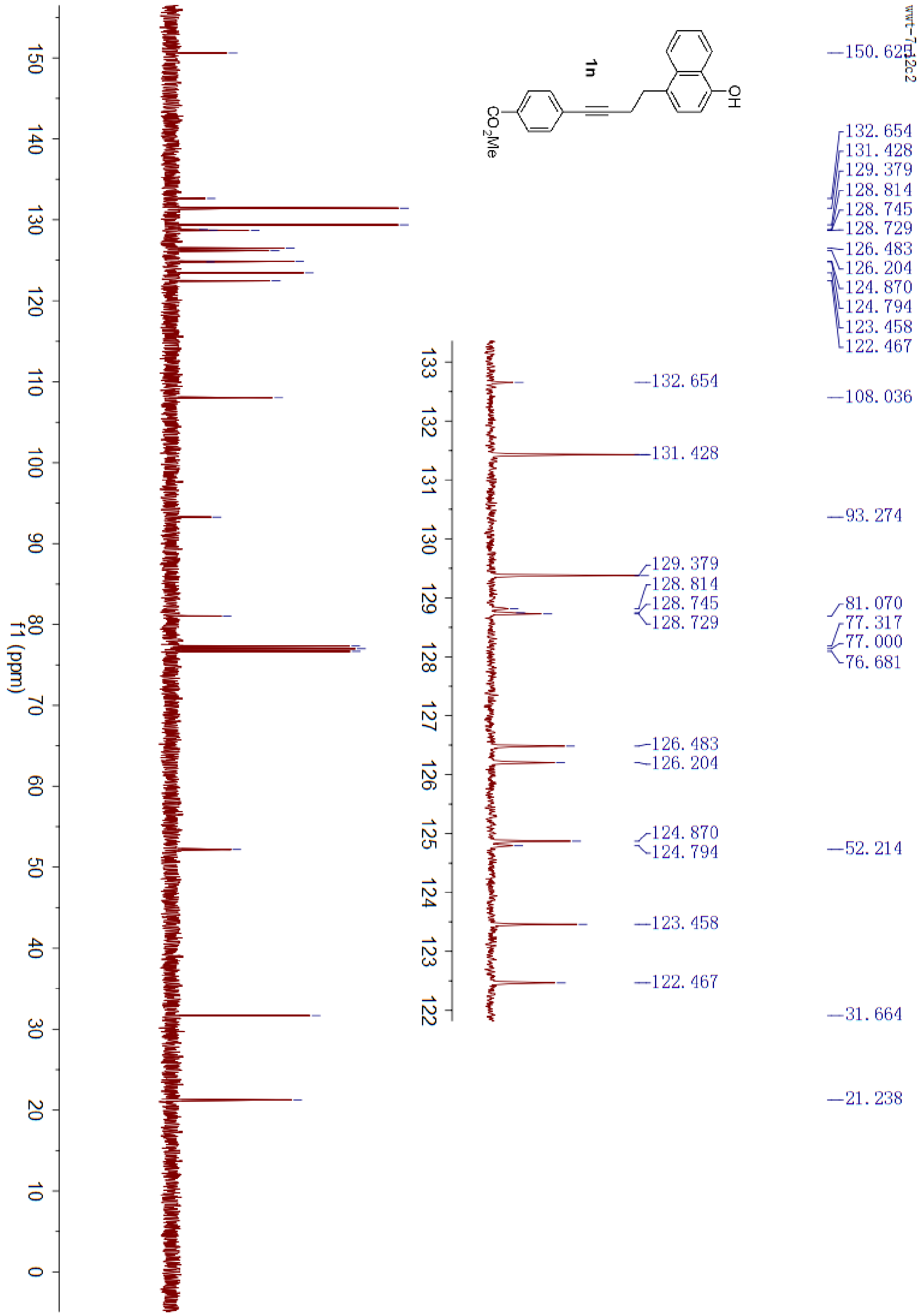
wwt-7-1n2c2
1n
OH
CO2Me
150.621
132.654
131.428
129.379
128.814
128.745
128.729
126.483
126.204
124.870
124.794
123.458
122.467
108.036
93.274
81.070
77.317
77.000
76.681
52.214
31.664
21.238
f1 (ppm)

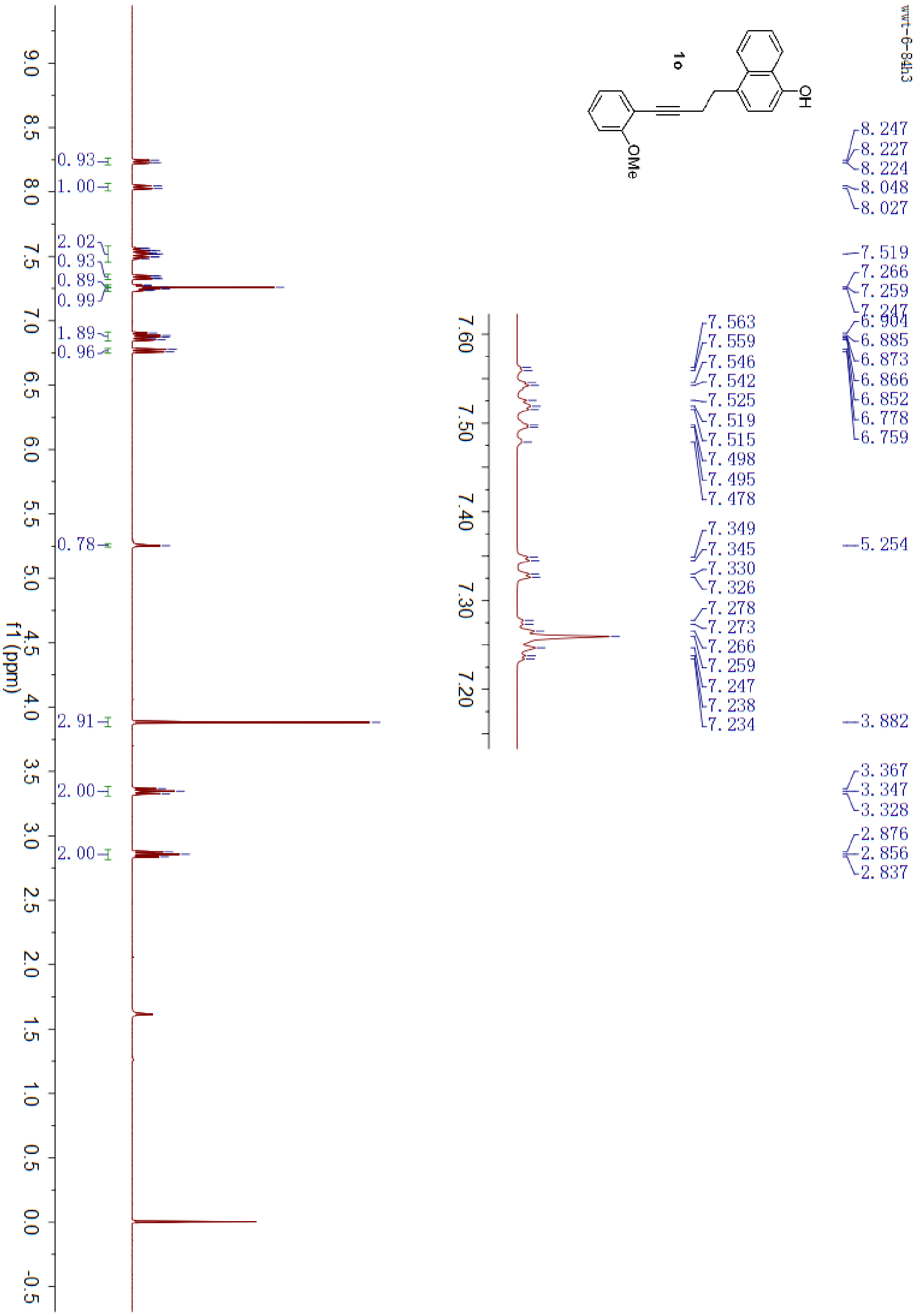
wwt-6-84h3
1o
OH
OMe
8.247
8.227
8.224
8.048
8.027
7.519
7.266
7.259
7.247
6.904
6.885
6.873
6.866
6.852
6.778
6.759
5.254
3.882
3.367
3.347
3.328
2.876
2.856
2.837
7.563
7.559
7.546
7.542
7.525
7.519
7.515
7.498
7.495
7.478
7.349
7.345
7.330
7.326
7.278
7.273
7.266
7.259
7.247
7.238
7.234
0.93
1.00
2.02
0.93
0.89
0.99
1.89
0.96
0.78
2.91
2.00
2.00
f1 (ppm)

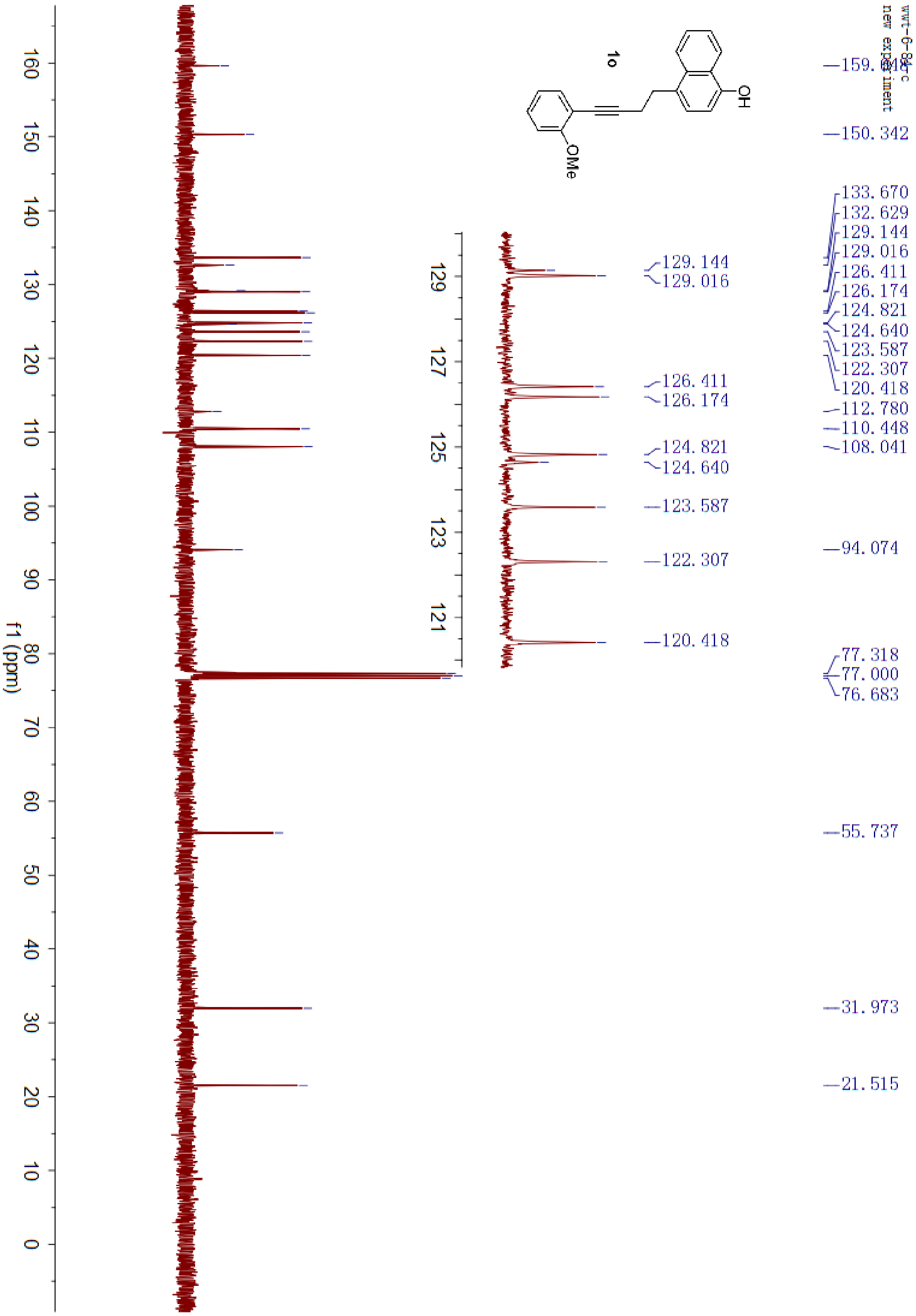
wwt-6-84-c
new experiment
1o
OH
OMe
159.841
150.342
133.670
132.629
129.144
129.016
126.411
126.174
124.821
124.640
123.587
122.307
120.418
112.780
110.448
108.041
94.074
77.318
77.000
76.683
55.737
31.973
21.515
f1 (ppm)

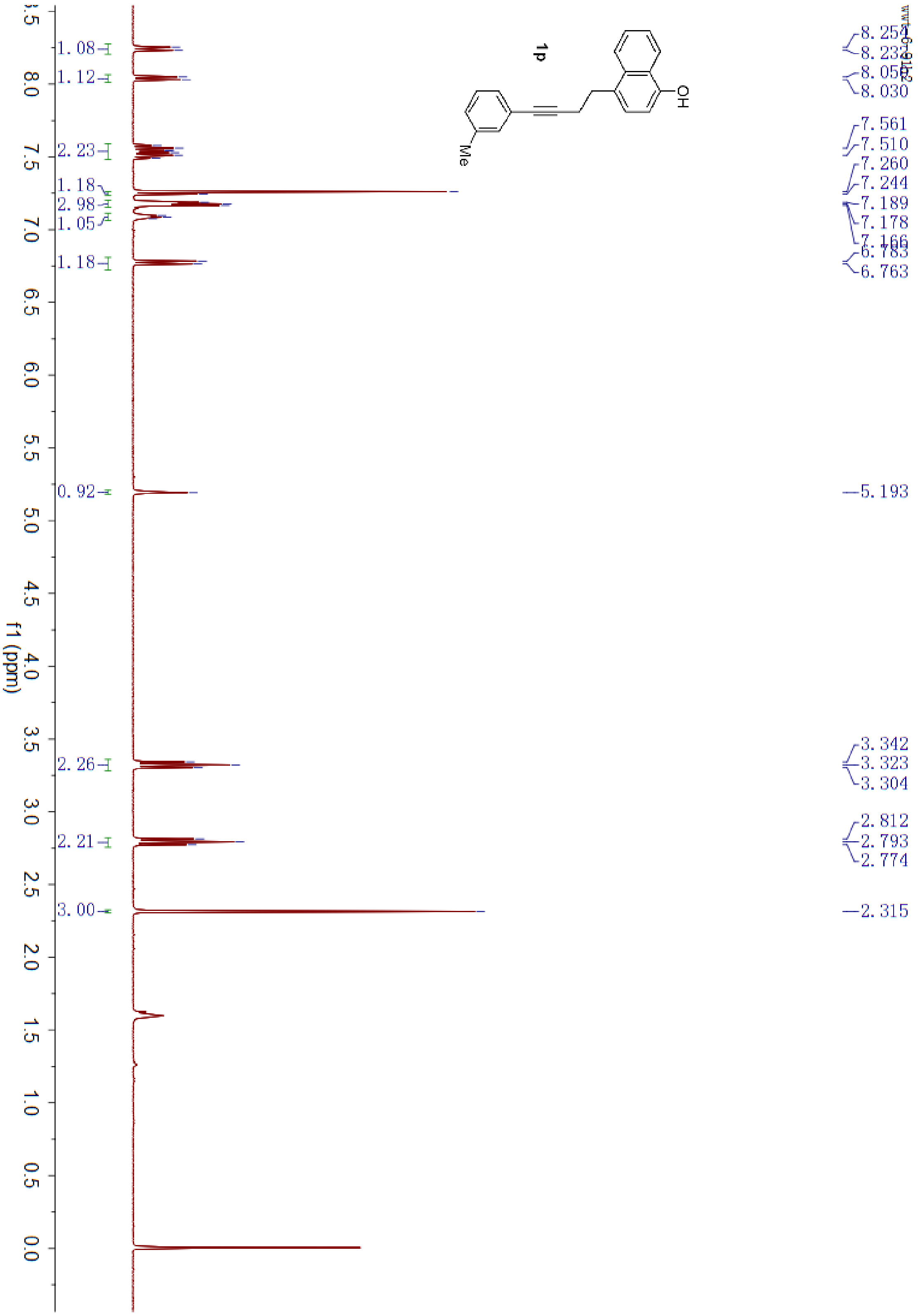
1p
OH
Me
8.251
8.231
8.050
8.030
7.561
7.510
7.260
7.244
7.189
7.178
7.166
6.783
6.763
5.193
3.342
3.323
3.304
2.812
2.793
2.774
2.315
f1 (ppm)

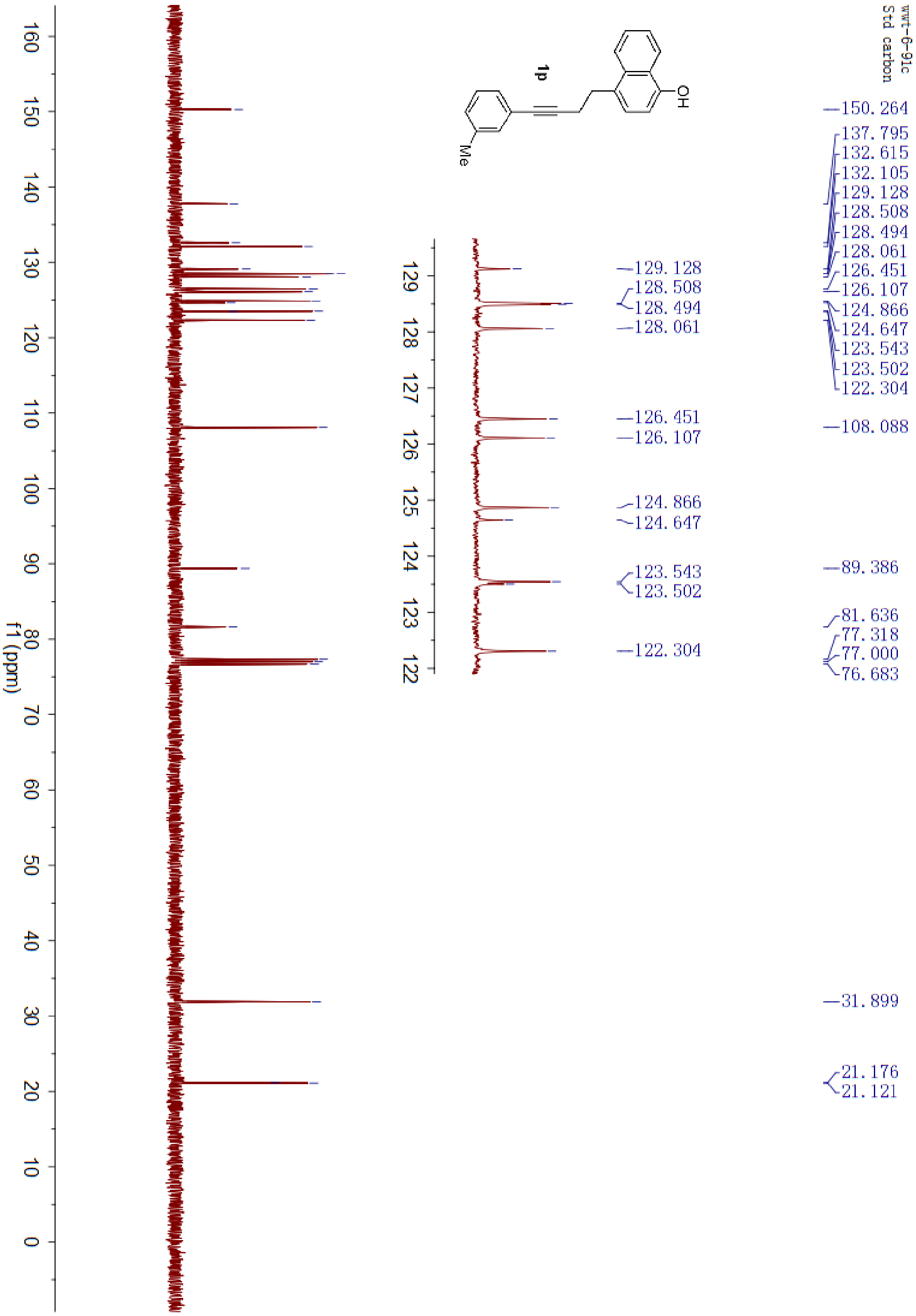
wwt-6-91c
Std carbon
1p
OH
Me
150.264
137.795
132.615
132.105
129.128
128.508
128.494
128.061
126.451
126.107
124.866
124.647
123.543
123.502
122.304
108.088
89.386
81.636
77.318
77.000
76.683
31.899
21.176
21.121
f1 (ppm)

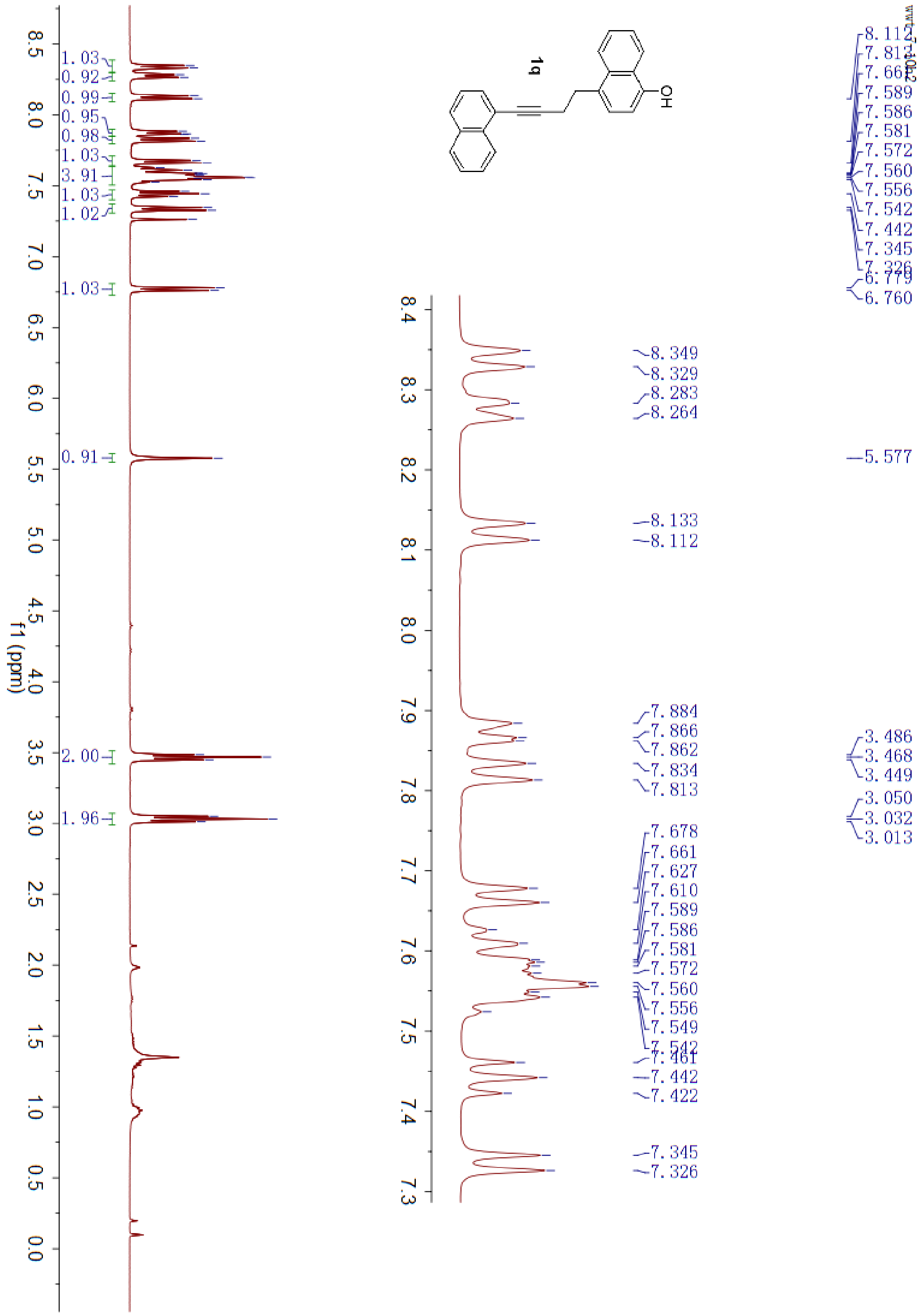
1q
OH
f1 (ppm)
8.349
8.329
8.283
8.264
8.133
8.112
7.884
7.866
7.862
7.834
7.813
7.678
7.661
7.627
7.610
7.589
7.586
7.581
7.572
7.560
7.556
7.549
7.442
7.422
7.345
7.326
6.760
5.577
3.486
3.468
3.449
3.050
3.032
3.013
1.03
0.92
0.99
0.95
0.98
1.03
3.91
1.03
1.02
1.03
0.91
2.00
1.96

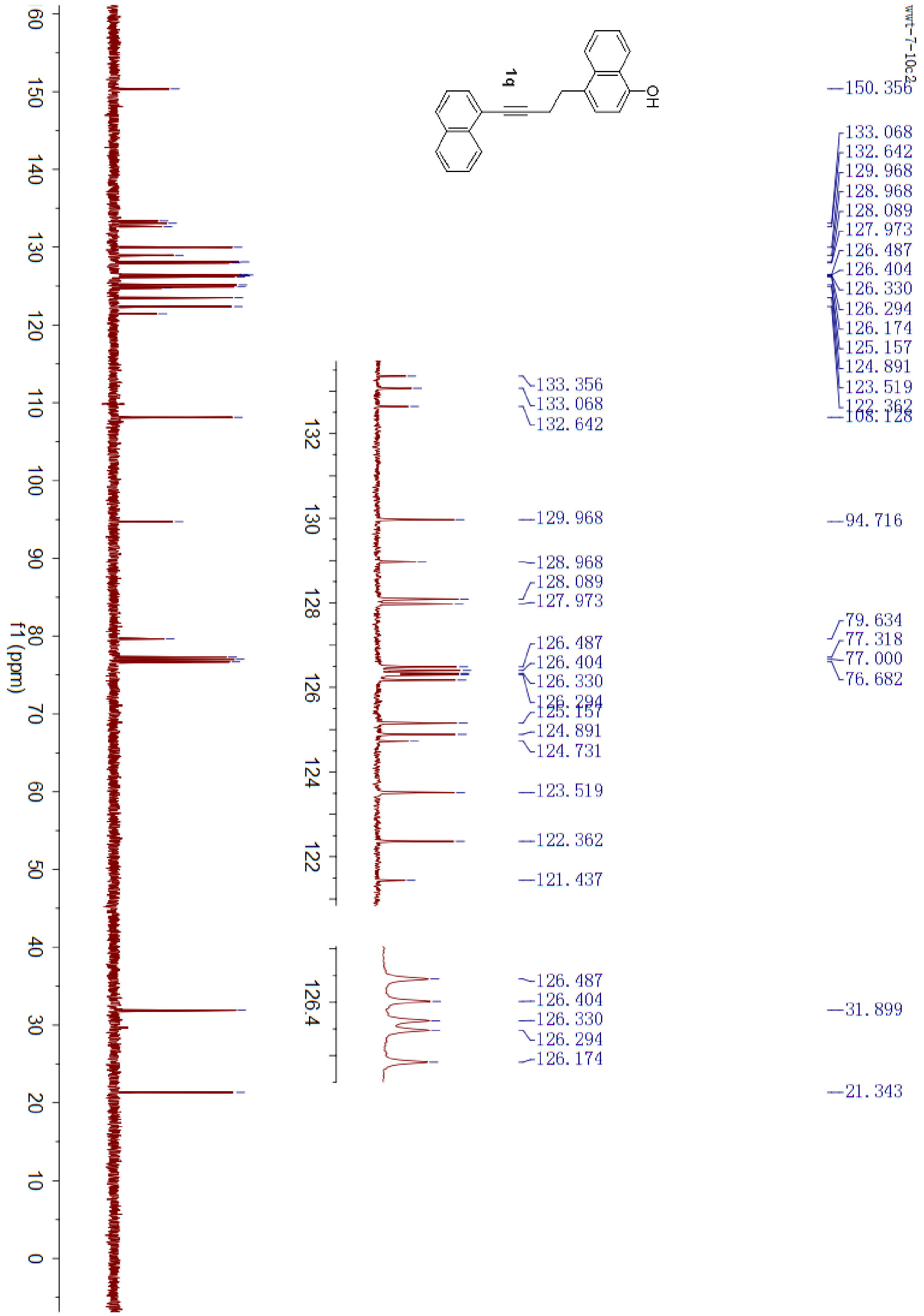

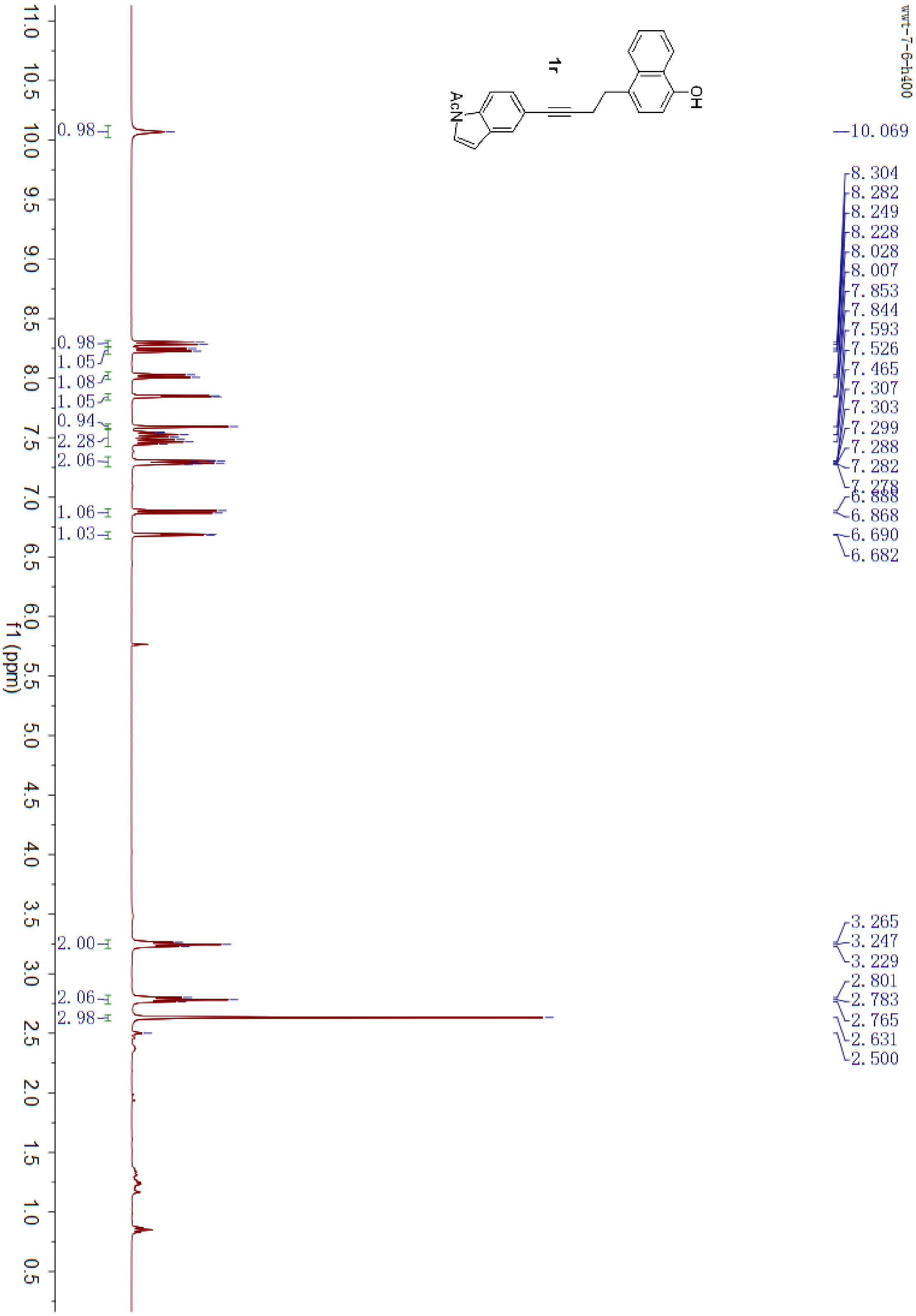
wwt-7-6-h400
1r
AcN
OH
10.069
8.304
8.282
8.249
8.228
8.028
8.007
7.853
7.844
7.593
7.526
7.465
7.307
7.303
7.299
7.288
7.282
7.278
6.888
6.868
6.690
6.682
3.265
3.247
3.229
2.801
2.783
2.765
2.631
2.500
0.98
0.98
1.05
1.08
1.05
0.94
2.28
2.06
1.06
1.03
2.00
2.06
2.98
f1 (ppm)

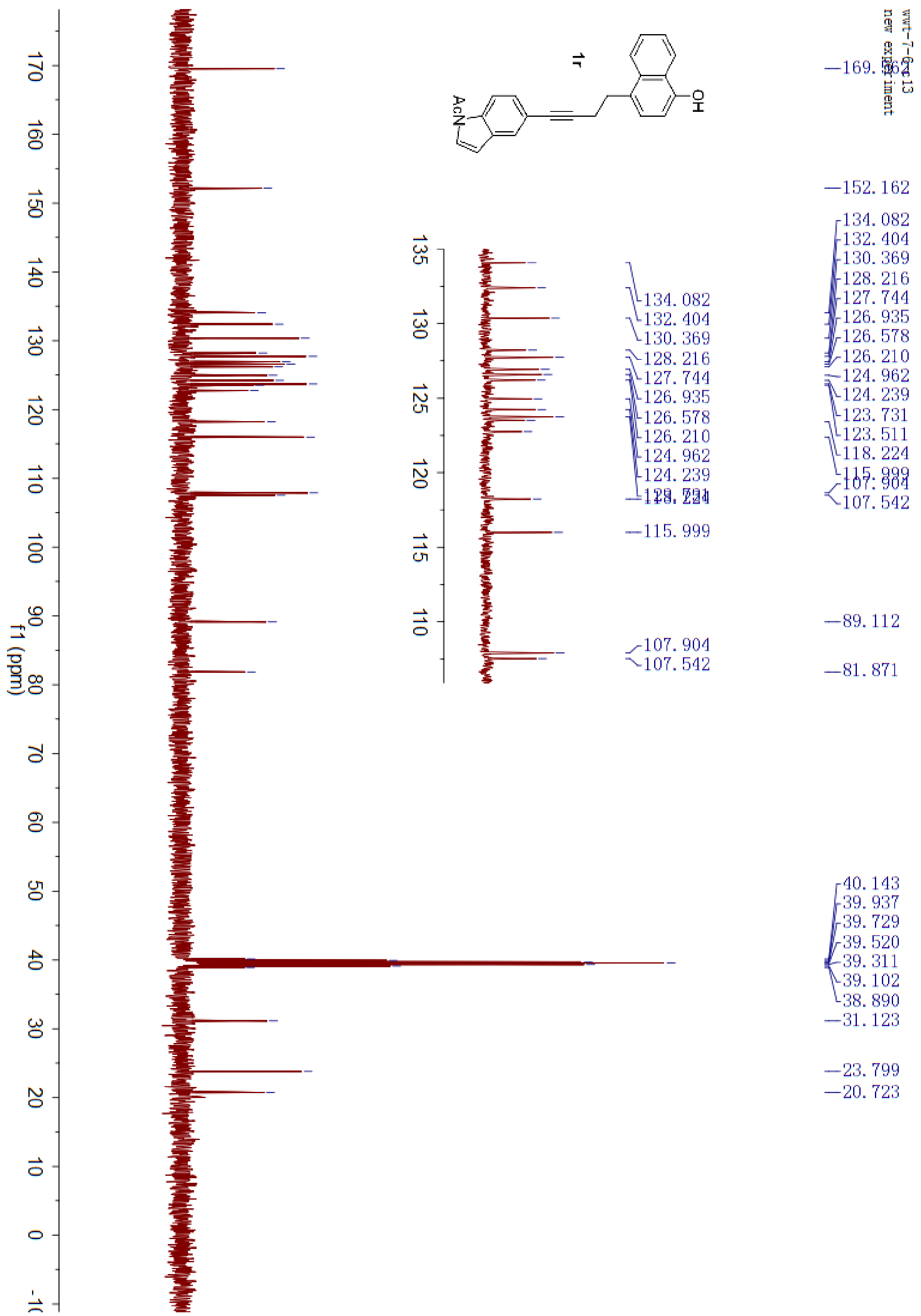
wwt-7-6.c13
new experiment
1r
OH
AcN
169.
152.162
134.082
132.404
130.369
128.216
127.744
126.935
126.578
126.210
124.962
124.239
123.731
123.511
118.224
107.542
89.112
81.871
40.143
39.937
39.729
39.520
39.311
39.102
38.890
31.123
23.799
20.723
115.999
107.904
f1 (ppm)

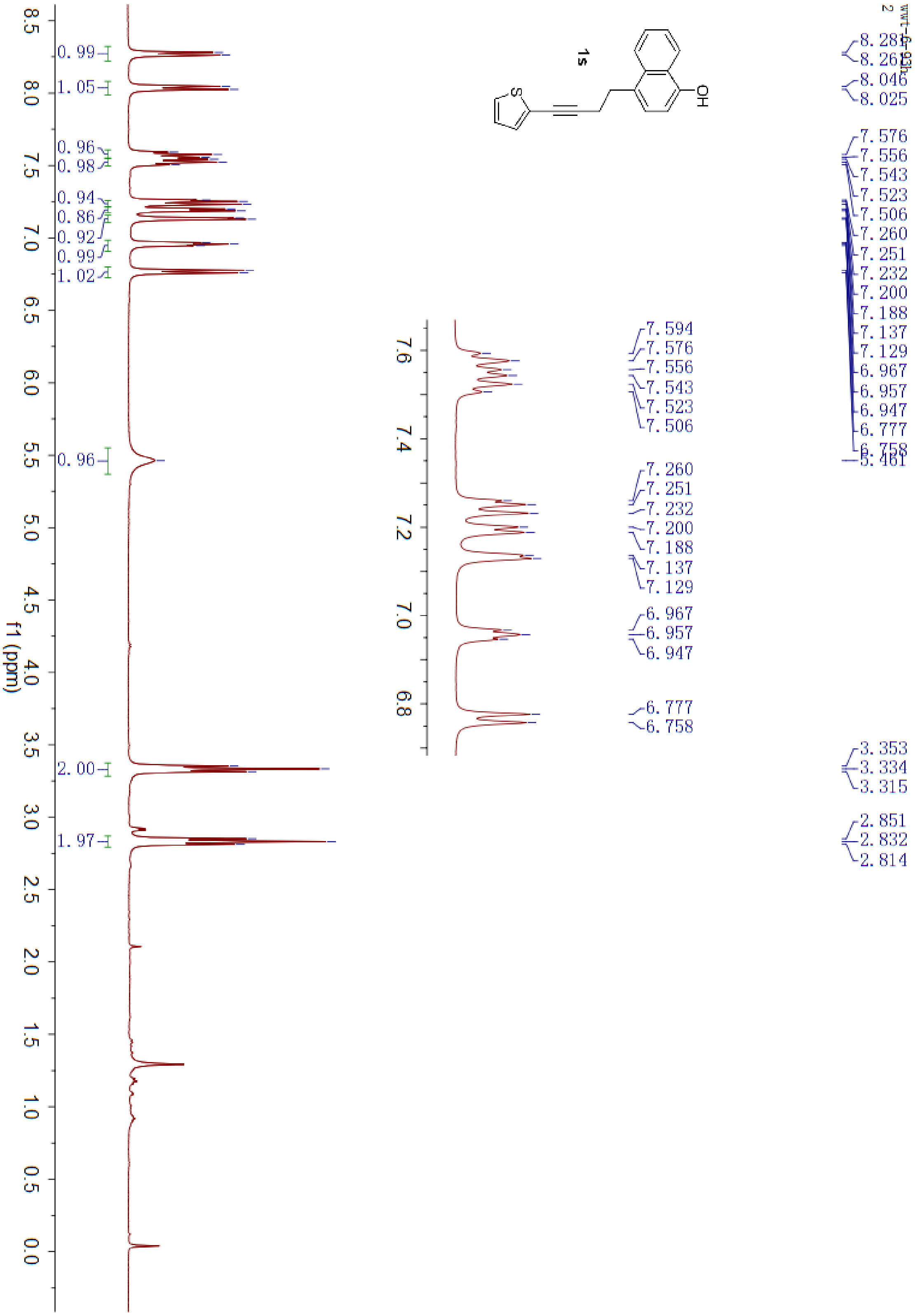

1s
OH
S
8.261
8.046
8.025
7.576
7.556
7.543
7.523
7.506
7.260
7.251
7.232
7.200
7.188
7.137
7.129
6.967
6.957
6.947
6.777
5.461
3.353
3.334
3.315
2.851
2.832
2.814
7.594
7.576
7.556
7.543
7.523
7.506
7.260
7.251
7.232
7.200
7.188
7.137
7.129
6.967
6.957
6.947
6.777
6.758
0.99
1.05
0.96
0.98
0.94
0.86
0.92
0.99
1.02
0.96
2.00
1.97
f1 (ppm)

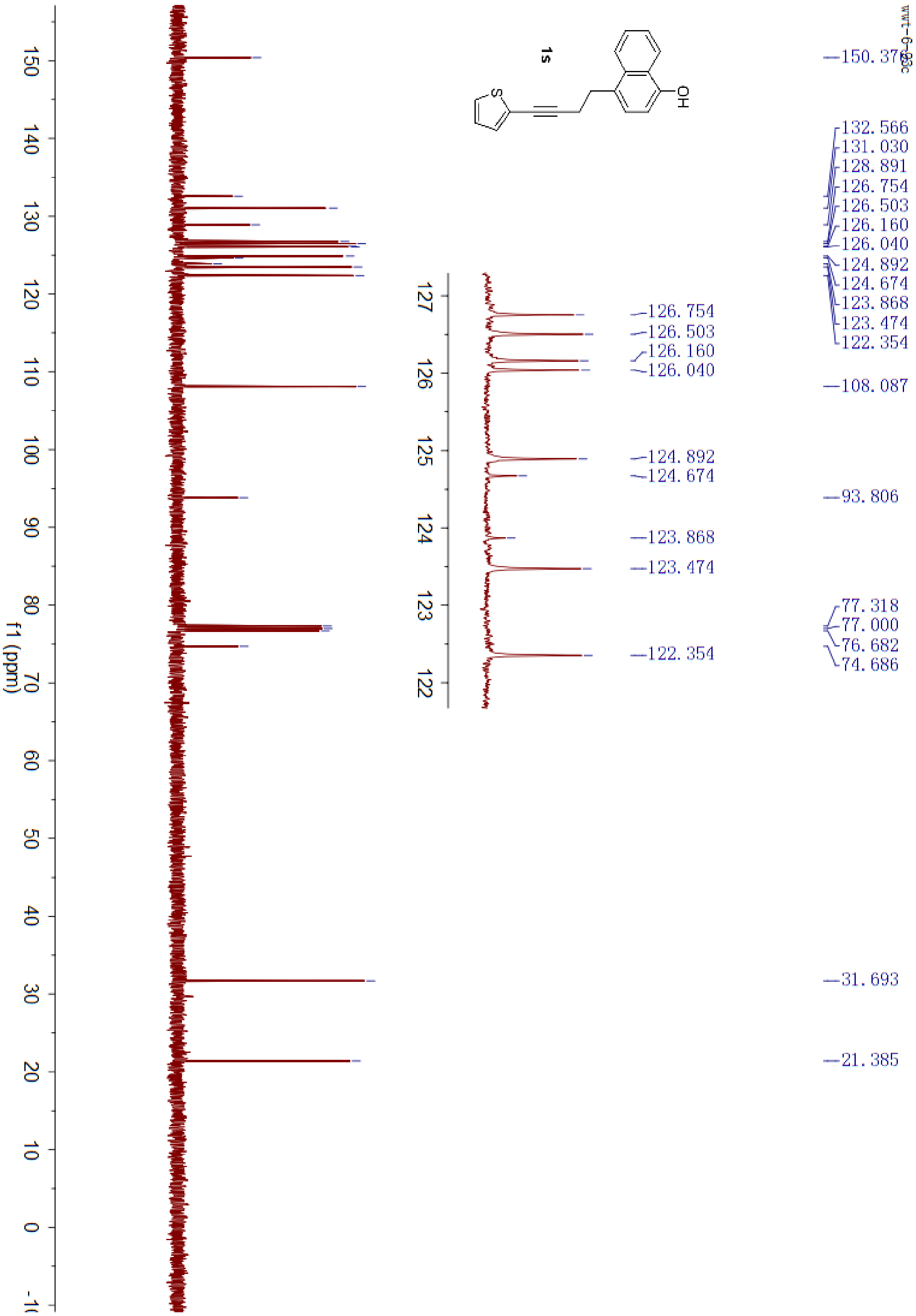
ww1-6-13c
1s
OH
S
150.378
132.566
131.030
128.891
126.754
126.503
126.160
126.040
124.892
124.674
123.868
123.474
122.354
108.087
93.806
77.318
77.000
76.682
74.686
31.693
21.385
f1 (ppm)

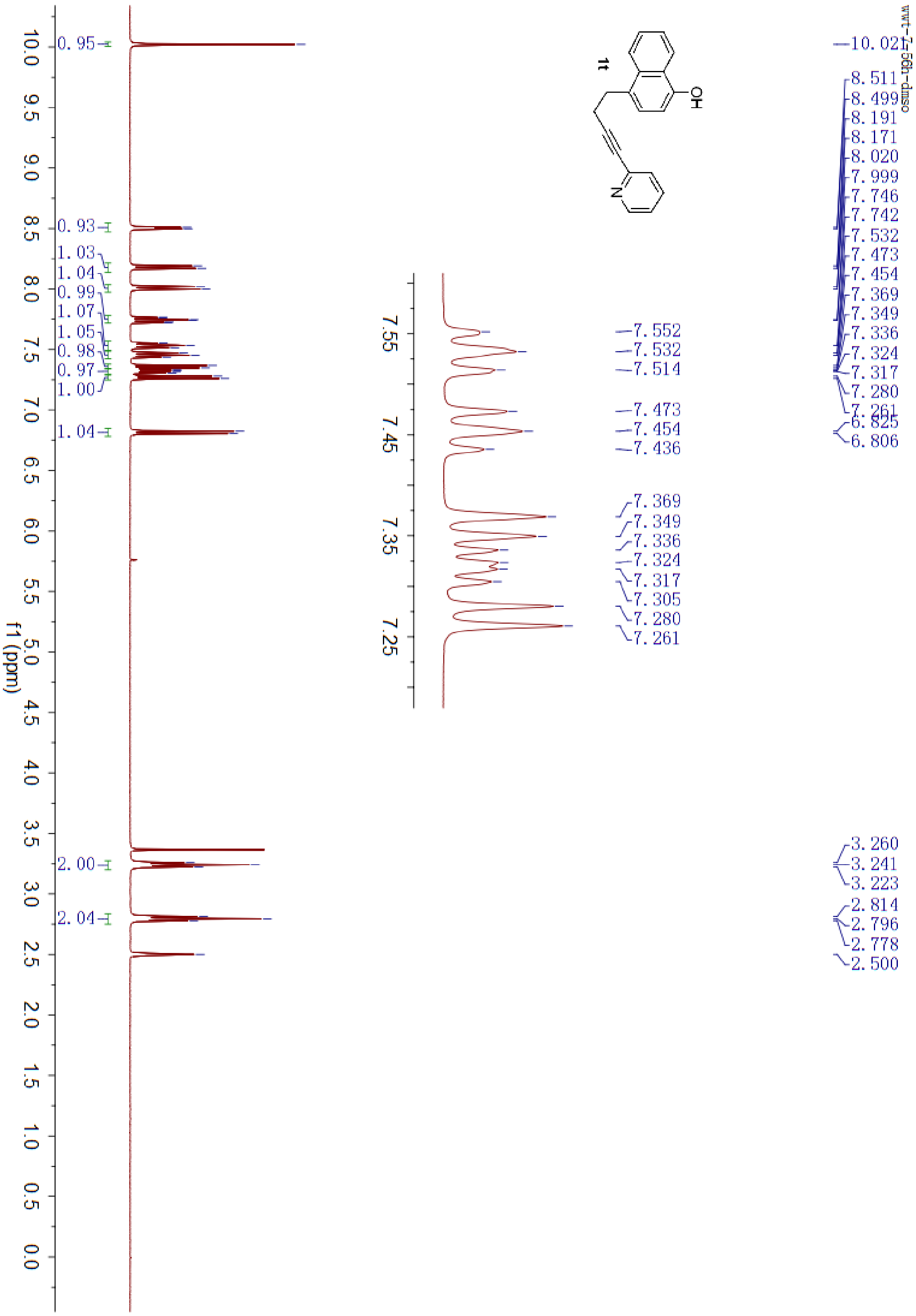

1t
OH
N
10.021
8.511
8.499
8.191
8.171
8.020
7.999
7.746
7.742
7.532
7.473
7.454
7.369
7.349
7.336
7.324
7.317
7.280
7.261
6.825
6.806
3.260
3.241
3.223
2.814
2.796
2.778
2.500
7.552
7.532
7.514
7.473
7.454
7.436
7.369
7.349
7.336
7.324
7.317
7.305
7.280
7.261
0.95
0.93
1.03
1.04
0.99
1.07
1.05
0.98
0.97
1.00
1.04
2.00
2.04
f1 (ppm)

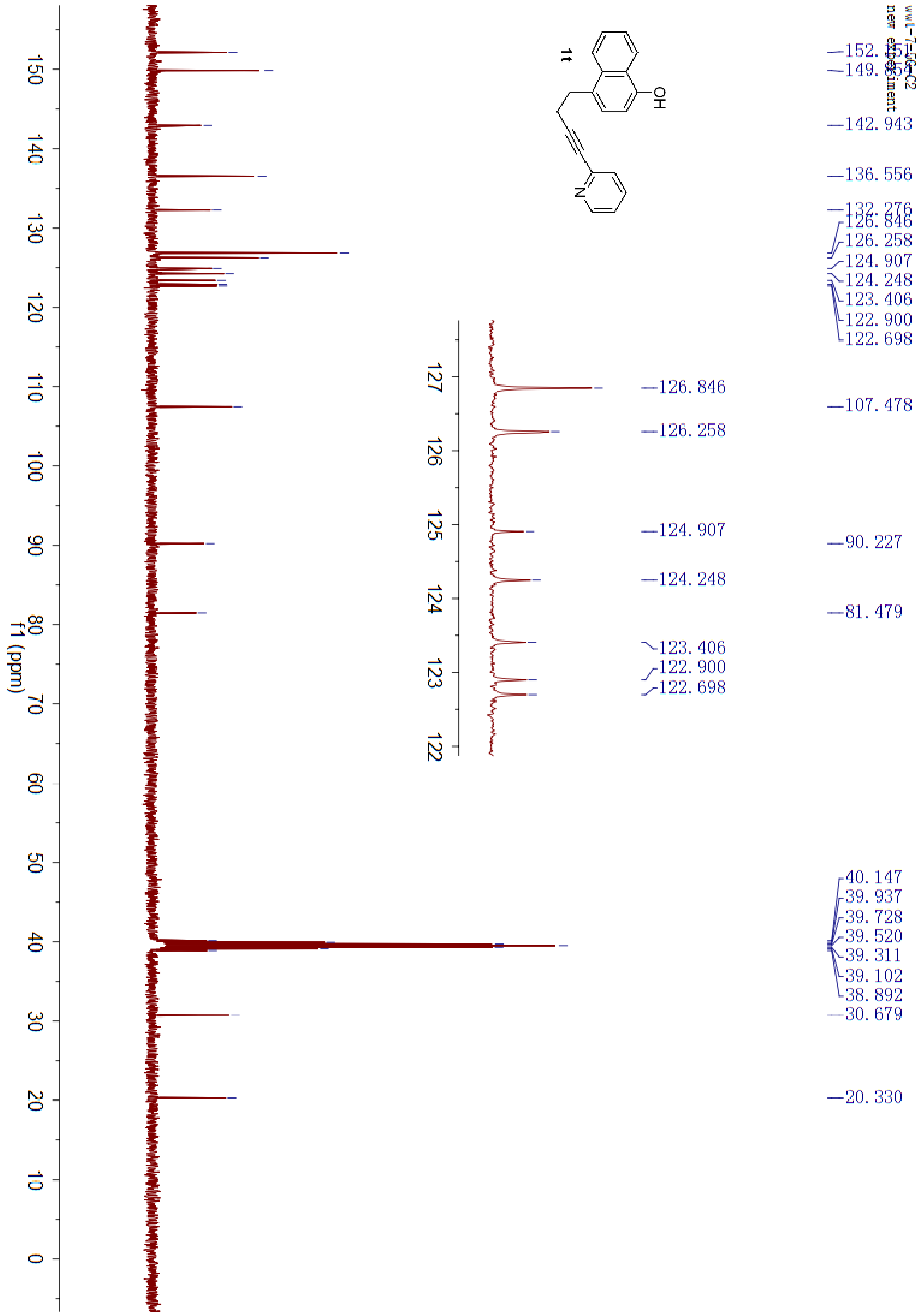
wwt-7-56-C2
new experiment
142.943
136.556
132.276
126.846
126.258
124.907
124.248
123.406
122.900
122.698
107.478
90.227
81.479
40.147
39.937
39.728
39.520
39.311
39.102
38.892
30.679
20.330
OH
N
1t
f1 (ppm)

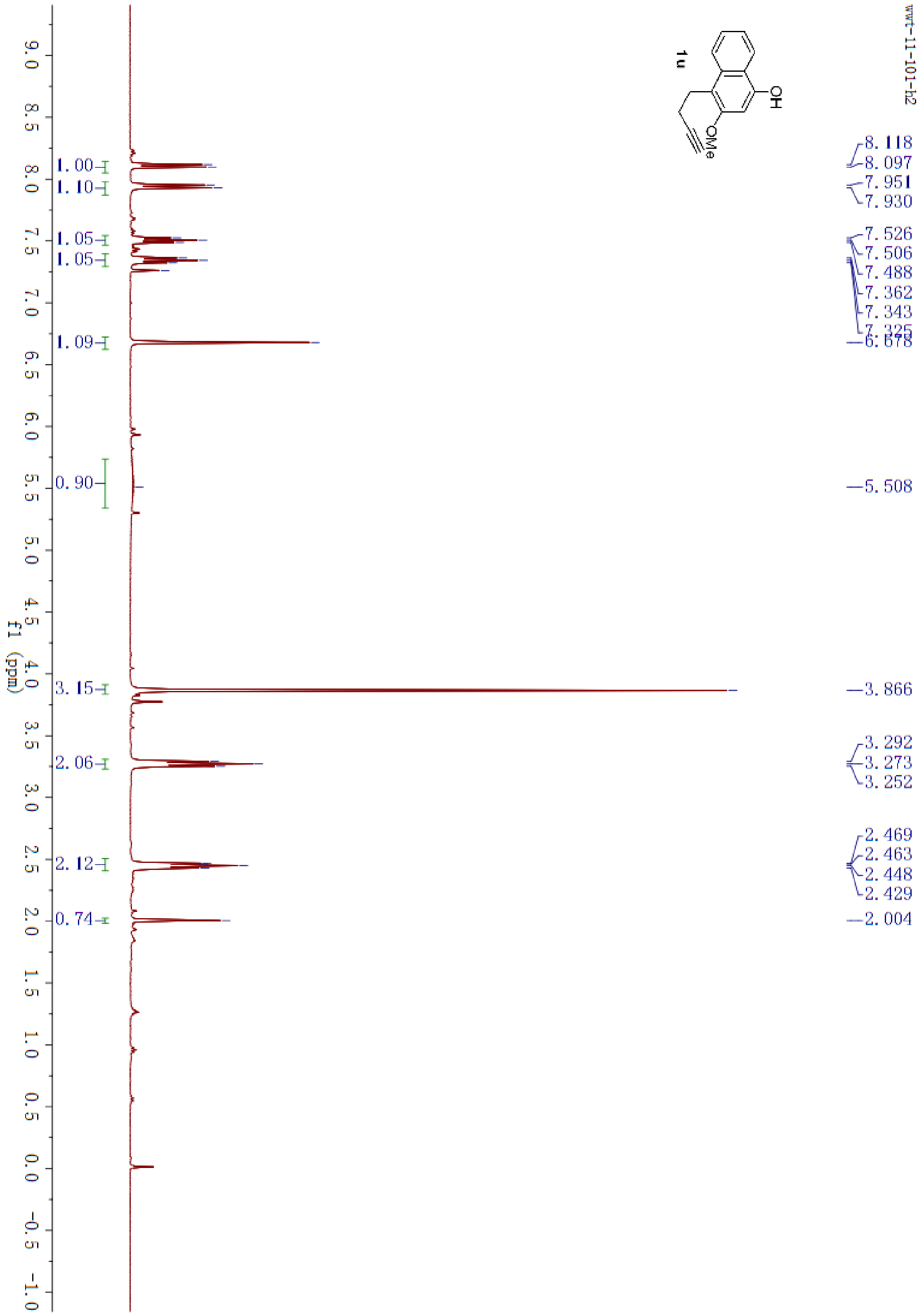
wwt-11-101-h2
1u
OMe
OH
8.118
8.097
7.951
7.930
7.526
7.506
7.488
7.362
7.343
7.325
6.678
5.508
3.866
3.292
3.273
3.252
2.469
2.463
2.448
2.429
2.004
1.00
1.10
1.05
1.05
1.09
0.90
3.15
2.06
2.12
0.74
f1 (ppm)

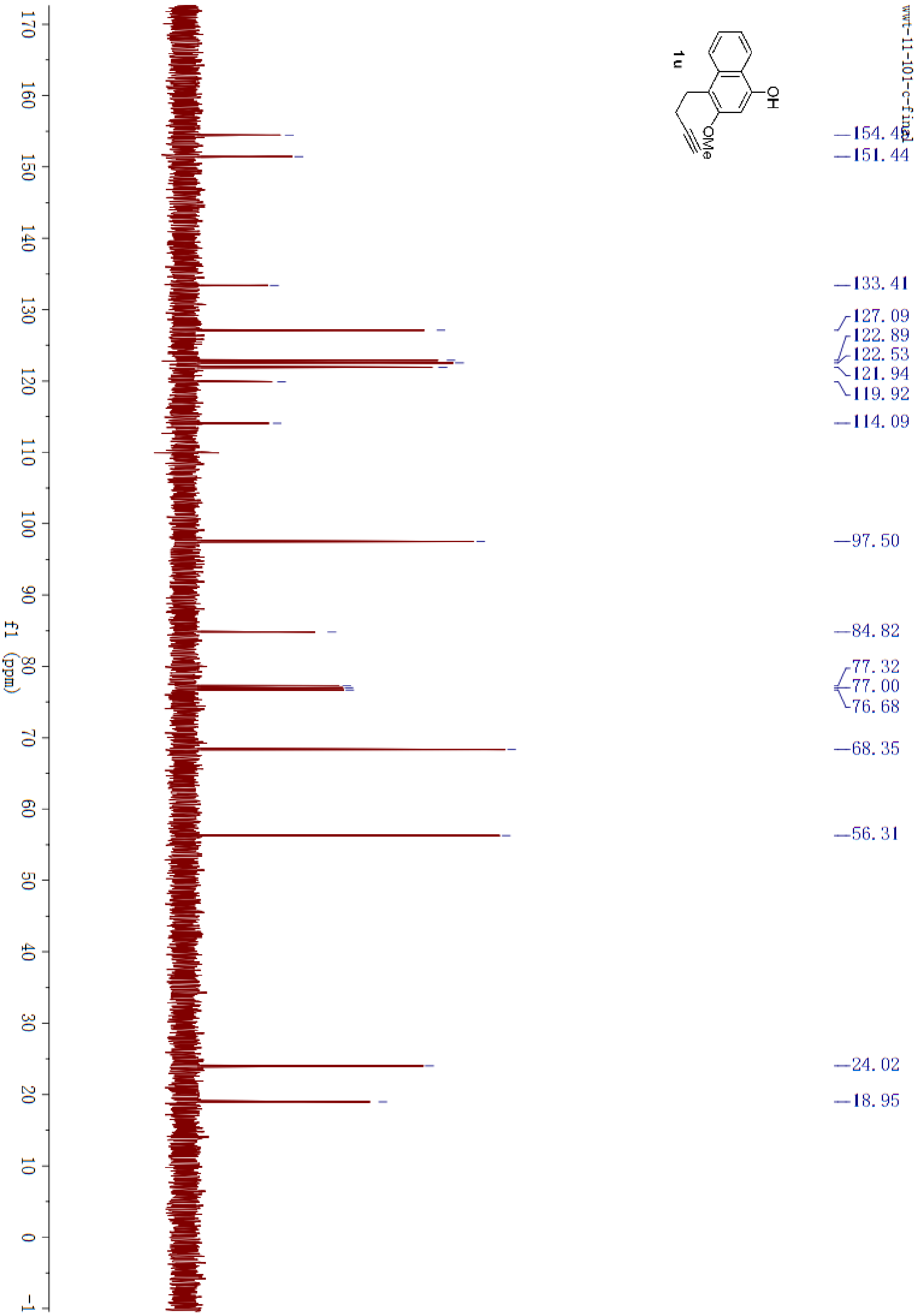
wwt-11-101-c-final
1u
OH
OMe
154.42
151.44
133.41
127.09
122.89
122.53
121.94
119.92
114.09
97.50
84.82
77.32
77.00
76.68
68.35
56.31
24.02
18.95
f1 (ppm)
170
160
150
140
130
120
110
100
90
80
70
60
50
40
30
20
10
0
-1

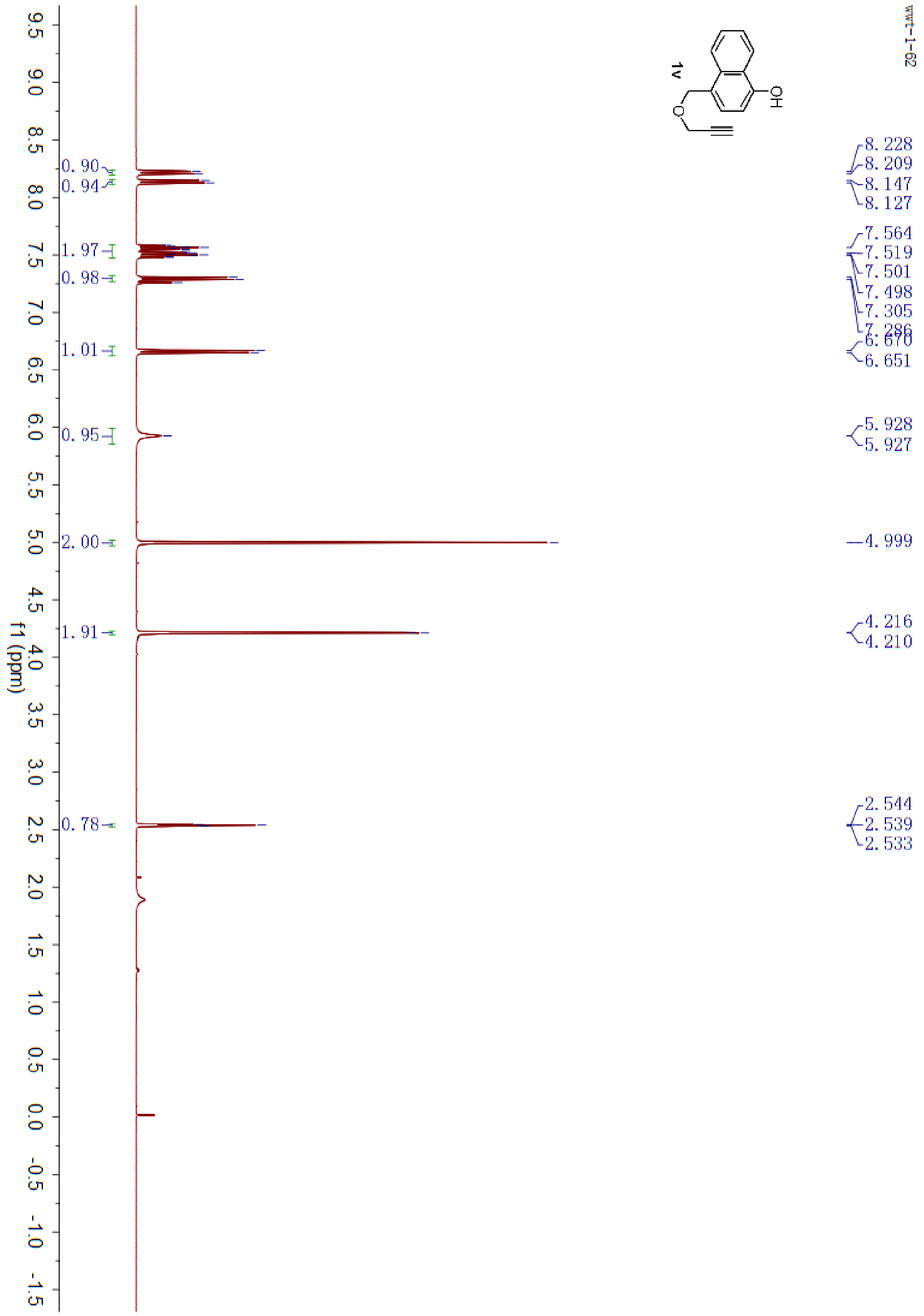
wwt-1-62
1v
OH
O
8.228
8.209
8.147
8.127
7.564
7.519
7.501
7.498
7.305
7.286
6.670
6.651
5.928
5.927
4.999
4.216
4.210
2.544
2.539
2.533
0.90
0.94
1.97
0.98
1.01
0.95
2.00
1.91
0.78
f1 (ppm)

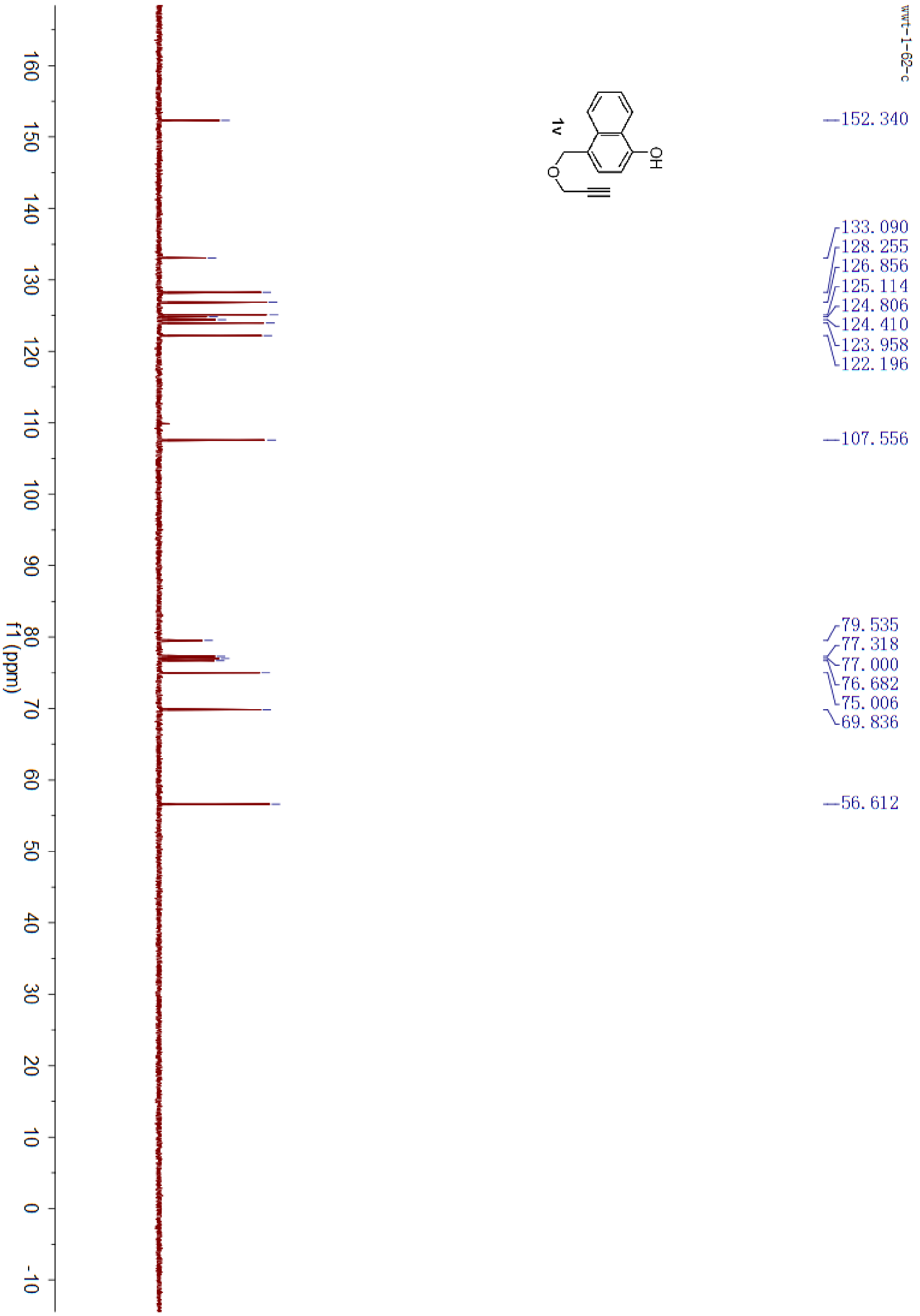
wwt-1-62-c
1v
OH
O
152.340
133.090
128.255
126.856
125.114
124.806
124.410
123.958
122.196
107.556
79.535
77.318
77.000
76.682
75.006
69.836
56.612
160
150
140
130
120
110
100
90
80
70
60
50
40
30
20
10
0
-10
f1 (ppm)

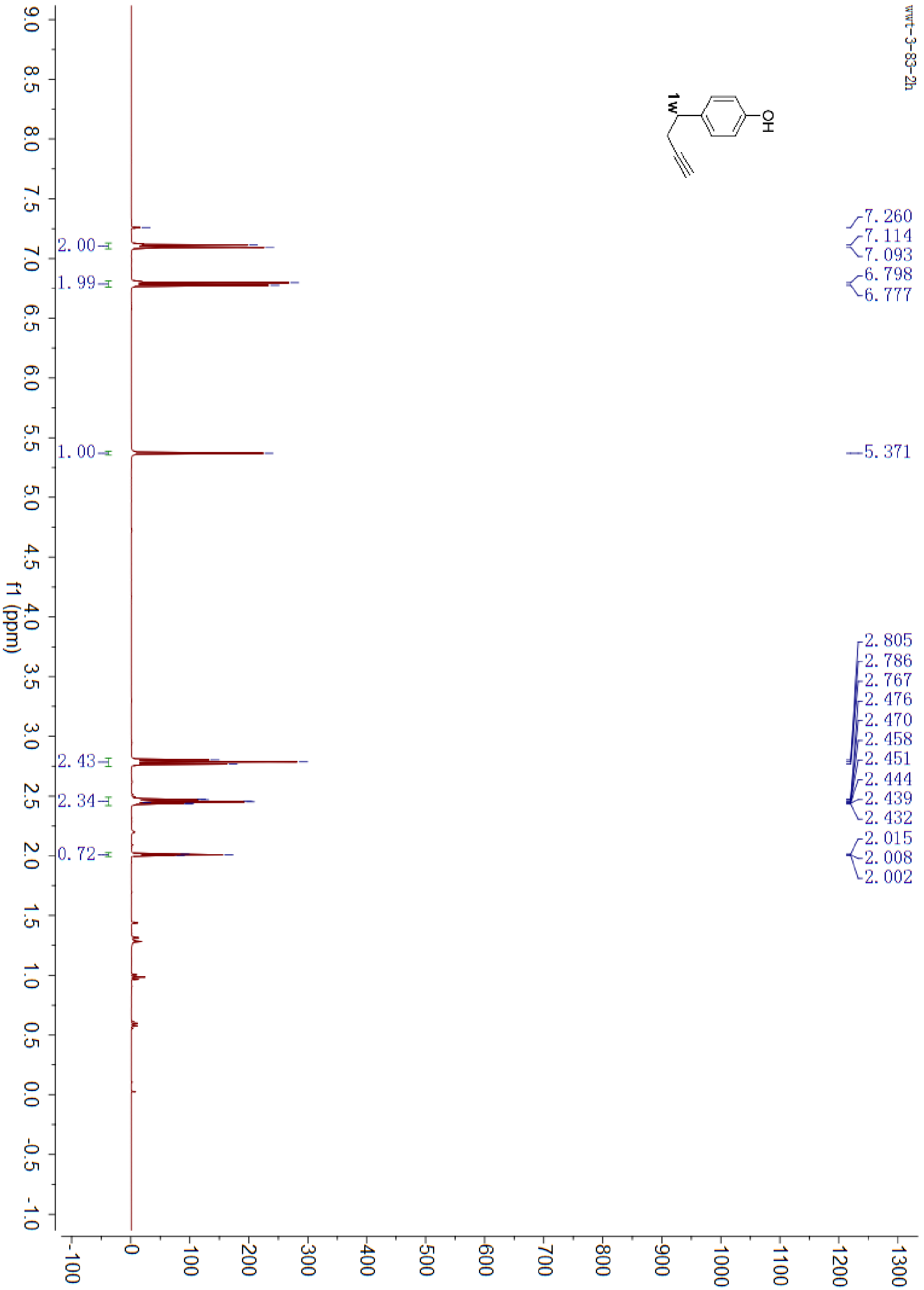

wwt-3-83-2h
OH
1w
7.260
7.114
7.093
6.798
6.777
5.371
2.805
2.786
2.767
2.476
2.470
2.458
2.451
2.444
2.439
2.432
2.015
2.008
2.002
2.00
1.99
1.00
2.43
2.34
0.72
f1 (ppm)

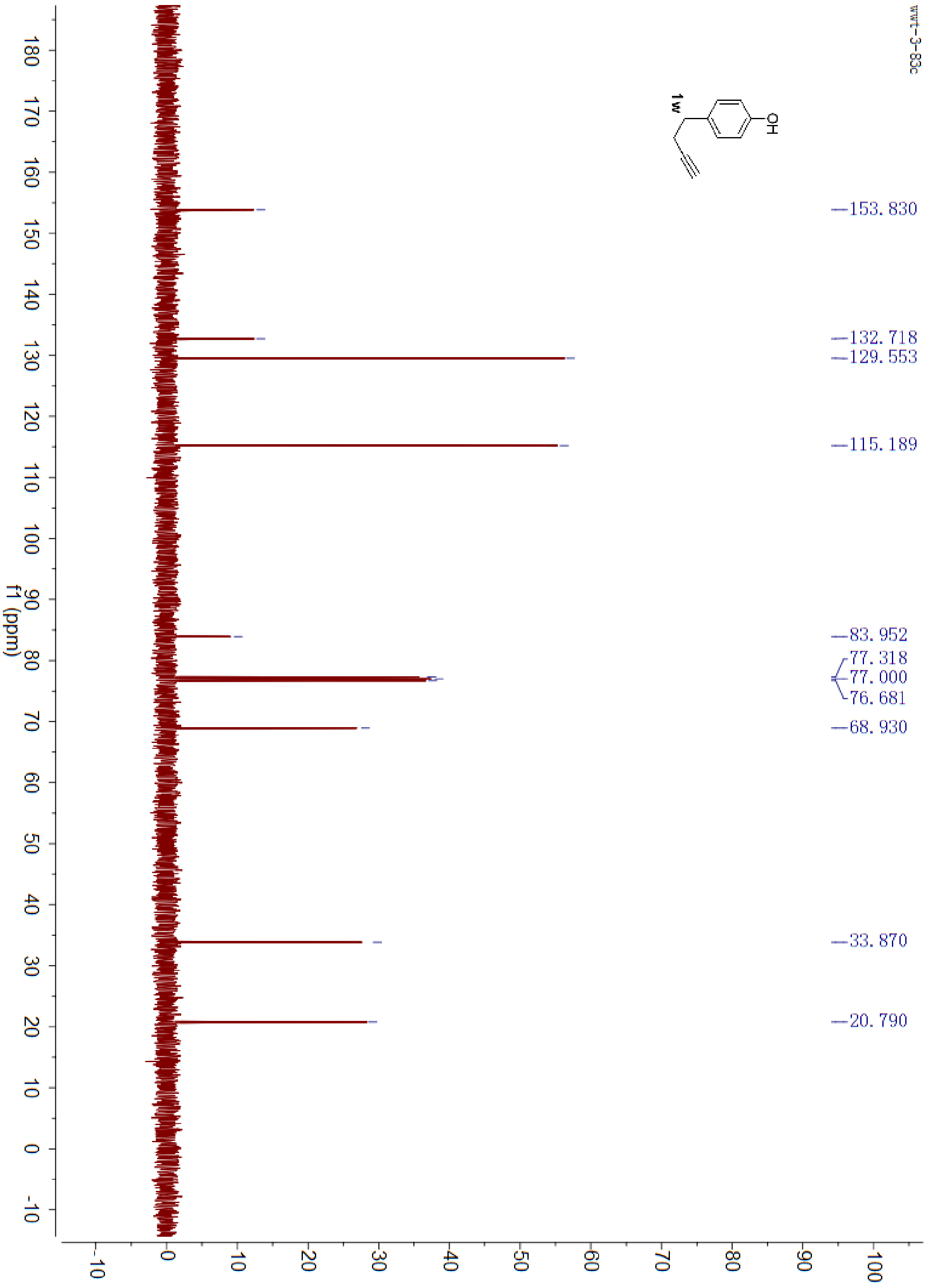
wwt-3-83c
1w
OH
153.830
132.718
129.553
115.189
83.952
77.318
77.000
76.681
68.930
33.870
20.790
f1 (ppm)

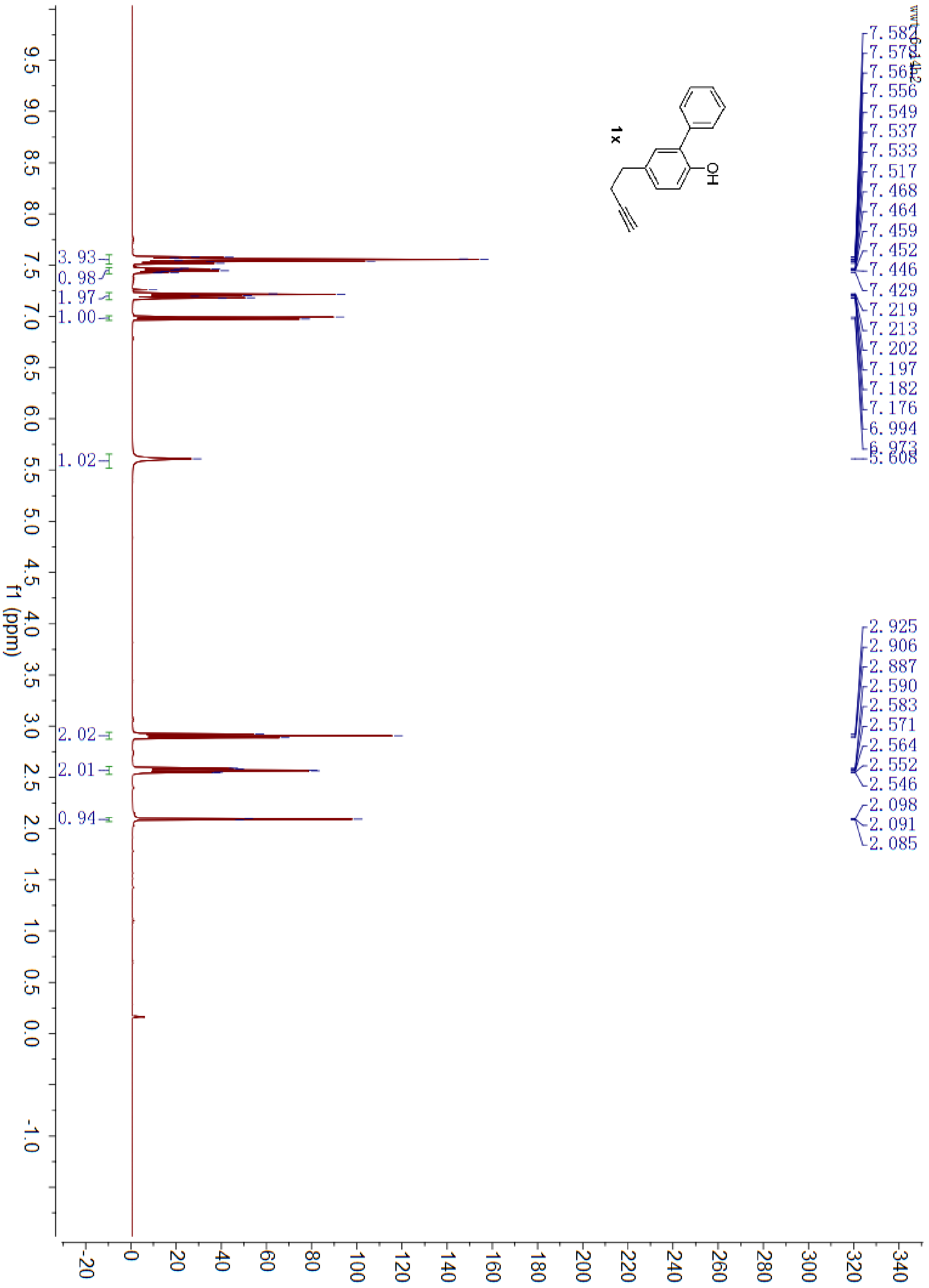

1x
OH
7.587
7.578
7.568
7.556
7.549
7.537
7.533
7.517
7.468
7.464
7.459
7.452
7.446
7.429
7.219
7.213
7.202
7.197
7.182
7.176
6.994
6.973
5.608
2.925
2.906
2.887
2.590
2.583
2.571
2.564
2.552
2.546
2.098
2.091
2.085
3.93
0.98
1.97
1.00
1.02
2.02
2.01
0.94
f1 (ppm)

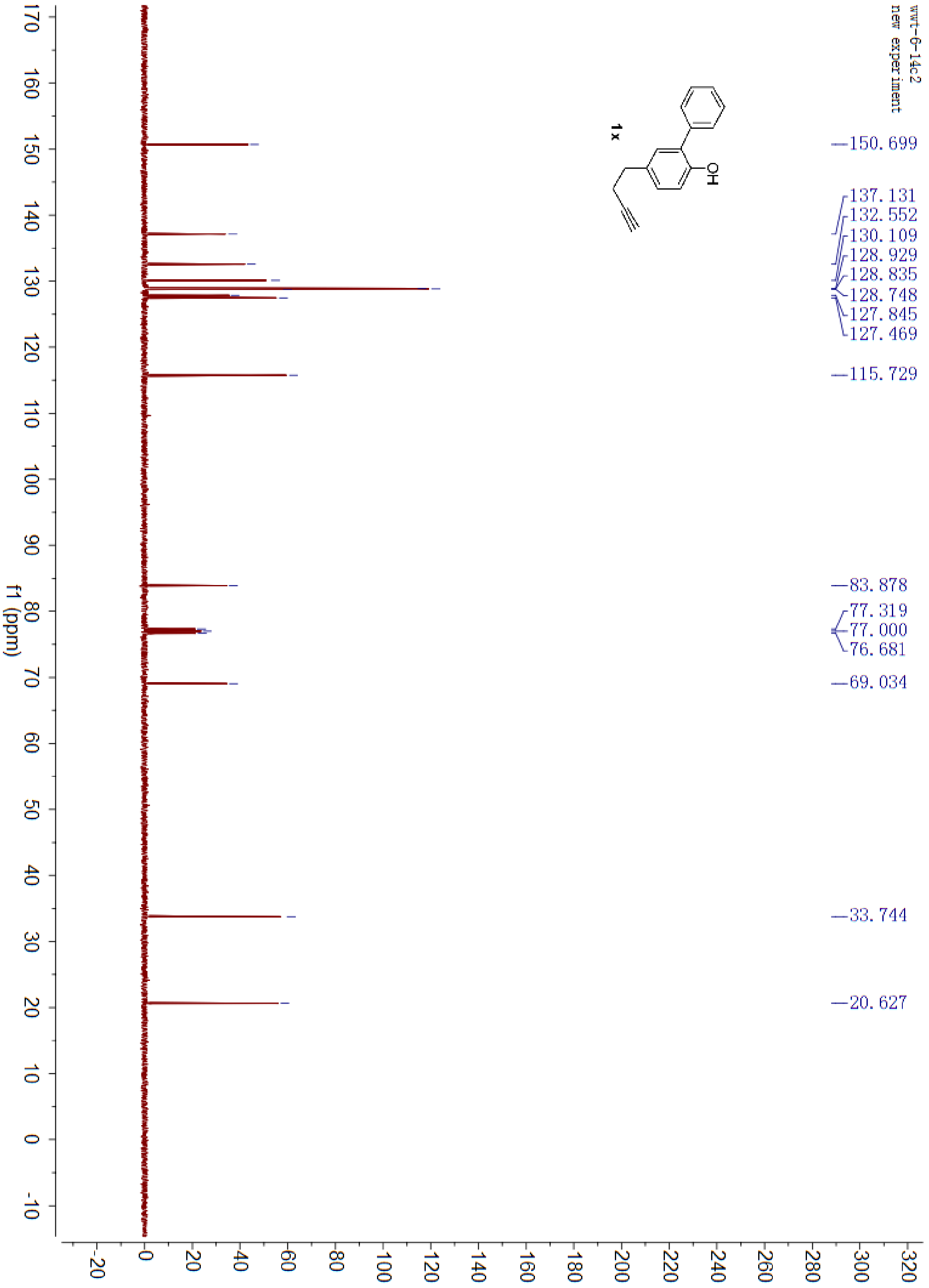
wwt-6-14c2
new experiment
1x
OH
150.699
137.131
132.552
130.109
128.929
128.835
128.748
127.845
127.469
115.729
83.878
77.319
77.000
76.681
69.034
33.744
20.627
f1 (ppm)

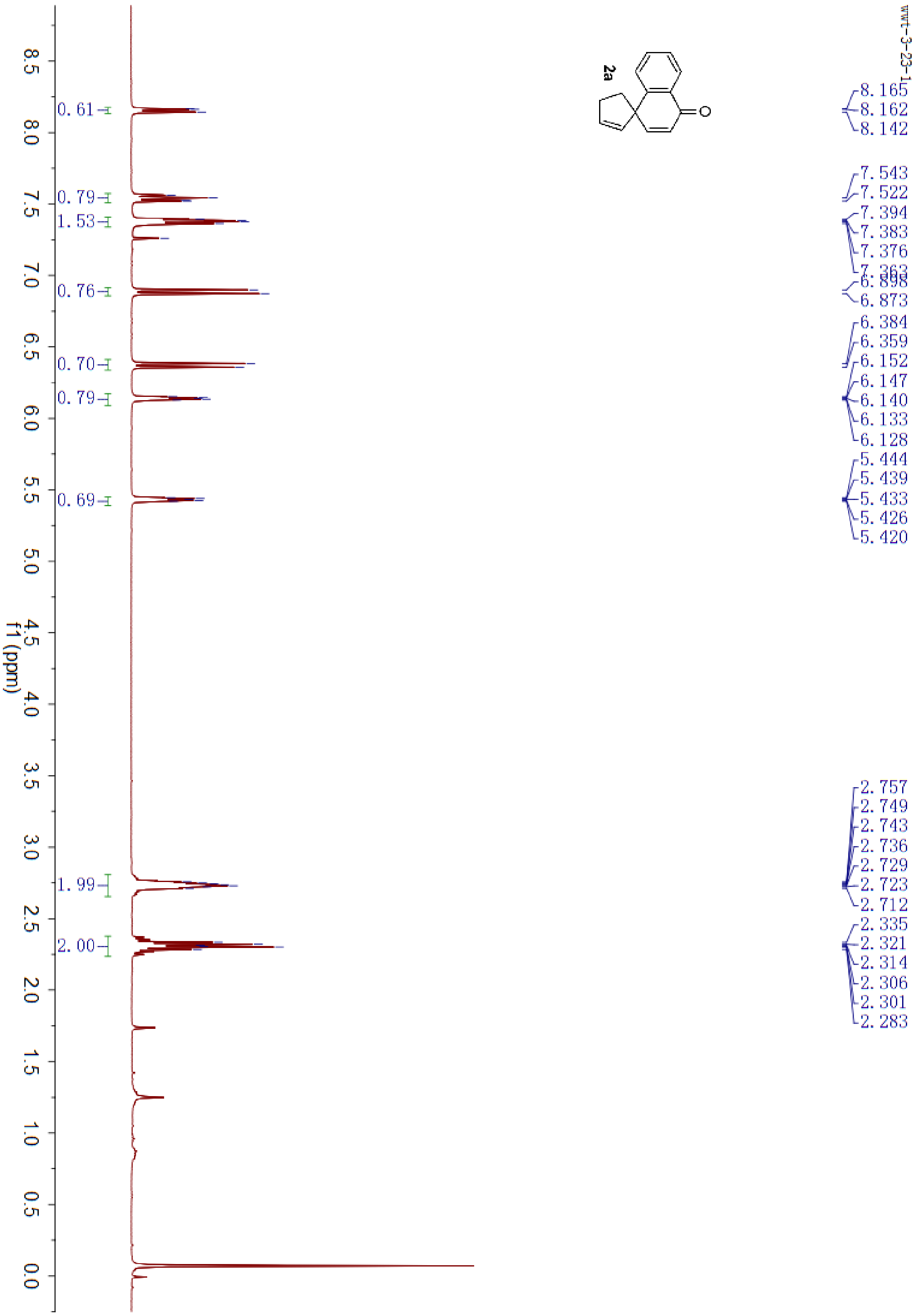

wwt-3-23-1
2a
8.165
8.162
8.142
7.543
7.522
7.394
7.383
7.376
7.363
6.898
6.873
6.384
6.359
6.152
6.147
6.140
6.133
6.128
5.444
5.439
5.433
5.426
5.420
2.757
2.749
2.743
2.736
2.729
2.723
2.712
2.335
2.321
2.314
2.306
2.301
2.283
0.61
0.79
1.53
0.76
0.70
0.79
0.69
1.99
2.00
f1 (ppm)

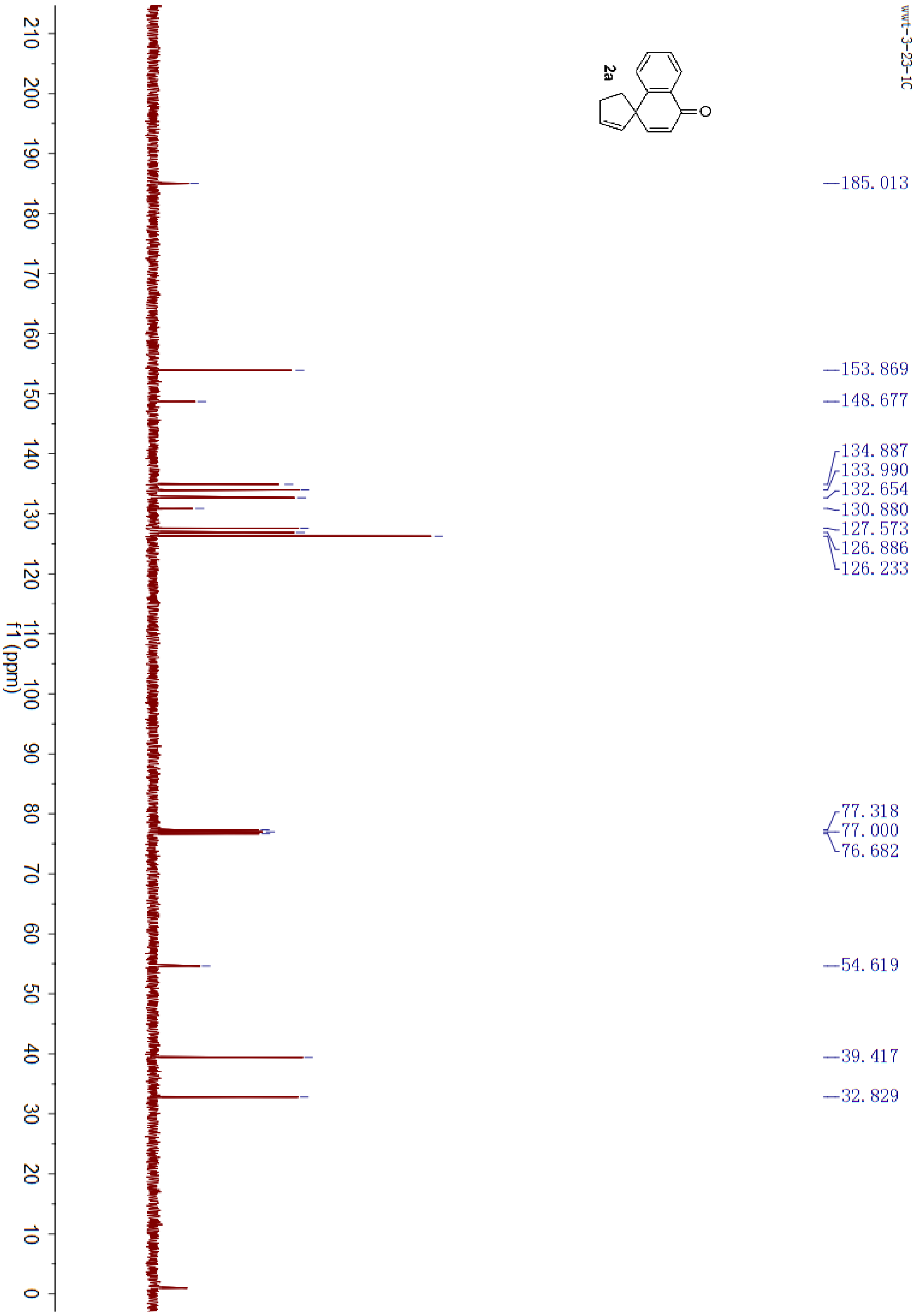
wwt-3-23-1C
2a
185.013
153.869
148.677
134.887
133.990
132.654
130.880
127.573
126.886
126.233
77.318
77.000
76.682
54.619
39.417
32.829
f1 (ppm)

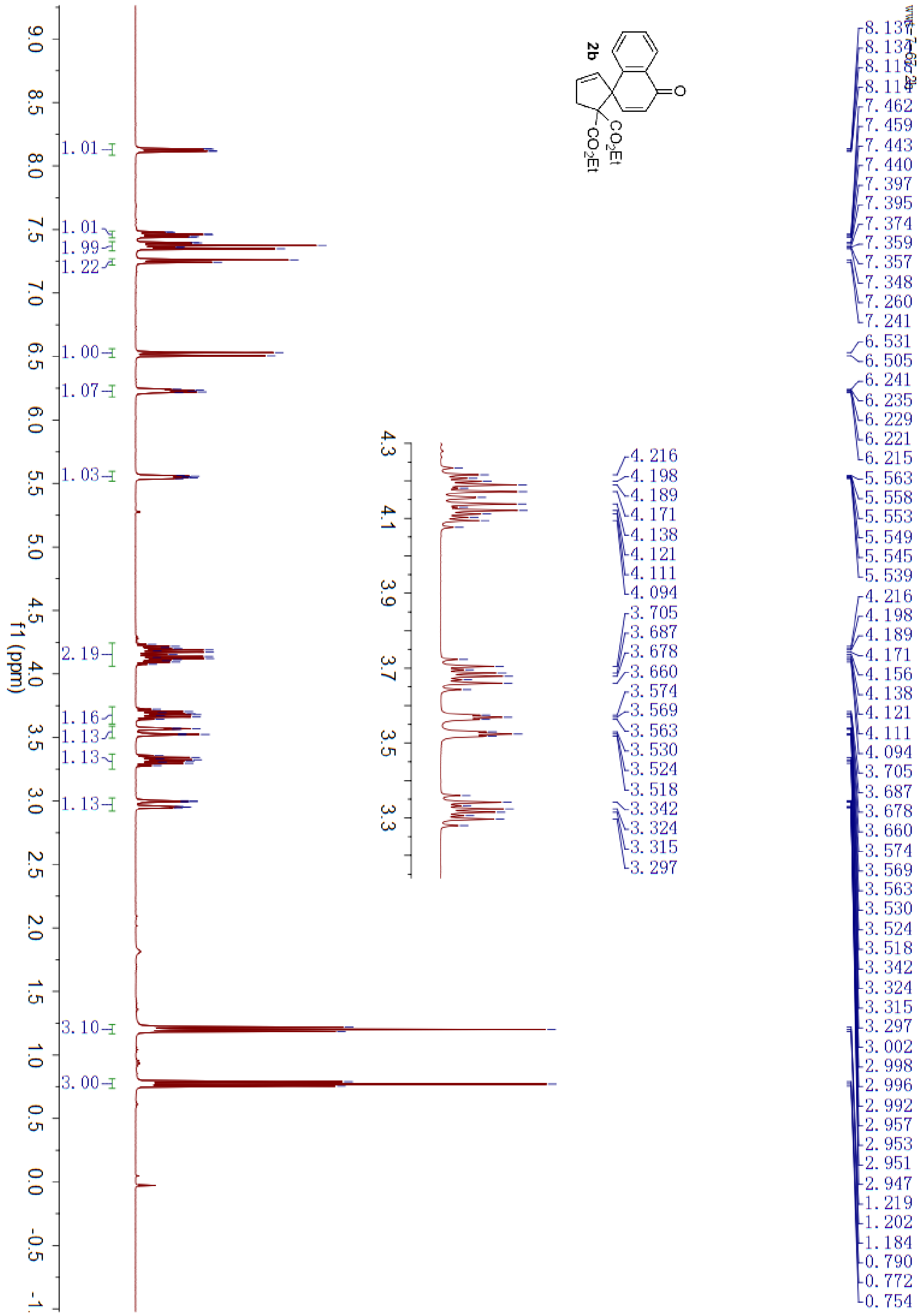

wwb-7-65-2b
2b
O
CO2Et
CO2Et
8.137
8.134
8.118
8.114
7.462
7.459
7.443
7.440
7.397
7.395
7.374
7.359
7.357
7.348
7.260
7.241
6.531
6.505
6.241
6.235
6.229
6.221
6.215
5.563
5.558
5.553
5.549
5.545
5.539
4.216
4.198
4.189
4.171
4.156
4.138
4.121
4.111
4.094
3.705
3.687
3.678
3.660
3.574
3.569
3.563
3.530
3.524
3.518
3.342
3.324
3.315
3.297
3.002
2.998
2.996
2.992
2.957
2.953
2.951
2.947
1.219
1.202
1.184
0.790
0.772
0.754
4.216
4.198
4.189
4.171
4.138
4.121
4.111
4.094
3.705
3.687
3.678
3.660
3.574
3.569
3.563
3.530
3.524
3.518
3.342
3.324
3.315
3.297
4.3
4.1
3.9
3.7
3.5
3.3
1.01
1.01
1.99
1.22
1.00
1.07
1.03
2.19
1.16
1.13
1.13
1.13
3.10
3.00
9.0
8.5
8.0
7.5
7.0
6.5
6.0
5.5
5.0
4.5
4.0
3.5
3.0
2.5
2.0
1.5
1.0
0.5
0.0
-0.5
-1.
f1 (ppm)

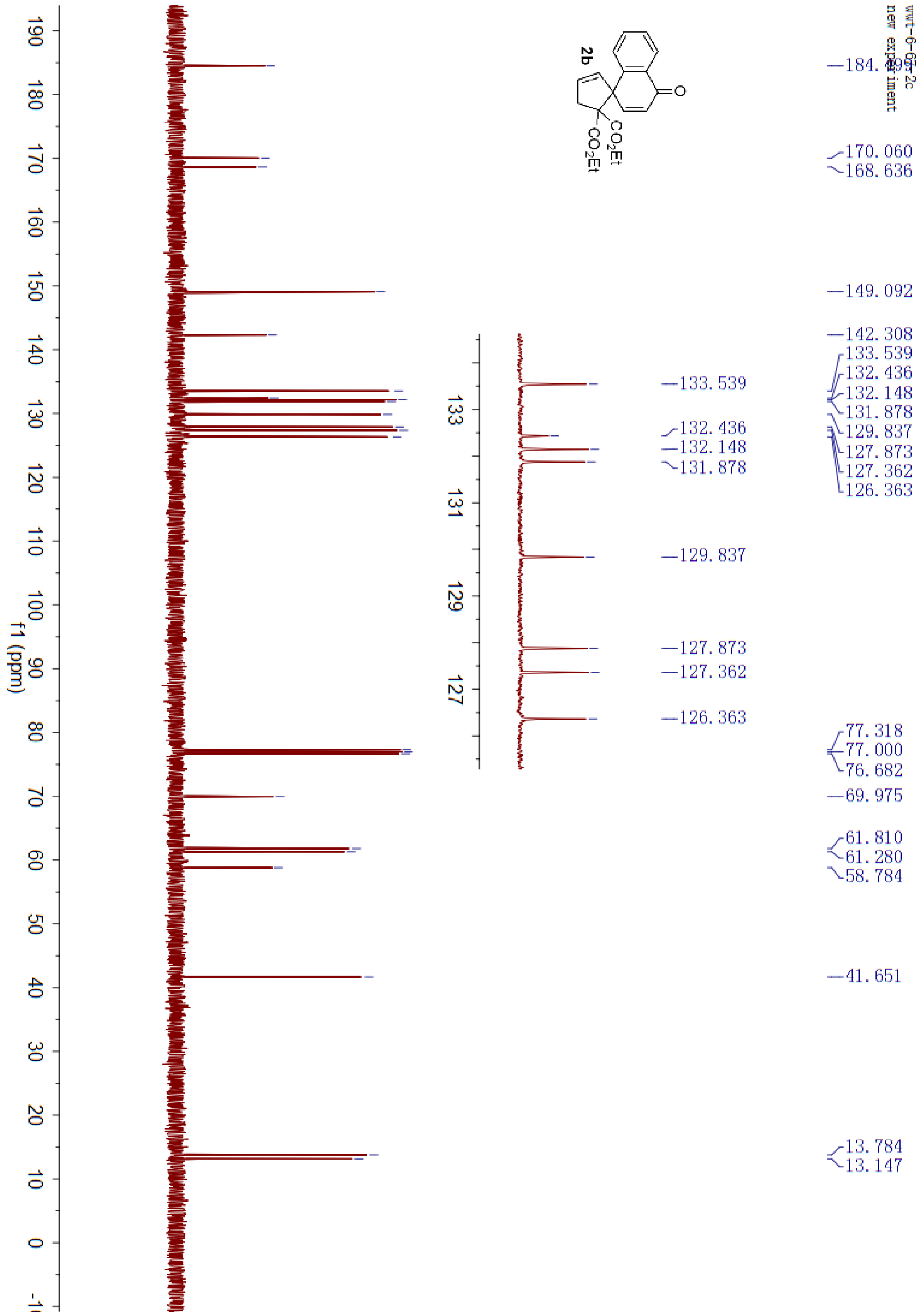
new experiment
2b
CO2Et
CO2Et
170.060
168.636
149.092
142.308
133.539
132.436
132.148
131.878
129.837
127.873
127.362
126.363
77.318
77.000
76.682
69.975
61.810
61.280
58.784
41.651
13.784
13.147
f1 (ppm)

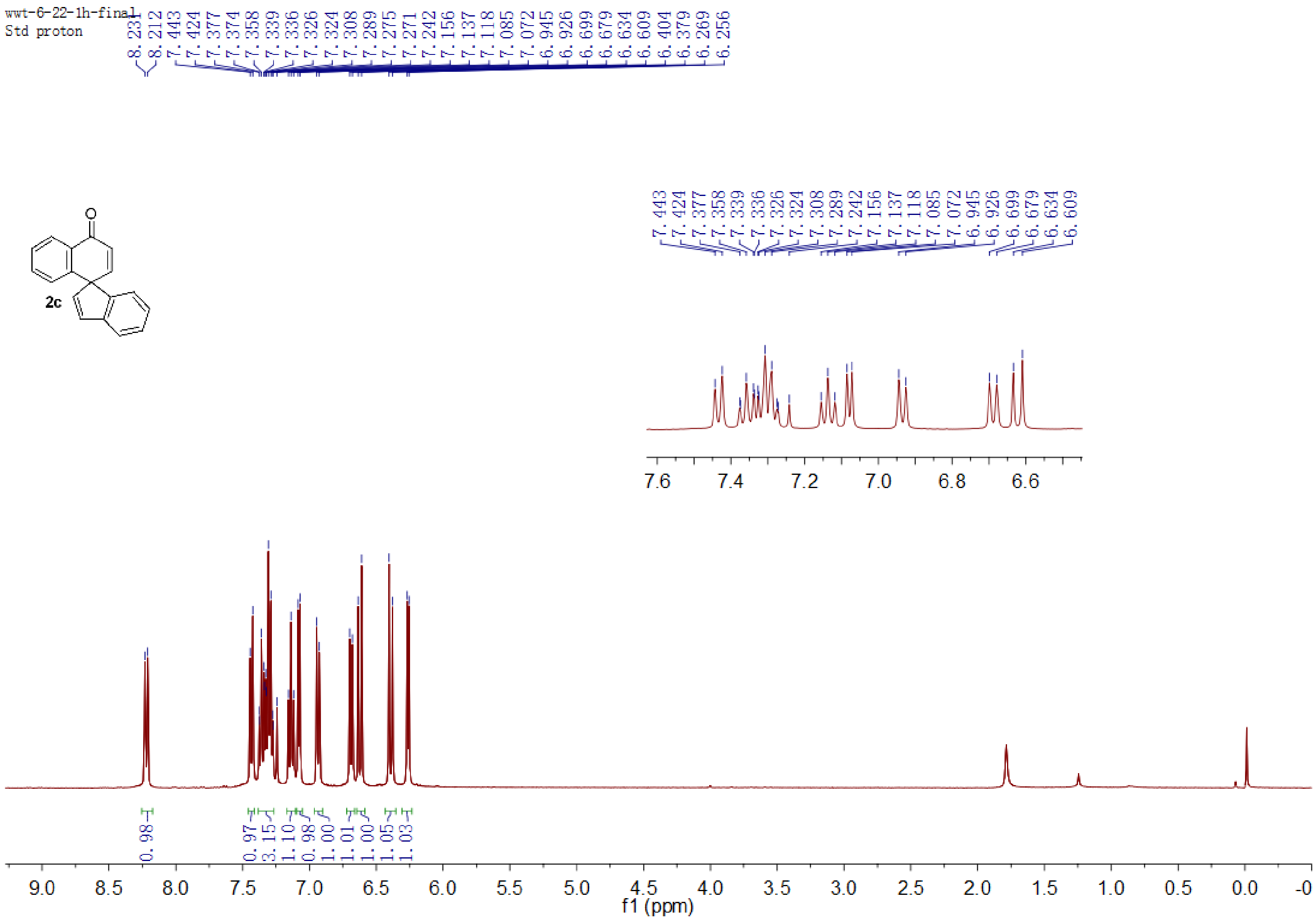

wwt-6-22-1h-final
Std proton
8.231
8.212
7.443
7.424
7.377
7.374
7.358
7.339
7.336
7.326
7.324
7.308
7.289
7.275
7.271
7.242
7.156
7.137
7.118
7.085
7.072
6.945
6.926
6.699
6.679
6.634
6.609
6.404
6.379
6.269
6.256
2c
0.98
0.97
3.15
1.10
0.98
1.00
1.01
1.00
1.05
1.03
9.0
8.5
8.0
7.5
7.0
6.5
6.0
5.5
5.0
4.5
4.0
3.5
3.0
2.5
2.0
1.5
1.0
0.5
0.0
-0
f1 (ppm)

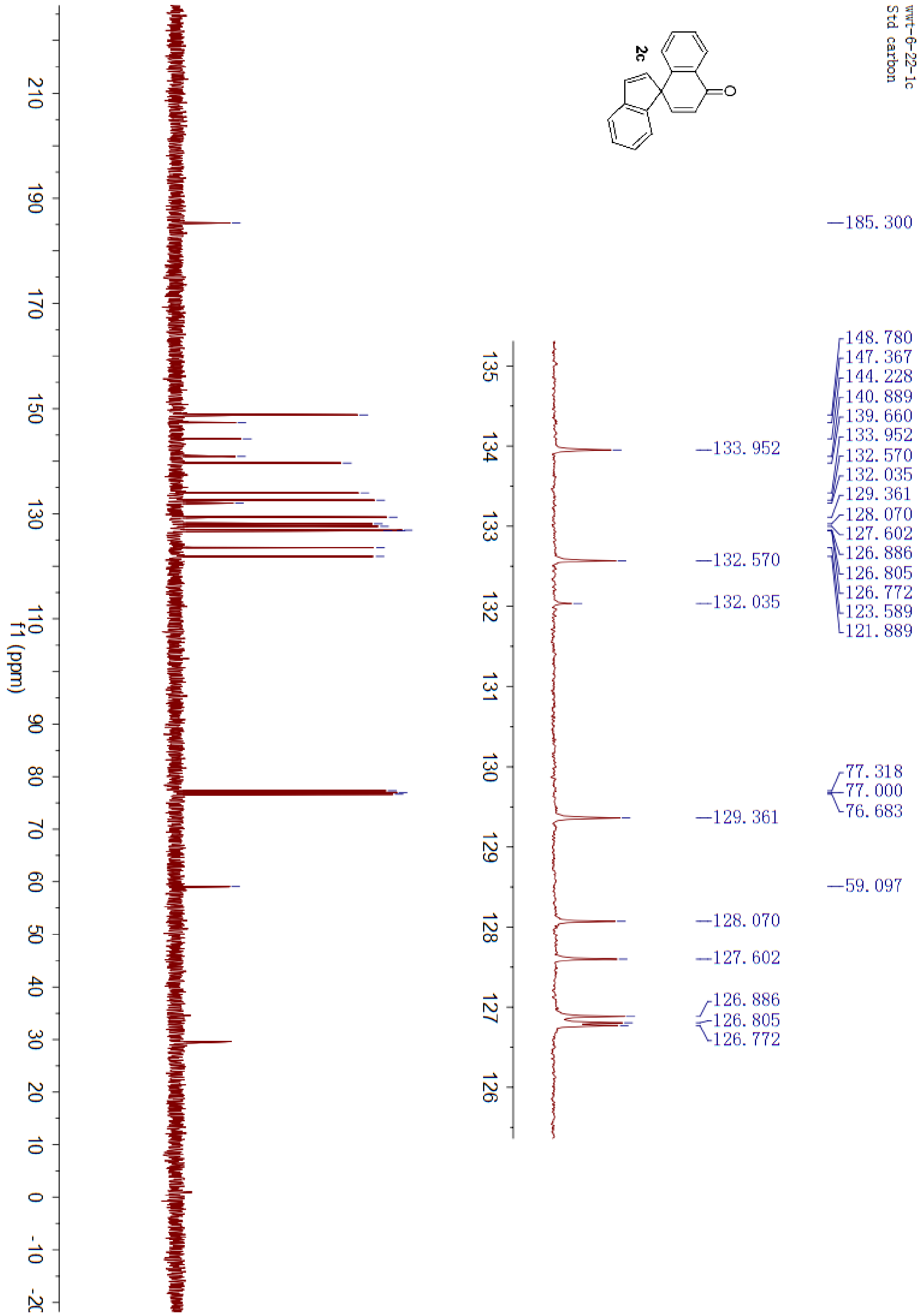

wwt-6-22-1c
Std carbon
2c
185.300
148.780
147.367
144.228
140.889
139.660
133.952
132.570
132.035
129.361
128.070
127.602
126.886
126.805
126.772
123.589
121.889
77.318
77.000
76.683
59.097
f1 (ppm)

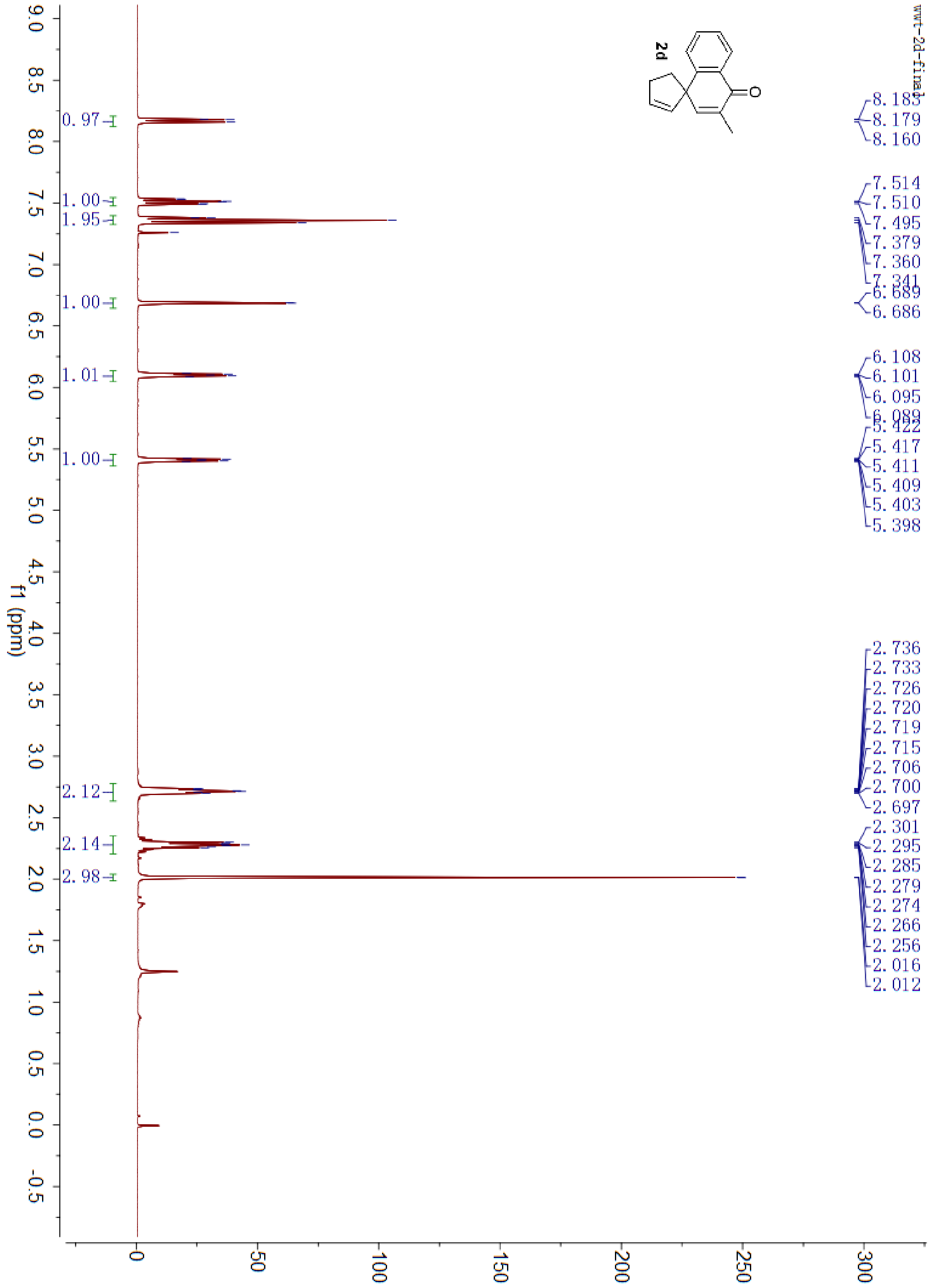

wwt-2d-final
2d
O
8.183
8.179
8.160
7.514
7.510
7.495
7.379
7.360
7.341
6.689
6.686
6.108
6.101
6.095
6.089
5.422
5.417
5.411
5.409
5.403
5.398
2.736
2.733
2.726
2.720
2.719
2.715
2.706
2.700
2.697
2.301
2.295
2.285
2.279
2.274
2.266
2.256
2.016
2.012
0.97
1.00
1.95
1.00
1.01
1.00
2.12
2.14
2.98
f1 (ppm)

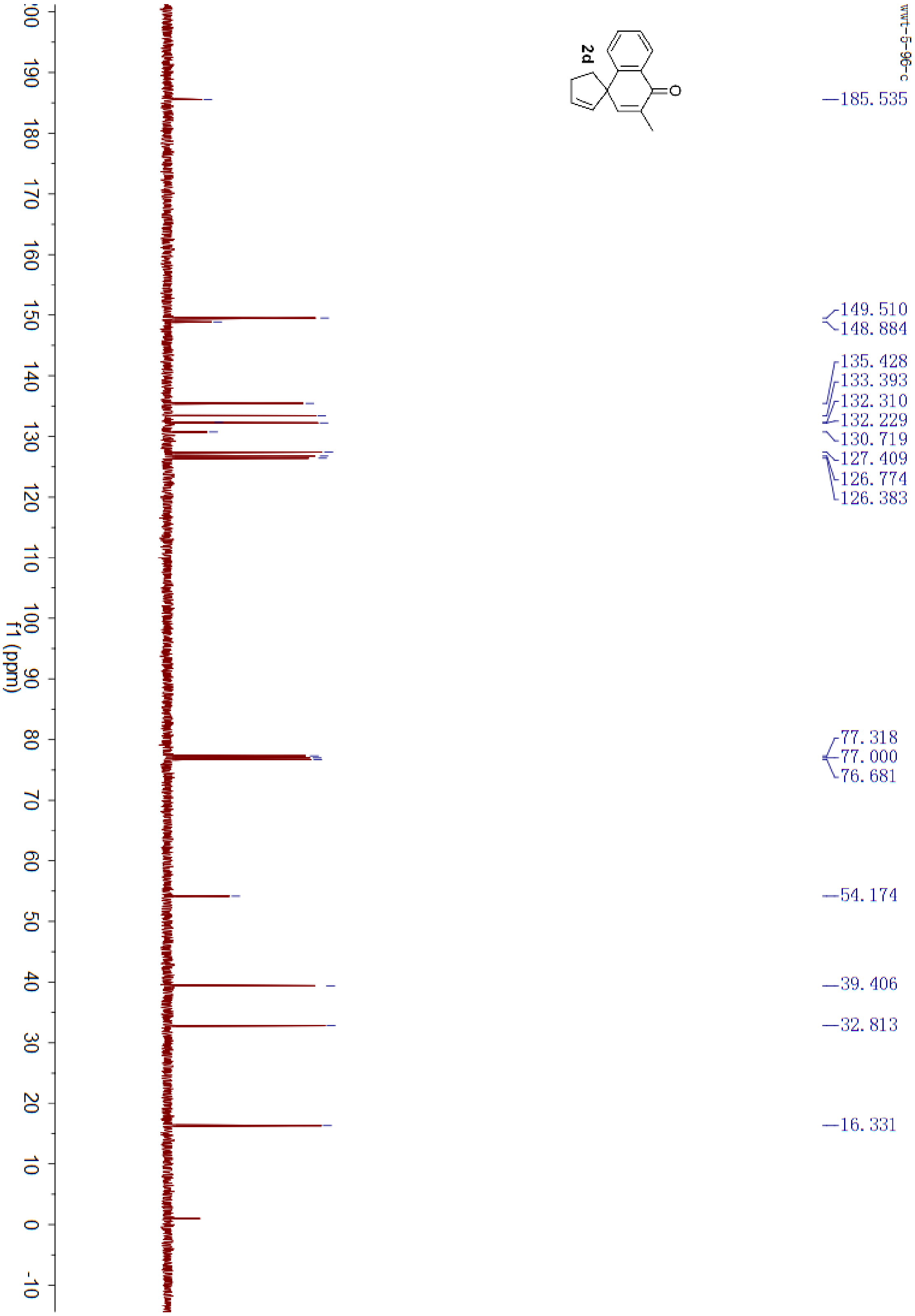
wwt-5-96-c
2d
185.535
149.510
148.884
135.428
133.393
132.310
132.229
130.719
127.409
126.774
126.383
77.318
77.000
76.681
54.174
39.406
32.813
16.331
f1 (ppm)

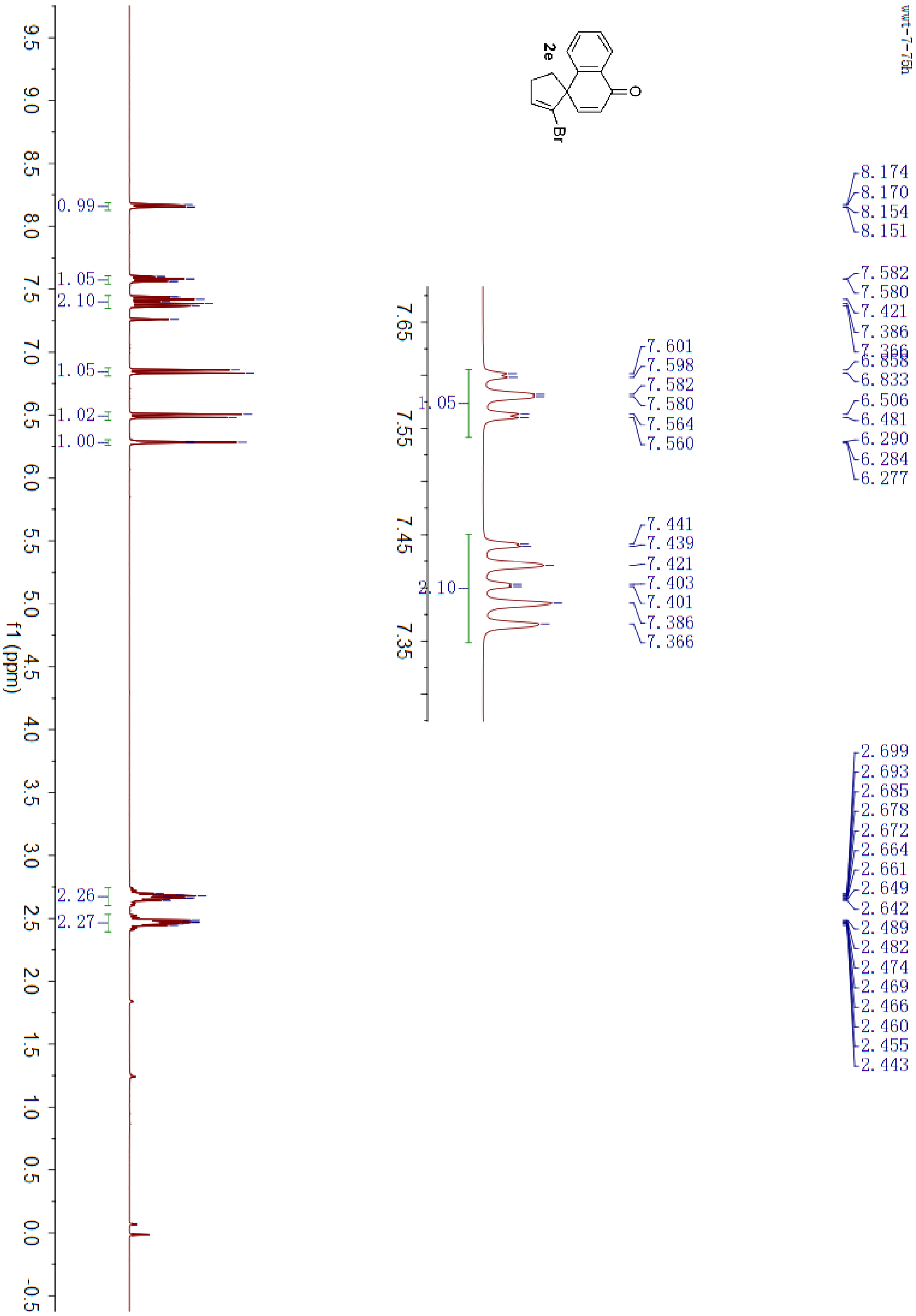
2e
Br
O
8.174
8.170
8.154
8.151
7.582
7.580
7.421
7.386
7.366
6.858
6.833
6.506
6.481
6.290
6.284
6.277
2.699
2.693
2.685
2.678
2.672
2.664
2.661
2.649
2.642
2.489
2.482
2.474
2.469
2.466
2.460
2.455
2.443
7.601
7.598
7.582
7.580
7.564
7.560
7.441
7.439
7.421
7.403
7.401
7.386
7.366
1.05
2.10
7.65
7.55
7.45
7.35
0.99
1.05
2.10
1.05
1.02
1.00
2.26
2.27
f1 (ppm)

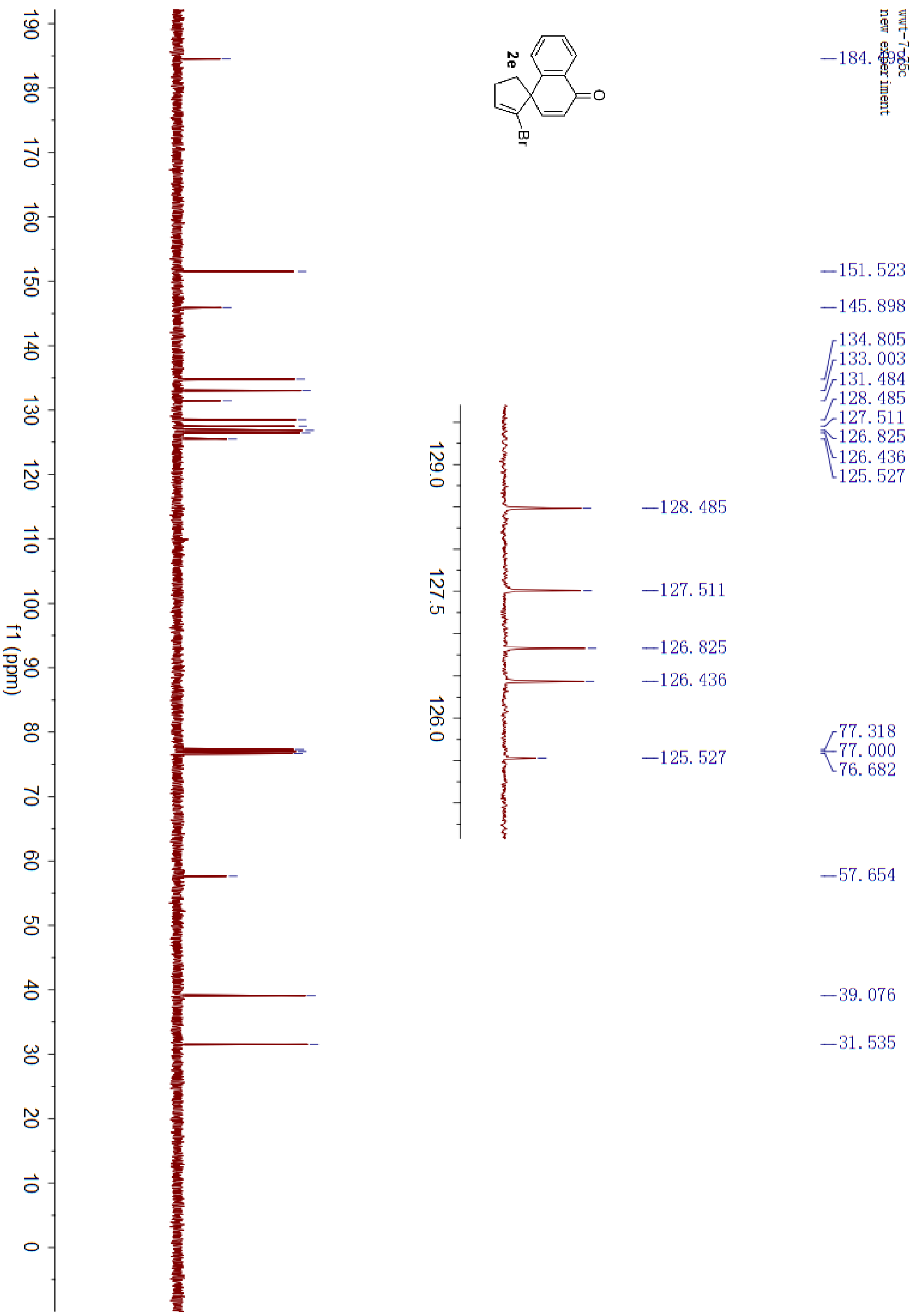
wwt-7-56c
new experiment
2e
Br
O
184.
151.523
145.898
134.805
133.003
131.484
128.485
127.511
126.825
126.436
125.527
77.318
77.000
76.682
57.654
39.076
31.535
129.0
127.5
126.0
f1 (ppm)

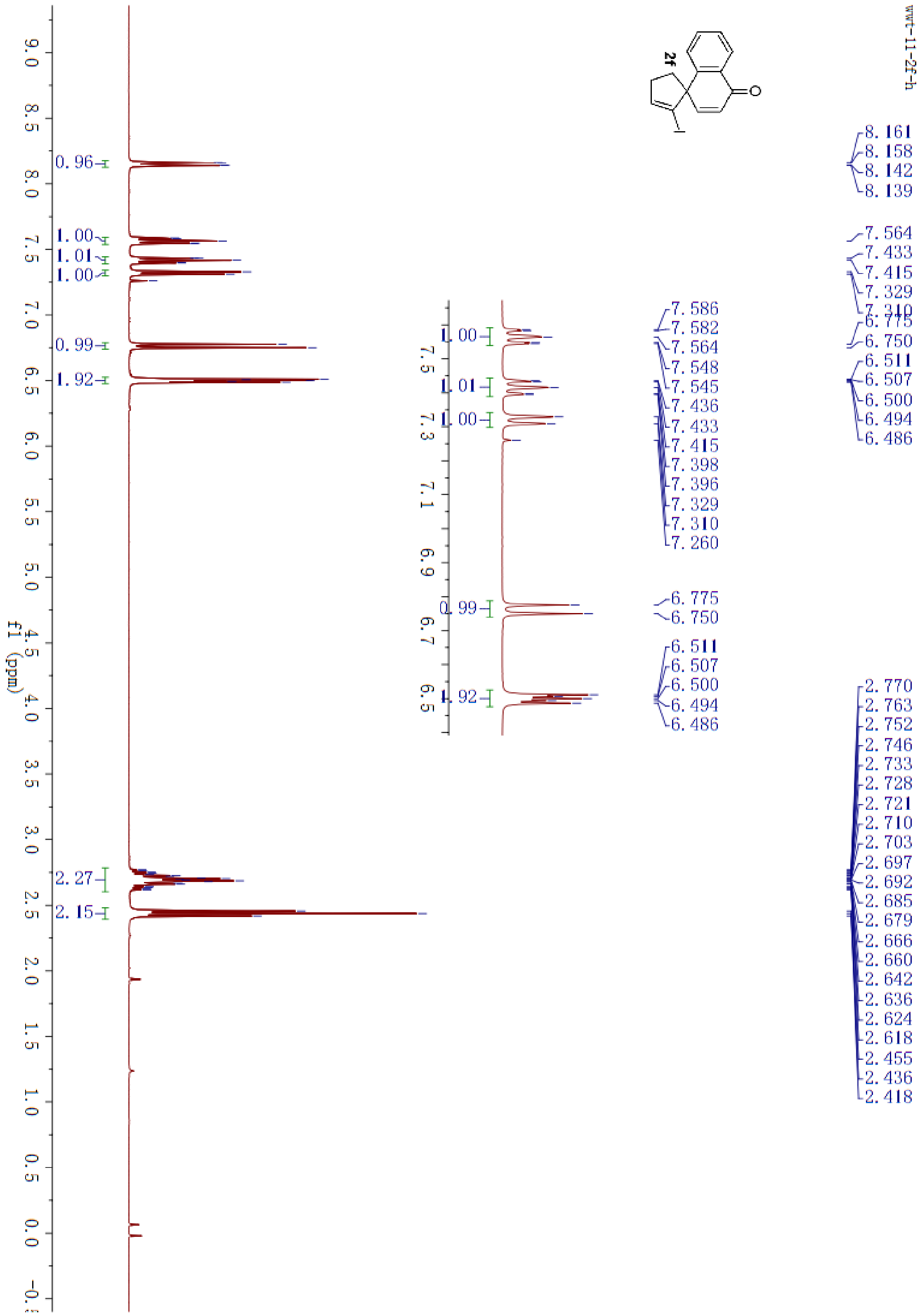
wwt-11-2f-h
2f
8.161
8.158
8.142
8.139
7.564
7.433
7.415
7.329
7.310
6.775
6.750
6.511
6.507
6.500
6.494
6.486
2.770
2.763
2.752
2.746
2.733
2.728
2.721
2.710
2.703
2.697
2.692
2.685
2.679
2.666
2.660
2.642
2.636
2.624
2.618
2.455
2.436
2.418
7.586
7.582
7.564
7.548
7.545
7.436
7.433
7.415
7.398
7.396
7.329
7.310
7.260
6.775
6.750
6.511
6.507
6.500
6.494
6.486
0.96
1.00
1.01
1.00
0.99
1.92
2.27
2.15
f1 (ppm)

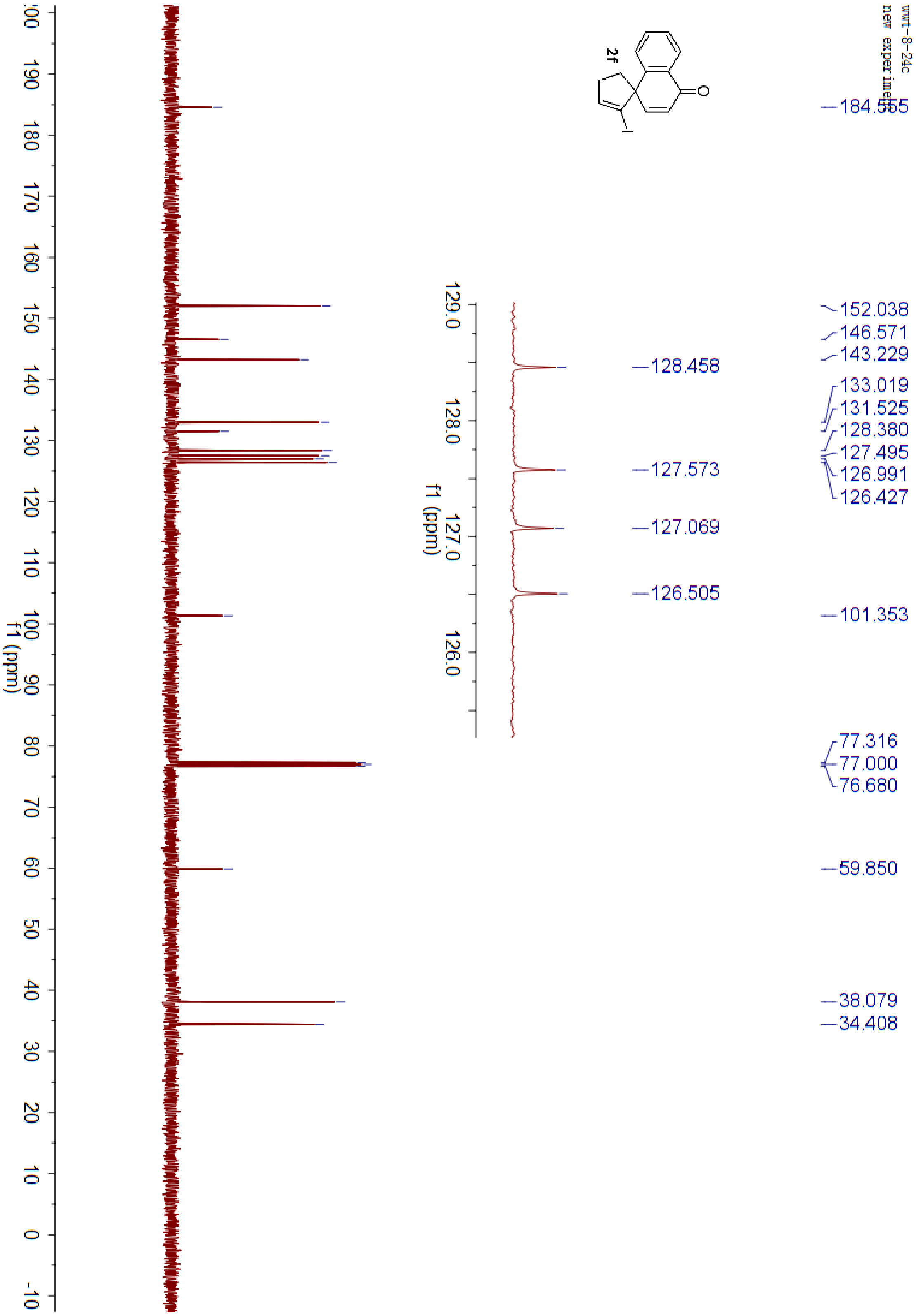
wwt-8-24c
new experiment
2f
184.555
152.038
146.571
143.229
133.019
131.525
128.380
127.495
126.991
126.427
101.353
77.316
77.000
76.680
59.850
38.079
34.408
128.458
127.573
127.069
126.505
f1 (ppm)
f1 (ppm)

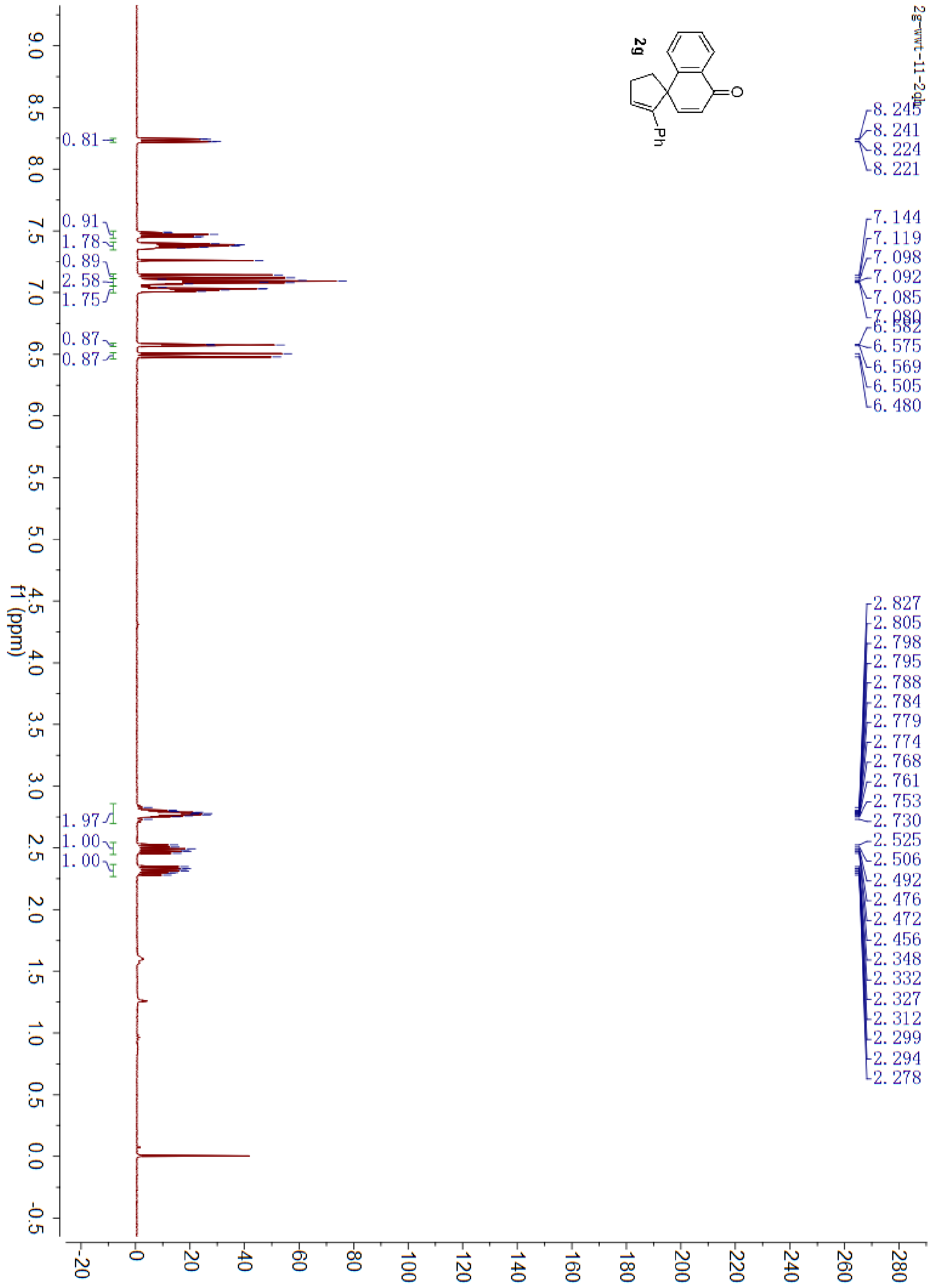
2g-wwt-11-2qb
2g
Ph
8.245
8.241
8.224
8.221
7.144
7.119
7.098
7.092
7.085
7.080
6.582
6.575
6.569
6.505
6.480
2.827
2.805
2.798
2.795
2.788
2.784
2.779
2.774
2.768
2.761
2.753
2.730
2.525
2.506
2.492
2.476
2.472
2.456
2.348
2.332
2.327
2.312
2.299
2.294
2.278
0.81
0.91
1.78
0.89
2.58
1.75
0.87
0.87
1.97
1.00
1.00
f1 (ppm)

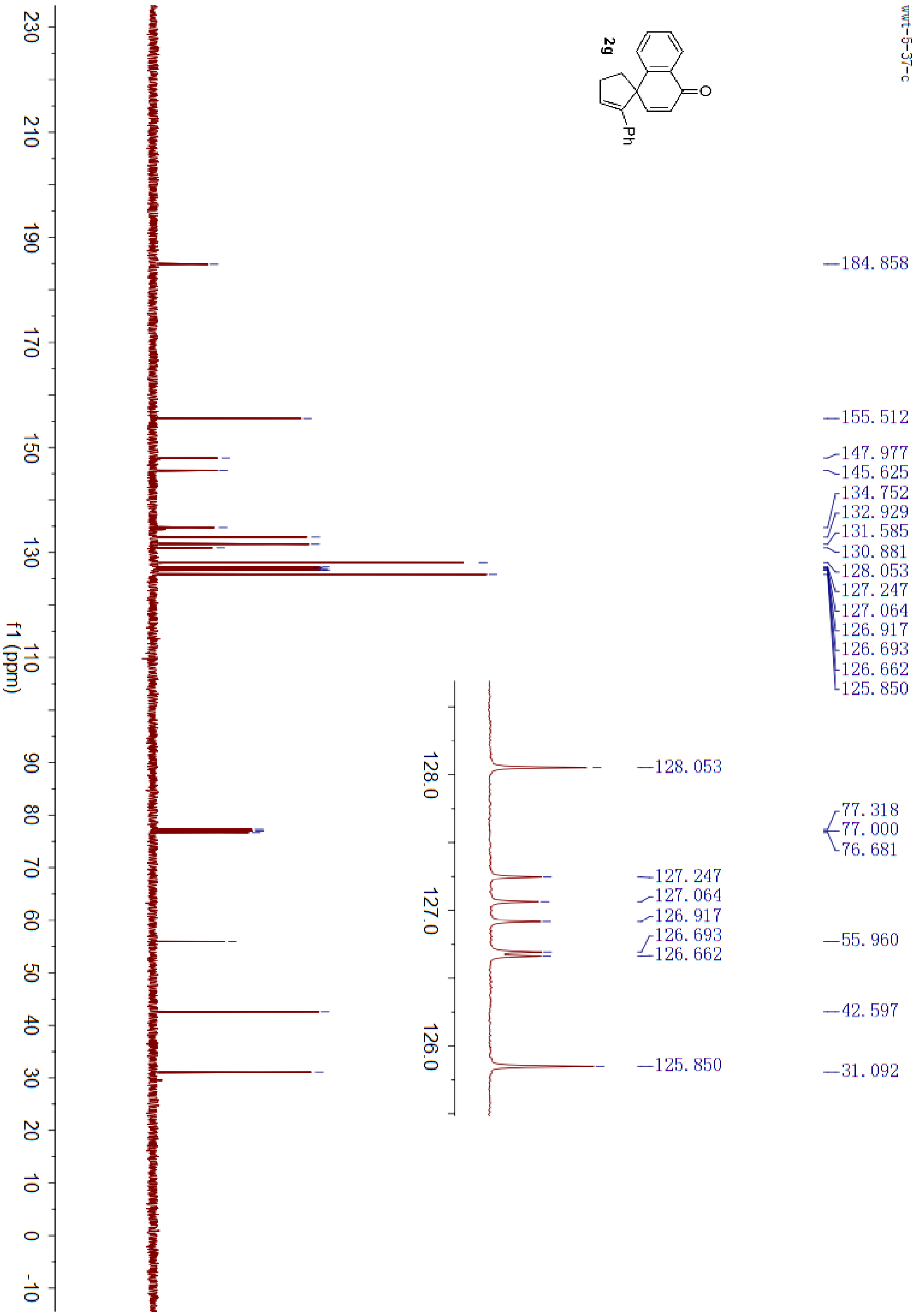
wwt-6-37-c
2g
Ph
O
184.858
155.512
147.977
145.625
134.752
132.929
131.585
130.881
128.053
127.247
127.064
126.917
126.693
126.662
125.850
77.318
77.000
76.681
55.960
42.597
31.092
f1 (ppm)

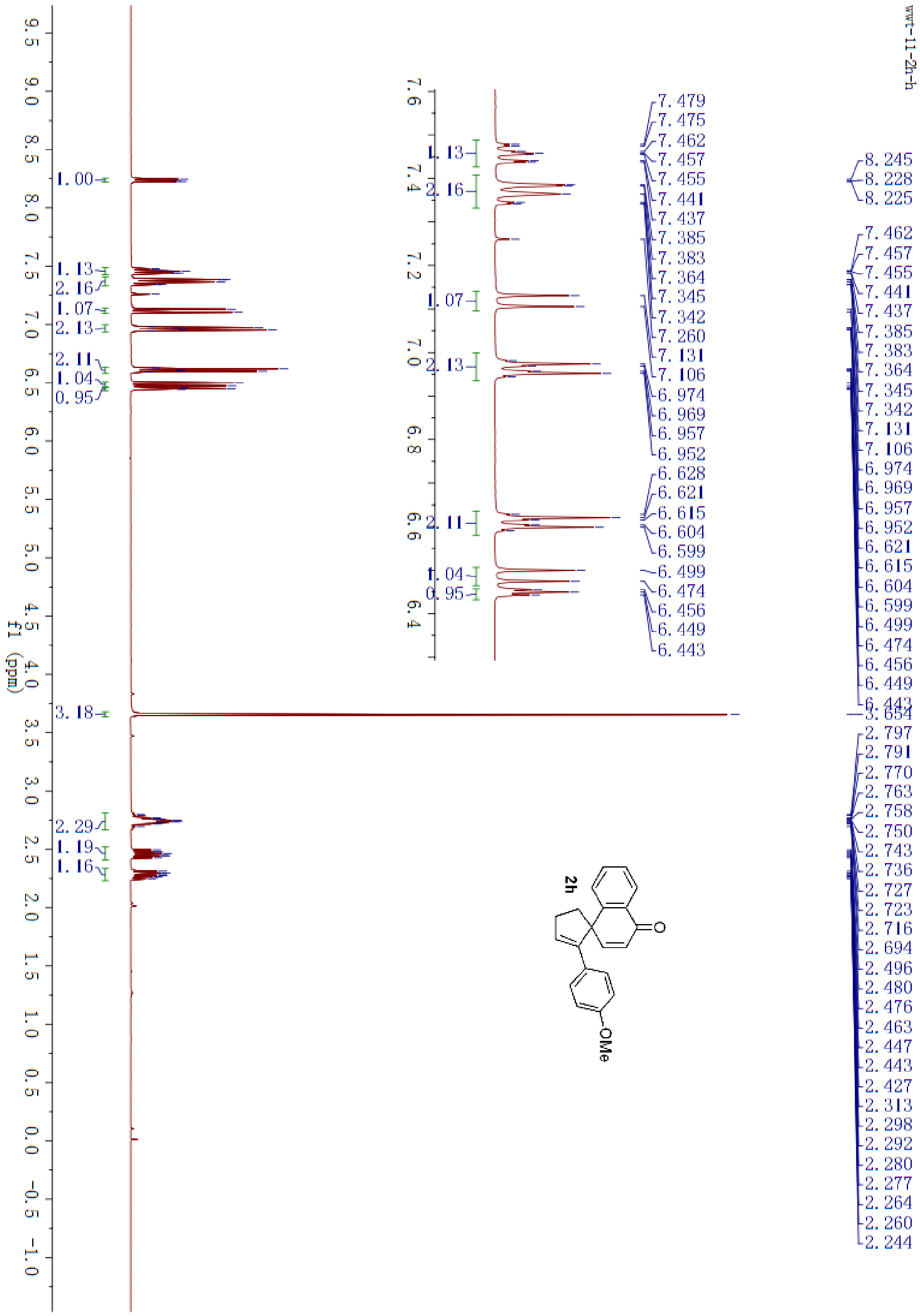
wwt-11-2h-h
8.245
8.228
8.225
7.462
7.457
7.455
7.441
7.437
7.385
7.383
7.364
7.345
7.342
7.131
7.106
6.974
6.969
6.957
6.952
6.621
6.615
6.604
6.599
6.499
6.474
6.456
6.449
6.443
3.654
2.797
2.791
2.770
2.763
2.758
2.750
2.743
2.736
2.727
2.723
2.716
2.694
2.496
2.480
2.476
2.463
2.447
2.443
2.427
2.313
2.298
2.292
2.280
2.277
2.264
2.260
2.244
7.479
7.475
7.260
6.628
1.00
1.13
2.16
1.07
2.13
2.11
1.04
0.95
3.18
2.29
1.19
1.16
2h
O
OMe
f1 (ppm)

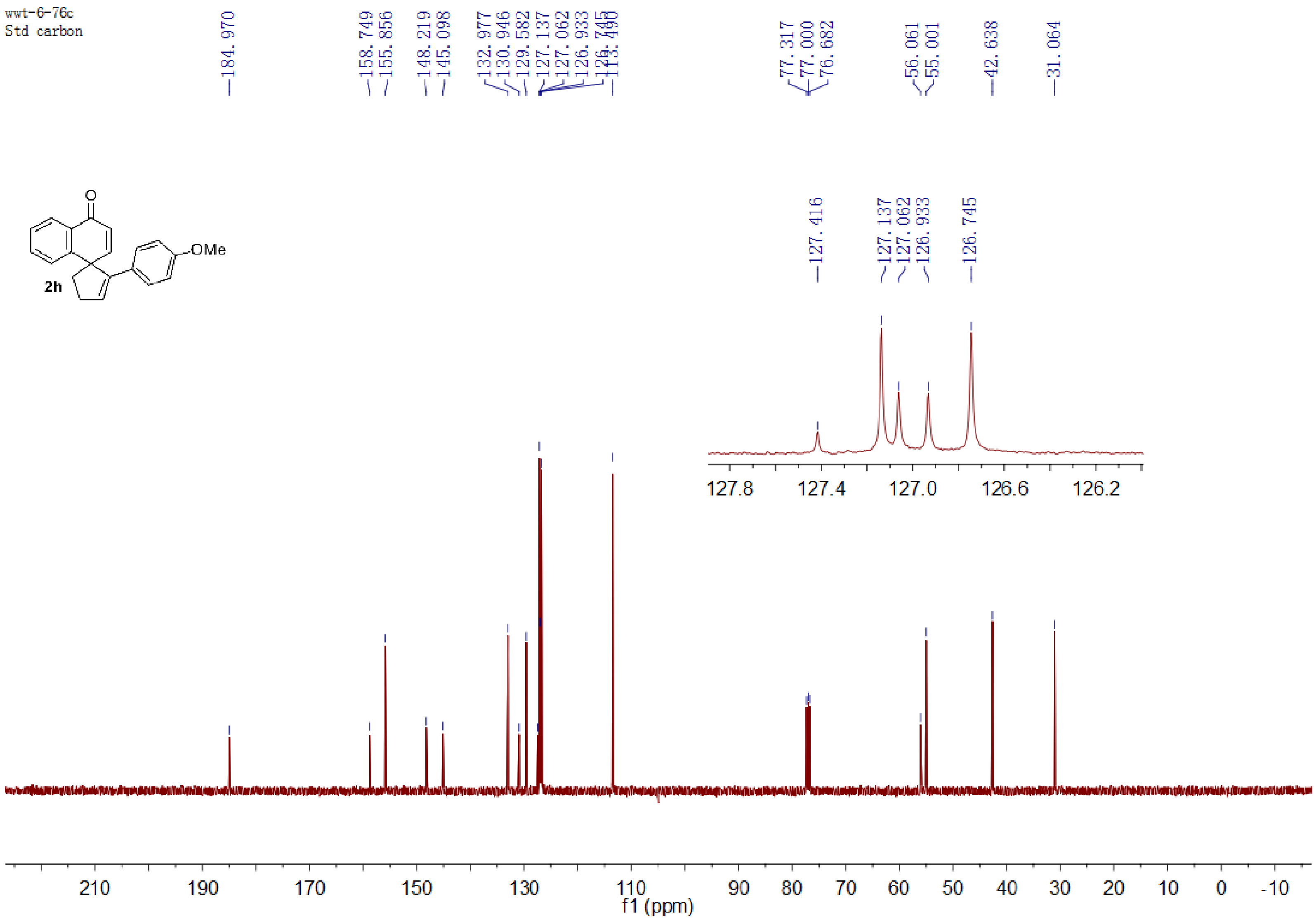
wwt-6-76c
Std carbon
184.970
158.749
155.856
148.219
145.098
132.977
130.946
129.582
127.137
127.062
126.933
126.745
113.490
77.317
77.000
76.682
56.061
55.001
42.638
31.064
OMe
2h
127.416
127.137
127.062
126.933
126.745
127.8
127.4
127.0
126.6
126.2
210
190
170
150
130
110
90
80
70
60
50
40
30
20
10
0
-10
f1 (ppm)

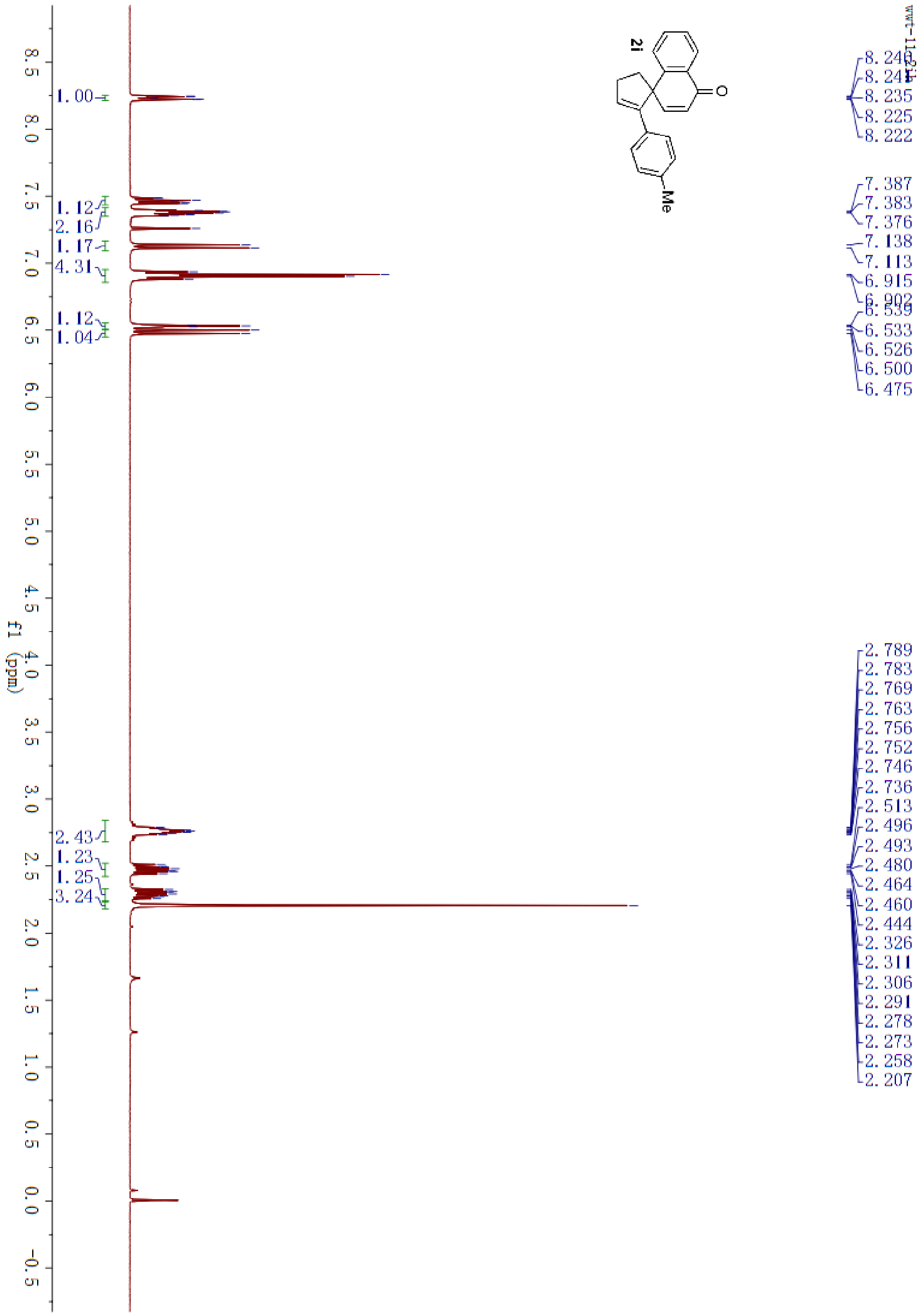
2i
O
Me
8.235
8.225
8.222
7.387
7.383
7.376
7.138
7.113
6.915
6.533
6.526
6.500
6.475
2.789
2.783
2.769
2.763
2.756
2.752
2.746
2.736
2.513
2.496
2.493
2.480
2.464
2.460
2.444
2.326
2.311
2.306
2.291
2.278
2.273
2.258
2.207
1.00
1.12
2.16
1.17
4.31
1.12
1.04
2.43
1.23
1.25
3.24
f1 (ppm)

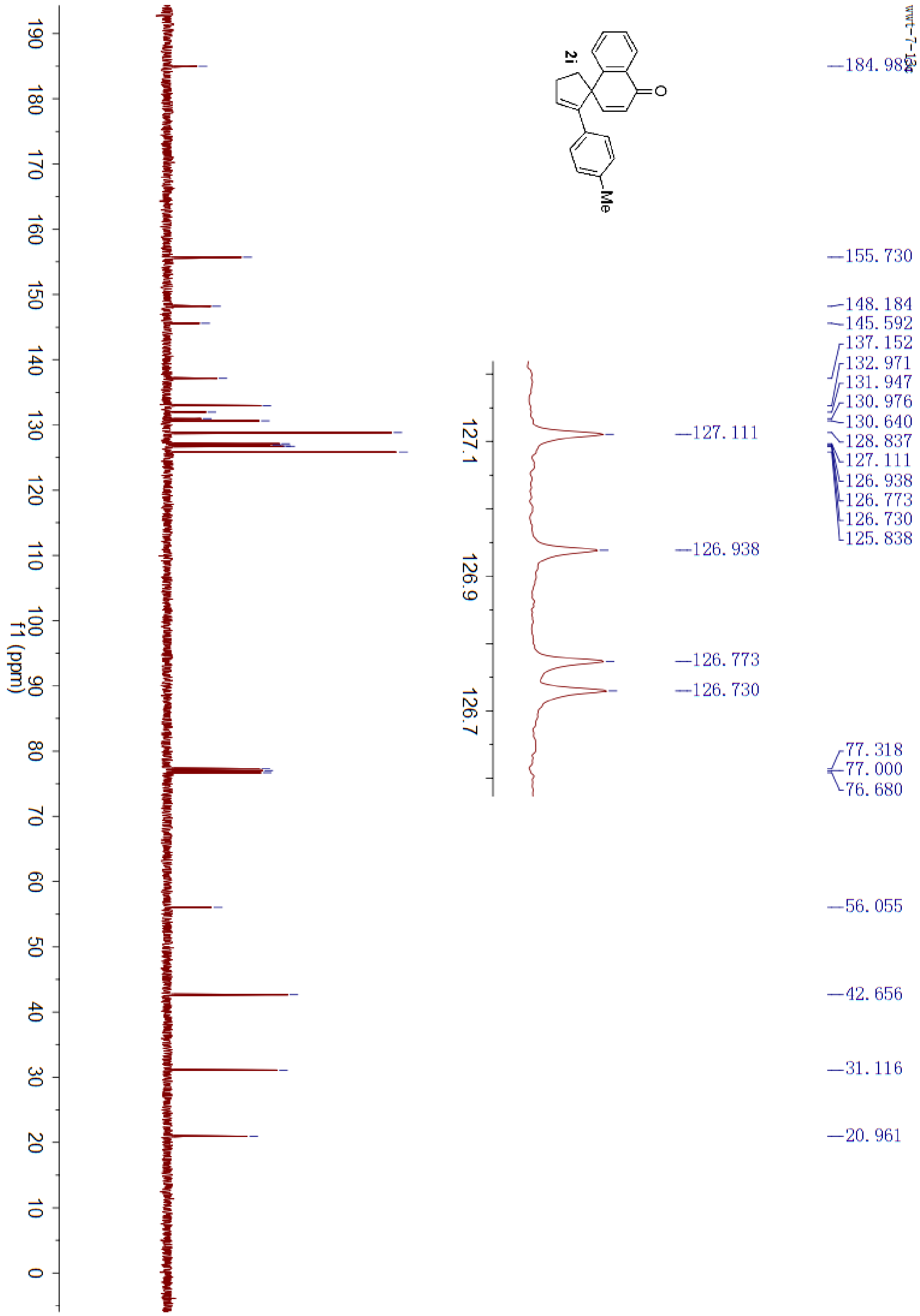
2i
Me
O
184.982
155.730
148.184
145.592
137.152
132.971
131.947
130.976
130.640
128.837
127.111
126.938
126.773
126.730
125.838
77.318
77.000
76.680
56.055
42.656
31.116
20.961
127.1
126.9
126.7
f1 (ppm)

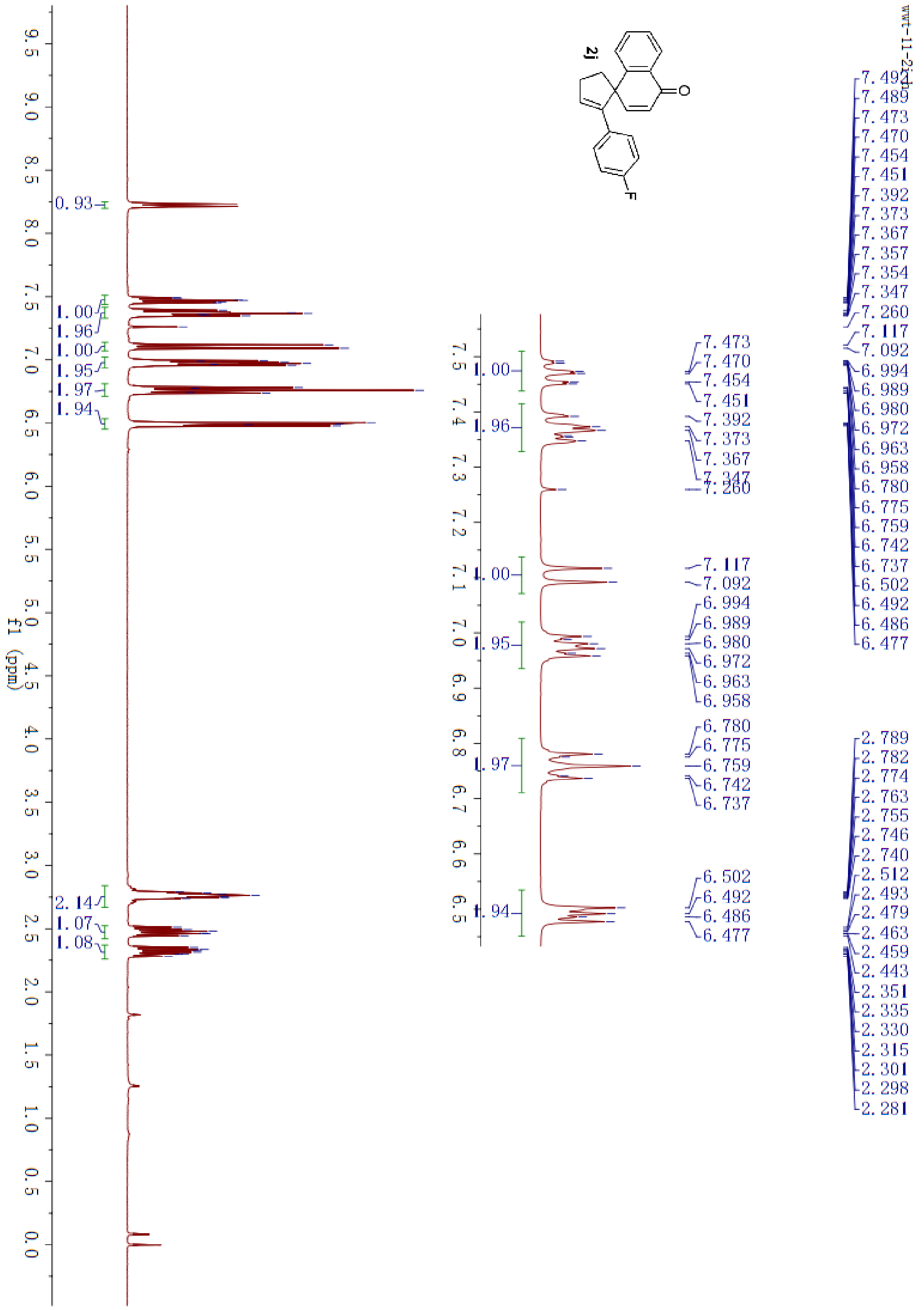
2j
f1 (ppm)

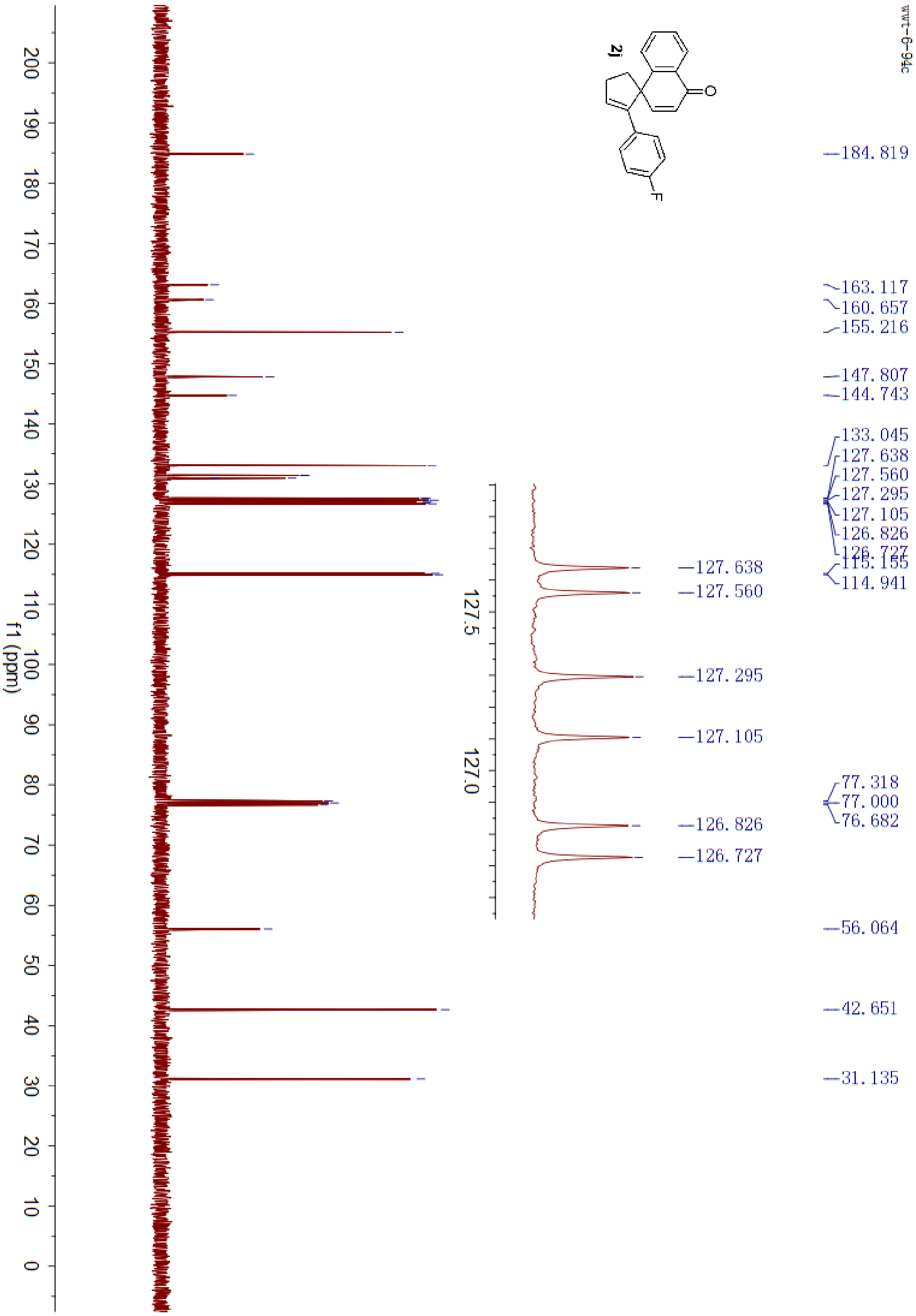
wwt-6-94c
2j
184.819
163.117
160.657
155.216
147.807
144.743
133.045
127.638
127.560
127.295
127.105
126.826
126.727
115.155
114.941
77.318
77.000
76.682
56.064
42.651
31.135
127.5
127.0
f1 (ppm)

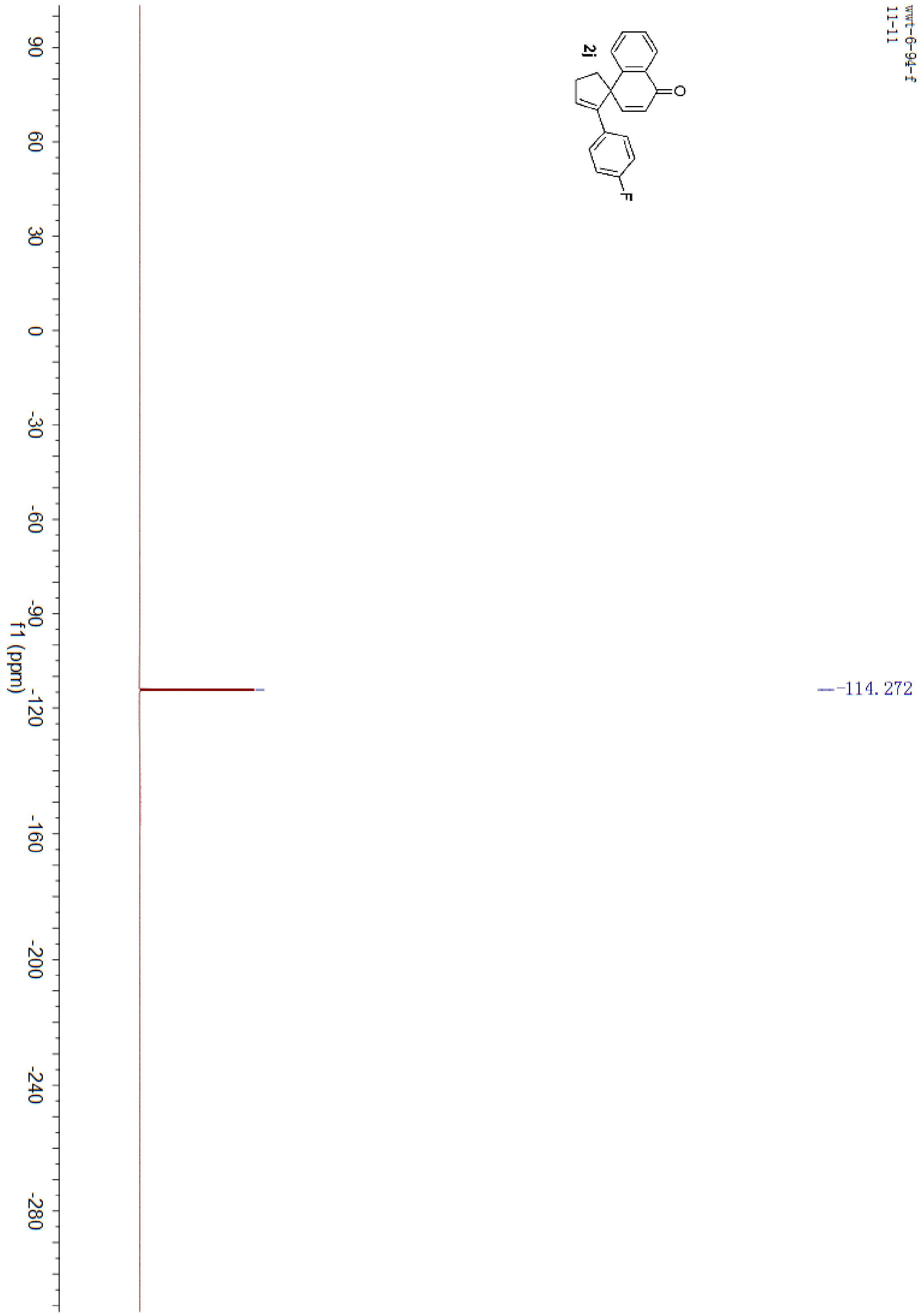
wwt-6-94-f
11-11
-114.272
2j
F
O
90
60
30
0
-30
-60
-90
-120
-160
-200
-240
-280
f1 (ppm)

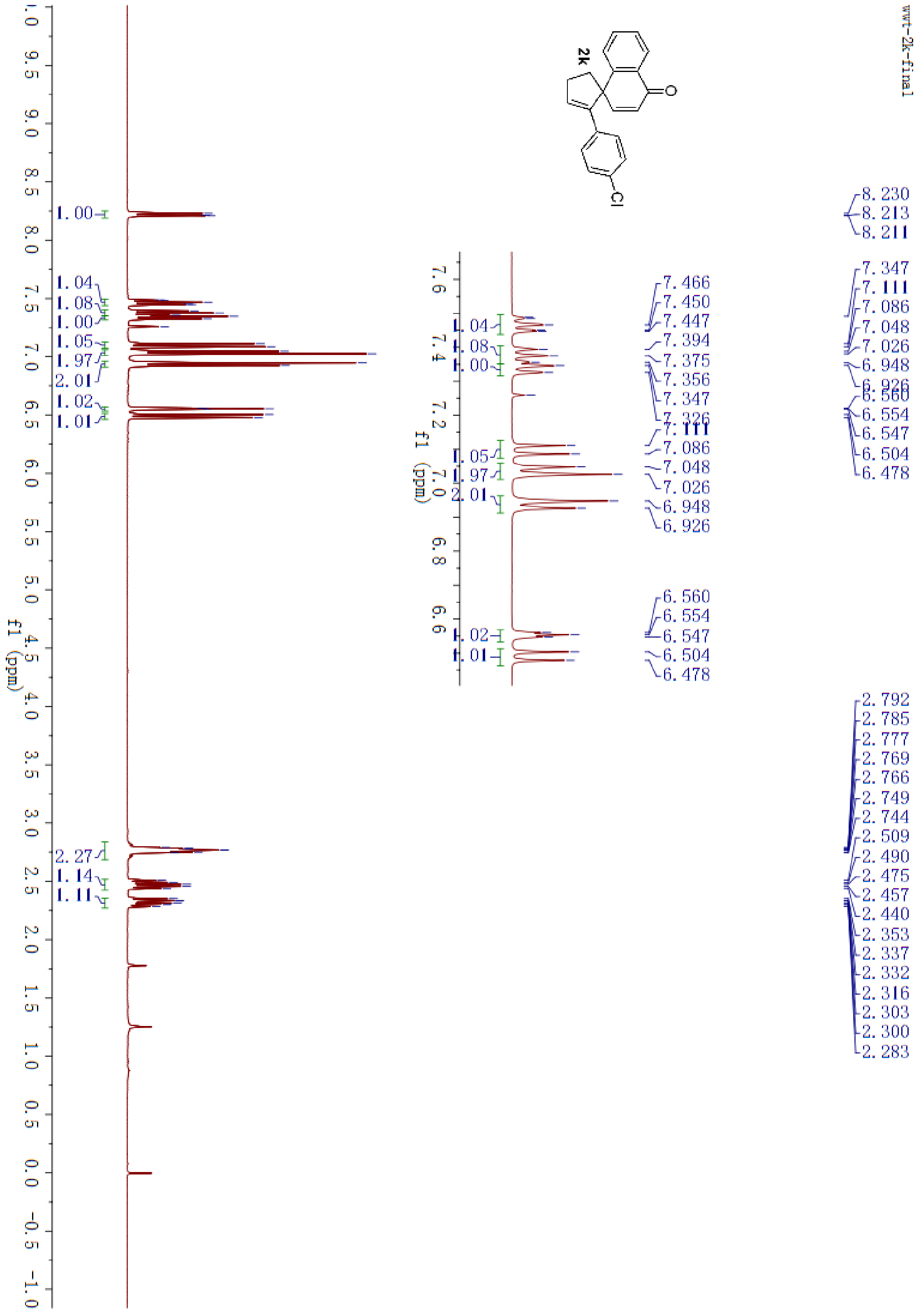
wwt-2k-final
2k
Cl
8.230
8.213
8.211
7.347
7.111
7.086
7.048
7.026
6.948
6.926
6.560
6.554
6.547
6.504
6.478
7.466
7.450
7.447
7.394
7.375
7.356
7.347
7.326
7.111
7.086
7.048
7.026
6.948
6.926
6.560
6.554
6.547
6.504
6.478
2.792
2.785
2.777
2.769
2.766
2.749
2.744
2.509
2.490
2.475
2.457
2.440
2.353
2.337
2.332
2.316
2.303
2.300
2.283
1.00
1.04
1.08
1.00
1.05
1.97
2.01
1.02
1.01
2.27
1.14
1.11
f1 (ppm)

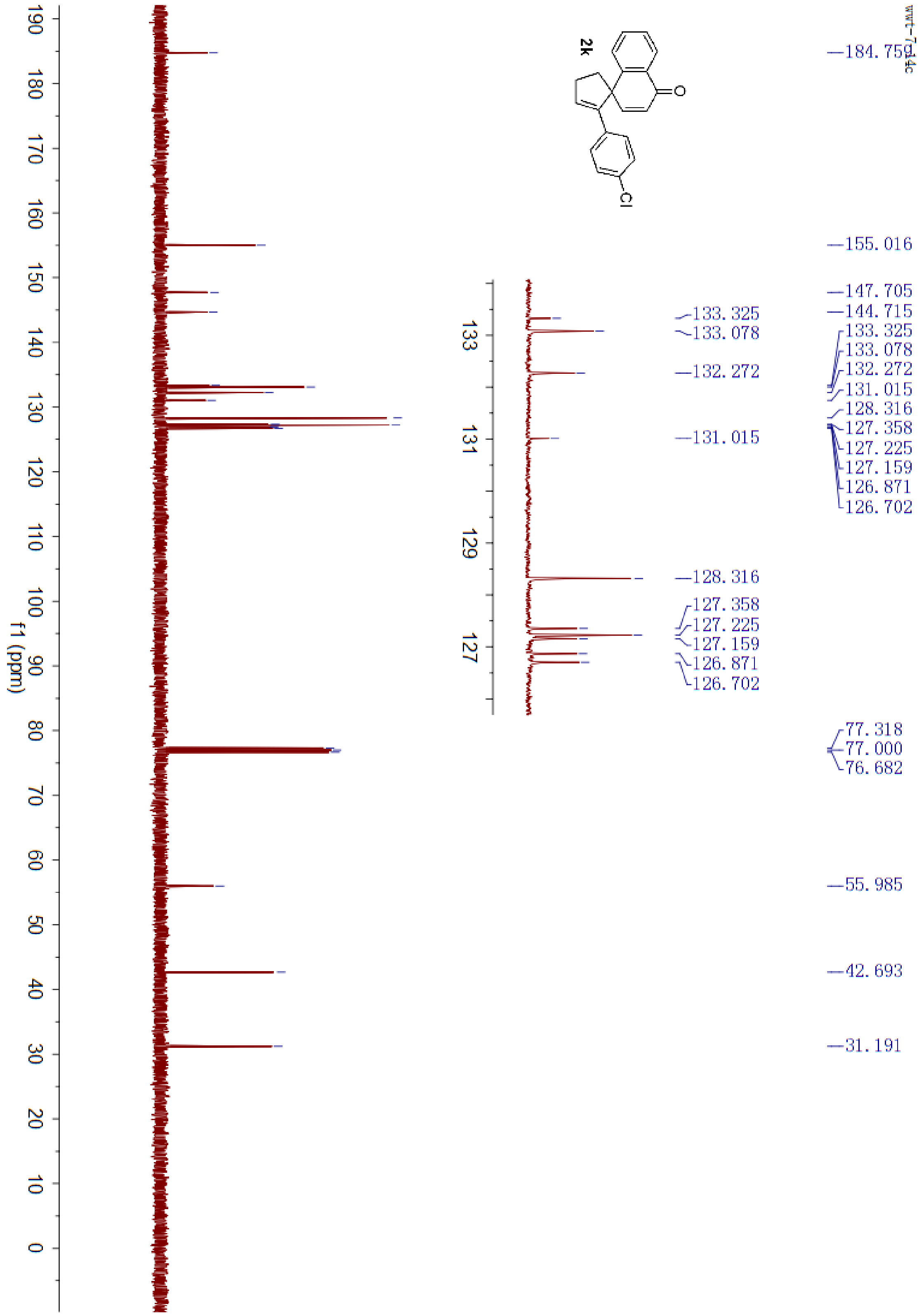
2k
184.759
155.016
147.705
144.715
133.325
133.078
132.272
131.015
128.316
127.358
127.225
127.159
126.871
126.702
77.318
77.000
76.682
55.985
42.693
31.191
f1 (ppm)

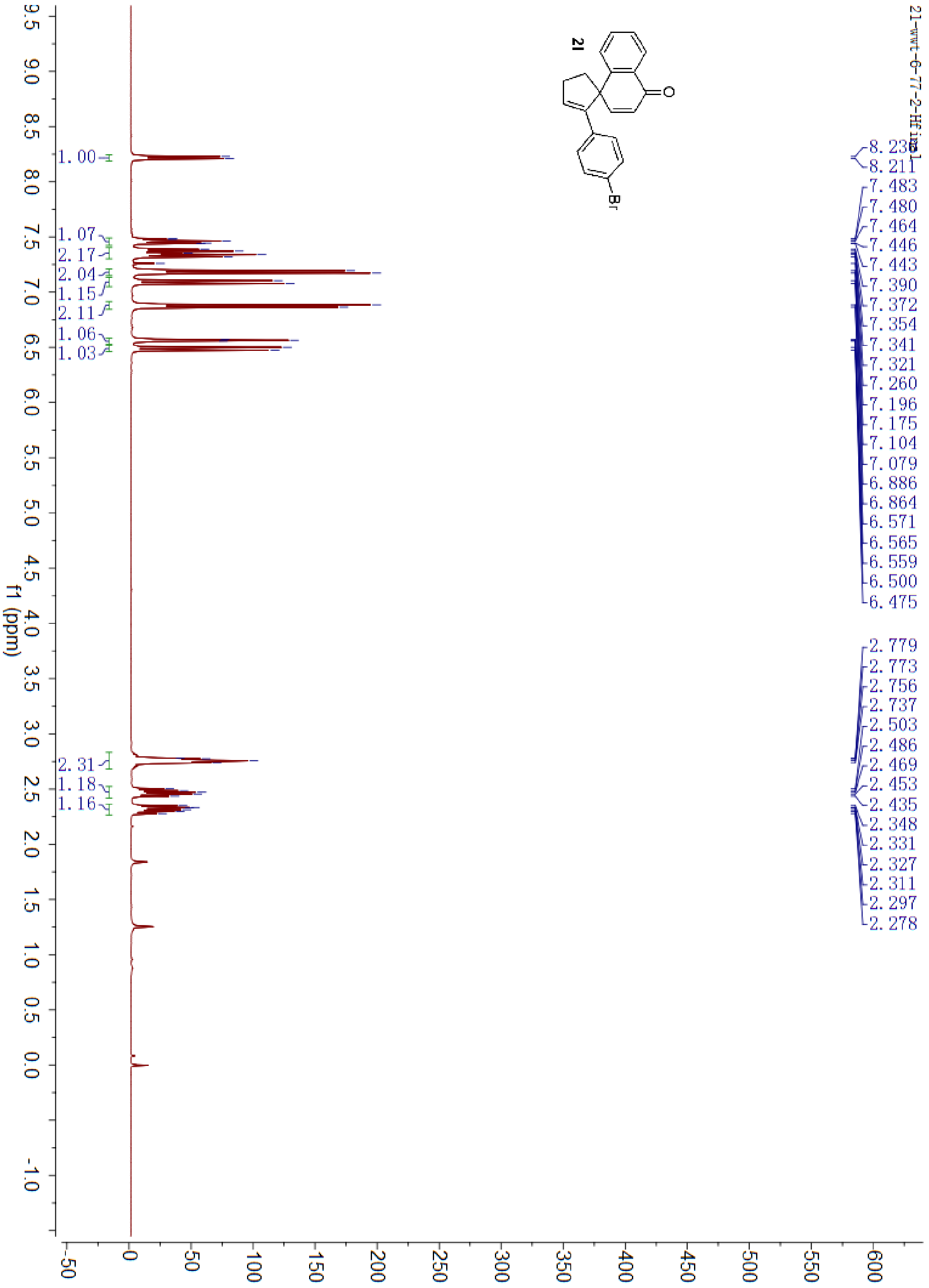
21-wwt-6-77-2-H
2l
Br
O
8.238
8.211
7.483
7.480
7.464
7.446
7.443
7.390
7.372
7.354
7.341
7.321
7.260
7.196
7.175
7.104
7.079
6.886
6.864
6.571
6.565
6.559
6.500
6.475
2.779
2.773
2.756
2.737
2.503
2.486
2.469
2.453
2.435
2.348
2.331
2.327
2.311
2.297
2.278
1.00
1.07
2.17
2.04
1.15
2.11
1.06
1.03
2.31
1.18
1.16
f1 (ppm)

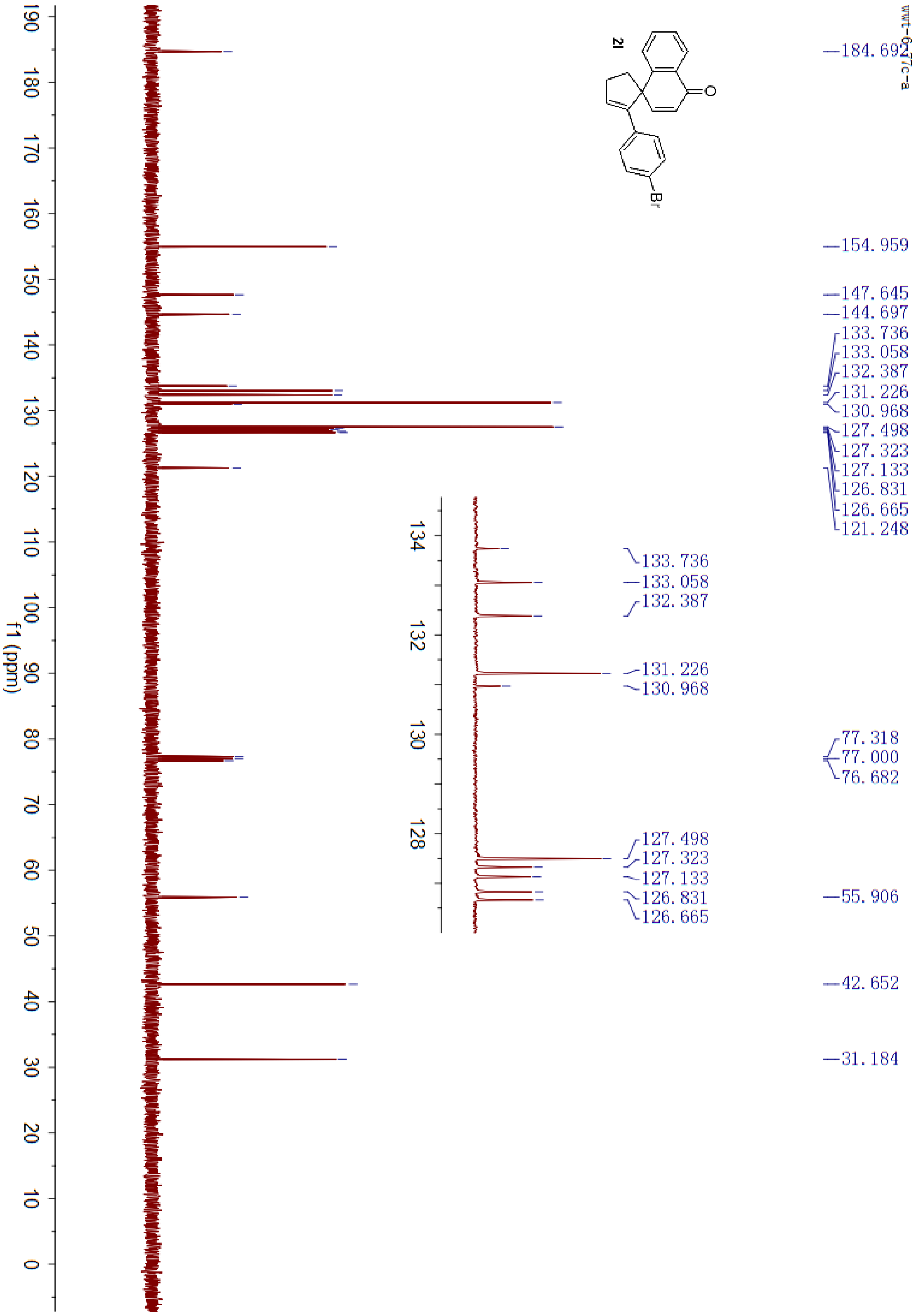
wwt-6-77c-a
2l
184.692
154.959
147.645
144.697
133.736
133.058
132.387
131.226
130.968
127.498
127.323
127.133
126.831
126.665
121.248
77.318
77.000
76.682
55.906
42.652
31.184
f1 (ppm)

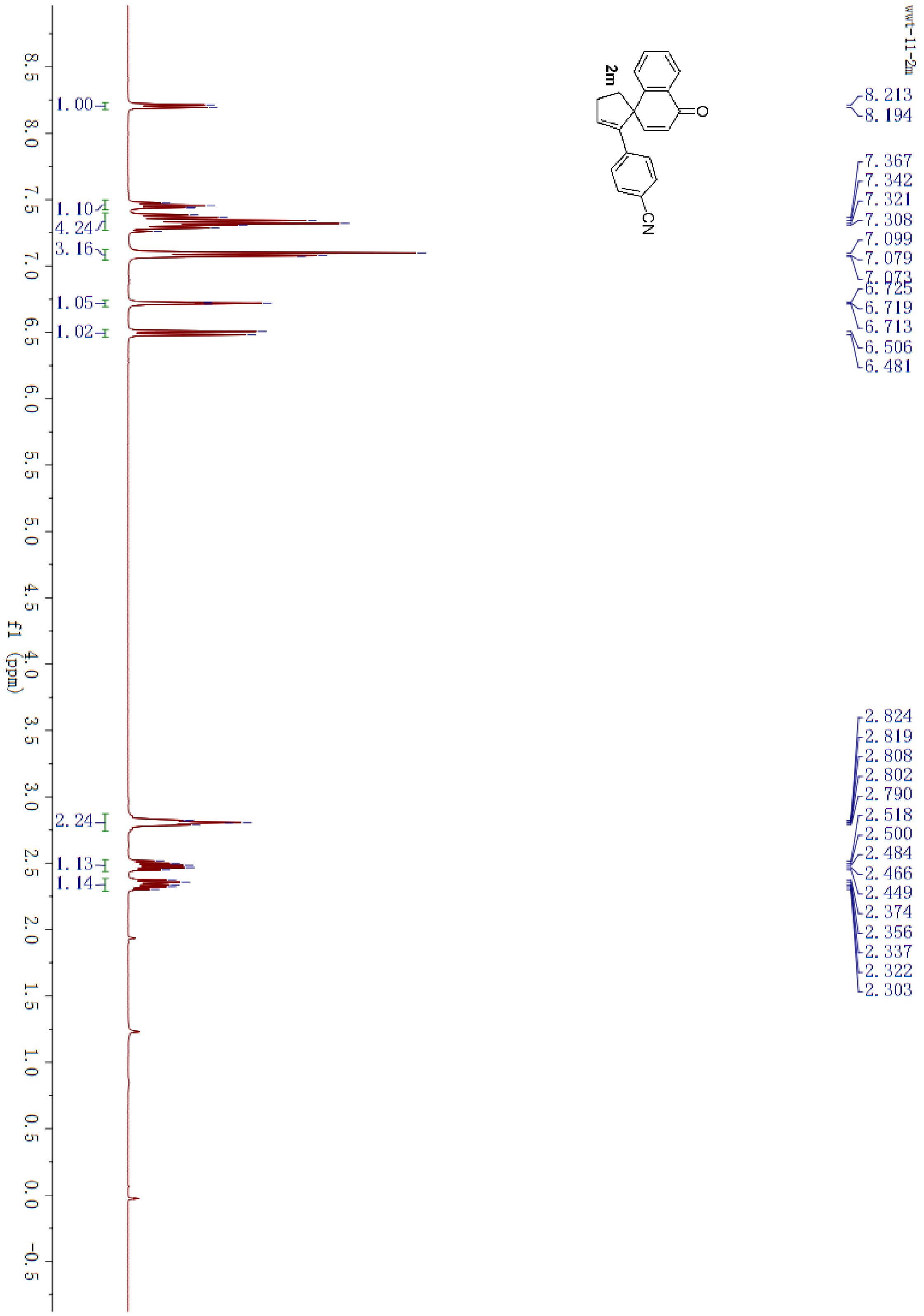
wwt-11-2m
8.213
8.194
7.367
7.342
7.321
7.308
7.099
7.079
7.073
6.725
6.719
6.713
6.506
6.481
2.824
2.819
2.808
2.802
2.790
2.518
2.500
2.484
2.466
2.449
2.374
2.356
2.337
2.322
2.303
2m
O
CN
1.00
1.10
4.24
3.16
1.05
1.02
2.24
1.13
1.14
8.5
8.0
7.5
7.0
6.5
6.0
5.5
5.0
4.5
4.0
3.5
3.0
2.5
2.0
1.5
1.0
0.5
0.0
-0.5
f1 (ppm)

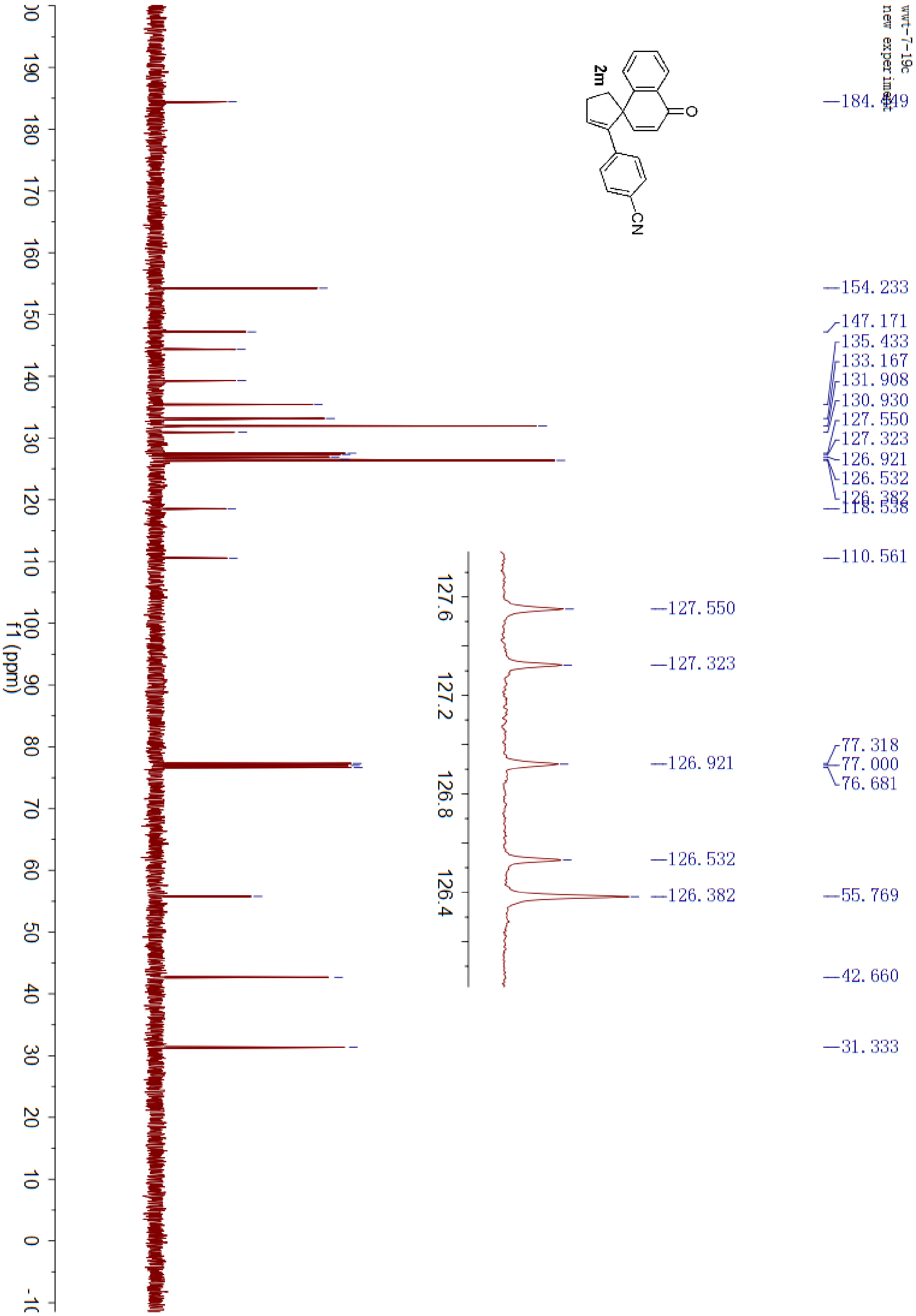
wwt-7-19c
new experiment
2m
CN
O
184.449
154.233
147.171
135.433
133.167
131.908
130.930
127.550
127.323
126.921
126.532
126.382
118.538
110.561
77.318
77.000
76.681
55.769
42.660
31.333
f1 (ppm)

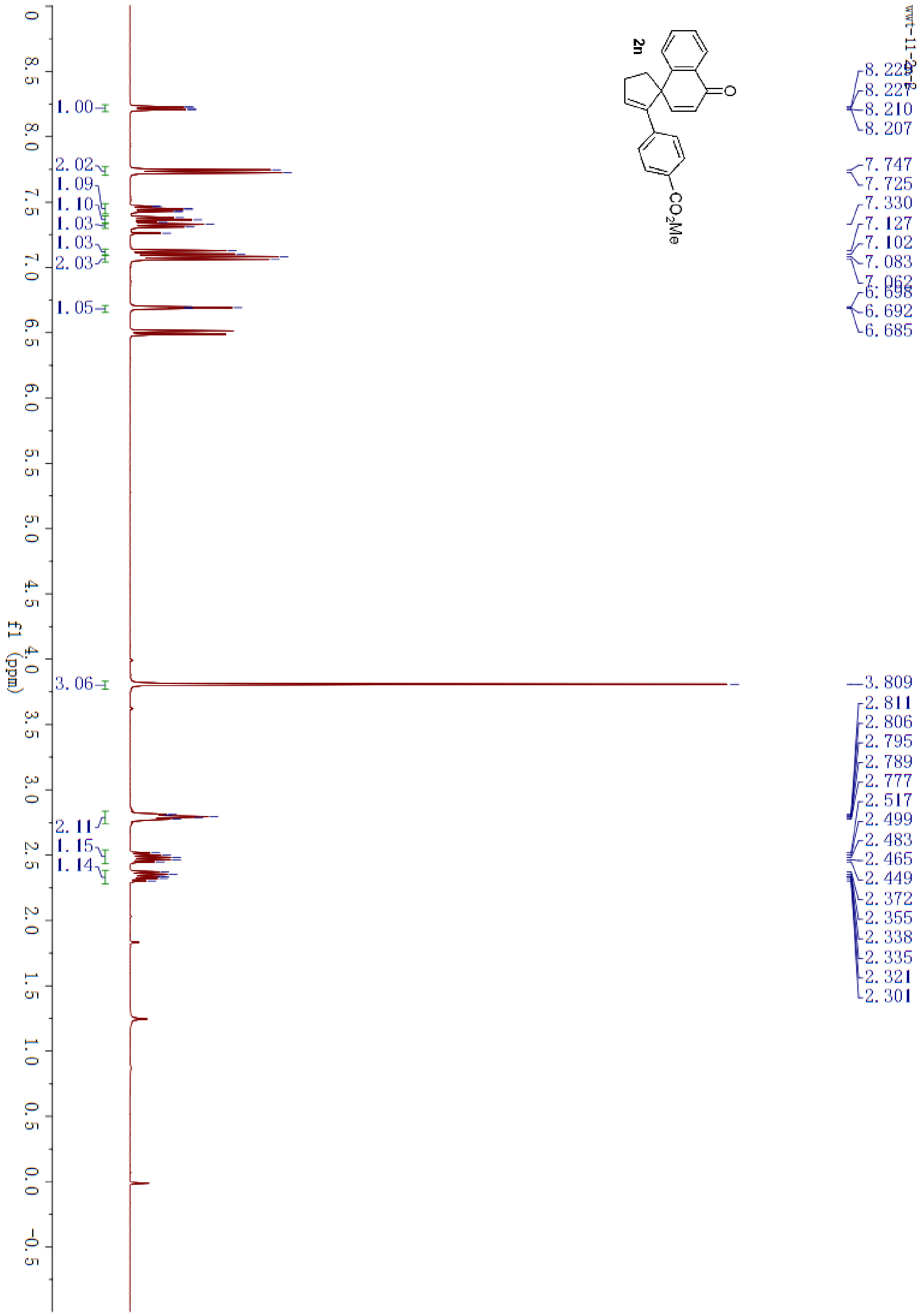

wwt-11-2n-2
2n
CO_2Me
8.229
8.227
8.210
8.207
7.747
7.725
7.330
7.127
7.102
7.083
7.062
6.698
6.692
6.685
3.809
2.811
2.806
2.795
2.789
2.777
2.517
2.499
2.483
2.465
2.449
2.372
2.355
2.338
2.335
2.321
2.301
1.00
2.02
1.09
1.10
1.03
1.03
2.03
1.05
3.06
2.11
1.15
1.14
f1 (ppm)

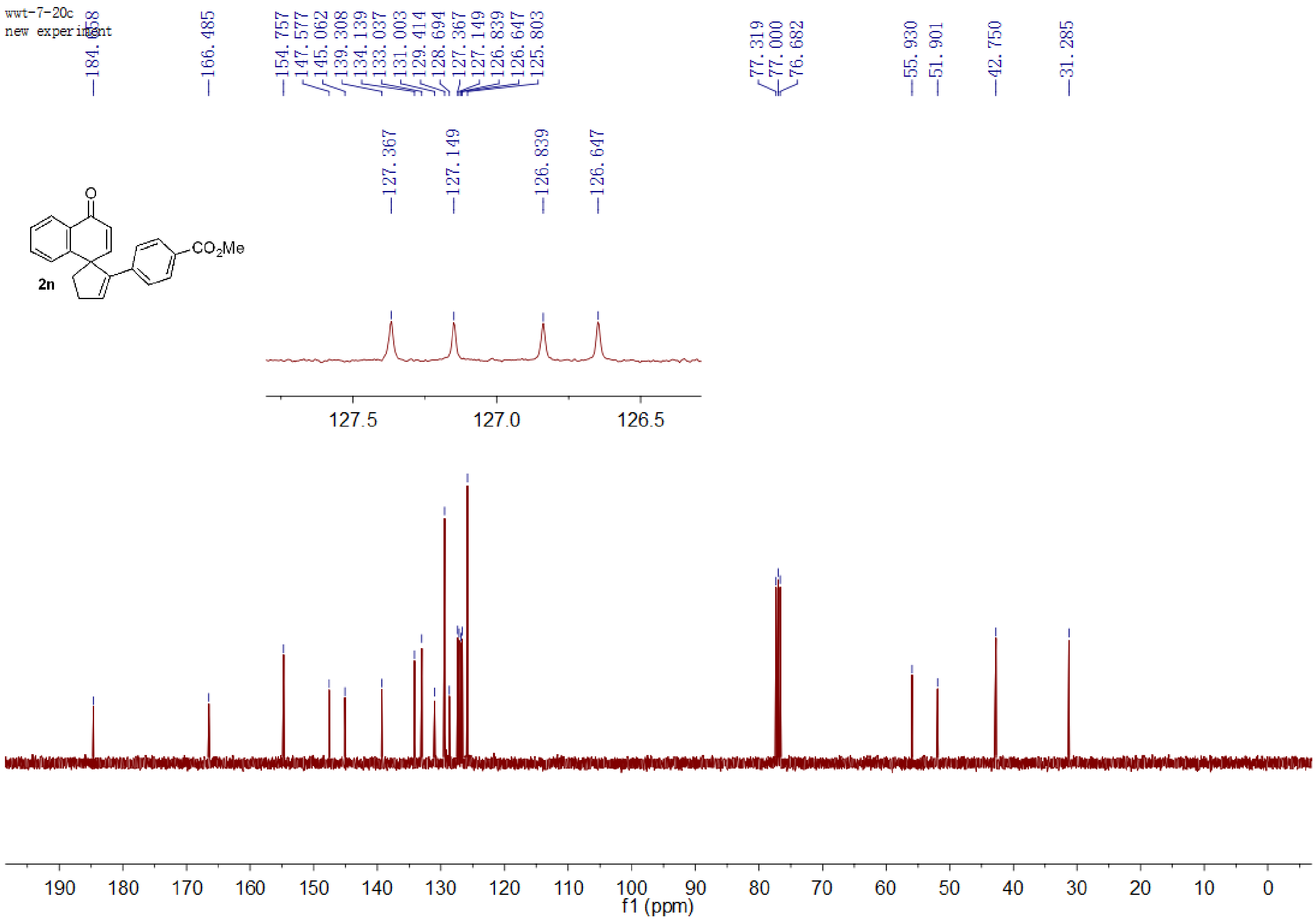
wwt-7-20c
166.485
154.757
147.577
145.062
139.308
134.139
133.037
131.003
129.414
128.694
127.367
127.149
126.839
126.647
125.803
77.319
77.000
76.682
55.930
51.901
42.750
31.285
127.367
127.149
126.839
126.647
CO2Me
2n
127.5
127.0
126.5
190
180
170
160
150
140
130
120
110
100
90
80
70
60
50
40
30
20
10
0
f1 (ppm)

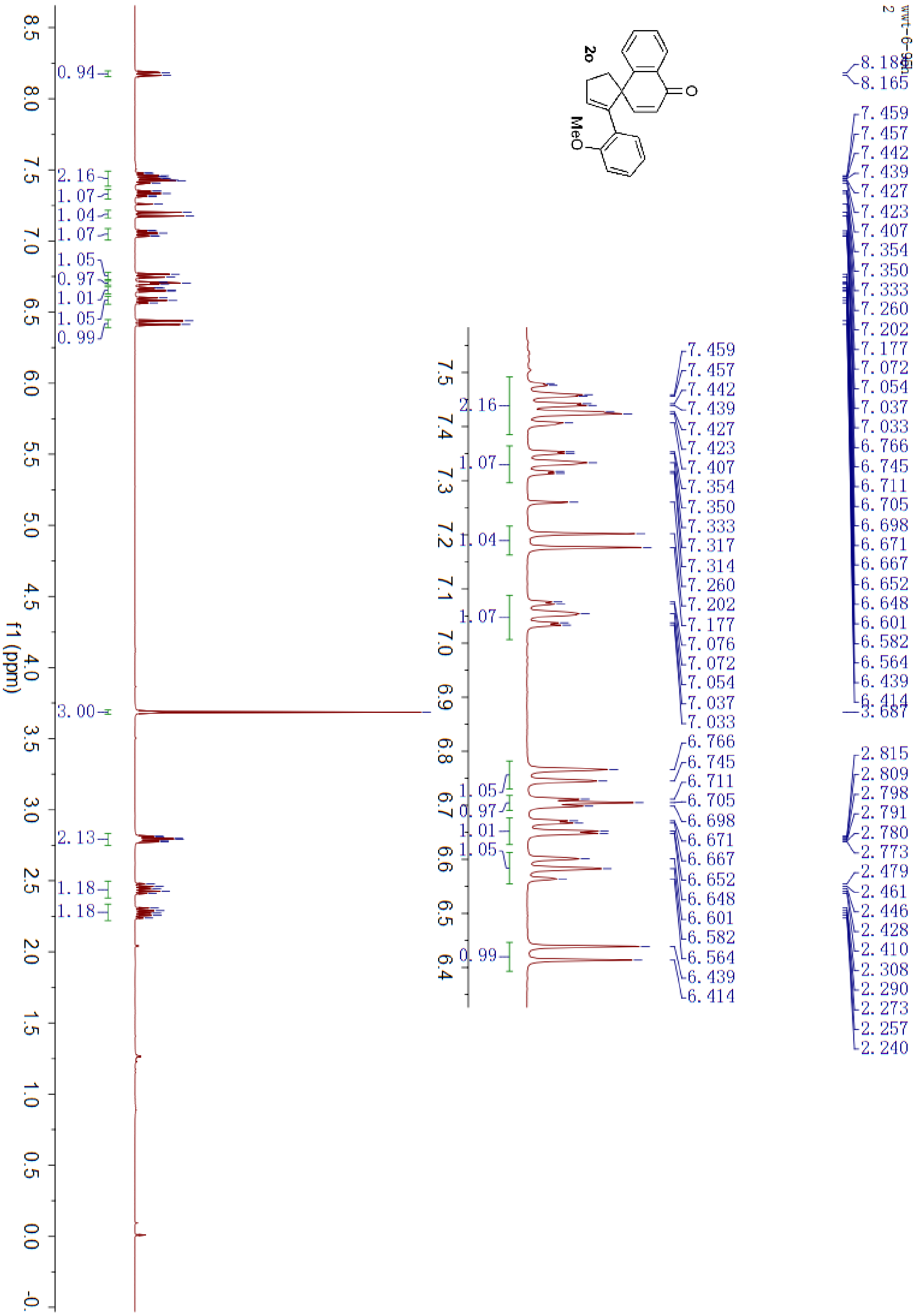

2o
MeO
O
f1 (ppm)
8.165
7.459
7.457
7.442
7.439
7.427
7.423
7.407
7.354
7.350
7.333
7.317
7.314
7.260
7.202
7.177
7.076
7.072
7.054
7.037
7.033
6.766
6.745
6.711
6.705
6.698
6.671
6.667
6.652
6.648
6.601
6.582
6.564
6.439
6.414
3.687
2.815
2.809
2.798
2.791
2.780
2.773
2.479
2.461
2.446
2.428
2.410
2.308
2.290
2.273
2.257
2.240

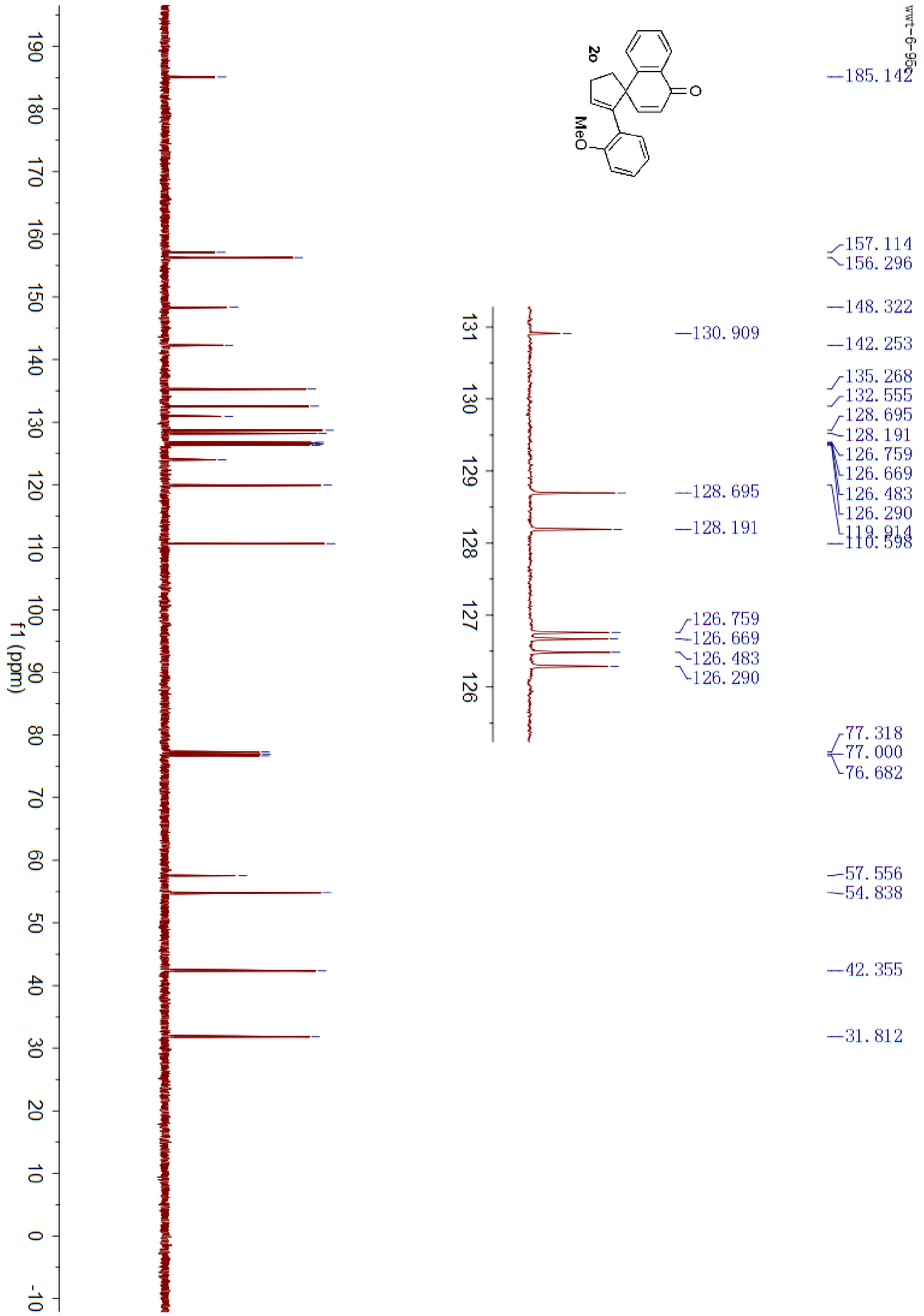

2o
MeO
wwt-6-95
185.142
157.114
156.296
148.322
142.253
135.268
132.555
128.695
128.191
126.759
126.669
126.483
126.290
119.914
110.598
77.318
77.000
76.682
57.556
54.838
42.355
31.812
130.909
128.695
128.191
126.759
126.669
126.483
126.290
f1 (ppm)

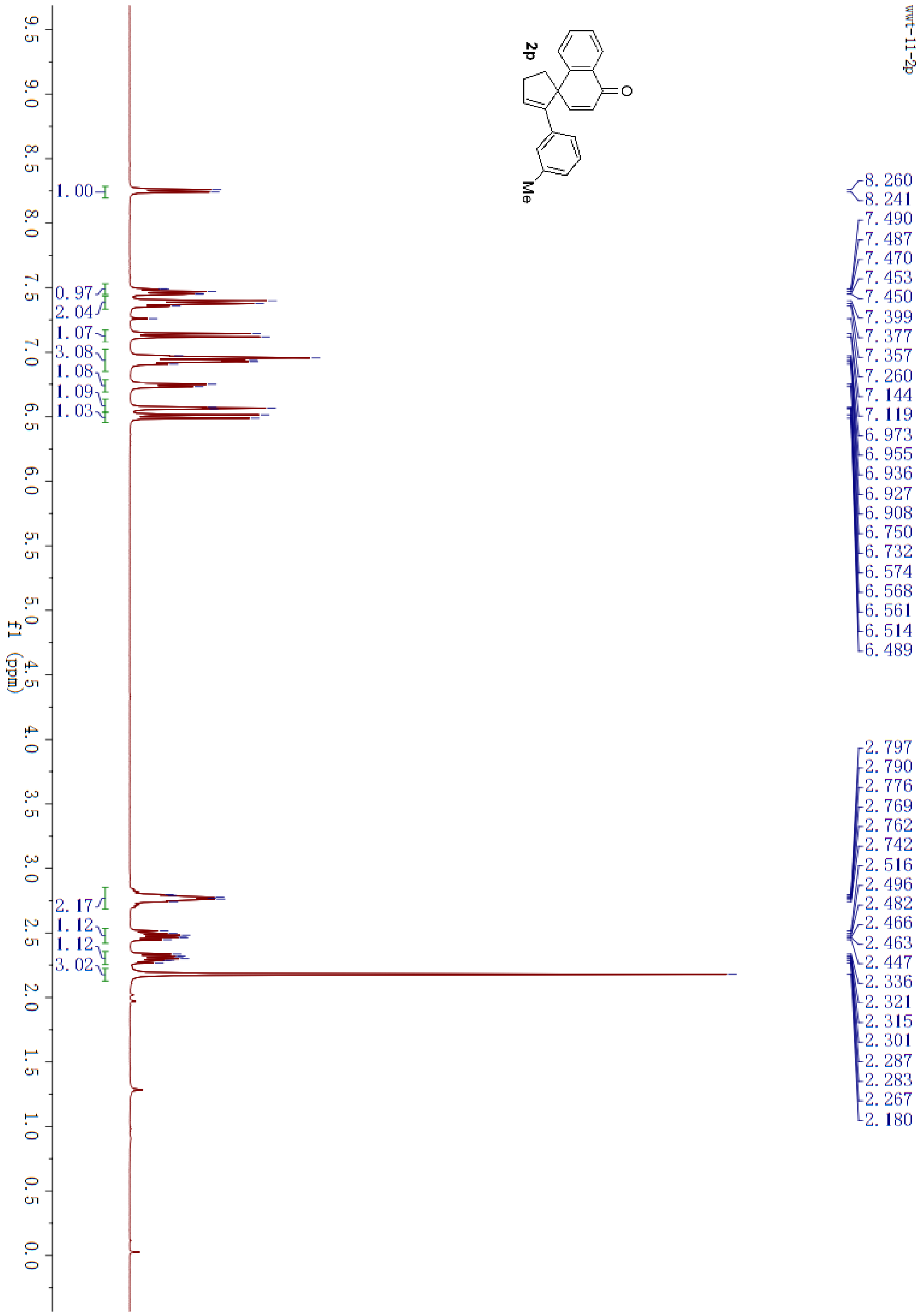
wwt-11-2p
2p
Me
O
8.260
8.241
7.490
7.487
7.470
7.453
7.450
7.399
7.377
7.357
7.260
7.144
7.119
6.973
6.955
6.936
6.927
6.908
6.750
6.732
6.574
6.568
6.561
6.514
6.489
2.797
2.790
2.776
2.769
2.762
2.742
2.516
2.496
2.482
2.466
2.463
2.447
2.336
2.321
2.315
2.301
2.287
2.283
2.267
2.180
1.00
0.97
2.04
1.07
3.08
1.08
1.09
1.03
2.17
1.12
1.12
3.02
9.5
9.0
8.5
8.0
7.5
7.0
6.5
6.0
5.5
5.0
4.5
4.0
3.5
3.0
2.5
2.0
1.5
1.0
0.5
0.0
f1 (ppm)

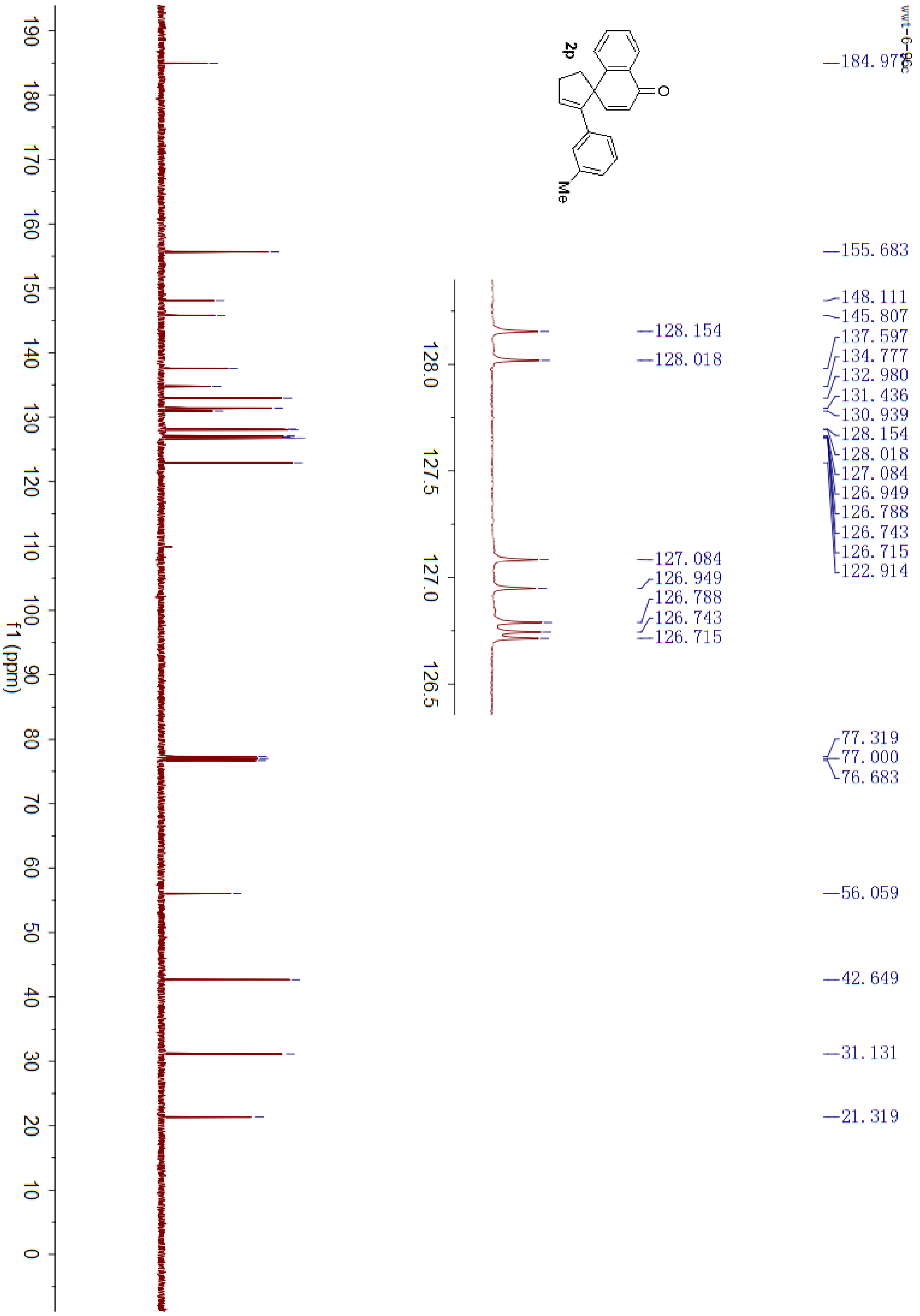

2p
Me
O
184.97
155.683
148.111
145.807
137.597
134.777
132.980
131.436
130.939
128.154
128.018
127.084
126.949
126.788
126.743
126.715
122.914
77.319
77.000
76.683
56.059
42.649
31.131
21.319
f1 (ppm)

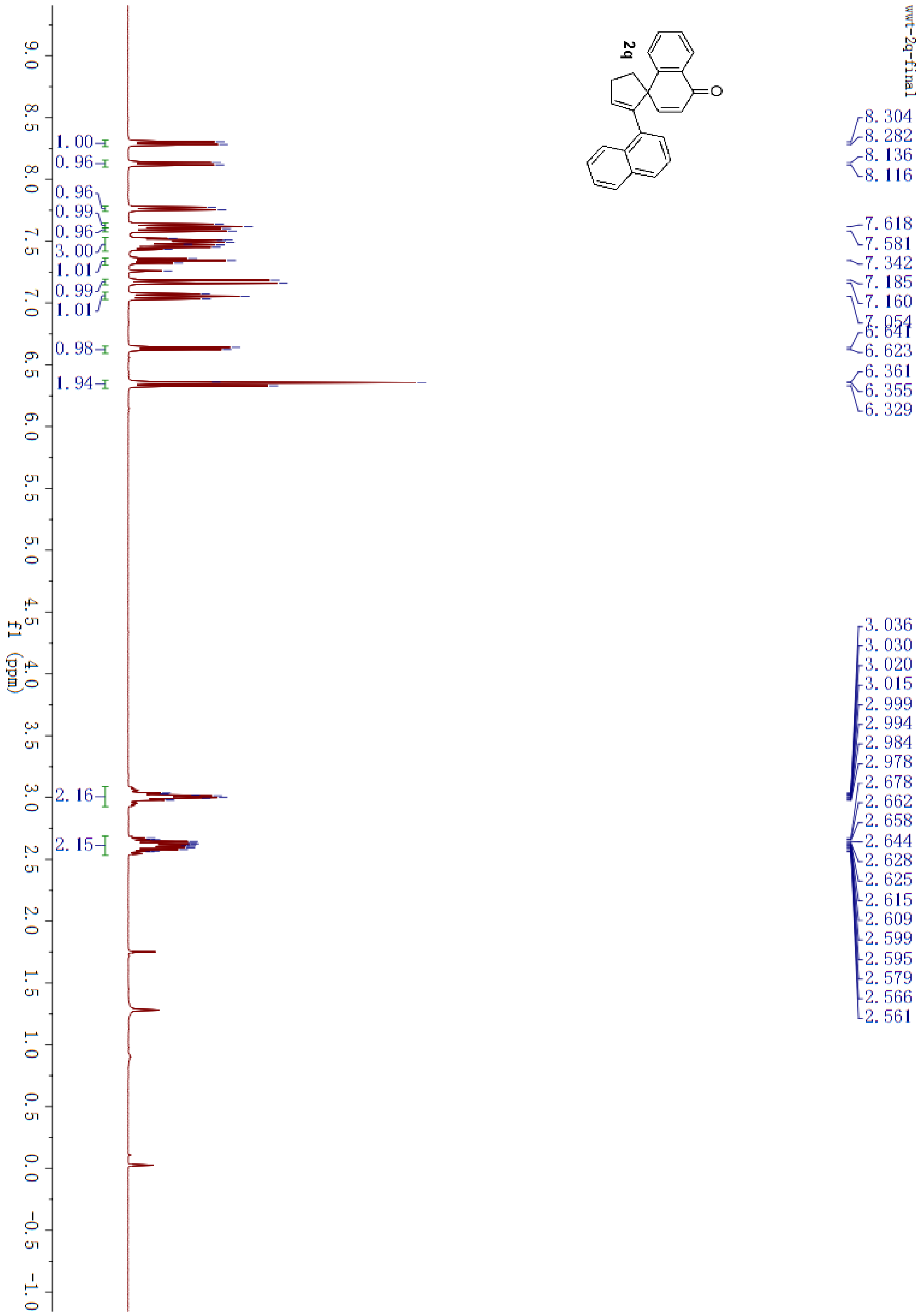
wwt-2q-final
2q
8.304
8.282
8.136
8.116
7.618
7.581
7.342
7.185
7.160
7.054
6.641
6.623
6.361
6.355
6.329
3.036
3.030
3.020
3.015
2.999
2.994
2.984
2.978
2.678
2.662
2.658
2.644
2.628
2.625
2.615
2.609
2.599
2.595
2.579
2.566
2.561
1.00
0.96
0.96
0.99
0.96
3.00
1.01
0.99
1.01
0.98
1.94
2.16
2.15
f1 (ppm)

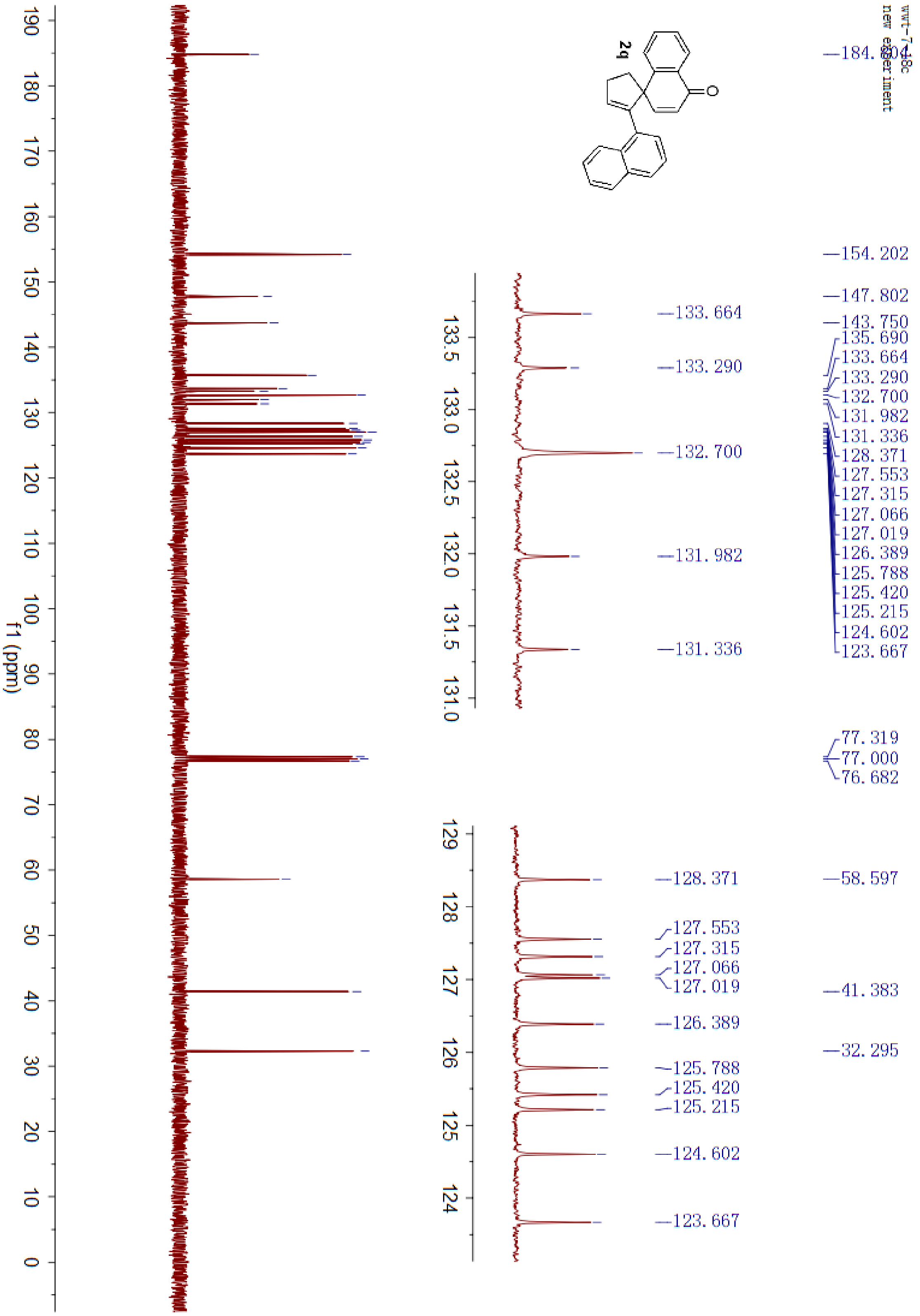

new experiment
184.804
154.202
147.802
143.750
135.690
133.664
133.290
132.700
131.982
131.336
128.371
127.553
127.315
127.066
127.019
126.389
125.788
125.420
125.215
124.602
123.667
77.319
77.000
76.682
58.597
41.383
32.295
2q
f1 (ppm)

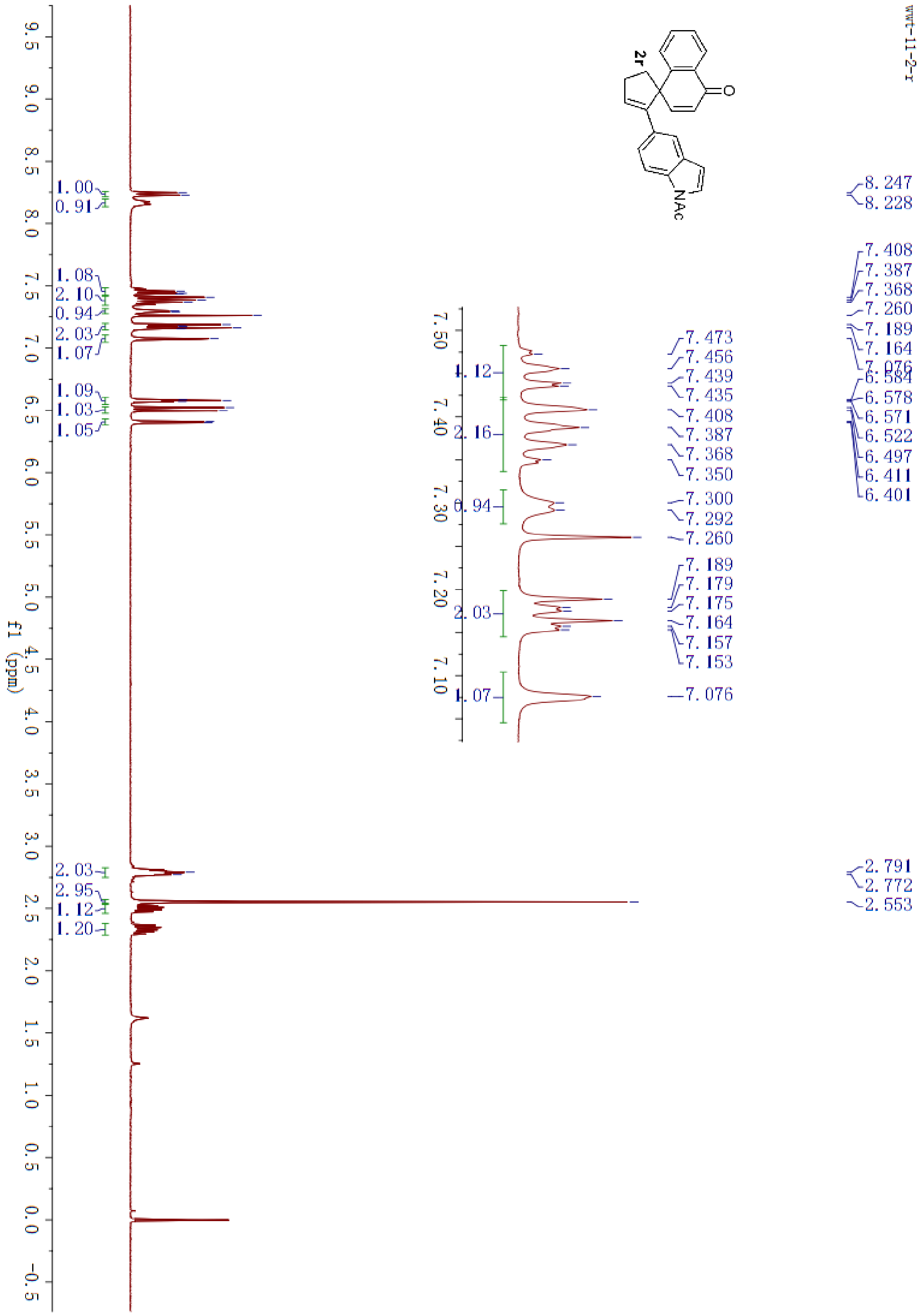
wwt-11-2-r
2r
NAc
8.247
8.228
7.408
7.387
7.368
7.260
7.189
7.164
7.076
6.584
6.578
6.571
6.522
6.497
6.411
6.401
2.791
2.772
2.553
7.473
7.456
7.439
7.435
7.408
7.387
7.368
7.350
7.300
7.292
7.260
7.189
7.179
7.175
7.164
7.157
7.153
7.076
1.00
0.91
1.08
2.10
0.94
2.03
1.07
1.09
1.03
1.05
2.03
2.95
1.12
1.20
4.12
2.16
0.94
2.03
1.07
f1 (ppm)

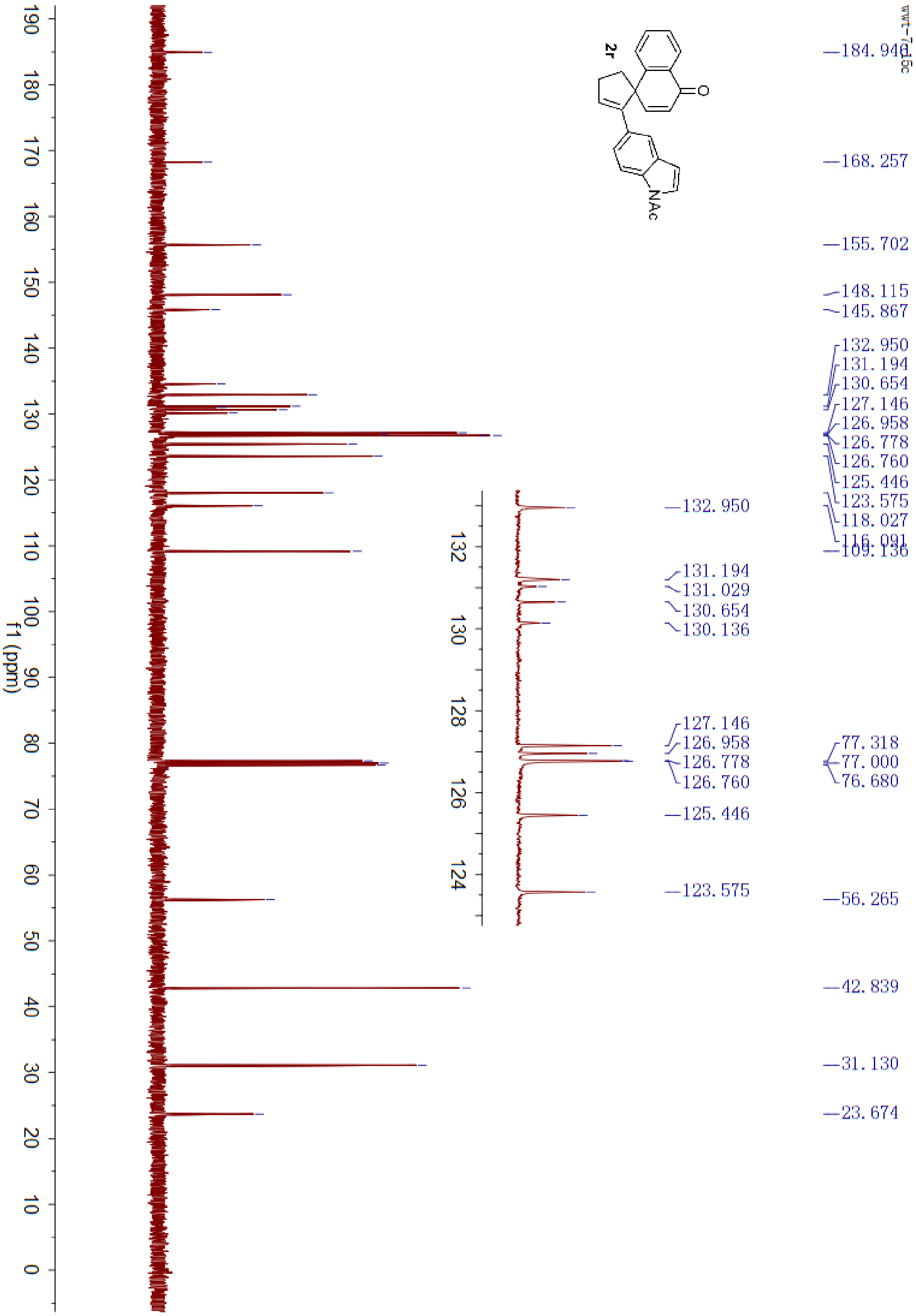
2r
NAc
O
184.940
168.257
155.702
148.115
145.867
132.950
131.194
130.654
127.146
126.958
126.778
126.760
125.446
123.575
118.027
116.091
109.136
77.318
77.000
76.680
56.265
42.839
31.130
23.674
132.950
131.194
131.029
130.654
130.136
127.146
126.958
126.778
126.760
125.446
123.575
f1 (ppm)

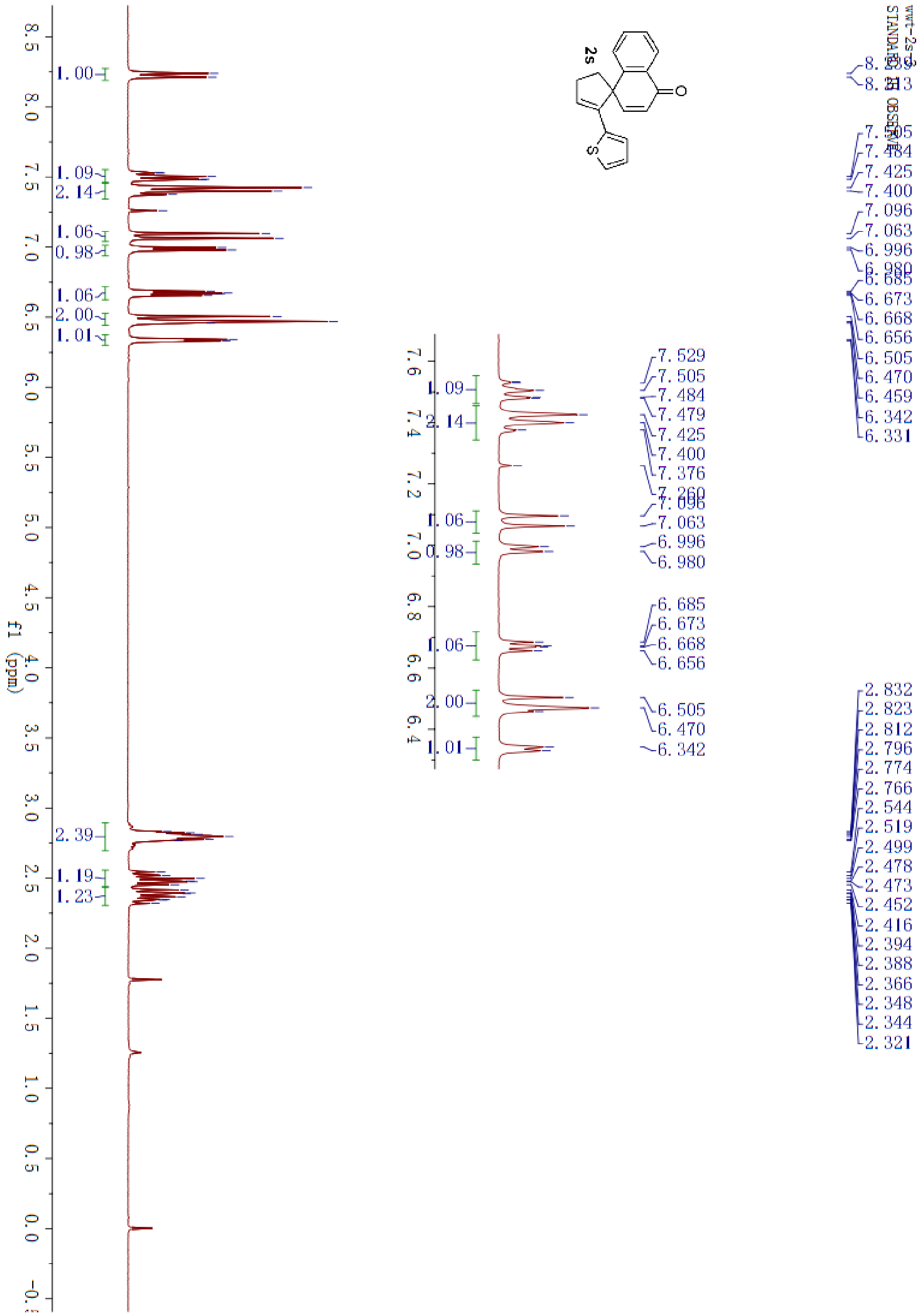

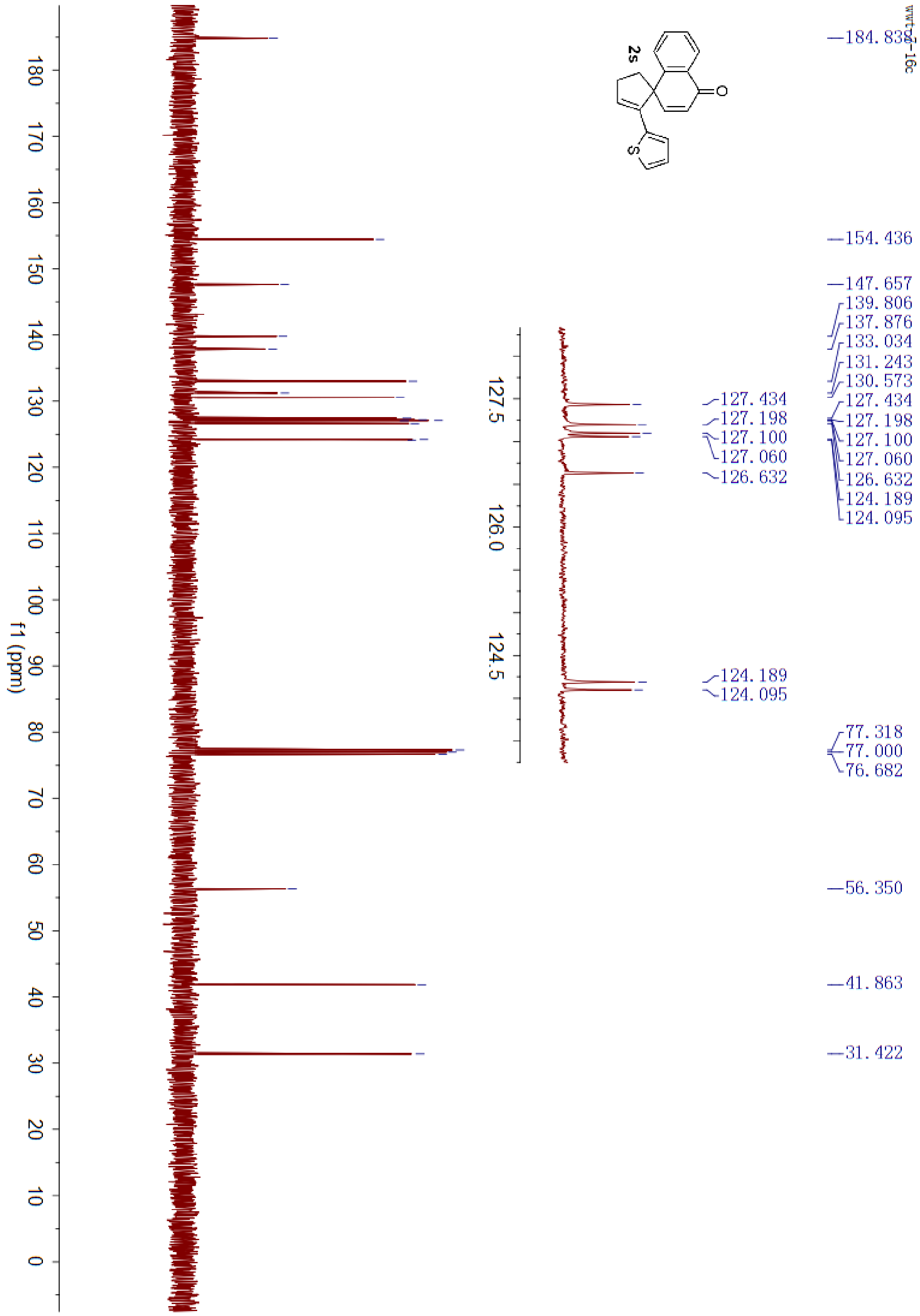

2s
184.838
154.436
147.657
139.806
137.876
133.034
131.243
130.573
127.434
127.198
127.100
127.060
126.632
124.189
124.095
77.318
77.000
76.682
56.350
41.863
31.422
127.5
126.0
124.5
f1 (ppm)

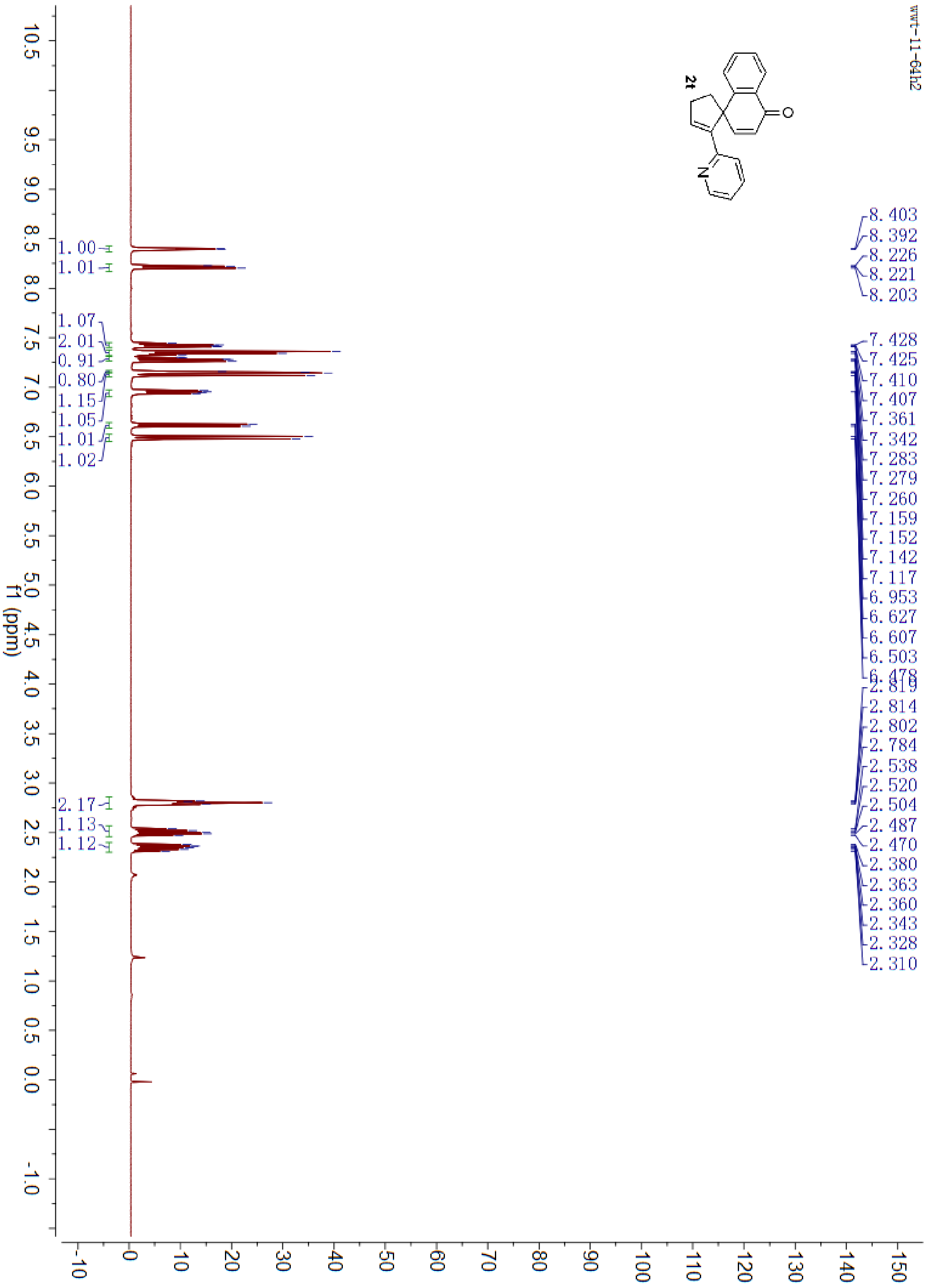

wwt-11-64h2
2t
8.403
8.392
8.226
8.221
8.203
7.428
7.425
7.410
7.407
7.361
7.342
7.283
7.279
7.260
7.159
7.152
7.142
7.117
6.953
6.627
6.607
6.503
6.478
2.819
2.814
2.802
2.784
2.538
2.520
2.504
2.487
2.470
2.380
2.363
2.360
2.343
2.328
2.310
1.00
1.01
1.07
2.01
0.91
0.80
1.15
1.05
1.01
1.02
2.17
1.13
1.12
f1 (ppm)

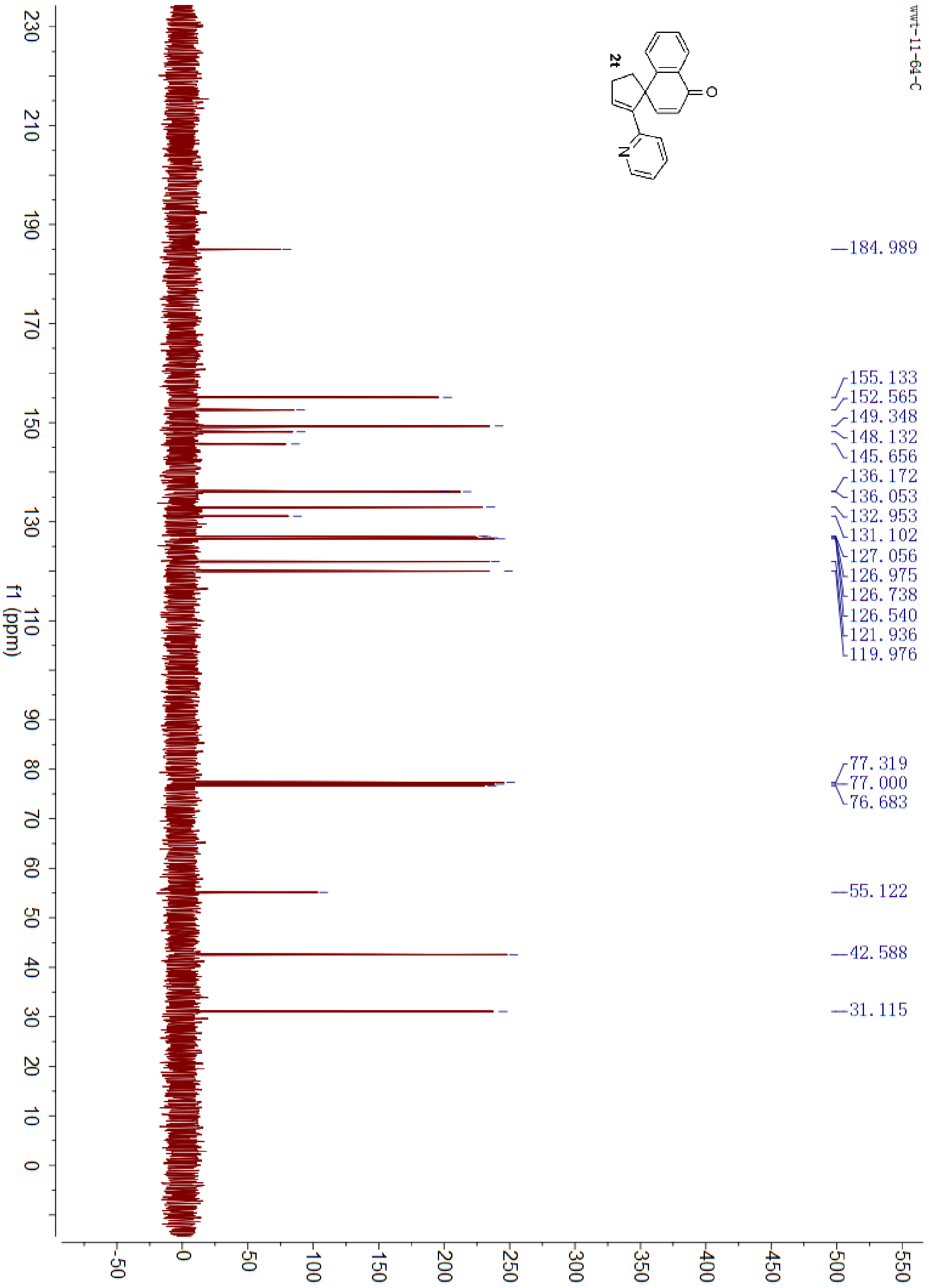
wwt-11-64-C
2t
184.989
155.133
152.565
149.348
148.132
145.656
136.172
136.053
132.953
131.102
127.056
126.975
126.738
126.540
121.936
119.976
77.319
77.000
76.683
55.122
42.588
31.115
f1 (ppm)

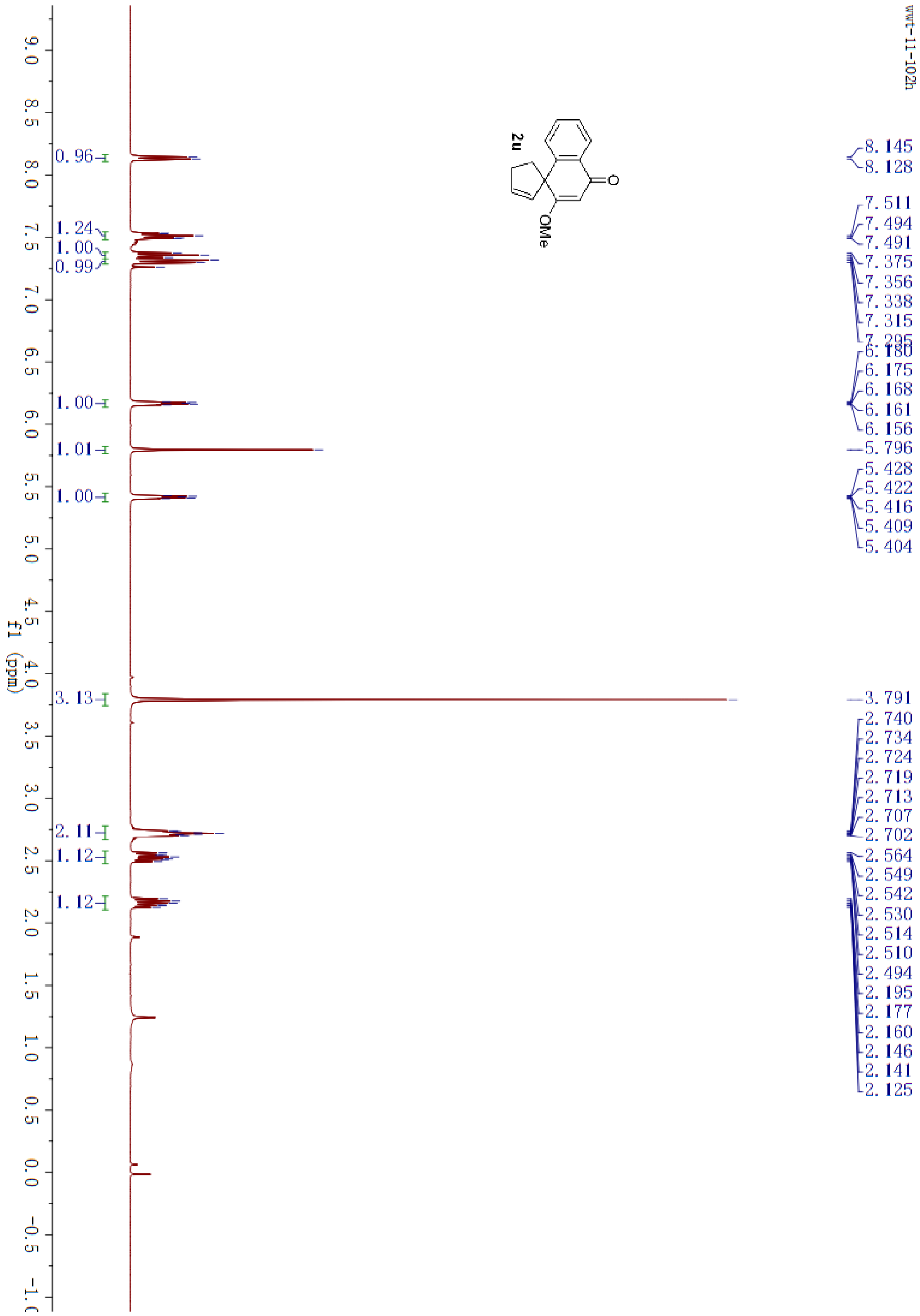

wwt-11-102h
2u
OMe
O
8.145
8.128
7.511
7.494
7.491
7.375
7.356
7.338
7.315
7.295
6.180
6.175
6.168
6.161
6.156
5.796
5.428
5.422
5.416
5.409
5.404
3.791
2.740
2.734
2.724
2.719
2.713
2.707
2.702
2.564
2.549
2.542
2.530
2.514
2.510
2.494
2.195
2.177
2.160
2.146
2.141
2.125
0.96
1.24
1.00
0.99
1.00
1.01
1.00
3.13
2.11
1.12
1.12
f1 (ppm)

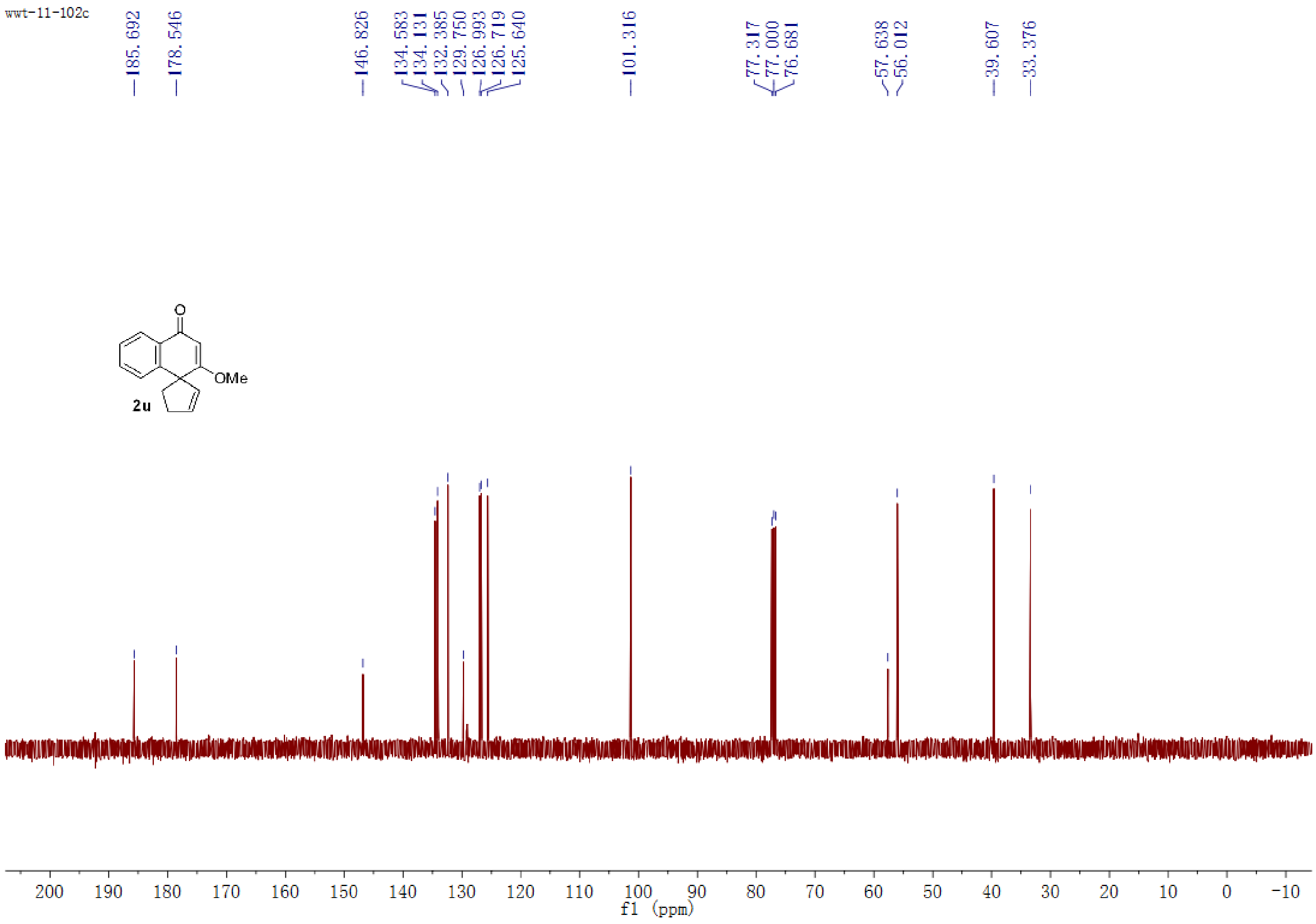
wwt-11-102c
185.692
178.546
146.826
134.583
134.131
132.385
129.750
126.993
126.719
125.640
101.316
77.317
77.000
76.681
57.638
56.012
39.607
33.376
OMe
2u
f1 (ppm)
200 190 180 170 160 150 140 130 120 110 100 90 80 70 60 50 40 30 20 10 0 -10

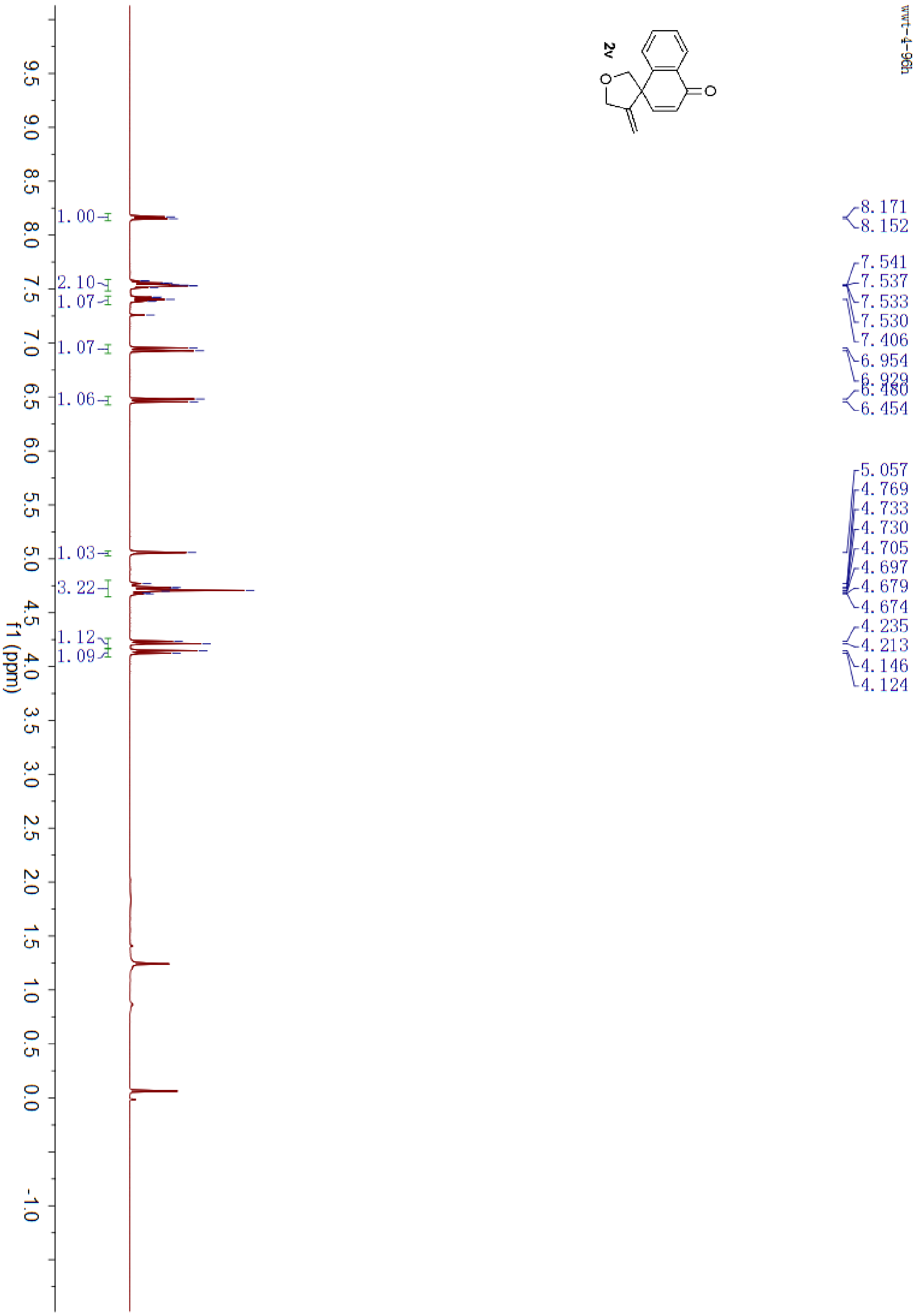
wwt-4-96h
2v
O
O
8.171
8.152
7.541
7.537
7.533
7.530
7.406
6.954
6.929
6.480
6.454
5.057
4.769
4.733
4.730
4.705
4.697
4.679
4.674
4.235
4.213
4.146
4.124
1.00
2.10
1.07
1.07
1.06
1.03
3.22
1.12
1.09
f1 (ppm)

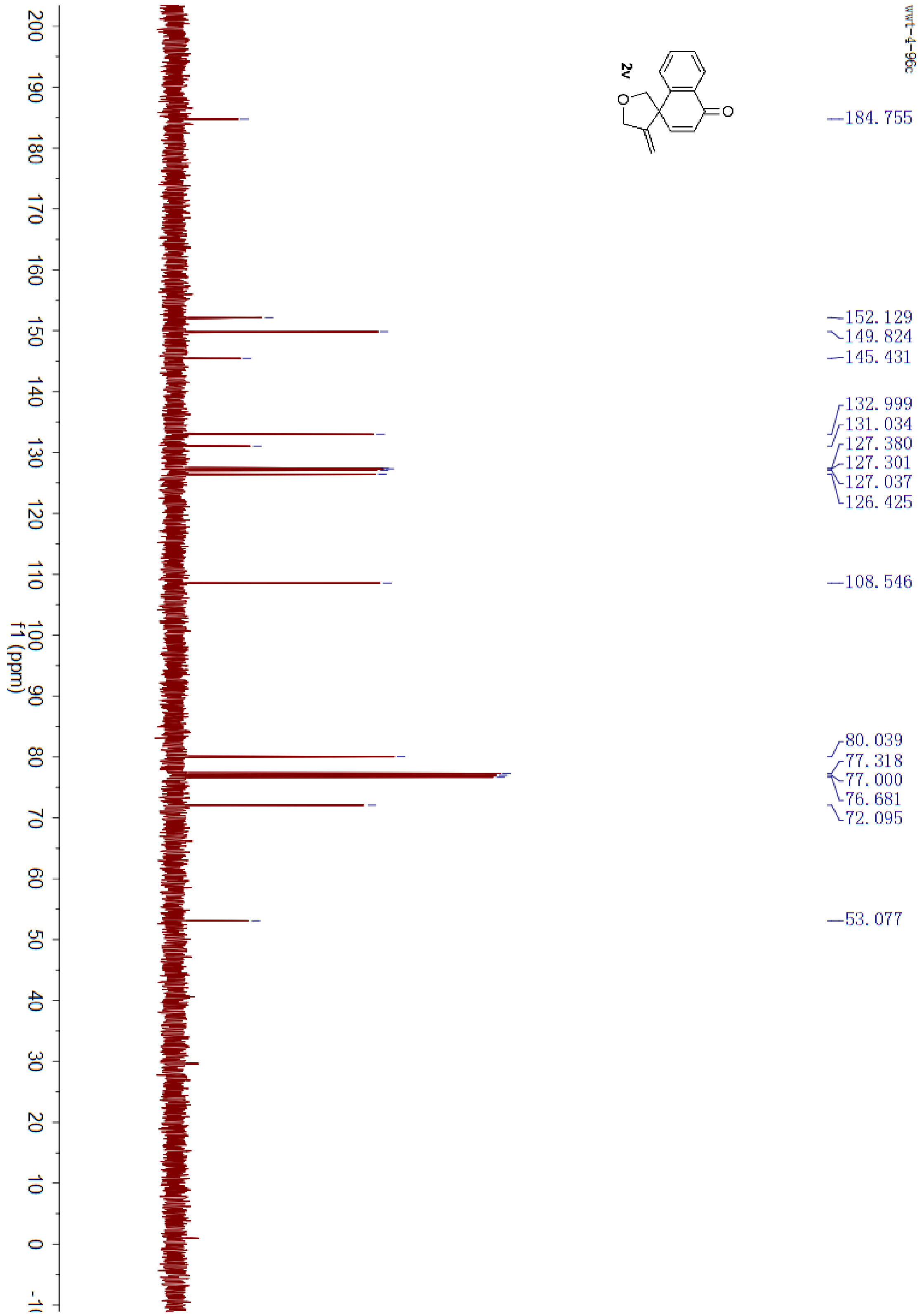
wwt-4-96c
2v
184.755
152.129
149.824
145.431
132.999
131.034
127.380
127.301
127.037
126.425
108.546
80.039
77.318
77.000
76.681
72.095
53.077
f1 (ppm)

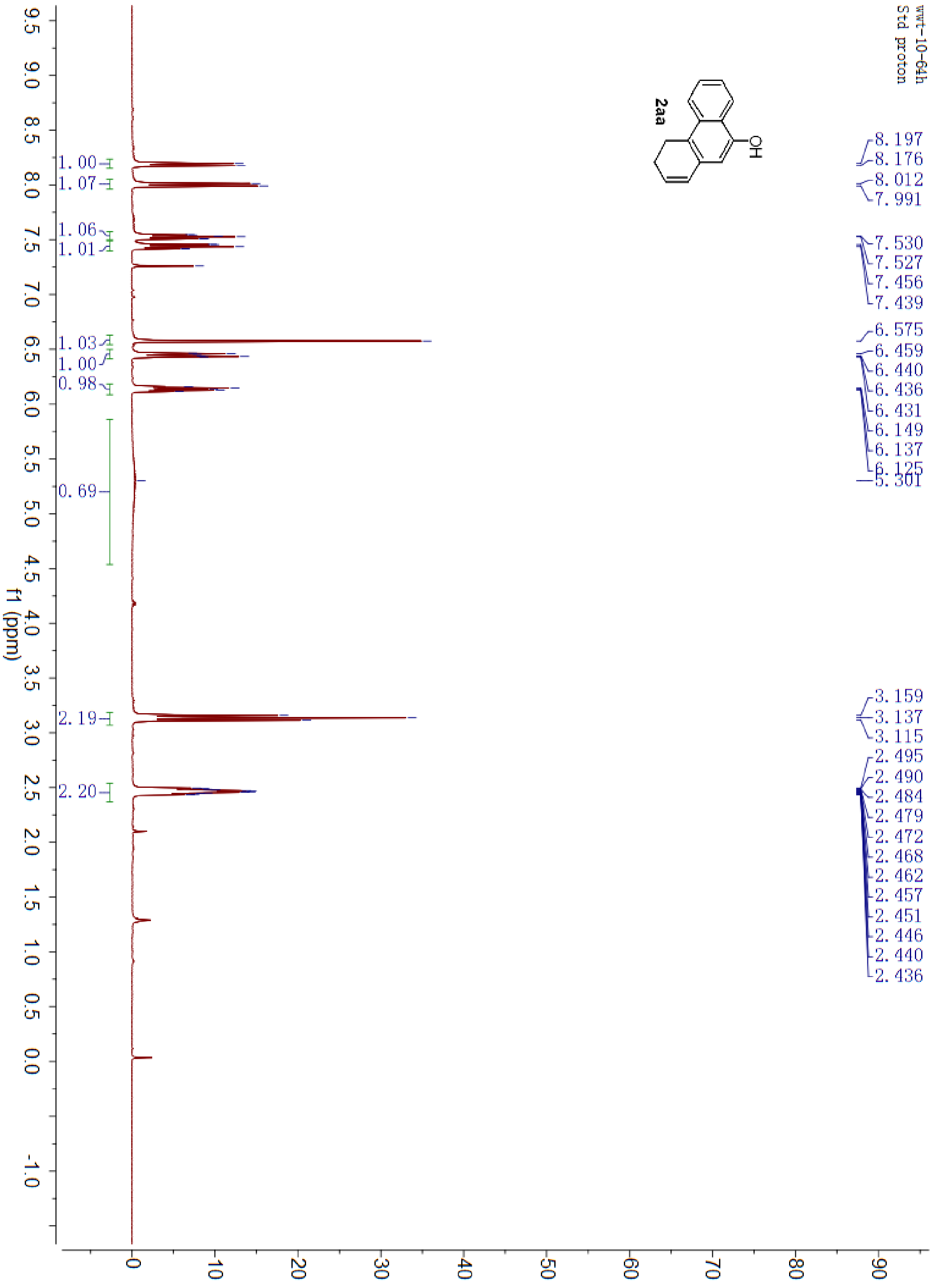

wwt-10-64h
Std proton
2aa
OH
8.197
8.176
8.012
7.991
7.530
7.527
7.456
7.439
6.575
6.459
6.440
6.436
6.431
6.149
6.137
6.125
5.301
3.159
3.137
3.115
2.495
2.490
2.484
2.479
2.472
2.468
2.462
2.457
2.451
2.446
2.440
2.436
1.00
1.07
1.06
1.01
1.03
1.00
0.98
0.69
2.19
2.20
f1 (ppm)

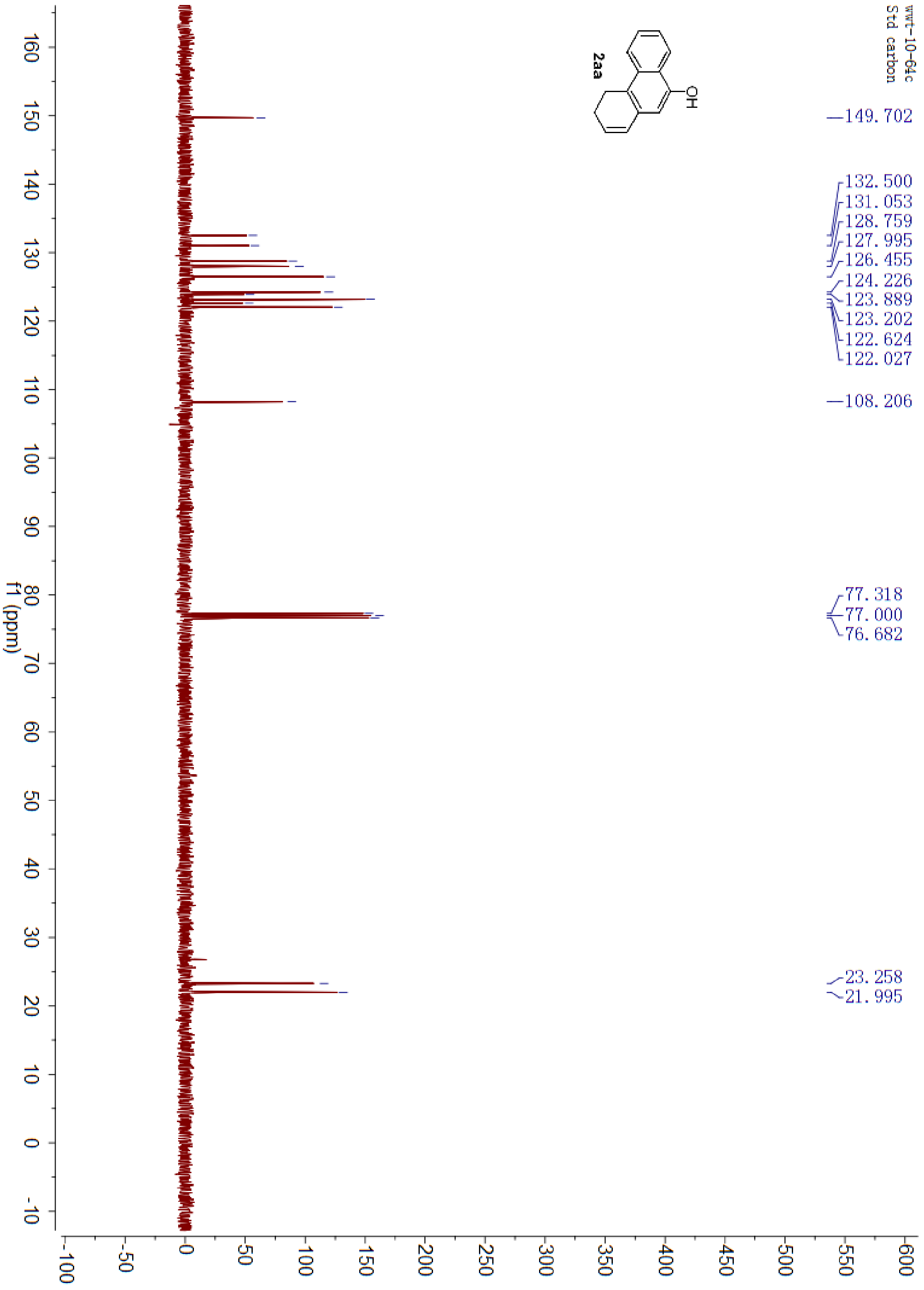

wwt-10-64c
Std carbon
2aa
OH
149.702
132.500
131.053
128.759
127.995
126.455
124.226
123.889
123.202
122.624
122.027
108.206
77.318
77.000
76.682
23.258
21.995
f1 (ppm)

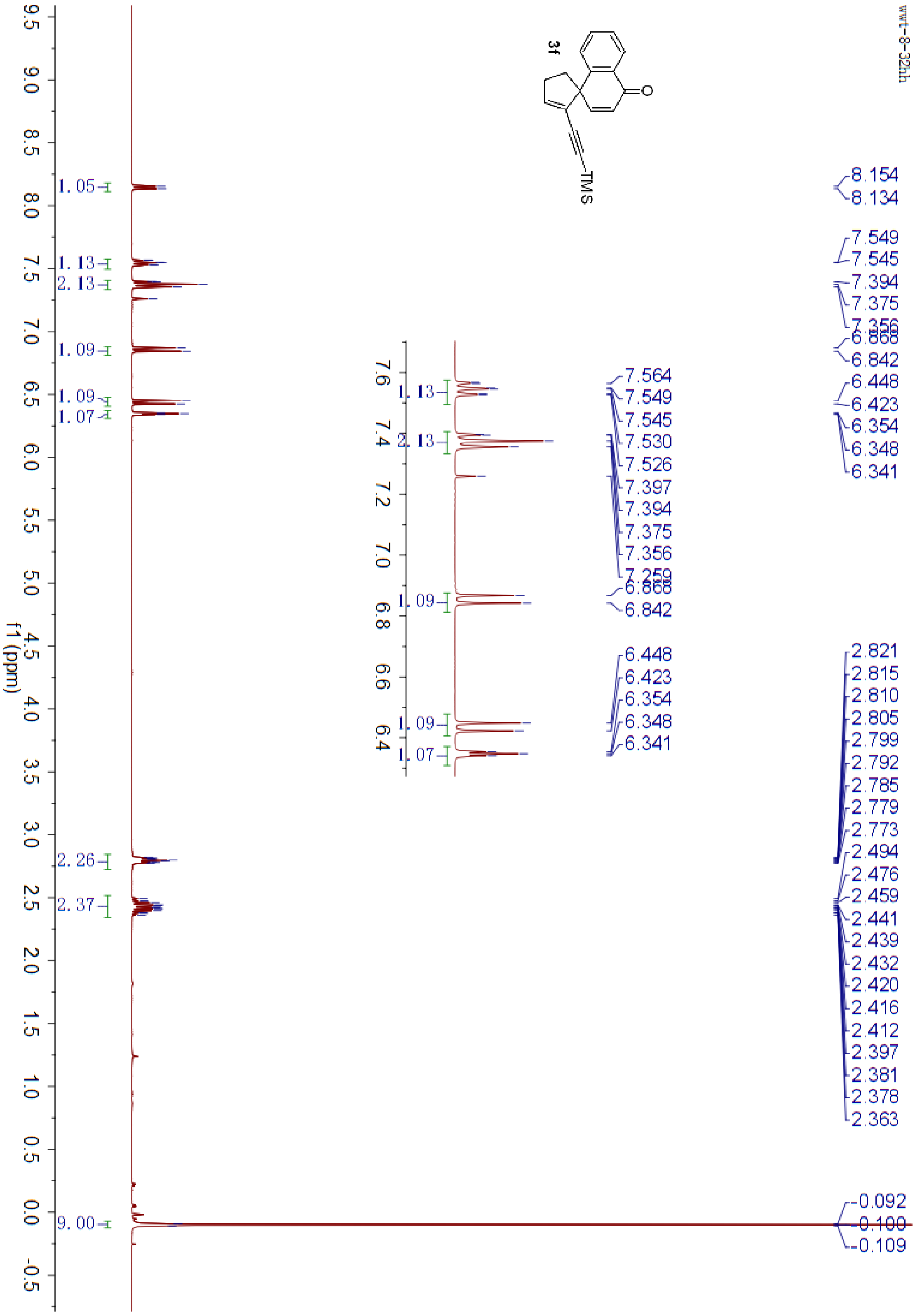
wwt-8-32hh
3f
TMS
f1 (ppm)
8.154
8.134
7.549
7.545
7.394
7.375
7.356
6.868
6.842
6.448
6.423
6.354
6.348
6.341
2.821
2.815
2.810
2.805
2.799
2.792
2.785
2.779
2.773
2.494
2.476
2.459
2.441
2.439
2.432
2.420
2.416
2.412
2.397
2.381
2.378
2.363
0.092
0.100
0.109

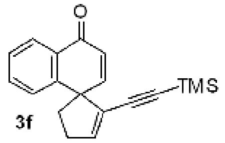
O
TMS

3f

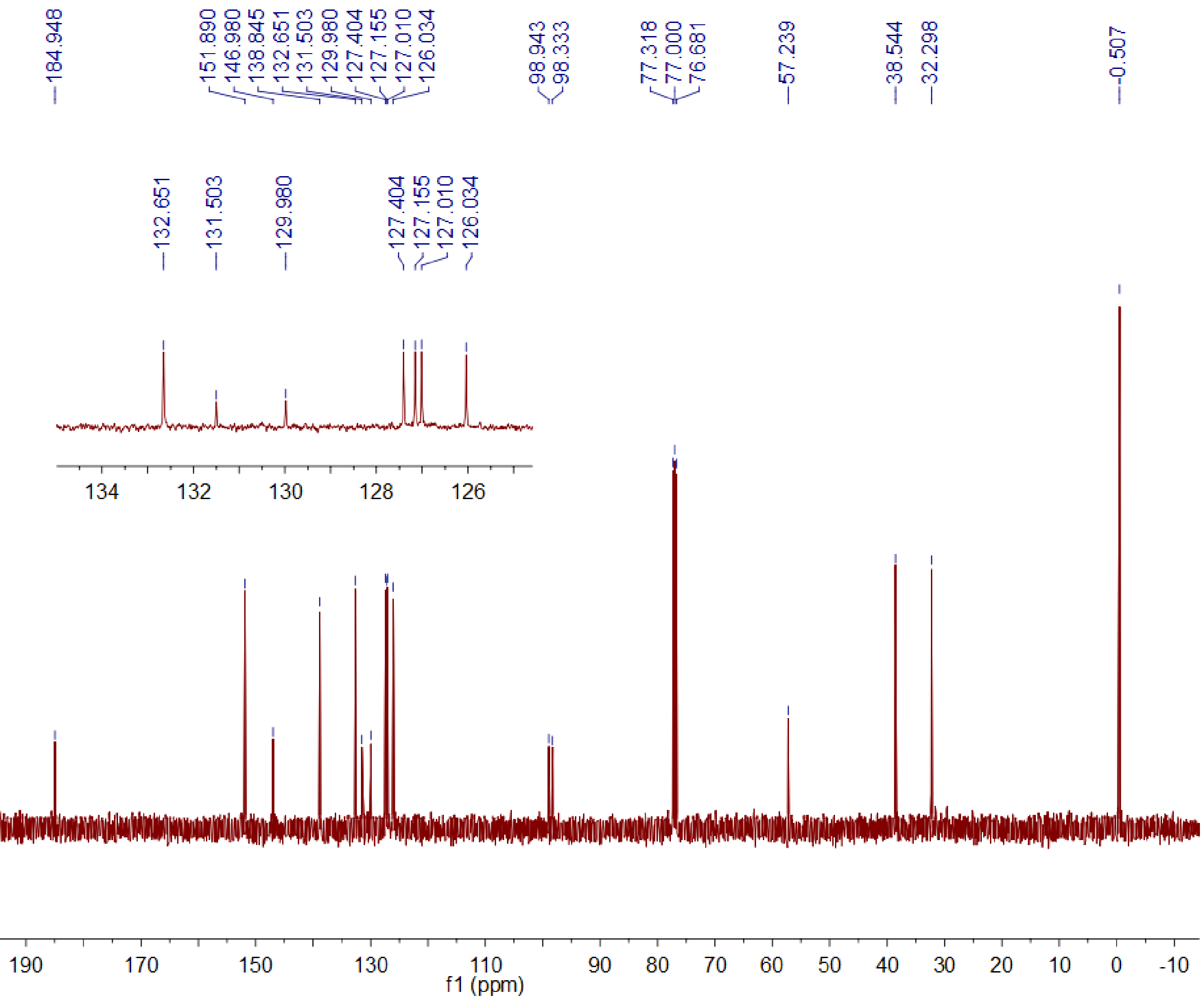
wwt-8-32-c
new experiment
184.948
151.890
146.980
138.845
132.651
131.503
129.980
127.404
127.155
127.010
126.034
98.943
98.333
77.318
77.000
76.681
57.239
38.544
32.298
-0.507
132.651
131.503
129.980
127.404
127.155
127.010
126.034
134
132
130
128
126
230
210
190
170
150
130
110
90
80
70
60
50
40
30
20
10
0
-10
f1 (ppm)

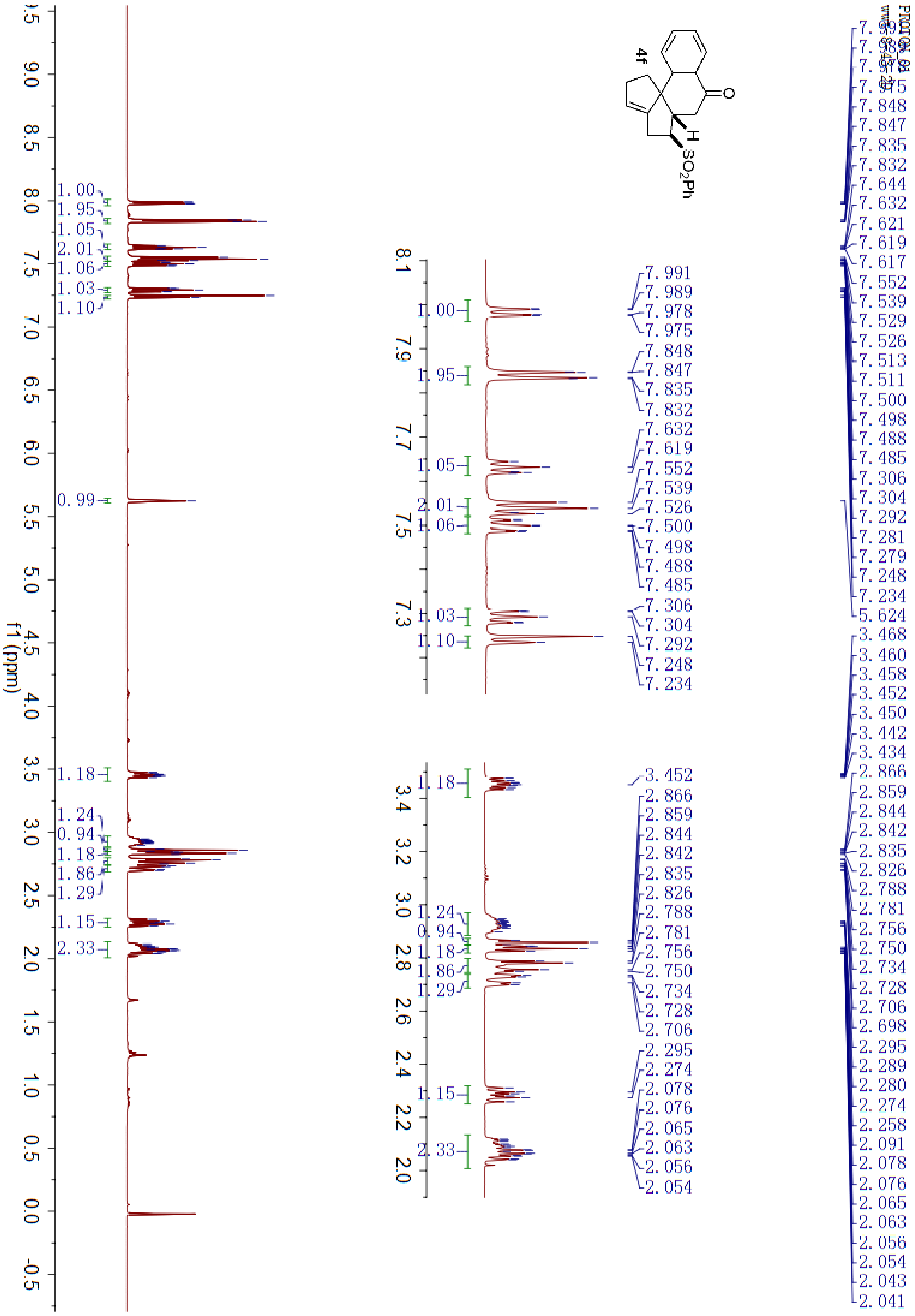

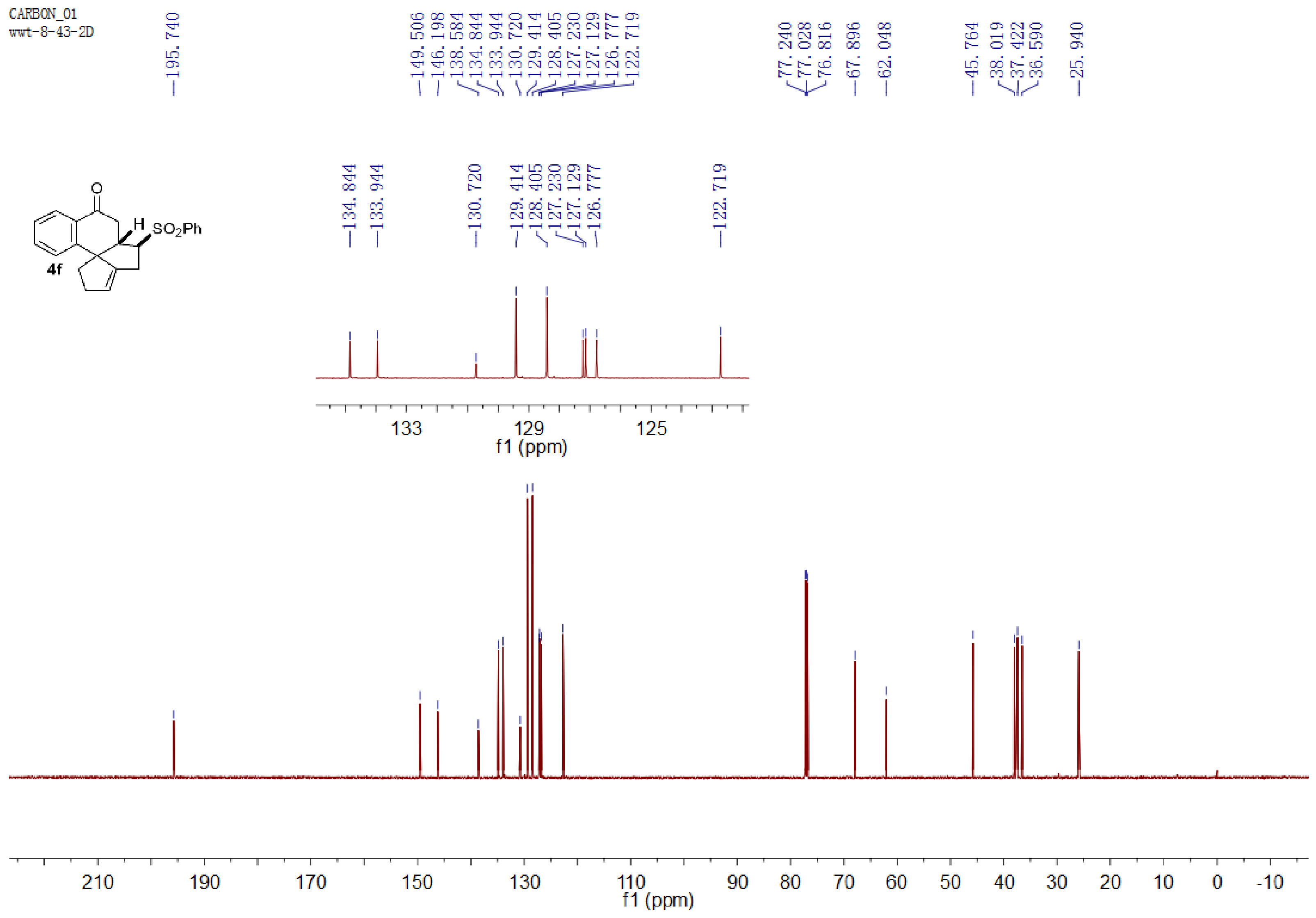
CARBON_01
wwt-8-43-2D
4f
SO2Ph
H
195.740
149.506
146.198
138.584
134.844
133.944
130.720
129.414
128.405
127.230
127.129
126.777
122.719
77.240
77.028
76.816
67.896
62.048
45.764
38.019
37.422
36.590
25.940
134.844
133.944
130.720
129.414
128.405
127.230
127.129
126.777
122.719
133
129
125
f1 (ppm)
210
190
170
150
130
110
90
80
70
60
50
40
30
20
10
0
-10
f1 (ppm)

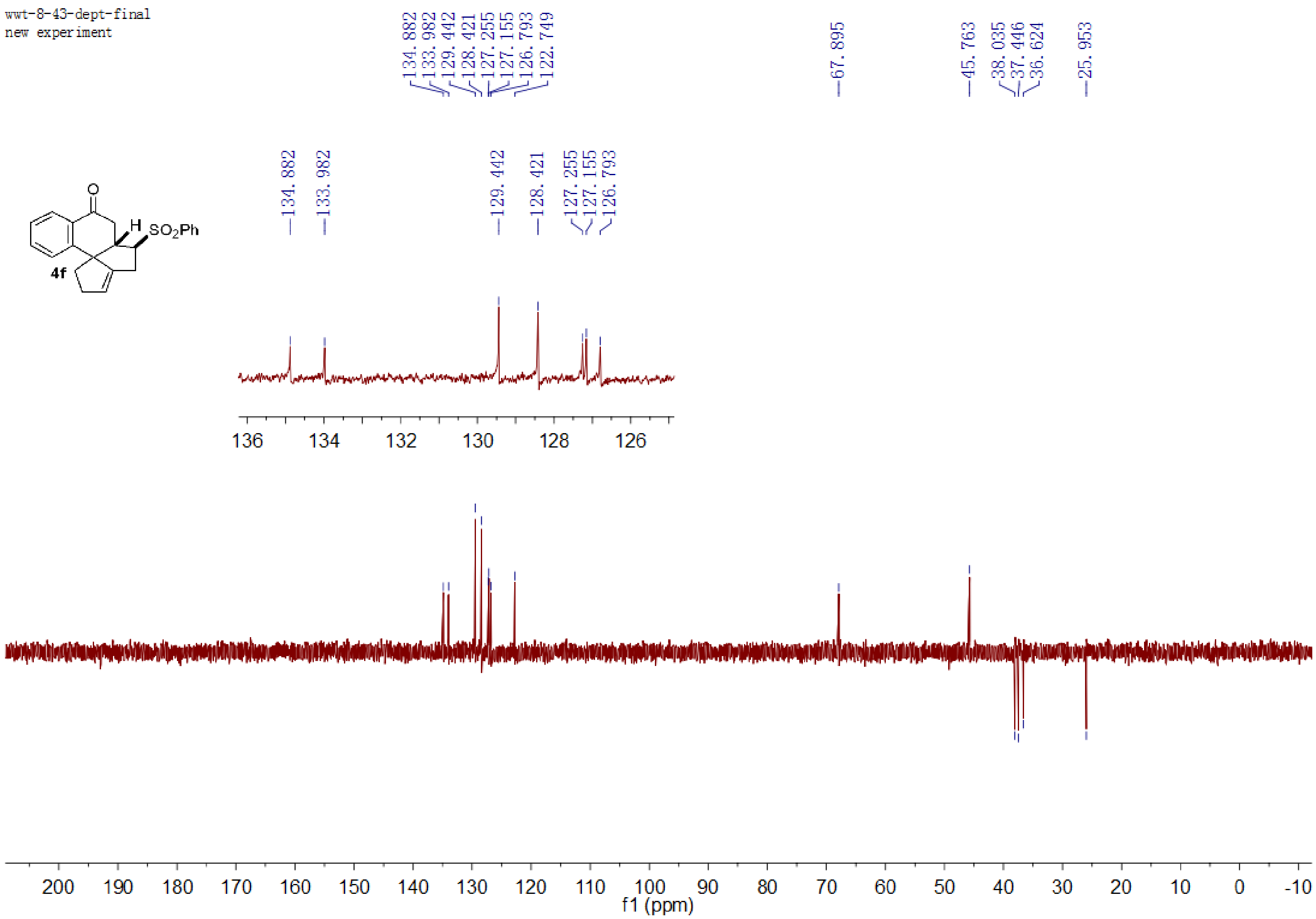
wwt-8-43-dept-final
new experiment
4f
SO2Ph
H
O
134.882
133.982
129.442
128.421
127.255
127.155
126.793
122.749
67.895
45.763
38.035
37.446
36.624
25.953
136
134
132
130
128
126
200
190
180
170
160
150
140
130
120
110
100
90
80
70
60
50
40
30
20
10
0
-10
f1 (ppm)

wwt-8-43-2D

Sample Name:
wwt-8-43-2D
Data Collected on:
OMC-NMR600-vnmrs600
Archive directory:
/home/omc/vnmrsys/data
Sample directory:
wwt-8-43-2D_20150324_01
FidFile: gCOSY_01

Pulse Sequence: gCOSY
Solvent: cdcl3
Data collected on: Mar 24 2015

Temp. 25.0 C / 298.1 K
Operator: omc

Relax. delay 1.000 sec
Acq. time 0.249 sec
Width 6188.1 Hz
2D Width 6188.1 Hz
2 repetitions
128 increments
OBSERVE H1, 599.7754542 MHz
DATA PROCESSING
Sq. sine bell 0.080 sec
F1 DATA PROCESSING
Sq. sine bell 0.021 sec
FT size 4096 x 4096
Total time 5 min 11 sec

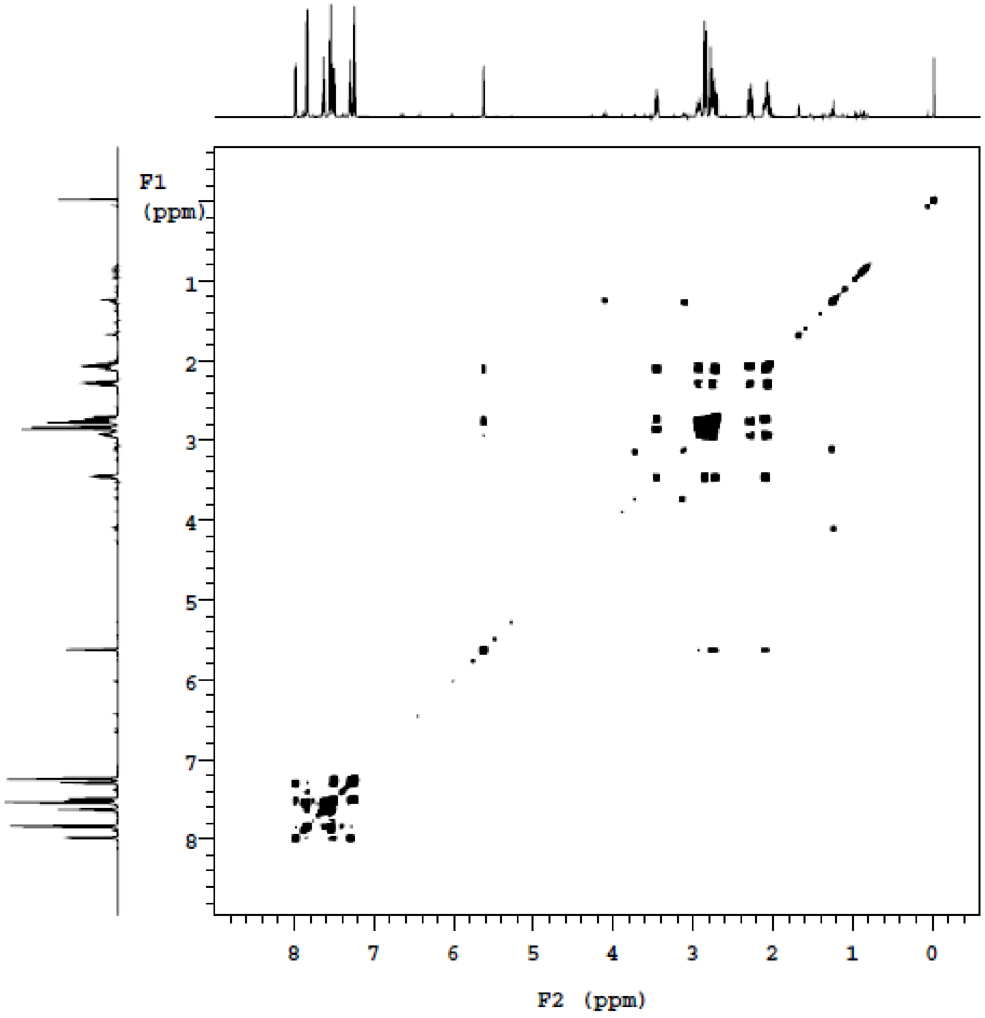

wwt-8-43-2D

Sample Name:
wwt-8-43-2D
Data Collected on:
OMC-NMR600-vnmrs600
Archive directory:
/home/omc/vnmrsys/data
Sample directory:
wwt-8-43-2D_20150324_01
FidFile: gHMBCAD_01

Pulse Sequence: gHMBCAD
Solvent: cdcl3
Data collected on: Mar 24 2015

Temp. 25.0 C / 298.1 K
Operator: omc

Relax. delay 2.000 sec
Acq. time 0.233 sec
Width 6613.8 Hz
2D Width 35445.3 Hz
8 repetitions
2 x 200 increments
OBSERVE H1, 599.7754542 MHz
DATA PROCESSING
Sq. sine bell 0.080 sec
F1 DATA PROCESSING
Gauss apodization 0.005 sec
FT size 4096 x 4096
Total time 1 hr, 59 min

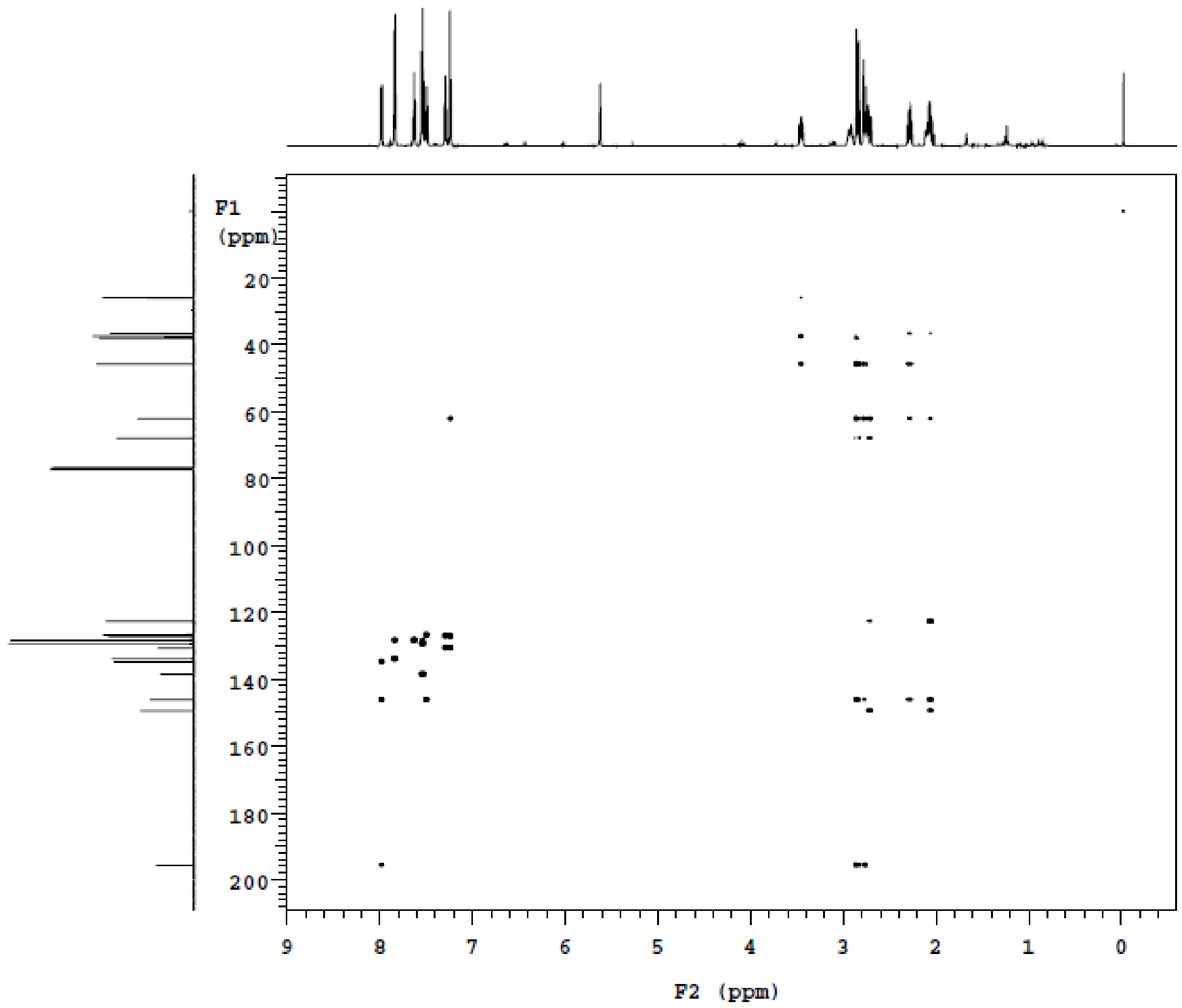

wwt-8-43-2D

Sample Name:
wwt-8-43-2D
Data Collected on:
OMC-NMR600-vnmrs600
Archive directory:
/home/omc/vnmrsys/data
Sample directory:
wwt-8-43-2D_20150324_01
FidFile: gHSQCAD_01

Pulse Sequence: gHSQCAD
Solvent: cdcl3
Data collected on: Mar 24 2015

Temp. 25.0 C / 298.1 K
Operator: omc

Relax. delay 1.000 sec
Acq. time 0.233 sec
Width 6613.8 Hz
2D Width 30165.9 Hz
8 repetitions
2 x 200 increments
OBSERVE H1, 599.7754542 MHz
DECOUPLE C13, 150.8272311 MHz
Power 44 dB
on during acquisition
off during delay
W40_OneProbe modulated
DATA PROCESSING
Gauss apodization 0.074 sec
F1 DATA PROCESSING
Gauss apodization 0.006 sec
FT size 4096 x 2048
Total time 1 hr, 4 min

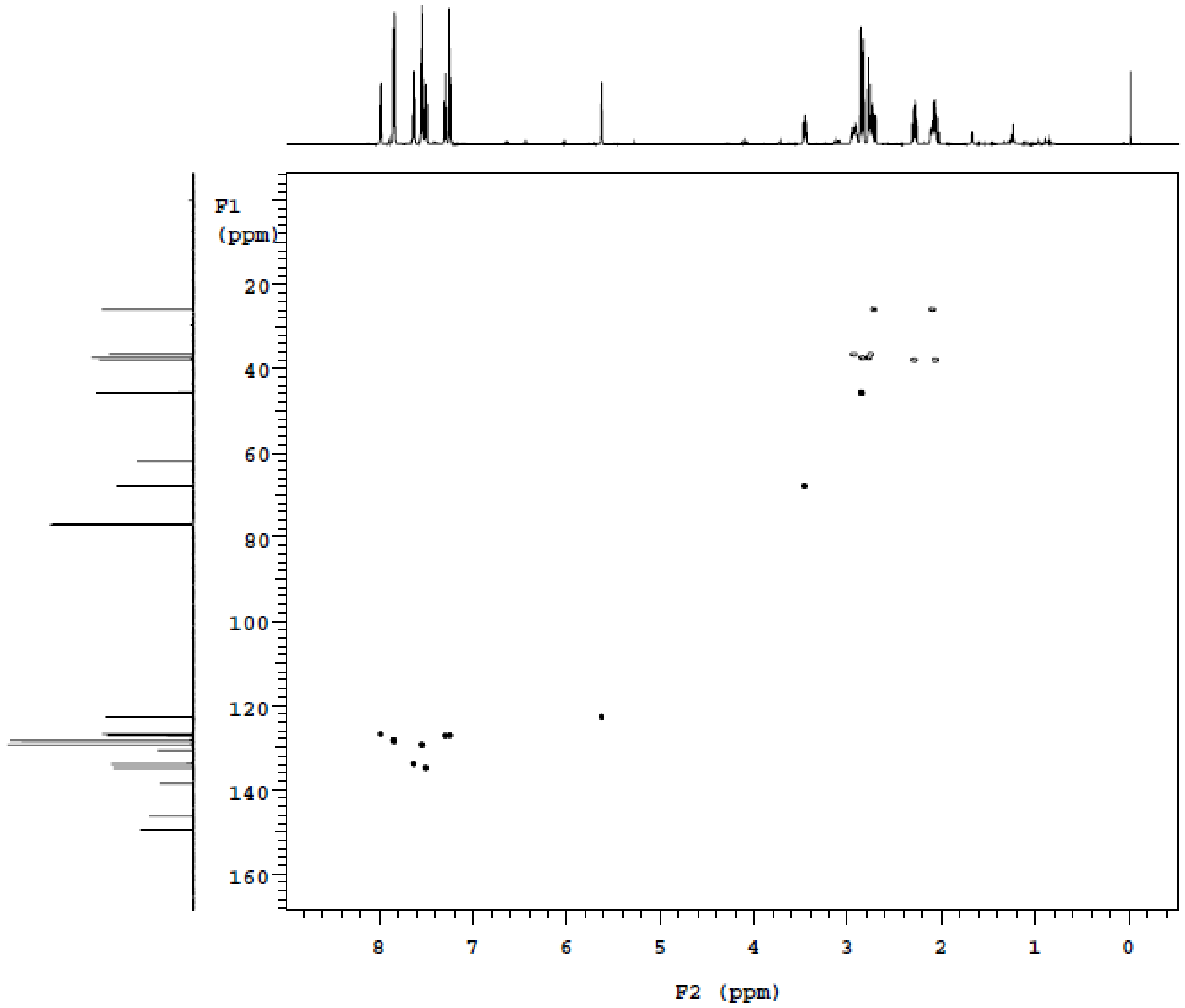

wwt-8-43-noesy

Sample Name:
wwt-8-43-noesy
Data Collected on:
OMC-NMR600-vnmrs600
Archive directory:
/home/omc/vnmrsys/data
Sample directory:
wwt-8-43-noesy_20150512_01
FidFile: NOESY_01

Pulse Sequence: NOESY
Solvent: cdcl3
Data collected on: May 12 2015

Temp. 25.0 C / 298.1 K
Operator: omc

Relax. delay 1.500 sec
Acq. time 0.251 sec
Width 6127.5 Hz
2D Width 6127.5 Hz
8 repetitions
2 x 128 increments
OBSERVE H1, 599.7754542 MHz
DATA PROCESSING
Line broadening 3.0 Hz
Gauss apodization 0.052 sec
F1 DATA PROCESSING
Gauss apodization 0.012 sec
FT size 4096 x 4096
Total time 1 hr, 17 min

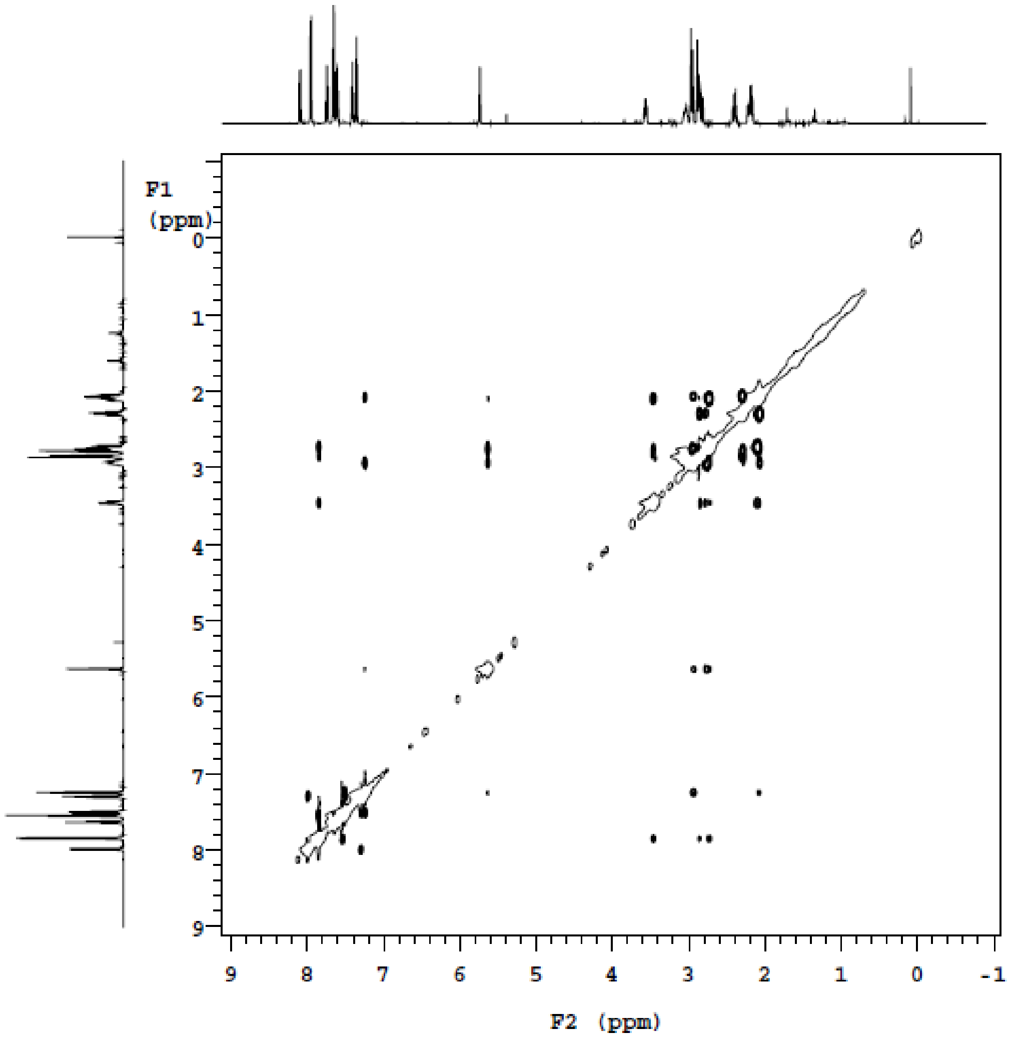

References

[1] M.-L. Tao, D.-Z. Liu, X.-Q. Zhou, *Chemical Industry and Engineering.* **2004**, *21*, 410-413.

[2] T. Miura, N. Iwasawa, *J. Am. Chem. Soc.* **2002**, *124*, 518-519.

[3] S. F. Yip, H. Y. Cheung, Z. Zhou, F. Y. Kwong, *Org. Lett.* **2007**, *9*, 3469-3472.

[4] M. Weimar, G. Dürner, J. W. Bats, M. W. Göbel, *J. Org. Chem.* **2010**, *75*, 2718-2721.

[5] W.-J. Yoo, A. Allen, K. Villeneuve, W. Tam, *Org. Lett.* **2005**, *7*, 5853-5856.
